# Polydentate *N*,*O*-Ligands Possessing Unsymmetrical Urea Fragments Attached to a *p*-Cresol Scaffold

**DOI:** 10.3390/molecules28186540

**Published:** 2023-09-09

**Authors:** Stanislava E. Todorova, Rusi I. Rusew, Boris L. Shivachev, Vanya B. Kurteva

**Affiliations:** 1 Institute of Organic Chemistry with Centre of Phytochemistry, Bulgarian Academy of Sciences, Acad. G. Bonchev Str., bl. 9, 1113 Sofia, Bulgaria; 2 Institute of Mineralogy and Crystallography “Acad. Ivan Kostov”, Bulgarian Academy of Sciences, Acad. G. Bonchev Str., bl. 107, 1113 Sofia, Bulgaria; r.rusev93@gmail.com

**Keywords:** polydentate *N*,*O*-ligands, unsymmetrical urea, fused aryloxazinones, NMR, XRD, ITC

## Abstract

In this study, three series of polydentate *N*,*O*-ligands possessing unsymmetrical urea fragments attached to a *p*-cresol scaffold are obtained, namely mono- and bi-substituted open-chain aromatics, synthesised using a common experiment, as well as fused aryloxazinones. Separate protocols for the preparation of each series are developed. It is found that in the case of open-chain compounds, the reaction output is strongly dependent on both bis-amine and carbamoyl chloride substituents, while oxazinones can be effectively obtained via a common protocol. The products are characterized via 1D and 2D NMR spectra in solution and using single-crystal XRD. A preliminary study on the coordination abilities of the products performed via ITC shows that there are no substantial interactions in the pH range of 5.0–8.5 in general.

## 1. Introduction

Solvent extraction is among the most powerful techniques applied in separation science and technology [1,2,3,4,5,6,7,8] at both the laboratory and industrial scale. In particular, synergistic extraction is an active field of research and development in modern coordination chemistry, having a great impact on metal separation and treatment and the recycling of industrial wastes [9,10,11,12,13,14,15]. The proper selection of a combination of extractants and synergists results in a multiple increase in the extraction efficiency and the better separation of metals. Therefore, the discovery of new extraction systems is a topical and rapidly evolving trend in modern chemistry.

Polydentate molecules are of growing interest as they are widely applied as scaffolds in the combinatorial synthesis of artificial receptors for ions with medical and environmental potential [16,17,18,19]. Among the broad variety of synthetic compounds, polyoxaaza ligands have received special attention due to their outstanding coordination abilities [20,21,22,23,24,25,26,27,28,29,30,31,32]. Compounds with urea moieties in particular have shown a wide range of applications in various fields such as polymers, agrochemicals and pharmaceuticals, as well as good biological activity profiles [33,34,35,36,37,38,39,40]. However, the direct synthesis of asymmetric urea possesses a serious drawback, namely the formation of unwanted symmetric urea. Therefore, synthesis efforts nowadays are directed towards developing efficient indirect protocols.

**Figure 1:**
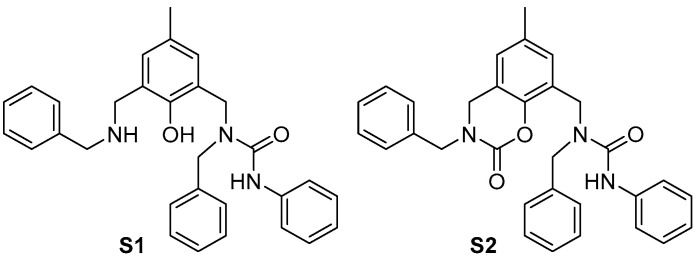
Structures of the ligands **S1** and **S2**.

Recently, we reported on the efficiency of two novel polydentate ligands, shown in Figure 1, as synergists in the isolation and separation of metal ions [41]. These ligands possess unsymmetrical urea fragments attached to a *p*-cresol scaffold and can be generally divided into open-chain substituted aromatics (**S1**) and fused aryloxazinones (**S2**). The concept was to design polydentate ligands with variable coordination abilities, controlled according to differences in the substitution pattern and geometry of the molecules [42]. The compounds were obtained in a common experiment and were isolated from the complex mixture with a low overall yield [43].

Herein, we report on the optimization of separate synthetic protocols for each ligand series, solution and solid-state characterization, and report a preliminary study on the coordination properties of the products.

## 2. Results and Discussion

**Scheme 1:**
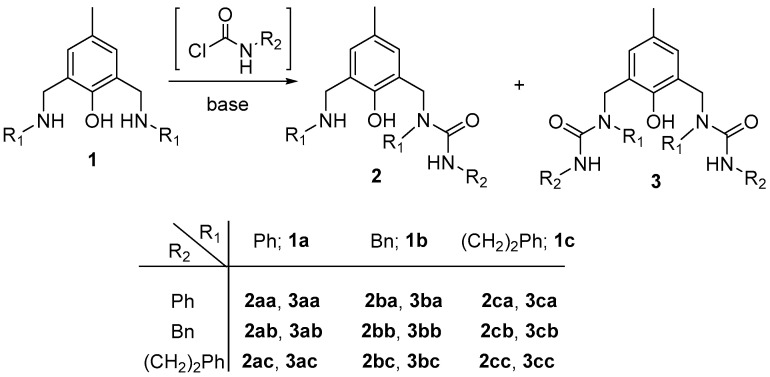
Synthesis of ligands **2** and **3**.

Our efforts were initially directed towards open-chained ligands due to the observed diverse extraction efficiency of **S1** towards various metal ions [41]. Starting bis-amines were obtained by grinding commercially available 2-hydroxy-5-methyl-1,3-benzenedicarboxaldehyde and primary amine in solventless conditions, followed by sodium borohydride reduction. As reported [43], the compounds **S1** and **S2** were isolated from a very complex mixture obtained via the direct reaction of the corresponding *p*-cresol-based secondary bis-amine (**1b**), phosgene as toluene solution and aniline in the presence of pyridine as a base. In an attempt to override the formation of oxazinone-ring-containing compounds, the reaction among bis-amine **1**, phosgene and primary amine was performed as a two-step protocol; the initial formation of carbamic chloride and the subsequent reaction with bis-amine (Scheme 1).

Three types of *N*-substituents were chosen, namely phenyl, benzyl and phenethyl, i.e., aryl, benzyl and alkyl, in order to tune the nitrogen basicity and steric flexibility. The conditions were varied and it was found that the optimal conditions for the predominant formation of product **2** or **3** are different for each example, and that the reaction out-put is strongly dependent on the base, reagents’ proportions and solvent; meanwhile, prolongation and dilution have no significant impact. Selected results are summarized in Table 1.

**Figure 2:**
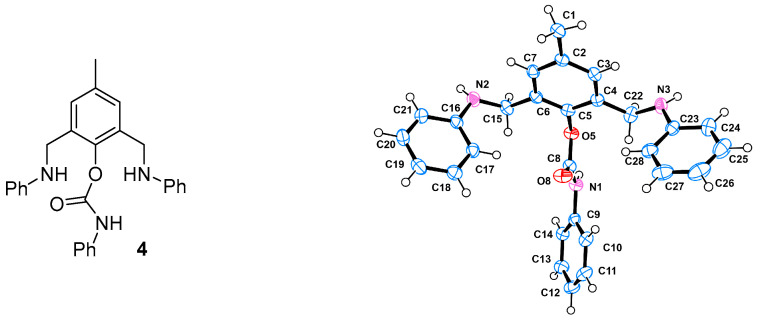
Structure and ORTEP view of the *O*-acylated product **4**.

**Figure 3:**
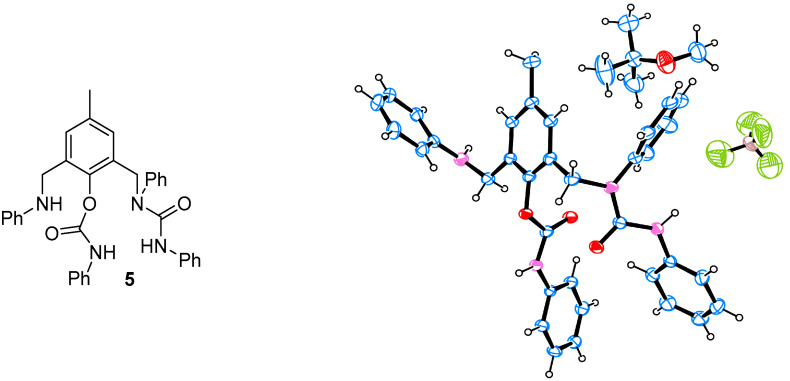
Structure and ORTEP view of the *O*,*N*-diacylated product **5**.

The transformation performed between aniline-derived bis-amine (**1a**) and phenylcarbamic chloride in the presence of pyridine as a base led to the formation of two main products, isolated in a moderate overall yield (up to 57%); mono- and bis-acylated ligands **2aa** and **3aa** (Scheme 1, Table 1), i.e., only *N*-acylated products. On the contrary, the oxygen was also attacked when using *N*,*N*-diisopropylethylamine (DIPEA), leading to the formation of compounds **4** (Figure 2 and Figure S1) and **5** (Figure 3), together with **2aa** in a 51% overall yield (entry 1). For this reason, all further experiments were performed in the presence of pyridine as a base.

As seen in Table 1, increasing the carbamic chloride portion leads to an increase in the percentage of product **3** in general, while the solvent effect is dependent on the *N*-substituents. The best conversion is achieved in toluene, except in ligands **2ac**/**3ac**, where the overall yield is higher in dichloroethane (entries 9 vs. 10), **2ba**/**3ba** (entries 12 vs. 13) and **2cc**/**3cc** (entries 26 vs. 28), and where the results in toluene and benzene are commensurable. However, the best conditions for the preparation of a particular ligand, **2** or **3**, are more sensitive to the solvent used. All carbamic chlorides operate the most effectively in toluene, except for a few examples. Phenylcarbamic chloride is the most effective in benzene only for the ligand **2ba** (entry 12). Similar conversions with benzylcarbamic chloride are achieved for **2cb** in toluene and dichloroethane (entries 22 vs. 25). When using phenylethylcarbamic chloride, benzene is the right solvent for **2bc** (entry 16) and **2cc** (entry 26), dichloroethane is the right solvent for **3ac** (entry 10), while the yields of **3cc** are identical in toluene and dichloroethane (entries 28 vs. 29). The best results for all bis-amines are also obtained in toluene, with few exceptions. Starting from **1a,** dichloroethane is the preferable solvent only for ligand **3ac** (entry 10). The best yields from **1b** are obtained in benzene for ligands **2ba** (entry 12) and **2bc** (entry 16). When starting from **1c,** benzene is the right solvent for **2cc** (entry 26), whereas the results in toluene and dichloroethane are comparable for ligands **2cb** (entries 22 vs. 25) and **3cc** (entries 28 vs. 29).

**Figure 4:**
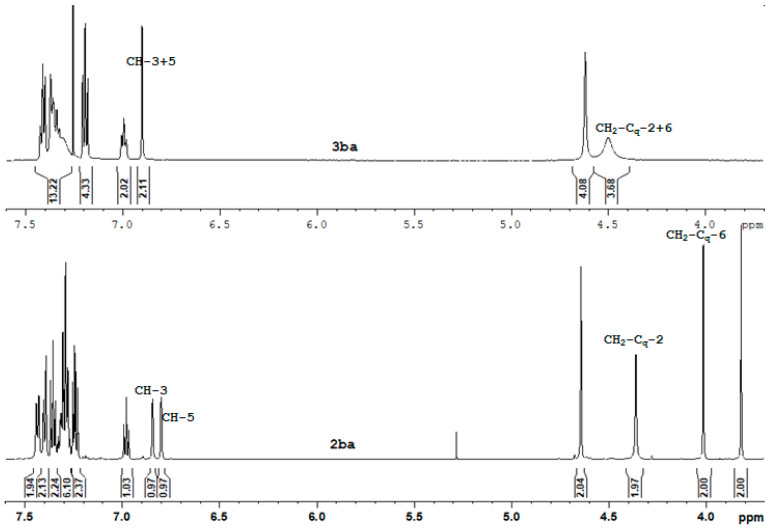
^1^H NMR spectra of products **2ba** and **3ba**.

The structures of the products are assigned using 1D and 2D NMR spectra and confirmed via single-crystal XRD of selected samples. The NMR spectra show characteristics for each series pattern (Table 2). Separate signals are observed for each group in compounds **2**, while all groups of both *p*-cresol scaffold and substituents show common signals in symmetrical molecules **3**, the effect being the most discernible for CH protons of *p*-cresol scaffold (*CH*-3 and *CH*-5) and methylene groups, which are in an area free of other signals. The latter is demonstrated in the example of the **1b**-derived ligands **2ba** and **3ba,** as shown in Figure 4.

At the same time, the chemical shifts in some signals are strongly dependent on the substitution pattern (Table 2). The proton resonances for *CH*-3 and *CH*-5 of the *p*-cresol scaffold are shifted upfield and downfield, respectively, in mono-substituted ligands obtained from **1a** (R_1_ = Ph, **2aa**, **2ab**, **2ac**); meanwhile, in bi-substituted analogues, the signals are in between. For the rest of the ligands (R_1_ = Bn, phenethyl), the chemical shifts in the two signals in the spectra of ligand **2** and the signal in the spectra of **3** possess very close values. The signals for both bridged methylene groups (C*H_2_*-C_q_-2 and C*H_2_*-C_q_-6) are shifted upfield in the spectra of all ligands of **2** with respect to the corresponding signal in **3**. The carbon resonances for CH-3 and CH-5 are slightly shifted downfield in **3** with respect to **2** in all couples, except in ligands **2ab**/**3ab** and **2ac**/**3ac**, in which the signal of compound **3** is in between those of the corresponding ligand **2**; meanwhile, that for the methylene group of **3** is in between both signals in the spectra of **2** in all examples.

**Figure 5:**
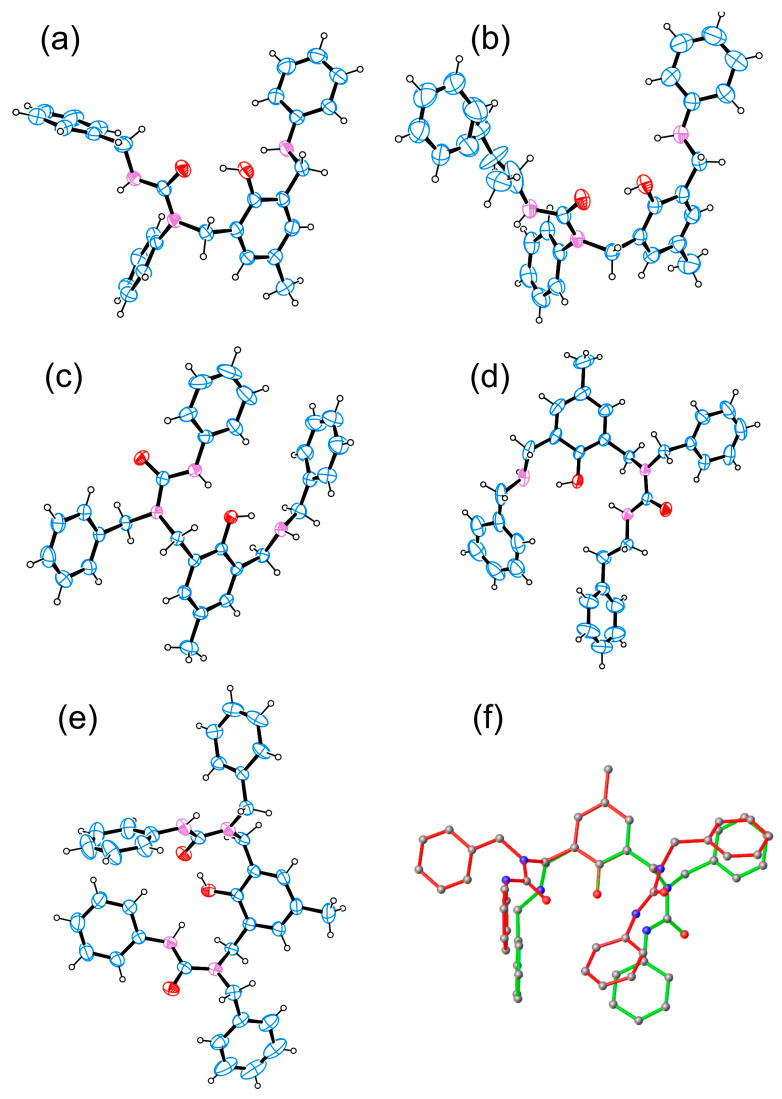
ORTEP view of the products: (**a**) **2ab**, (**b**) **2ac**, (**c**) **2ba**, (**d**) **2bc**, (**e**) **3ba**; and (**f**) overlay of the two independent molecules in the asymmetric unit of **2ba** (green) and **3ba** (red).

Several phases appropriate for single-crystal XRD were grown and analysed. The ORTEP views are shown in Figure 5. A comparison between the two types of ligand shows that the fragments bearing unsymmetrical urea moiety possess conserved molecular geometry, while the remaining part(s) of the molecules seems to be more flexible and lead to variations in the molecular geometry and distance between the coordination centres. The latter is illustrated in the example of ligands **2ba** and **3ba** in Figure 5f.

**Figure 6:**
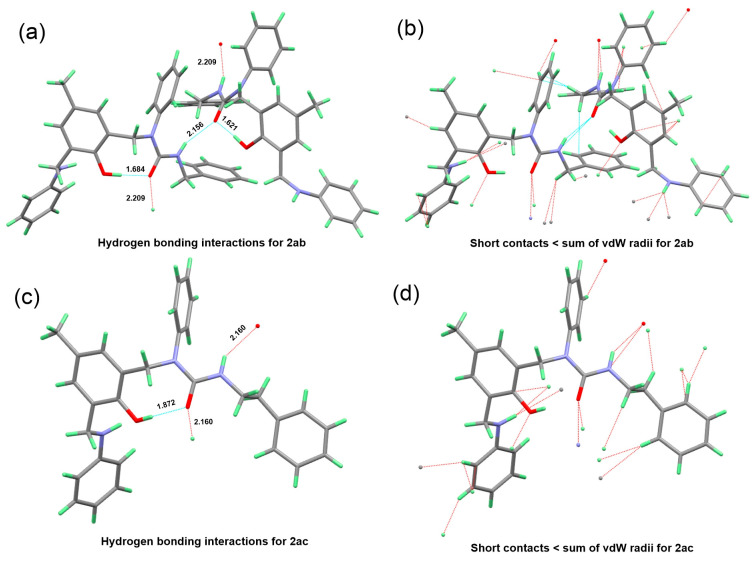
Visualization of the hydrogen bonds (**a**,**c**) and weak interactions (**b**,**d**) in **2ab** and **2ac,** respectively.

Compound **2ab** crystallizes in the monoclinic *Cc* space group with two molecules in the ASU. The overlay of the two molecules provides an RMSD of 1.0635 Å (Figure S2a), and shows that the molecular geometry differs due to the flexibility of the side chains and the observed rotation of the terminal benzyl moieties around the N–C bond, e.g., rotamers or conformational isomerism is plausible. For both molecules, an intramolecular O–H…O hydrogen bond stabilizes the molecular geometry. A classical N–H…O interaction between adjacent molecules produces C^2^_2_(8) chains, propagating along the *c* axis. Although several weak interactions, namely C–H…O, N–H…π (Table S6, Figure 6a,b), are detected, the three-dimensional packing molecules of the molecules of **2ab** produce pseudo-layers stacked along *a* (Figure S2b).

**Figure 7:**
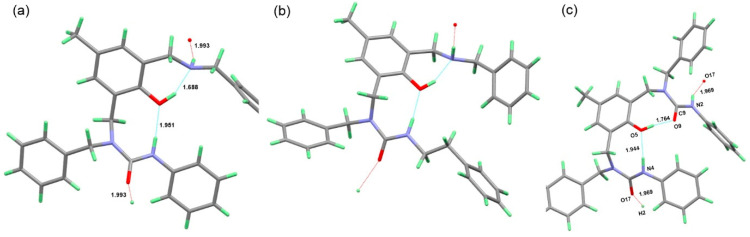
Observed hydrogen bonds in (**a**) **2ba**, (**b**) **2bc** and (**c**) **3ba**.

Compound **2ac** crystallizes in the orthorhombic *Pca*2_1_ space group with one molecule in the ASU. The hydrogen bonding interactions are similar to those observed in compound **2ab**: one intramolecular O5-H5…O9 and intermolecular N2-H2…O9 (Figure 6c and Table S7)**.** The intermolecular hydrogen bond again produces a chain motif C^1^_1_(4) propagating along *a*. Again, the presence of an aromatic ring in the molecule of **2ac** is responsible for several weak interactions (Figure 6). The combination of hydrogen bonds and a weak interaction produces pseudo-layers stacked along c (Figure S3). The hydrogen bonding patter in **2ba**, **2bc** and **3ba** is analogous to **2ab** and **2ac**. The basic difference is the presence of two intramolecular hydrogen bonds instead of one. The intermolecular interaction again produces chains with graphsets C^1^_1_(10) for **2ba**, **2bc** and C^1^_1_(12) **3ba** (Figure 7 and Tables S8–S10). The three-dimensional arrangement of the molecules again produces pseudo-layers, as shown in Figure S4.

**Scheme 2:**
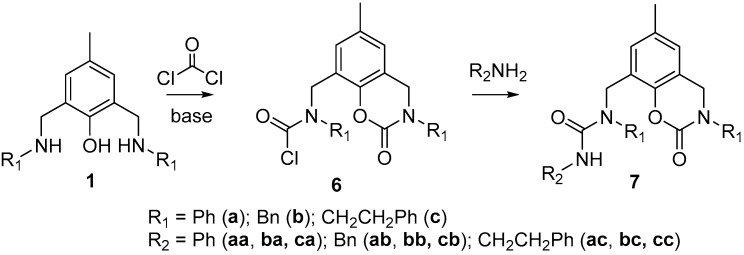
Synthesis of ligands **7**.

A two-step sequence was chosen for the preparation of fused aryloxazinone ligands with an unsymmetrical urea fragment (**7**). Bis-amines **1** were submitted to a reaction with phosgene solution (Scheme 2). The proportions, base, and reaction duration were varied and it was found that two equivalents of phosgene, pyridine as a base, and a 2 h reaction time at room temperature are the optimal conditions. The intermediate products **6** were easily isolated via chromatography in 40–50% yields. Further prolongation did not result in better conversion, while the use of equimolar amounts of the reagents significantly decreased the yields.

**Figure 8:**
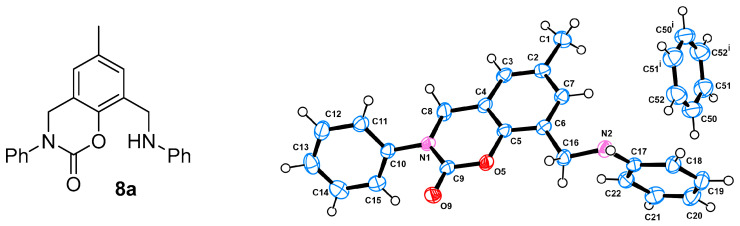
The structure and ORTEP drawing of compound **8a;** symmetry operation ^(i)^ *x*, *2–y*, *1–z*.

It has to be noted that a side-product was isolated in a low yield (2%) from the reaction of **1a** in parallel with chloride **6a**, whose structure was determined to be, via NMR and XRD, amino oxazinone **8a** (Figure 8), the precursor of **6a**. Based on this observation, it can be suggested that phenol oxygen is initially attacked by phosgene, followed by cyclization and *N*-acylation.

The second step was performed in different proportions and with different bases, solvents, temperatures, and reaction durations. Finally, the conditions were optimized and the products were isolated via chromatography in a 87% yield after 19 h of stirring at 50 °C in dichloroethane with 2.5 equivalents of primary amine, in the absence of another base. The results are summarized in Table 3.

**Figure 9:**
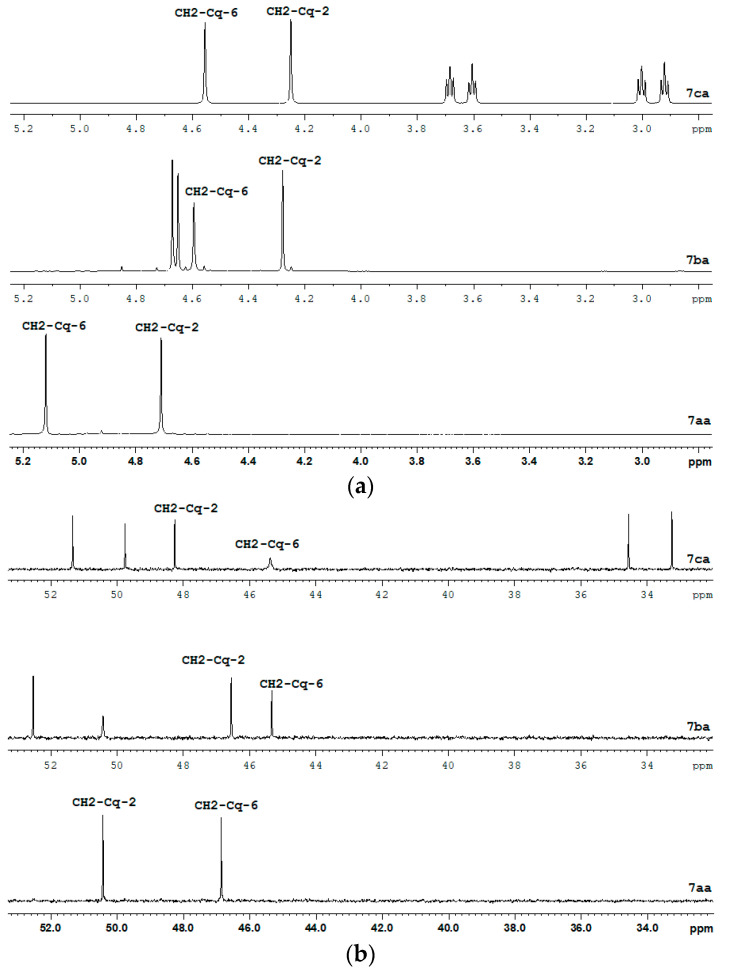
Methylene groups’ area of the ^1^H (**a**) and ^13^C (**b**) NMR spectra of products **7aa**, **7ba** and **7ca**.

The structures of the products were assigned using 1D and 2D NMR spectra. The chemical shifts in the bridge methylene (C*H*_2_-C_q_-2 and C*H*_2_-C_q_-6) and skeleton methyne (CH-3 and CH-5) groups are dependent on the substitution pattern within the series, the effect being more significant on methylene group resonances (Table 4). The influence of the chloride **6** substituent (R_1_) is inessential in general, while that of primary amine (R_2_) causes substantial shifts in particular signals. The latter is illustrated for the ligands obtained with **6a**–**c** and aniline in Figure 9.

**Figure 10:**
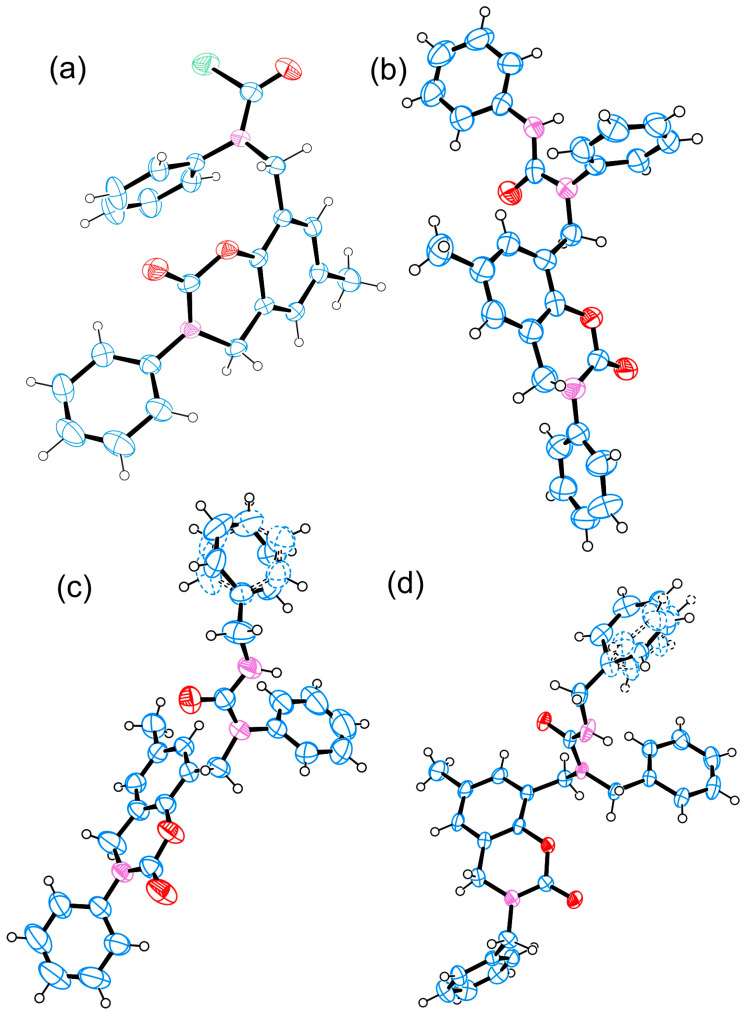
ORTEP view of the products **6a** (**a**), **7aa** (**b**), **7ab** (**c**), and **7bb** (**d**).

**Figure 11:**
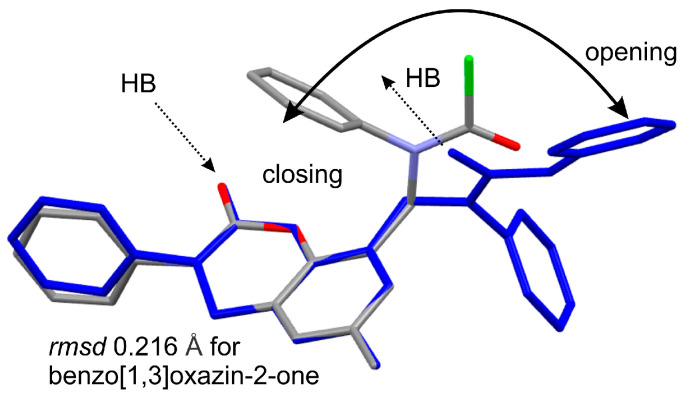
Overlay of the two independent molecules in the asymmetric unit of **6a** (grey) and **7aa** (blue).

**Figure 12:**
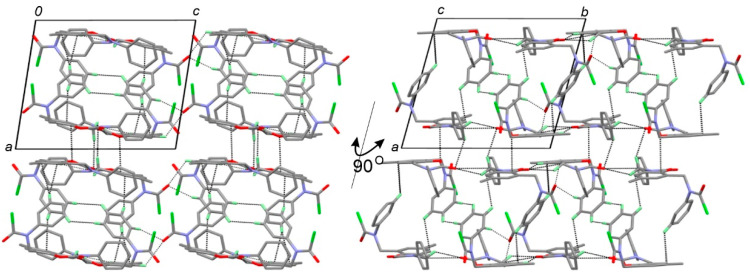
Visualization of the weak interactions in the structure of **6a**.

**Figure 13:**
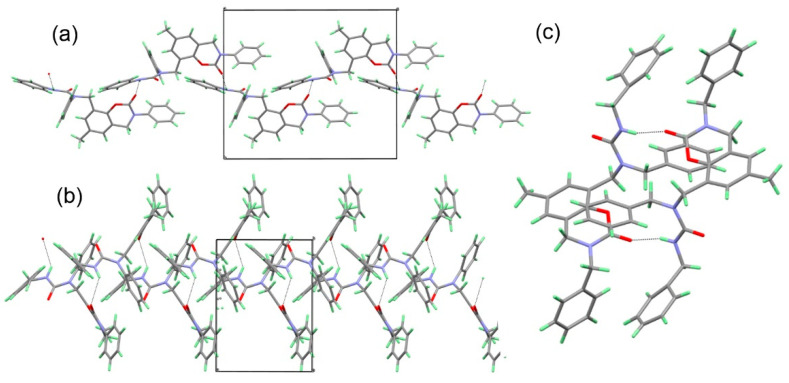
Visualization of the molecular interactions in (**a**) **7aa**, (**b**) **7ab**, and (**c**) **7bb**.

The structures of the products were confirmed via the single-crystal XRD of selected samples, as shown in Figure 10. The overlay of the molecules present in the ASU of the crystal structures revealed that the geometry of the benzoxazinone unit is highly conserved (Figure 11). In **6a,** only an acceptor (C=O) is available and the molecular geometry minimizes its surficial area/interactions by closing on itself in order to promote halogen bonding and π…π/CH_3_ interactions (Figure 12). In **7aa, 7ab**, and **7bb,** a donor and acceptor are present and the crystal structure stabilization is “dominated” by a hydrogen-bonding NH…O interaction. In **7aa** and **7ab,** neighbouring molecules produce a zig-zag chain with a C^1^_1_(10) graphset (Figure 13a,b, Tables S11 and S12), while in **7bb,** the formation of a dimmer is preferred (R^2^_2_(20), Figure 13c, Table S13).

**Figure 14:**
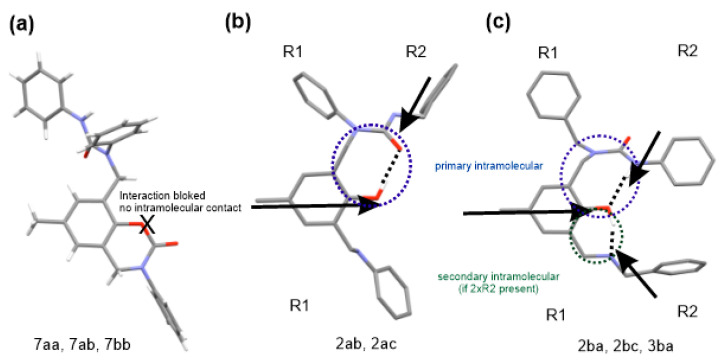
Interactions in selected samples of the products: (**a**) oxazinones, **7aa**, **7ab** and **7bb**; (**b**) open-chain compounds, **2ab** and **2ac**; (**c**) open-chain compounds, **2ba**, **2bc** and **3ba**.

A comparison between the geometry of the three types of ligands, namely **2**, **3** and **7**, shows that the open-chain substituted compounds are oriented towards the optimal intramolecular H-bonding of the urea’s heteroatoms, while the preferred geometry of oxazinones is driven by intermolecular bonding, as illustrated in Figure 14. At the same time, two types of H-bonding are observed in the open-chain compounds. In some products, like **2ab** and **2ac**, H-bonding involves hydroxyl proton and carbonyl oxygen (Figure 14b), while hydroxyl proton and nitrogen are bonded in others (Figure 14c).

**Figure 15:**
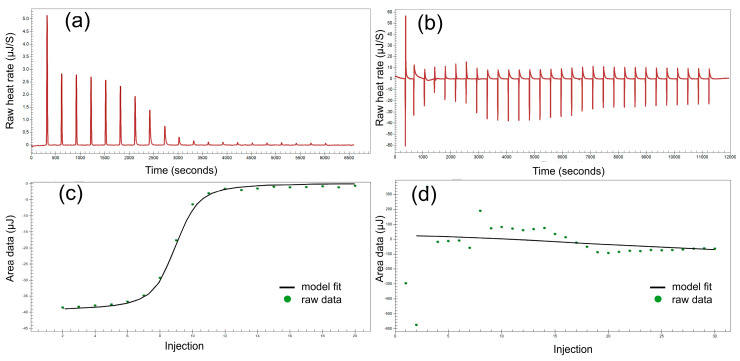
Typical calorimetric titration isotherms of (**a**) the binding interaction between **2ba** and Pb^2+^, along with the fitted association constant; (**b**) titration isotherm showing no interaction between **7ab** and Ca^2+^; (**c**) successful model fit for **2ba** and; (**d**) unsuccessful attempt to model the interaction for **7ab**.

Finally, a preliminary study on the coordination abilities of the products was performed by using isothermal titration calorimetry (ITC). This test is a sensitive and effective method that can provide information about complexation reactions and hence the strength of metal–ligand coordination. In this work, ITC is employed to detect the interactions of calcium (II), lead (II) and potassium (I) ions with the synthesized polydentate *N*,*O*-ligands (Table S6) at an approximately neutral pH range (5.0–8.5). The latter is chosen in an attempt to keep the conditions as green as possible. Typical representations of the ITC data showing interaction and a lack of interaction are shown in Figure 15a,b, respectively.

The remaining data are given in Table S14 (Supplementary Material). The fitted association constants of the interaction of compounds **2ba**, **2bc**, **2cb**, **3bb**, and **3cc** with metals are provided in Table 5 and illustrated in Figures S5–S10.

The ITC data reveal that most of the compounds do not interact with the particular metal ions tested. Only a few unveil interactions with a sensible Ka strength, mostly in slightly acidic conditions. The latter shows that the ligands likely need more acidic or more basic media to bind metal ions. This suggestion outlines a possible direction for further study regarding the interaction of compounds in a more broad pH range with an enlarged panel of metal ions.

## 3. Materials and Methods

### 3.1. General

All reagents were purchased from Aldrich, Merck and Fluka, and were used without any further purification. The deuterated solvents were purchased from Deutero GmbH. Fluka silica gel (TLC-cards 60778 with fluorescent indicator 254 nm) was used for TLC chromatography and R_f_-value determination. Merck Silica gel 60 (0.040–0.063 mm) was used for the flash chromatography purification of the products. The melting points were determined in capillary tubes using the SRS MPA100 OptiMelt (Sunnyvale, CA, USA) automated melting point system with a heating rate of 1 °C per min. The NMR spectra were recorded on the Bruker Avance II+ 600 spectrometer (Rheinstetten, Germany) in CDCl_3_; the chemical shifts were quoted in ppm in δ-values against tetramethylsilane (TMS) as an internal standard, and the coupling constants were calculated in Hz. The assignment of the signals was confirmed by applying two-dimensional COSY, NOESY, HSQC and HMBC techniques. The spectra were processed using the Topspin 2.1 program. The turbo spray mass spectra of compounds **2aa**, **3ba**, **6a**, **4**, **7aa**, **7ab**, and **7bb** were obtained using API 150EX (AB/MAS Sciex), and the ESI spectra of compounds **3aa**, **2ab**, **3ab**, **2ac**, **3ac**, **2bb**, **3bb**, **2bc**, **3bc**, **2ca**, **3ca**, **2cb**, **3cb**, **2cc**, **3cc**, **5**, **6c**, **7ac**, **7bc**, **7ca**, **7cb**, and **7cc** were obtained using the Single Quadrupole Liquid Chromatograph Mass Spectrometer Shimadzu LCMS-2020. The spectra were processed using Xcalibur Free Style program version 4.5 (Thermo Fisher Scientific Inc., Waltham, MA, USA).

The characterization of compounds **2ba** and **7ba** is given in ref. [43]. Compound **6b** was an object of individual further study and its characterization will be published in due course.

### 3.2. General Procedure for the Preparation of Bis-Amines **1**

*Step 1:* A mixture of 2,6-diformyl-4-methylphenol (657 mg; 4 mmol) and its corresponding amine (8.1 mmol) was ground in a mortar. In the case of solid bis-imines, the solid phase formed was triturated with hexane.

2,6-bis((phenylimino)methyl)-4-methylphenol: 92% yield; reddish solid; m. p. 118.1–118.3 °C (lit. 99–100 °C [44], red crystals 134–135.5 °C and yellow crystals 117–119 °C [45]).
2,6-bis((benzylimino)methyl)-4-methylphenol: 99% yield; orange solid; m. p. 69.6–69.7 °C (lit. 68–72°C [46]).
2,6-bis((phenylethylimino)methyl)-4-methylphenol: 68% yield; orange oil; ^1^H NMR 2.28 (s, 3H, C*H_3_*), 3.01 (t, 4H, J 7.4, C*H_2_*-Ph), 3.85 (t, 4H, J 7.4, C*H_2_*-N), 7.19–7.24 (m, 6H, C*H*-2+6 and C*H*-4 Ph), 7.29 (t, 4H, J 7.5, C*H*-3+5 Ph), 7.45 (bs, 2H, C*H*-3 and C*H*-5), 8.438 (bs, 2H, C*H*=N), 13.92 (bs, 1H, O*H*).

*Step 2:* To a solution of 2,6-bis-iminomethyl-4-methylphenol (4 mmol) in CH_3_OH (10–15 mL), NaBH_4_ (341 mg; 9 mmol) was added portion-wise and the mixture was stirred at room temperature for 1 h. The solvent was removed in vacuo and the products were partitioned between CH_2_Cl_2_ and water. The organic phase was washed with water, dried over Na_2_SO_4_ and evaporated to dryness to obtain pure oily products, which were subjected to a further reaction without purification, except **1c**, which was purified via flash chromatography on silica gel.

**1a**: 99% yield; reddish oil; ^1^H NMR 2.32 (s, 3H, C*H_3_*), 4.39 (s, 4H, C*H_2_*), 6.82 (dd, 4H, J 7.6, 1.1, C*H*-2+*H*-6 Ph), 6.88 (tt, 2H, J 7.3, 1.1, C*H*-4 Ph), 7.03 (s, 2H, C*H*-3 and C*H*-5), 7.27 (td, 4H, J 7.4, 1.1, C*H*-3+5 Ph); ^13^C NMR 20.6 (*C*H_3_), 46.4 (*C*H_2_), 114.7 (*C*H-2+6 Ph), 119.3 (*C*H-4 Ph), 124.4 (*C*_q_-2 and *C*_q_-6), 128.8 (*C*H-3 and *C*H-5), 128.9 (*C*_q_-4), 129.3 (*C*H-3+5 Ph), 147.9 (*C*_q_-1 Ph), 152.8 (*C*_q_-1).
**1b**: 98% yield; yellowish oil; NMR identical with the literature data [47].
**1c**: 88% yield; colourless oil; ^1^H NMR 2.20 (s, 3H, C*H_3_*), 2.86 (t, 4H, J 6.9, C*H_2_*-Ph), 2.92 (td, 4H, J 6.9, 1.3, C*H_2_*-N), 3.87 (s, 4H, C*H_2_*-bridge), 4.89 (bs, 2H, N*H*), 6.80 (s, 2H, C*H*-3 and C*H*-5), 7.19-7.22 (m, 6H, C*H*-2+6 and C*H*-4 Ph), 7.29 (td, 4H, J 7.4, 1.7, C*H*-3+5 Ph); ^13^C NMR 20.40 (*C*H_3_), 35.8 (*C*H_2_-Ph), 49.9 (*C*H_2_-N), 50.6 (*C*H_2_-bridge), 123.5 (*C*_q_-2 and *C*_q_-6), 126.3 (*C*H-4 Ph), 127.7 (*C*_q_-4), 128.6 (*C*H-2+6 Ph), 128.7 (*C*H-3+5 Ph), 128.8 (*C*H-3 and *C*H-5), 139.4 (*C*_q_-1 Ph), 154.1 (*C*_q_-1).

### 3.3. General Procedure for the Preparation of Ligands **2** and **3**

*Step 1:* To a commercial 15% solution of phosgene in toluene (2 mmol), an amine (1 mmol) was added in an argon atmosphere and the mixture was stirred at room temperature for 40 min. The mixture was bubbled with argon to eliminate the excess phosgene and the crude carbamic chloride (CC) was further used without purification.

*Step 2:* To a solution of bis-amine (**1**) and pyridine in toluene, carbamic chloride was added portion-wise in an argon atmosphere and the mixture was stirred at room temperature for 24 h. The products were partitioned between toluene and brine. The organic layer was washed with 10 % aq. HCl and then with brine, dried over MgSO_4_, and evaporated to dryness. The product was purified via flash chromatography on silica gel by using a mobile phase with a gradient of polarity from DCM to 2% MeOH/DCM. The reagents’ proportions and the corresponding yields are summarized in Table 1. 
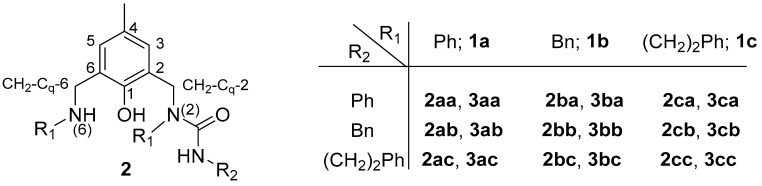


Structure and numeration scheme of ligands **2**; ligands **3** are symmetrical.

*Ligand **2aa**:* R_f_ 0.65 (0.5% MeOH/DCM); colourless solid; m. p. 111.6–112.8 °C; ^1^H NMR 2.09 (s, 3H, C*H*_3_), 4.36 (s, 2H, C*H*_2_-C_q_-6), 4.77 (s, 2H, C*H*_2_-C_q_-2), 6.13 (s, 1H, N*H*-CO), 6.37 (d, 1H, J 2.0, C*H*-3), 6.70 (tt, 1H, J 7.3, 0.9, C*H*-4 Ph-N(6) of R_1_), 6.72 (dd, 2H, J 8.6, 0.9, C*H*-2+6 Ph-N(6) of R_1_), 7.03 (m, 1H, C*H*-4 Ph of R_2_), 7.04 (d, 1H, J 2.1, C*H*-5), 7.16 (ddt, 2H, J 8.5, 7.3, 1.9, C*H*-3+5 Ph-N(6) of R_1_), 7.19 (dd, 2H, J 8.5, 1.5, C*H*-2+6 Ph-N(2) of R_1_), 7.25 (m, 4H, C*H*-2+6 and C*H*-3+5 Ph of R_2_), 7.46 (tt, 1H, J 7.1, 1.3, C*H*-4 Ph-N(2) of R_1_), 7.48 (ddt, 2H, J 8.6, 7.0, 1.3, C*H*-3+5 Ph-N(2) of R_1_), 9.94 (bs, 1H, O*H*); ^13^C NMR 20.36 (*C*H_3_), 44.5 (*C*H_2_-C_q_-6), 50.5 (*C*H_2_-C_q_-2), 113.6 (*C*H-2+6 Ph-N(6) of R_1_), 117.6 (*C*H-4 Ph-N(6) of R_1_), 119.8 (*C*H-2+6 Ph of R_2_), 122.6 (*C*_q_-2), 123.7 (*C*H-4 Ph of R_2_), 126.8 (*C*_q_-6), 127.9 (*C*_q_-4), 128.9 (*C*H-3+5 Ph of R_2_), 129.0 (*C*H-4 Ph-N(2) of R_1_), 129.1 (*C*H-2+6 Ph-N(2) of R_1_), 129.2 (*C*H-3+5 Ph-N(6) of R_1_), 130.1 (*C*H-5), 130.5 (*C*H-3+5 Ph-N(2) of R_1_), 130.9 (*C*H-3), 137.8 (*C*_q_-1 Ph of R_2_), 140.2 (*C*_q_-1 Ph-N(2) of R_1_), 148.3 (*C*_q_-1 Ph-N(6) of R_1_), 152.0 (*C*_q_-1), 156.1 (*C*=O); ESI MS *m*/*z* 226 [M-NPhCONHPh]^+^ (100), 371 [M − Cl]^+^ (36), 407 [M+1]^+^ (58), 429 [M + Na]^+^ (18).
*Ligand **3aa**:* R_f_ 0.35 (0.5% MeOH/DCM); pale brown solid; m. p. 91.1–91.3 °C; ^1^H NMR 2.07 (s, 3H, C*H*_3_), 4.88 (s, 4H, C*H*_2_-C_q_-2 and C*H*_2_-C_q_-6), 6.59 (s, 2H, N*H*-CO), 6.677 (s, 2H, C*H*-3 and C*H*-5), 7.00 (tt, 2H, J 7.4, 1.0, C*H*-4 Ph of R_2_), 7.22 (m, 8H, C*H*-2+6 Ph of R_1_ and C*H*-3+5 Ph of R_2_), 7.31 (dd, 4H, J 8.6, 1.0, C*H*-2+6 Ph of R_2_), 7.33 (tt, 2H, J 7.4, 1.1, C*H*-4 Ph of R_1_), 7.42 (t, 4H, J 7.6, C*H*-3+5 Ph of R_1_), 10.06 (bs, 1H, O*H*); ^13^C NMR ; 20.4 (*C*H_3_), 49.4 (*C*H_2_-C_q_-2 and *C*H_2_-C_q_-6), 119.6 (*C*H-2+6 Ph of R_2_), 123.2 (*C*H-4 Ph of R_2_), 124.0 (*C*_q_-2 and *C*_q_-6), 128.16 (*C*_q_-4), 128.24 (*C*H-4 Ph of R_1_), 128.6 (*C*H-2+6 Ph of R_1_), 128.8 (*C*H-3+5 Ph of R_2_), 130.1 (*C*H-3+5 Ph of R_1_), 131.0 (*C*H-3 and *C*H-5), 138.5 (*C*_q_-1 Ph of R_2_), 141.1 (*C*_q_-1 Ph of R_1_), 151.2 (*C*_q_-1), 155.5 (*C*=O); ESI MS *m*/*z* 557 [M + 1]^+^ (16), 579 [M + Na]^+^ (54), 595 [M + K]^+^ (7), 1135 [2M + Na]^+^ (100).
*Ligand **2ab**:* R_f_ 0.70 (1% MeOH/DCM); colourless solid; m. p. 137.2–137.3 °C; ^1^H NMR 2.09 (s, 3H, C*H*_3_), 4.37 (s, 2H, C*H*_2_-C_q_-6), 4.40 (d, 2H, J 5.8, C*H*_2_ of R_2_), 4.58 (t, 1H, J 5.8, N*H*-CO), 4.72 (s, 2H, C*H*_2_-C_q_-2), 6.34 (d, 1H, J 2.0, C*H*-3), 6.69 (tt, 1H, J 7.3, 1.0, C*H*-4 Ph-N(6) of R_1_), 6.72 (dd, 2H, J 8.4, 0.7, C*H*-2+6 Ph-N(6) of R_1_), 7.04 (d, 1H, J 1.8, C*H*-5), 7.11 (dd, 2H, J 8.6, 1.4, C*H*-2+6 Ph-N(2) of R_1_), 7.17 (m, 4H, C*H*-3+5 Ph-N(6) of R_1_ and C*H*-2+6 Ph of R_2_), 7.23 (tt, 1H, J 7.4, 1.9, C*H*-4 Ph of R_2_), 7.29 (td, 2H, J 7.6, 1.6, C*H*-3+5 Ph of R_2_), 7.36 (tt, 1H, J 7.3, 1.2, C*H*-4 Ph-N(2) of R_1_), 7.41 (ddt, 2H, J 8.5, 7.1, 1.1, C*H*-3+5 Ph-N(2) of R_1_), 10.24 (bs, 1H, O*H*); ^13^C NMR 20.38 (*C*H_3_), 44.4 (*C*H_2_-C_q_-6), 44.9 (*C*H_2_ of R_2_), 50.8 (*C*H_2_-C_q_-2), 113.5 (*C*H-2+6 Ph-N(6) of R_1_), 117.4 (*C*H-4 Ph-N(6) of R_1_), 123.0 (*C*_q_-2), 126.9 (*C*_q_-6), 127.3 (*C*H-2+6 Ph of R_2_), 127.4 (*C*H-4 Ph of R_2_), 127.7 (*C*_q_-4), 128.63 (*C*H-4 Ph-N(2) of R_1_), 128.65 (*C*H-3+5 Ph of R_2_), 128.9 (*C*H-2+6 Ph-N(2) of R_1_), 129.1 (*C*H-3+5 Ph-N(6) of R_1_), 130.1 (*C*H-5), 130.4 (*C*H-3+5 Ph-N(2) of R_1_), 130.9 (*C*H-3), 138.8 (*C*_q_-1 Ph of R_2_), 140.6 (*C*_q_-1 Ph-N(2) of R_1_), 148.6 (*C*_q_-1 Ph-N(6) of R_1_), 152.2 (*C*_q_-1), 158.7 (*C*=O); ESI MS *m*/*z* 359 [M-PhCH_2_ + 1]^+^ (100), 452 [M + 1]^+^ (47), 474 [M + Na]^+^ (16), 490 [M + K]^+^ (2), 926 [2M + Na]^+^ (20).
*Ligand **3ab**:* R_f_ 0.27 (1% MeOH/DCM); colourless solid; m. p. 142.7–142.9 °C; ^1^H NMR 2.09 (s, 3H, C*H*_3_), 4.40 (d, 4H, J 5.8, C*H*_2_ of R_2_), 4.49 (t, 2H, J 5.8, N*H*-CO), 4.83 (s, 4H, C*H*_2_-C_q_-2 and C*H*_2_-C_q_-6), 6.68 (s, 2H, C*H*-3 and C*H*-5), 7.16 (dd, 4H, J 8.1, 1.4, C*H*-2+6 Ph of R_1_), 7.17 (m, 6H, C*H*-2+6 and C*H*-4 Ph of R_2_), 7.28 (m, 6H, C*H*-4 Ph of R_1_ and C*H*-3+5 Ph of R_2_), 7.35 (ddt, 4H, J 7.9, 7.4, 1.7, C*H*-3+5 Ph of R_1_), 10.02 (bs, 1H, O*H*); ^13^C NMR 20.5 (*C*H_3_), 44.4 (*C*H_2_-C_q_-6), 44.8 (*C*H_2_ of R_2_), 49.6 (*C*H_2_-C_q_-2 and *C*H_2_-C_q_-6), 124.4 (*C*_q_-2 and *C*_q_-6), 127.2 (*C*H-4 Ph of R_2_), 127.3 (*C*H-2+6 Ph of R_2_), 127.67 (*C*_q_-6), 127.69 (*C*_q_-4), 128.5 (*C*H-2+6 Ph of R_1_), 128.6 (*C*H-3+5 Ph of R_2_), 129.9 (*C*H-3+5 Ph of R_1_), 130.2 (*C*H-3 and *C*H-5), 139.3 (*C*_q_-1 Ph of R_2_), 141.6 (*C*_q_-1 Ph of R_1_), 151.5 (*C*_q_-1), 158.0 (*C*=O); ESI MS *m*/*z* 585 [M + 1]^+^ (43), 607 [M + Na]^+^ (89), 623 [M + K]^+^ (15), 1191 [2M + Na]^+^ (100).
*Ligand **2ac***: R_f_ 0.55 (1% MeOH/DCM); colourless solid; m. p. 134.5–134.6 °C; ^1^H NMR 2.08 (s, 3H, C*H*_3_), 2.73 (t, 2H, J 6.8, C*H*_2_-Ph of R_2_), 3.42 (td, 2H, J 6.8, 5.9, C*H*_2_-N of R_2_), 4.22 (t, 1H, J 5.7, N*H*-CO), 4.37 (s, 2H, C*H*_2_-C_q_-6), 4.67 (s, 2H, C*H*_2_-C_q_-2), 6.33 (d, 1H, J 2.0, C*H*-3), 6.69 (tt, 1H, J 7.3, 1.0, C*H*-4 Ph-N(6) of R_1_), 6.72 (dd, 2H, J 8.6, 1.0, C*H*-2+6 Ph-N(6) of R_1_), 6.96 (m, 2H, C*H*-2+6 Ph-N(2) of R_1_), 7.02 (dd, 2H, J 8.4, 1.2, C*H*-2+6 Ph of R_2_), 7.04 (d, 1H, J 1.8, C*H*-5), 7.15-7.20 (m, 5H, C*H*-3+5 Ph-N(6) of R_1_ and C*H*-3+5 and C*H*-4 Ph of R_2_), 7.34 (m, 3H, C*H*-3+5 and C*H*-4 Ph-N(2) of R_1_), 10.28 (bs, 1H, O*H*); ^13^C NMR 20.3 (*C*H_3_), 36.0 (*C*H_2_-Ph of R_2_), 42.1 (*C*H_2_-N of R_2_), 44.4 (*C*H_2_-C_q_-6), 50.5 (*C*H_2_-C_q_-2), 113.4 (*C*H-2+6 Ph-N(6) of R_1_), 117.3 (*C*H-4 Ph-N(6) of R_1_), 123.0 (*C*_q_-2), 126.4 (*C*H-4 Ph of R_2_), 126.9 (*C*_q_-6), 127.6 (*C*_q_-4), 128.4 (*C*H-4 Ph-N(2) of R_1_), 128.5 (*C*H-2+6 Ph of R_2_), 128.7 (*C*H-2+6 Ph-N(2) of R_1_), 128.8 (*C*H-3+5 Ph of R_2_), 129.1 (*C*H-3+5 Ph-N(6) of R_1_), 130.0 (*C*H-5), 130.1 (*C*H-3+5 Ph-N(2) of R_1_), 130.8 (*C*H-3), 138.7 (*C*_q_-1 Ph of R_2_), 140.5 (*C*_q_-1 Ph-N(2) of R_1_), 148.6 (*C*_q_-1 Ph-N(6) of R_1_), 152.1 (*C*_q_-1), 158.6 (*C*=O); ESI MS *m*/*z* 373 [M-PhCH_2_ + 1]^+^ (100), 466 [M + 1]^+^ (55), 489 [M + Na]^+^ (5), 504 [M + K]^+^ (1), 954 [2M + Na]^+^ (15).
*Ligand **3ac**:* R_f_ 0.21 (1% MeOH/DCM); colourless solid; m. p. 132.8–132.9 °C; ^1^H NMR 2.09 (s, 3H, C*H*_3_), 2.74 (t, 4H, J 6.9, C*H*_2_-Ph of R_2_), 3.42 (td, 4H, J 6.9, 6.0, C*H*_2_-N of R_2_), 4.42 (t, 2H, J 5.7, N*H*-CO), 4.37 (s, 4H, C*H*_2_-C_q_-2 and C*H*_2_-C_q_-6), 6.65 (d, 2H, J 2.0, C*H*-3 and C*H*-5), 7.02 (dd, 4H, J 8.8, 1.4, C*H*-2+6 Ph of R_1_), 7.06 (dd, 4H, J 8.5, 1.4, C*H*-2+6 Ph of R_2_), 7.16 (tt, 2H, J 7.3, 1.2, C*H*-4 Ph of R_2_), 7.20 (ddt 4H, J 8.3, 7.0, 1.2, C*H*-3+5 Ph of R_2_), 7.25 (tt, 2H, J 7.3, 1.3, C*H*-4 Ph of R_1_), 7.29 (ddt, 4H, J 8.4, 7.1, 1.3, C*H*-3+5 Ph of R_1_), 10.02 (bs, 1H, O*H*); ^13^C NMR 20.5 (*C*H_3_), 36.1 (*C*H_2_-Ph of R_2_), 42.1 (*C*H_2_-N of R_2_), 49.3 (*C*H_2_-C_q_-2 and *C*H_2_-C_q_-6), 124.4 (*C*_q_-2 and *C*_q_-6), 126.2 (*C*H-4 Ph of R_2_), 127.5 (*C*_q_-4), 127.6 (*C*H-4 Ph of R_1_), 128.46 (*C*H-3+5 Ph of R_2_), 128.48 (*C*H-2+6 Ph of R_1_), 128.7 (*C*H-2+6 Ph of R_2_), 129.7 (*C*H-3+5 Ph of R_1_), 130.1 (*C*H-3 and *C*H-5), 139.1 (*C*_q_-1 Ph of R_2_), 141.5 (*C*_q_-1 Ph of R_1_), 151.4 (*C*_q_-1), 157.9 (*C*=O); ESI MS *m*/*z* 613 [M + 1]^+^ (34), 635 [M + Na]^+^ (100), 652 [M + K]^+^ (10).
*Ligand **3ba**:* R_f_ 0.47 (1% MeOH/DCM); colourless solid; m. p. 181.5–181.6 °C; ^1^H NMR 2.24 (s, 3H, C*H*_3_), 4.50 (bs, 4H, C*H*_2_-C_q_-2 and C*H*_2_-C_q_-6), 4.62 (s, 4H, C*H*_2_ of R_1_), 6.90 (s, 2H, C*H*-3 and C*H*-5), 6.99 (bt, 2H, J 7.3, C*H*-4 Ph of R_2_), 7.19 (bt 4H, J 7.9, C*H*-3+5 Ph of R_2_), 7.30-7.37 (bm, 10H, CH Ph), 7.41 (bt 4H, J 7.7, C*H*-3+5 Ph of R_1_), 11.23 (bs, 1H, O*H*); ^13^C NMR 20.4 (*C*H_3_), 49.7 (*C*H_2_-C_q_-2 and *C*H_2_-C_q_-6), 49.8 (*C*H_2_- of R_1_), 119.9 (*C*H-2+6 Ph of R_2_), 123.1 (*C*_q_-2 and *C*_q_-6), 123.7 (*C*H-4 Ph of R_2_), 127.2 (*C*H-4 Ph of R_1_), 128.7 (*C*_q_-4), 128.8 (*C*H-3+5 Ph of R_2_), 128.9 (*C*H-2+6 Ph of R_1_), 129.2 (*C*H-3+5 Ph of R_1_), 132.2 (*C*H-3 and *C*H-5), 138.6 (*C*_q_-1 Ph of R_1_), 138.9 (*C*_q_-1 Ph of R_2_), 151.6 (*C*_q_-1), 157.2 (*C*=O); ESI MS *m*/*z* 226 [PhNHCONHCH_2_Ph]^+^ (28), 360 [M-PhCH_2_NHCONHPh+1]^+^ (95), 585 [M + 1]^+^ (100), 1170 [2M + 1]^+^ (59).
*Ligand **2bb**:* R_f_ 0.26 (3% MeOH/DCM); yellow solid; m. p. 121.0–121.2 °C; ^1^H NMR 2.21 (s, 3H, C*H*_3_), 3.72 (s, 2H, C*H*_2_-N(6) of R_1_), 3.87 (s, 2H, C*H*_2_-C_q_-6), 4.34 (s, 2H, C*H*_2_-C_q_-2), 4.45 (d, 2H, J 5.6, C*H*_2_-NH of R_2_), 4.66 (s, 2H, C*H*_2_-N(2) of R_1_), 6.01 (bs, 1H, N*H*-CO), 6.74 (d, 1H, J 1.3, C*H*-5), 6.82 (d, 1H, J 1.3, C*H*-3), 7.17 (dd, 2H, J 7.7, 1.1, C*H*-2+6 Ph of R_2_), 7.19-7.24 (m, 5H, C*H* Ph), 7.28 (m, 2H, 2 C*H*-4 Ph), 7.33 (m, 6H, C*H* Ph), 11.34 (bs, 1H, O*H*); ^13^C NMR 20.5 (*C*H_3_), 44.8 (*C*H_2_-C_q_-2), 44.9 (*C*H_2_ of R_2_), 50.2 (*C*H_2_-N(2) of R_1_), 51.5 (*C*H_2_-C_q_-6), 52.6 (*C*H_2_-N(6) of R_1_), 122.4 (*C*_q_-6), 123.7 (*C*_q_-2), 126.8 (*C*H Ph), 127.2 (*C*H-2+6 Ph of R_2_), 127.4 (2 *C*H Ph), 127.4 (*C*_q_-4), 127.6 (*C*H Ph), 127.8 (*C*H Ph), 128.29 (2 *C*H Ph), 128.33 (2 *C*H Ph), 128.6 (2 *C*H Ph), 128.69 (2 *C*H Ph), 128.72 (*C*H-5), 129.5 (*C*H-3), 138.2 (*C*_q_-1 Ph of N(6)-R_1_), 138.6 (*C*_q_-1 Ph of N(2)-R_1_), 139.9 (*C*_q_-1 Ph of R_2_), 153.2 (*C*_q_-1), 158.8 (*C*=O); ESI MS *m*/*z* 480 [M + 1]^+^ (100), 502 [M + Na]^+^ (7), 982 [2M + Na]^+^ (20).
*Ligand **3bb**:* R_f_ 0.36 (1% MeOH/DCM); colourless solid; m. p. 152.6–152.7 °C; ^1^H NMR 2.20 (s, 3H, C*H*_3_), 4.39 (d, 4H, J 5.5, C*H*_2_-NH of R_2_), 4.42 (s, 4H, C*H*_2_-C_q_-2 and C*H*_2_-C_q_-6), 4.52 (d, 4H, J 5.5, C*H*_2_ of R_1_), 5.35 (bs, 1H, N*H*-CO), 6.83 (s, 2H, C*H*-3 and C*H*-5), 7.14 (d 4H, J 7.4, C*H*-2+6 Ph of R_1_), 7.20 (t, 2H, J 7.4, C*H*-4 Ph of R_1_), 7.26 (m, 8H, C*H*-3+5 Ph of R_1_ and C*H*-2+6 Ph of R_2_). 7.29 (t, 2H, J 7.3, C*H*-4 Ph of R_2_), 7.35 (dd 4H, J 7.6, 7.3, C*H*-3+5 Ph of R_2_), 10.71 (bs, 1H, O*H*); ^13^C NMR 20.4 (*C*H_3_), 45.0 (*C*H_2_ of R_2_), 46.9 (*C*H_2_-C_q_-2 and *C*H_2_-C_q_-6), 50.2 (*C*H_2_ of R_1_), 123.9 (*C*_q_-2 and *C*_q_-6), 127.0 (*C*H-2+6 Ph of R_2_), 127.1 (*C*H-4 Ph of R_1_), 127.3 (*C*H-2+6 Ph of R_1_), 127.6 (*C*H-4 Ph of R_2_), 128.2 (*C*_q_-4), 128.5 (*C*H-3+5 Ph of R_1_), 128.9 (*C*H-3+5 Ph of R_2_), 130.9 (*C*H-3 and *C*H-5), 137.1 (*C*_q_-1 Ph of R_1_), 139.2 (*C*_q_-1 Ph of R_2_), 151.9 (*C*_q_-1), 159.3 (*C*=O); ESI MS *m*/*z* 613 [M + 1]^+^ (26), 635 [M + Na]^+^ (100), 651 [M + K]^+^ (15).
*Ligand **2bc**:* R_f_ 0.22 (3% MeOH/DCM); yellow solid; m. p. 140.9–141.1 °C; ^1^H NMR 2.21 (s, 3H, C*H*_3_), 2.78 (t, 2H, J 7.0, C*H*_2_-Ph of R_2_), 3.49 (td, 2H, J 7.0, 5.7, C*H*_2_-N of R_2_), 3.78 (s, 2H, C*H*_2_-N(6) of R_1_), 3.92 (s, 2H, C*H*_2_-C_q_-6), 4.30 (s, 2H, C*H*_2_-C_q_-2), 4.55 (s, 2H, C*H*_2_-N(2) of R_1_), 5.45 (bs, 1H, N*H*-CO), 6.77 (bs, 1H, C*H*-5), 6.80 (bs, 1H, C*H*-3), 7.09 (dd, 2H, J 7.5, 1.1, C*H*-2+6 Ph of R_2_), 7.15 (tt, 1H, J 7.3, 1.0, C*H*-4 Ph of R_2_), 7.20 (ddt, 2H, J 7.5, 7.2, 1.0, C*H*-3+5 Ph of R_2_), 7.27 (m, 4H, C*H* Ph), 7.29 (dd, 2H, J 7.6, 1.2, C*H*-2+6 Ph), 7.33 (m, 4H, C*H* Ph), 11.13 (bs, 1H, O*H*); ^13^C NMR 20.6 (*C*H_3_), 36.4 (*C*H_2_-Ph of R_2_), 42.3 (*C*H_2_-N of R_2_), 45.2 (*C*H_2_-C_q_-2), 50.0 (*C*H_2_-N(2) of R_1_), 51.4 (*C*H_2_-C_q_-6), 52.6 (*C*H_2_-N(6) of R_1_), 122.5 (*C*_q_-6), 123.6 (*C*_q_-2), 126.1 (*C*H-4 Ph of R_2_), 127.2 (*C*H Ph), 127.55 (*C*H-2+6 Ph), 127.65 (*C*H Ph), 128.0 (*C*_q_-4), 128.2 (*C*H Ph), 128.36 (2 *C*H Ph), 128.39 (*C*H-3+5 Ph of R_2_), 128.6 (2 *C*H Ph), 128.7 (2 *C*H Ph), 128.8 (*C*H-2+6 Ph of R_2_), 128.9 (*C*H-5), 129.4 (*C*H-3), 138.2 (*C*_q_-1 Ph of N(2) R_2_), 138.3 (*C*_q_-1 Ph of N(6) R_2_), 139.6 (*C*_q_-1 Ph of R_2_), 153.2 (*C*_q_-1), 159.0 (*C*=O); ESI MS *m*/*z* 387 [M-PhCH_2_ + 1]^+^ (8), 494 [M + 1]^+^ (100), 516 [M + Na]^+^ (4), 987 [2M + 1]^+^ (14), 1010 [2M + Na + 1]^+^ (8).
*Ligand **3bc**:* R_f_ 0.34 (1% MeOH/DCM); colourless solid; m. p. 131.6–131.7 °C; ^1^H NMR 2.24 (s, 3H, C*H*_3_), 2.88 (t, 4H, J 7.1, C*H*_2_-Ph of R_2_), 3.49 (bt, 4H, J 7.1, C*H*_2_-N of R_2_), 4.24 (d, 4H, J 4.9, C*H*_2_ of R_1_), 4.34 (s, 4H, C*H*_2_-C_q_-2 and C*H*_2_-C_q_-6), 4.89 (bs, 2H, N*H*-CO), 6.88 (s, 2H, C*H*-3 and C*H*-5), 7.12 (d, 4H, J 7.3, C*H*-2+6 Ph of R_1_), 7.17 (m, 6H, C*H* Ph), 7.22-7.28 (m, 10H, C*H* Ph), 10.79 (bs, 1H, O*H*); ^13^C NMR 20.5 (*C*H_3_), 36.4 (*C*H_2_-Ph of R_2_), 45.0 (*C*H_2_ of R_1_), 46.6 (*C*H_2_-C_q_-2 and *C*H_2_-C_q_-6), 49.2 (*C*H_2_-N of R_2_), 123.8 (*C*_q_-2 and *C*_q_-6), 126.7 (*C*H Ph), 127.1 (*C*H Ph), 127.5 (*C*H-2+6 Ph of R_1_), 128.1 (*C*_q_-4), 128.5 (2 *C*H Ph), 128.8 (4 *C*H Ph), 130.6 (*C*H-3 and *C*H-5), 139.09 (*C*_q_-1 Ph), 139.11 (*C*_q_-1 Ph), 151.8 (*C*_q_-1), 159.1 (*C*=O); ESI MS *m*/*z* 641 [M + 1]^+^ (39), 663 [M + Na]^+^ (100), 679 [M + K]^+^ (15).
*Ligand **2ca**:* R_f_ 0.24 (1% MeOH/DCM); colourless oil; ^1^H NMR 2.23 (s, 3H, C*H*_3_), 2.82 (t, 2H, J 6.9, C*H*_2_-Ph of N(6) R_1_), 2.93 (m, 4H, C*H*_2_-Ph of N(2) R_1_ and C*H*_2_-N of N(6) R_1_), 3.63 (t, 2H, J 7.5, C*H*_2_-N of N(2) R_1_), 3.95 (s, 2H, C*H*_2_-C_q_-6), 4.35 (s, 2H, C*H*_2_-C_q_-2), 6.77 (d, 1H, J 1.6, C*H*-5), 6.94 (d, 1H, J 1.7, C*H*-3), 6.97 (tt, 1H, J 7.3, 1.1, C*H*-4 Ph of R_1_), 7.14 (dd, 2H, J 8.2, 1.2, C*H*-2+6 Ph of R_2_), 7.21 (m, 2H, C*H*-4 Ph of R_1_ and C*H*-4 Ph of R_2_), 7.24–7.32 (m, 8H, C*H*-3+5 Ph and C*H*-2+6 Ph of R_1_), 7.36 (bd, 2H, J 7.6, C*H*-2+6 Ph of R_1_); ^13^C NMR 20.5 (*C*H_3_), 34.4 (*C*H_2_-Ph of N(2) R_1_), 35.4 (*C*H_2_-Ph of N(6) R_1_), 46.4 (*C*H_2_-C_q_-2), 49.3 (*C*H_2_-N of N(6) R_1_), 49.6 (*C*H_2_-N of N(2) R_1_), 52.0 (*C*H_2_-C_q_-6), 119.3 (*C*H-2+6 Ph of R_1_), 122.1 (*C*H-4 Ph of R_1_), 122.3 (*C*_q_-6), 123.8 (*C*_q_-2), 126.6 (*C*H-4 Ph of R_2_), 128.5 (*C*_q_-4), 128.5 (*C*H-4 Ph of R_1_), 128.58 (2 *C*H Ph), 128.62 (2 *C*H Ph), 128.68 (2 *C*H Ph), 128.73 (2 *C*H Ph), 129.0 (2 *C*H Ph), 129.1 (*C*H-5), 130.1 (*C*H-3), 138.6 (*C*_q_-1 Ph of N(6) R_1_), 139. 7 (*C*_q_-1 Ph of N(2) R_1_), 140.1 (*C*_q_-1 Ph of R_2_), 153.2 (*C*_q_-1), 156.0 (*C*=O); ESI MS *m*/*z* 494 [M + 1]^+^ (100), 496 [M + Na]^+^ (8), 532 [M + K]^+^ (1), 987 [2M + 1]^+^ (17), 1010 [2M + Na + 1]^+^ (17).
*Ligand **3ca**:* R_f_ 0.35 (1% MeOH/DCM); yellow solid; m. p. 118.5–118.8°C; ^1^H NMR 2.30 (s, 3H, C*H*_3_), 2.97 (t, 4H, J 6.9, C*H*_2_-Ph of R_1_), 3.60 (t, 4H, J 6.9, C*H*_2_-N of R_1_), 4.45 (bs, 4H, C*H*_2_-C_q_-2 and C*H*_2_-C_q_-6), 6.95 (m, 3H, C*H* Ph), 6.99 (s, 2H, C*H*-3 and C*H*-5), 7.15 (m, 6H, C*H* Ph), 7.28 (m, 7H, C*H* Ph), 7.36 (m, 4H, C*H* Ph), 11.35 (bs, 1H, O*H*); ^13^C NMR 20.5 (*C*H_3_), 34.1 (*C*H_2_-Ph of R_1_), 46.7 (*C*H_2_-C_q_-2 and *C*H_2_-C_q_-6), 49.2 (*C*H_2_-N of R_1_), 119.8 (*C*_q_-2 and *C*_q_-6), 123.1 (2 *C*H-4 Ph), 128.6 (*C*_q_-4), 128.8 (8 *C*H Ph), 128.96 (2 *C*H-4 Ph), 129.03 (8 *C*H Ph), 131.9 (*C*H-3 and *C*H-5), 138.8 (*C*_q_-1 Ph of R_2_), 139.3 (*C*_q_-1 Ph of R_1_), 153.6 (*C*_q_-1), 158.2 (*C*=O); ESI MS *m*/*z* 494 [M-PhNHCO + 1]^+^ (23), 613[M + 1]^+^ (32), 635 [M + Na]^+^ (100), 651 [M + K]^+^ (13).
*Ligand **2cb**:* R_f_ 0.14 (2% MeOH/DCM); colourless solid; m. p. 90.3–90.5 °C; ^1^H NMR 2.20 (s, 3H, C*H*_3_), 2.77 (t, 2H, J 6.9, C*H*_2_-Ph of N(6) R_1_), 2.86 (t, 2H, J 6.9, C*H*_2_-N of N(6) R_1_), 2.91 (dd, 2H, J 7.8, 7.4, C*H*_2_-Ph of N(2) R_1_), 3.61 (dd, 2H, J 7.7, 7.5, C*H*_2_-N of N(2) R_1_), 3.86 (s, 2H, C*H*_2_-C_q_-6), 4.30 (s, 2H, C*H*_2_-C_q_-2), 4.38 (d, 2H, J 5.5, C*H*_2_ of R_2_), 5.66 (bs, 1H, N*H*-CO), 6.73 (d, 1H, J 1.7, C*H*-5), 6.89 (d, 1H, J 1.7, C*H*-3), 7.14 (dd, 2H, J 8.1, 1.2, C*H*-2+6 Ph), 7.17-7.30 (m, 13H, C*H* Ph); ^13^C NMR 20.5 (*C*H_3_), 34.8 (*C*H_2_-Ph of N(2) R_1_), 35.5 (*C*H_2_-Ph of N(6) R_1_), 44.8 (*C*H_2_ of R_2_), 45.880 (*C*H_2_-C_q_-2), 49.5 (*C*H_2_-N of N(6) R_1_), 49.9 (*C*H_2_-N of N(2) R_1_), 52.0 (*C*H_2_-C_q_-6), 122.2 (*C*_q_-6), 124.0 (*C*_q_-2), 126.3 (*C*H Ph), 126.6 (*C*H Ph), 126.9 (*C*H Ph), 127.4 (2 *C*H Ph), 128.3 (*C*_q_-4), 128.4 (2 *C*H Ph), 128.6 (2 *C*H Ph), 128.69 (2 *C*H Ph), 128.71 (*C*H-5), 128.73 (2 *C*H Ph), 129.0 (2 *C*H Ph), 129.1 (*C*H-3), 138.9 (*C*_q_-1 Ph of N(6) R_1_), 139.6 (*C*_q_-1 Ph of N(2) R_1_), 139.9 (*C*_q_-1 Ph of R_2_), 153.2 (*C*_q_-1), 158.5 (*C*=O); ESI MS *m*/*z* 508 [M + 1]^+^ (100), 530 [M + Na]^+^ (9), 1037 [2M + Na]^+^ (100).
*Ligand **3cb**:* R_f_ 0.41 (2% MeOH/DCM); colourless solid; m. p. 151.6–152.0 °C; ^1^H NMR 2.24 (s, 3H, C*H*_3_), 2.88 (t, 4H, J 7.1, C*H*_2_-Ph of R_1_), 3.49 (bt, 4H, J 7.0, C*H*_2_-N of R_1_), 4.24 (d, 4H, J 4.4, C*H*_2_ of R_2_), 4.34 (s, 4H, C*H*_2_-C_q_-2 and C*H*_2_-C_q_-6), 4.89 (bs, 2H, N*H*-CO), 6.88 (s, 2H, C*H*-3 and C*H*-5), 7.12 (d, 4H, J 7.3, C*H*-2+6 Ph), 7.17 (m, 6H, C*H* Ph), 7.25 (m, 10H, C*H* Ph), 10.79 (bs, 1H, O*H*); ^13^C NMR 20.5 (*C*H_3_), 34.4 (*C*H_2_-Ph of R_1_), 45.0 (*C*H_2_ of R_2_), 46.6 (*C*H_2_-C_q_-2 and *C*H_2_-C_q_-6), 49.2 (*C*H_2_-N of R_1_), 123.8 (*C*_q_-2 and *C*_q_-6), 126.7 (2 *C*H-4 Ph), 127.1 (2 *C*H-4 Ph), 127.5 (4 *C*H Ph), 128.1 (*C*_q_-4), 128.5 (4 *C*H Ph), 128.8 (8 *C*H Ph), 130.6 (*C*H-3 and *C*H-5), 138.7 (*C*_q_-1 Ph of R_1_), 139.1 (*C*_q_-1 Ph of R_2_), 151.8 (*C*_q_-1), 159.1 (*C*=O); ESI MS *m*/*z* 641 [M + 1]^+^ (18), 663 [M + Na]^+^ (100), 679 [M + K]^+^ (14).
*Ligand **2cc**:* R_f_ 0.11 (2% MeOH/DCM); colourless oil; ^1^H NMR 2.204 (s, 3H, C*H*_3_), 2.73 (t, 2H, J 7.1, C*H*_2_-Ph of R_2_), 2.84 (t, 2H, J 7.4, C*H*_2_-Ph of N(2) R_1_), 2.87 (dd, 2H, J 7.0, 6.7, C*H*_2_-Ph of N(6) R_1_), 2.96 (dd, 2H, J 7.0, 6.6, C*H*_2_-N of N(6) R_1_), 2.73 (td, 2H, J 7.0, 5.8, C*H*_2_-N of R_2_), 3.61 (bt, 2H, J 7.4, C*H*_2_-N of N(2) R_1_), 3.92 (s, 2H, C*H*_2_-C_q_-6), 4.24 (s, 2H, C*H*_2_-C_q_-2), 4.95 (bs, 1H, N*H*-CO), 6.77 (bs, 1H, C*H*-5), 6.84 (d, 1H, J 1.6, C*H*-3), 7.14-7.19 (m, 7H, *C*H Ph), 7.21-7.24 (m, 4H, *C*H Ph), 7.2707.31 (m, 4H, *C*H Ph); ^13^C NMR 20.5 (*C*H_3_), 34.5 (*C*H_2_-Ph of N(2) R_1_), 35.2 (*C*H_2_-Ph of N(6) R_1_), 36.4 (*C*H_2_-Ph of R_2_), 42.1 (*C*H_2_-N of R_2_), 45.9 (*C*H_2_-C_q_-2), 49.35 (*C*H_2_-N of N(6) R_1_), 49.44 (*C*H_2_-N of N(2) R_1_), 51.6 (*C*H_2_-C_q_-6), 122.0 (*C*_q_-6), 123.8 (*C*_q_-2), 126.2 (*C*H-4 Ph), 126.4 (*C*H-4 Ph), 126.6 (*C*H-4 Ph), 128.2 (*C*_q_-4), 128.4 (2 *C*H Ph), 128.6 (2 *C*H Ph), 128.68 (2 *C*H Ph), 128.73 (2 *C*H Ph), 128.80 (2 *C*H Ph), 128.85 (2 *C*H Ph), 129.0 (*C*H-5), 129.4 (*C*H-3), 138.6 (*C*_q_-1 Ph), 139.4 (*C*_q_-1 Ph), 139.5 (*C*_q_-1 Ph), 153.1 (*C*_q_-1), 158.6 (*C*=O); ESI MS *m*/*z* 522 [M + 1]^+^ (100), 544 [M + Na]^+^ (4), 1043 [2M + 1]^+^ (8), 1066 [2M + Na + 1]^+^ (2).
*Ligand **3cc**:* R_f_ 0.29 (1% MeOH/DCM); colourless solid; m. p. 107.2–107.3 °C; ^1^H NMR 2.22 (s, 3H, C*H*_3_), 2.73 (t, 4H, J 7.1, C*H*_2_-Ph of R_2_), 2.80 (t, 4H, J 7.2, C*H*_2_-Ph of R_1_), 3.35 (td, 4H, J 6.8, 6.1, C*H*_2_-N of R_2_), 3.40 (bd, 4H, J 7.1, C*H*_2_-N of R_1_), 4.28 (s, 4H, C*H*_2_-C_q_-2 and C*H*_2_-C_q_-6), 4.67 (bs, 1H, N*H*-CO), 6.84 (s, 2H, C*H*-3 and C*H*-5), 7.12 (m, 8H, *C*H-2+6 Ph), 7.18 (m, 2H, *C*H-4 Ph), 7.21-7.28 (m, 10H, *C*H Ph), 10.77 (bs, 1H, O*H*); ^13^C NMR 20.5 (*C*H_3_), 34.3 (*C*H_2_-Ph of R_1_), 36.2 (*C*H_2_-Ph of R_2_), 42.1 (*C*H_2_-N of R_2_), 46.6 (*C*H_2_-C_q_-2 and *C*H_2_-C_q_-6), 49.1 (*C*H_2_-N of R_1_), 124.0 (*C*_q_-2 and *C*_q_-6), 126.3 (*C*H Ph), 126.6 (*C*H Ph), 128.0 (*C*_q_-4), 128.3 (*C*H Ph), big common signals for *C*H Ph at 128.5, 128.7, 128.7 and 128.8, 130.5 (*C*H-3 and *C*H-5), 139.0 (*C*_q_-1 Ph), 139.2 (*C*_q_-1 Ph), 151.7 (*C*_q_-1), 158.9 (*C*=O); ESI MS *m*/*z* 669 [M + 1]^+^ (58), 691 [M + Na]^+^ (100), 707 [M + K]^+^ (11).

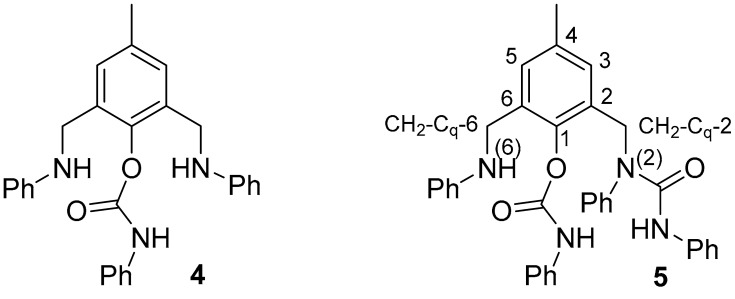


Structure and numeration scheme of ligands **4** and **5**.

*Ligand **4***: R_f_ 0.30 (DCM); pale red solid; m. p. 149.8–150.0 °C; ^1^H NMR 2.28 (s, 3H, C*H*_3_), 3.80 (bs, 2H, N*H*), 4.27 (s, 4H, C*H*_2_), 6.63 (d, 4H, J 7.9, C*H*-2+6 Ph of R_1_), 6.71 (t, 2H, J 7.3, C*H*-4 Ph of R_1_), 7.08 (bt, 1H, J 6.7, C*H*-4 Ph of R_2_), 7.14 (dd, 4H, J 8.5, 7.4, C*H*-3+5 Ph of R_1_), 7.17 (s, 2H, C*H*-3 and C*H*-5), 7.28 (m, 4H, C*H*-2+6 and C*H*-3+5 Ph of R_2_); ^13^C NMR 20.2 (*C*H_3_), 43.8 (*C*H_2_), 113.1 (*C*H-2+6 Ph of R_1_), 117.9 (*C*H-4 Ph of R_1_), 118.9 (*C*H-2+6 Ph of R_2_), 124.1 (*C*H-4 Ph of R_2_), 129.0 (*C*H-3+5 Ph of R_2_), 129.2 (*C*H-3+5 Ph of R_1_), 129.3 (*C*H-3 and *C*H-5), 131.9 (*C*_q_-2 and *C*_q_-6), 136.5 (*C*_q_-4), 137.0 (*C*_q_-1 Ph of R_2_), 144.6 (*C*_q_-1), 147.7 (*C*_q_-1 Ph of R_1_), 151.6 (*C*=O); ESI MS *m*/*z* 226 [M-CONHPh-NPh]^+^ (100), 319 [M-CONPh + 1]^+^ (14), 438 [M + 1]^+^ (41), 876 [2M + 1]^+^ (3).
*Ligand **5***: R_f_ 0.38 (0.5% MeOH/DCM); colourless solid; m. p. 112.6–112.7 °C; ^1^H NMR 2.16 (s, 3H, C*H*_3_), 4.17 (bs, 1H, N*H*), 4.27 (s, 2H, C*H*_2_-C_q_-6), 4.96 (s, 2H, C*H*_2_-C_q_-2), 6.14 (s, 1H, N*H*-CON), 6.61 (dd, 2H, J 8.4, 0.9, C*H*-2+6 Ph-N(6) of R_1_), 6.68 (tt, 1H, J 7.3, 1.0, C*H*-4 Ph-N(6) of R_1_), 6.74 (bs, 1H, C*H*-3), 6.95 (tt, 1H, J 6.8, 1.8, C*H*-4 Ph), 6.99 (tt, 1H, J 7.4, 1.0, C*H*-4 Ph of O R_2_), 7.15, m, 11H, C*H*-5 and 10 C*H* Ph), 7.28 (dd, 2H, J 8.4, 0.9, C*H*-2+6 Ph of O R_2_), 7.32 (tt, 1H, J 7.4, 1.3, C*H*-4 Ph), 7.36 (ddd, 2H, J 8.4, 7.0, 1.4, C*H*-3+5 Ph), 7.64 (bs, 1H, N*H*-COO); ^13^C NMR 20.9 (*C*H_3_), 43.4 (*C*H_2_-C_q_-6), 48.5 (*C*H_2_-C_q_-2), 112.9 (*C*H-2+6 Ph-N(6) of R_1_), 117.5 (*C*H-4 Ph-N(6) of R_1_), 118.9 (*C*H-2+6 Ph of O R_2_), 119.6 (*C*H-2+6 Ph of N R_2_), 123.0 (*C*H-4 Ph), 123.5 (*C*H-4 Ph of O R_2_), 128.4 (*C*H-4 Ph), 128.7 (2 *C*H Ph), 128.8 (2 *C*H Ph), 129.0 (2 *C*H Ph), 129.2 (2 *C*H Ph), 129.4 (*C*H-5), 130.1 (*C*H-3+5 Ph), 130.3 (*C*_q_-2), 130.7 (*C*H-3), 132.7 (*C*_q_-6), 135.7 (*C*_q_-4), 137.5 (*C*_q_-1 Ph), 138.5 (*C*_q_-1 Ph of N R_2_), 140.5 (*C*_q_-1 Ph), 145.1 (*C*_q_-1), 148.2 (*C*_q_-1 Ph of N R_2_), 151.7 (*C*=O-O), 154.2 (*C*=O-N); ESI MS *m*/*z* 557 [M + 1]^+^ (98), 579 [M + Na]^+^ (100), 595 [M + K]^+^ (14), 1135 [2M + Na]^+^ (47).

### 3.4. General Procedure for the Preparation of Ligands **7**

*General procedure for the preparation of chlorides **6**:* To a solution of bis-amine **1** (1 mmol) and pyridine (2 mmol), toluene phosgene (2 mmol, as 15% commercial solution in toluene) was added and the mixture was stirred at room temperature in an argon atmosphere for 2 h. The products were partitioned between toluene and brine. The organic layer was washed with 10 % aq. HCl and then with brine, dried over MgSO_4_, and evaporated to dryness. The product was purified via flash chromatography on silica gel by using a mobile phase with a gradient of polarity from DCM to 0.4% MeOH/DCM. 
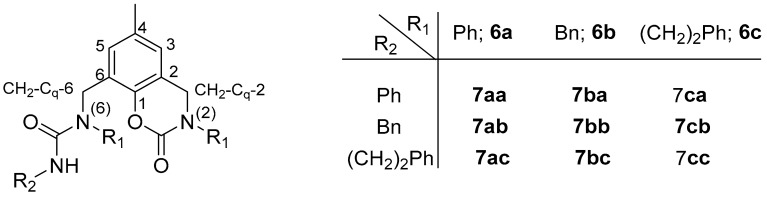


Structure and numeration scheme of intermediates **6**, ligands **7**, and their precursors **8**. This numeration scheme was chosen in an attempt to facilitate a comparison with ligands **2** and **3**.

*Ligand **6a**:* 49% yield; R_f_ 0.65 (1% MeOH/DCM); red solid; m. p. 178.3–178.6 °C; ^1^H NMR 2.37 (s, 3H, C*H*_3_), 4.71 (s, 2H, C*H*_2_-C_q_-2), 5.08 (s, 2H, C*H*_2_-C_q_-6), 6.86 (bs, 1H, C*H*-3), 7.17 (bdd, 2H, J 7.3, 1.0, C*H*-2+6 Ph-N(2)), 7.28 (bs, 1H, C*H*-5), 7.30 (bm, 3H, C*H*-2+6 and C*H*-4 Ph-N(6)), 7.37 (bm, 3H, C*H*-3+5 and C*H*-4 Ph-N(2)), 7.42 (bdd, 2H, J 8.0, 7.5, (bm, 3H, C*H*-3+5 Ph-N(6)); ^13^C NMR 20.9 (*C*H_3_), 50.0 (*C*H_2_-C_q_-6), 50.3 (*C*H_2_-C_q_-2), 118.0 (*C*_q_-2), 122.9 (*C*_q_-6), 125.0 (*C*H-2+6 Ph-N(6)), 125.5 (*C*H-3), 127.2 (*C*H-4 Ph-N(6)), 128.3 (*C*H-2+6 Ph-N(2)), 128.7 (*C*H-4 Ph-N(2)), 129.38 (*C*H-3+5 Ph-N(2)), 129.41 (*C*H-3+5 Ph-N(6)), 129.6 (*C*H-5), 134.3 (*C*_q_-4), 141.5 (*C*_q_-1 Ph-N(2)), 144.6 (*C*_q_-1 Ph-N(6)), 145.6 (*C*_q_-1), 149.8 (O=*C*-O), 150.0 (O=*C*-Cl); ESI MS *m*/*z* 252 [M-NPhCOCl]^+^ (73), 345 [M-NHPh]^+^ (100), 438 [M + 1]^+^ (49), 876 [2M + 1]^+^ (14).
*Ligand **6c**:* 39% yield; R_f_ 0.51 (1% MeOH/DCM); colourless oil; Some NMR signals are duplicated due to hindered rotation; depicted as “A” and “B”; A:B = 1.3:1; ^1^H NMR 2.26 (s, 3H, C*H*_3_, A), 2.28 (s, 3H, C*H*_3_, B), 2.96 (m, 4H, C*H*_2_-Ph of N(6) R_1_, A+B), 3.00 (t, 4H, J 7.4, C*H*_2_-Ph of N(2) R_1_, A+B), 3.60 (t, 2H, J 7.8, C*H*_2_-N of N(6) R_1_, B), 3.69 (t, 4H, J 7.6, C*H*_2_-N of N(2) R_1_, A+B), 3.72 (t, 2H, J 7.8, C*H*_2_-N of N(6) R_1_, A), 4.25 (s, 2H, C*H*_2_-C_q_-2, A), 4.26 (s, 2H, C*H*_2_-C_q_-2, B), 4.57 (s, 2H, C*H*_2_-C_q_-6, A), 4.71 (s, 2H, C*H*_2_-C_q_-6, B), 6.73 (s, 2H, C*H*-3, A+B), 6.93 (s, 1H, C*H*-5, B), 7.05 (s, 1H, C*H*-5, A), 7.20-7.24 (m, 12H, C*H* Ph, A+B), 7.27-7.33 (m, 8H, C*H* Ph, A+B); ^13^C NMR 20.8 (*C*H_3_, A), 20.9 (*C*H_3_, B), 33.3 (*C*H_2_-Ph of N(2) R_1_, A+B), 33.5 (*C*H_2_-Ph of N(6) R_1_, B), 34.8 (*C*H_2_-Ph of N(6) R_1_, A), 46.6 (*C*H_2_-C_q_-6, A), 48.26 (*C*H_2_-C_q_-2, B), 48.30 (*C*H_2_-C_q_-2, A), 48.7 (*C*H_2_-C_q_-6, B), 51.4 (*C*H_2_-N of N(2) R_1_, A+B), 51.7 (*C*H_2_-N of N(6) R_1_, B), 52.8 (*C*H_2_-N of N(6) R_1_, A), 117.3 (*C*_q_-2, A), 117.4 (*C*_q_-2, B), 122.9 (*C*_q_-6, B), 123.0 (*C*_q_-6, A), 125.2 (*C*H-3, B), 125.5 (*C*H-3, A), 126.7 (*C*H Ph), 126.8 (*C*H Ph), 127.5 (*C*H-5, B), 128.68 (*C*H Ph), 128.73 (*C*H Ph), 128.8 (*C*H Ph), 128.9 (*C*H Ph), 129.0 (*C*H Ph), 129.9 (*C*H-5, A), 134.0 (*C*_q_-4, A+B), 137.6 (*C*_q_-1 Ph-N(6), A), 137.8 (*C*_q_-1 Ph-N(6), B), 138.4 (*C*_q_-1 Ph-N(2), A+B), 145.2 (*C*_q_-1, A), 145.7 (*C*_q_-1, B), 150.1 (O=*C*-Cl, A+B), 150.1 (O=*C*-O, A+B); ESI MS *m*/*z* 401 [M-COCl + 1]^+^ (100), 463 [M + 1]^+^ (1.5), 925 [2M + 1]^+^ (2).
*Ligand **8a**:* 2% yield; R_f_ 0.54 (1% MeOH/DCM); deep red solid; m. p. 135.8–135.9 °C; ^1^H NMR 2.27 (s, 3H, C*H*_3_), 4.46 (s, 2H, C*H*_2_-C_q_-6), 4.76 (s, 2H, C*H*_2_-C_q_-2), 6.67 (dd, 2H, J 8.6, 1.0, C*H*-2+6 Ph-N(6)), 6.71 (tt, 1H, J 7.3, 1.0, C*H*-4 Ph-N(6)), 6.80 (bs, 1H, C*H*-3), 7.16 (m, 3H, C*H*-5 and C*H*-3+5 Ph-N(6)), 7.31 (tt, 1H, J 7.3, 1.2, C*H*-4 Ph-N(2)), 7.38 (dd, 2H, J 8.4, 1.2, C*H*-2+6 Ph-N(2)), 7.44 (dd, 2H, J 8.4, 7.4, C*H*-3+5 Ph-N(2)); ^13^C NMR 20.8 (*C*H_3_), 42.4 (*C*H_2_-C_q_-6), 50.6 (*C*H_2_-C_q_-2), 113.2 (*C*H-2+6 Ph-N(6)), 117.66 (*C*_q_-2), 117.70 (*C*H-4 Ph-N(6)), 124.4 (*C*H-3), 125.3 (*C*H-2+6 Ph-N(2)), 126.7 (*C*_q_-6), 127.3 (*C*H-4 Ph-N(2)), 128.9 (*C*H-5), 129.2 (*C*H-3+5 Ph-N(6)), 129.4 (*C*H-3+5 Ph-N(2)), 133.9 (*C*_q_-4), 141.7 (*C*_q_-1 Ph-N(2)), 145.6 (*C*_q_-1), 147.6 (*C*_q_-1 Ph-N(6)), 150.4 (*C*=O).

*General procedure for the preparation of ligands **7**:* A mixture of chloride **6** (1 mmol) and primary amine (2.5 mmol) in dichloroethane was stirred at 50 °C for 19 h. The products were partitioned between dichloroethane and brine. The organic layer was washed with 10 % aq. HCl and then with brine, dried over MgSO_4_, and evaporated to dryness. The product was purified via flash chromatography on silica gel by using a mobile phase with a gradient of polarity from DCM to 1% MeOH/DCM. The yields are given in Table 3.

*Ligand **7aa**:* R_f_ 0.62 (3% MeOH/DCM); colourless solid; m. p. 235.2–235.3 °C; ^1^H NMR 2.34 (s, 3H, C*H*_3_), 4.71 (s, 2H, C*H*_2_-C_q_-2), 5.12 (s, 2H, C*H*_2_-C_q_-6), 6.36 (s, 1H, N*H*), 6.80 (bs, 1H, C*H*-3), 7.01 (tt, 1H, J 7.4, 1.1, C*H*-4 Ph of R_2_), 7.26 (m, 3H, C*H* Ph), 7.29–7.37 (m, 9H, 8 C*H* Ph and C*H*-5 at 7.326), 7.43 (m, 4H, C*H* Ph); ^13^C NMR 21.0 (*C*H_3_), 46.9 (*C*H_2_-C_q_-6), 50.4 (*C*H_2_-C_q_-2), 117.6 (*C*_q_-2), 119.3 (*C*H-2+6 Ph of R_2_), 123.0 (*C*H-4 Ph of R_2_), 124.6 (*C*H-3), 125.1 (2 *C*H Ph), 125.6 (*C*_q_-6), 127.1 (*C*H-4 Ph), 128.2 (*C*H-4 Ph), 128.3 (2 *C*H Ph), 128.8 (2 *C*H Ph), 129.3 (*C*H-5), 129.3 (2 *C*H Ph), 130.2 (2 *C*H Ph), 134.1 (*C*_q_-4), 138.7 (*C*_q_-1 Ph of R_2_), 141.3 (*C*_q_-1 Ph of R_1_), 141.7 (*C*_q_-1 Ph of R_1_), 145.4 (*C*_q_-1), 150.2 (O=*C*-O), 154.5 (O=*C*-N); ESI MS *m*/*z* 226 [PhNHCONHPhCH_3_]^+^ (52), 345 [M-CONHPh + 1]^+^ (73), 464 [M + 1]^+^ (100), 486 [M + Na]^+^ (5), 928 [2M + 1]^+^ (3).
*Ligand **7ab**:* R_f_ 0.42 (3% MeOH/DCM); colourless solid; m. p. 130.9–131.0 °C; ^1^H NMR 2.34 (s, 3H, C*H*_3_), 4.46 (d, 2H, J 4.7, C*H*_2_ of R_2_), 4.72 (s, 2H, C*H*_2_-C_q_-2), 4.78 (bs, 1H, N*H*), 5.08 (s, 2H, C*H*_2_-C_q_-6), 6.79 (bs, 1H, C*H*-3), 7.25 (m, 5H, C*H* Ph), 7.27-7.33 (m, 7H, 6 C*H* Ph and C*H*-5 at 7.298), 7.36 (bt, 2H, J 7.6, C*H*-3+5 Ph), 7.47 (bt, 2H, J 7.7, C*H*-3+5 Ph); ^13^C NMR 21.0 (*C*H_3_), 44.8 (*C*H_2_ of R_2_), 47.0 (*C*H_2_-C_q_-6), 50.5 (*C*H_2_-C_q_-2), 117.6 (*C*_q_-2), 124.4 (*C*H-3), 125.1 (2 *C*H Ph), 126.0 (*C*_q_-6), 127.1 (*C*H-4 Ph), 127.2 (2 *C*H Ph), 127.3 (2 *C*H Ph), 127.8 (*C*H-4 Ph), 128.2 (*C*H-4 Ph), 128.6 (2 *C*H Ph), 129.2 (*C*H-5), 129.3 (*C*H-3+5 Ph), 130.0 (*C*H-3+5 Ph), 134.0 (*C*_q_-4), 139.5 (*C*_q_-1 Ph of R_2_), 141.69 (*C*_q_-1 Ph of R_1_), 141.73 (*C*_q_-1 Ph of R_1_), 145.4 (*C*_q_-1), 150.3 (O=*C*-O), 157.3 (O=*C*-N); ESI MS *m*/*z* 226 [PhNHCONHCH_2_Ph]^+^ (90), 345 [M-CONHCH_2_Ph+1]^+^ (71), 359 [M-CONPh]^+^ (68), 478 [M + 1]^+^ (100), 500 [M + Na]^+^ (7), 956 [2M + 1]^+^ (8).
*Ligand **7ac**:* R_f_ 0.41 (3% MeOH/DCM); colourless oil; ^1^H NMR 2.35 (s, 3H, C*H*_3_), 2.79 (t, 2H, J 6.8, C*H*_2_-Ph of R_2_), 3.48 (td, 2H, J 6.8, 5.8, C*H*_2_-N of R_2_), 4.40 (t, 1H, J 5.5, N*H*), 4.70 (s, 2H, C*H*_2_-C_q_-2), 5.04 (s, 2H, C*H*_2_-C_q_-6), 6.78 (bs, 1H, C*H*-3), 7.10 (m, 4H, 2 C*H*-2+6 Ph), 7.19 (tt, 1H, J 7.4, 1.2, C*H*-4 Ph), 7.24 (tt, 2H, J 7.4, 1.2, C*H*-3+5 Ph of R_2_), 7.26(d, 1H, J 1.1, C*H*-5), 7.27-7.32 (m, 6H, C*H* Ph), 7.41 (ddt, 2H, J 8.4, 7.4, 1.9, C*H*-3+5 Ph); ^13^C NMR 21.0 (*C*H_3_), 36.1 (*C*H_2_-Ph of R_2_), 42.0 (*C*H_2_-N of R_2_), 46.7 (*C*H_2_-C_q_-6), 50.4 (*C*H_2_-C_q_-2), 117.6 (*C*_q_-2), 124.3 (*C*H-3), 125.1 (2 *C*H Ph), 126.1 (*C*_q_-6), 126.3 (*C*H-4 Ph), 127.1 (*C*H-4 Ph), 127.6 (*C*H-4 Ph), 128.2 (2 *C*H Ph), 128.5 (2 *C*H Ph), 128.8 (2 *C*H Ph), 129.1 (*C*H-5), 129.3 (*C*H-3+5 Ph of R_1_), 129.8 (2 *C*H Ph), 133.9 (*C*_q_-4), 139.2 (*C*_q_-1 Ph of R_2_), 141.6 (*C*_q_-1 Ph of R_1_), 141.7 (*C*_q_-1 Ph of R_1_), 145.3 (*C*_q_-1), 150.3 (O=*C*-O), 157.2 (O=*C*-N); ESI MS *m*/*z* 492 [M + 1]^+^ (10), 514 [M + Na]^+^ (35), 1006 [2M + Na]^+^ (47).
*Ligand **7bb**:* R_f_ 0.43 (2% MeOH/DCM); colourless solid; m. p. 109.8–110.4 °C; ^1^H NMR 2.24 (s, 3H, C*H*_3_), 4.24 (s, 2H, C*H*_2_-C_q_-2), 4.45 (s, 2H, C*H*_2_ of R_2_), 4.54 (s, 2H, C*H*_2_-C_q_-6), 4.62 (s, 2H, C*H*_2_-N(6) of R_1_), 4.64 (s, 2H, C*H*_2_-N(2) of R_1_), 4.97 (bs, 1H, N*H*), 6.71 (bs, 1H, C*H*-3), 6.98 (bs, 1H, C*H*-5), 7.17 (dd, 2H, J 7.8, 1.2, C*H*-2+6 Ph), 7.21 (tt, 1H, J 7.4, 1.2, C*H*-4 Ph), 7.25 (tt, 2H, J 7.5, 1.2, C*H*-3+5 Ph of R_2_), 7.27 (m, 3H, C*H* Ph), 7.31–7.36 (m, 7H, C*H* Ph); ^13^C NMR 20.8 (*C*H_3_), 44.88 (*C*H_2_ of R_2_), 44.92 (*C*H_2_-C_q_-6), 46.5 (*C*H_2_-C_q_-2), 50.9 (s, 2H, C*H*_2_-N(6) of R_1_), 52.5 (s, 2H, C*H*_2_-N(2) of R_1_), 116.9 (*C*_q_-2), 124.4 (*C*_q_-6), 124.9 (*C*H-3), 127.0 (*C*H-4 Ph), 127.4 (2 *C*H Ph), 127.4 (*C*H-4 Ph), 127.4 (*C*H-4 Ph), 128.1 (*C*H-5), 128.2 (2 *C*H Ph), 128.3 (2 *C*H Ph), 128.4 (2 *C*H Ph), 128.7 (2 *C*H Ph), 128.9 (2 *C*H Ph), 134.0 (*C*_q_-4), 135.2 (*C*_q_-1 Ph of N(2) R_1_), 137.7 (*C*_q_-1 Ph of N(6) R_1_), 139.5 (*C*_q_-1 Ph of R_2_), 145.2 (*C*_q_-1), 150.5 (O=*C*-O), 158.3 (O=*C*-N); ESI MS *m*/*z* 373 [M-CONCH_2_Ph]^+^ (93), 506 [M + 1]^+^ (100), 528 [M + Na]^+^ (9), 1012 [2M + 1]^+^ (38).
*Ligand **7bc**:* R_f_ 0.39 (2% MeOH/DCM); colourless oil; ^1^H NMR 2.247 (s, 3H, C*H*_3_), 2.77 (t, 2H, J 6.9, C*H*_2_-Ph of R_2_), 3.50 (bt, 2H, J 6.8, C*H*_2_-N of R_2_), 4.26 (s, 2H, C*H*_2_-C_q_-2), 4.46 (s, 2H, C*H*_2_-C_q_-6), 4.54 (s, 2H, C*H*_2_-N(6) of R_1_), 4.63 (bs, 1H, N*H*), 4.66 (s, 2H, C*H*_2_-N(2) of R_1_), 6.71 (bs, 1H, C*H*-3), 6.94 (bs, 1H, C*H*-5), 7.06 (dd, 2H, J 7.7, 1.2, C*H*-2+6 Ph), 7.12 (tt, 1H, J 7.3, 1.3, C*H*-4 Ph), 7.19 (m, 4H, C*H*-3+5 Ph of R_2_ and 2 C*H* Ph), 7.25 (tt, 1H, J 7.3, 1.1, C*H*-4 Ph), 7.29–7.37 (m, 7H, C*H* Ph); ^13^C NMR 20.8 (*C*H_3_), 36.3 (*C*H_2_-Ph of R_2_), 42.2 (*C*H_2_-N of R_2_), 44.9 (*C*H_2_-C_q_-6), 46.6 (*C*H_2_-C_q_-2), 50.7 (*C*H_2_-N(6) of R_1_), 52.5 (s, 2H, C*H*_2_-N(2) of R_1_), 116.8 (*C*_q_-2), 124.5 (*C*_q_-6), 124.8 (*C*H-3), 126.1 (*C*H-4 Ph), 127.3 (2 *C*H Ph), 127.4 (*C*H-4 Ph), 128.1 (*C*H-5), 128.2 (*C*H-4 Ph), 128.3 (2 *C*H Ph), 128.49 (2 *C*H Ph), 128.70 (2 *C*H Ph), 128.71 (2 *C*H Ph), 128.9 (2 *C*H Ph), 133.9 (*C*_q_-4), 135.3 (*C*_q_-1 Ph of N(2) R_1_), 137.6 (*C*_q_-1 Ph of N(6) R_1_), 139.3 (*C*_q_-1 Ph of R_2_), 145.2 (*C*_q_-1), 150.6 (O=*C*-O), 158.3 (O=*C*-N); ESI MS *m*/*z* 520 [M + 1]^+^ (14), 542 [M + Na]^+^ (48), 558 [M + K]^+^ (2), 1062 [2M + Na]^+^ (100).
*Ligand **7ca**:* R_f_ 0.60 (3% MeOH/DCM); colourless solid; m. p. 133.1-133.2 °C; ^1^H NMR 2.27 (s, 3H, C*H*_3_), 2.92 (t, 2H, J 7.2, C*H*_2_-Ph of N(6) R_1_), 3.00 (t, 2H, J 7.4, C*H*_2_-Ph of N(2) R_1_), 3.61 (t, 2H, J 7.2, C*H*_2_-N of N(6) R_1_), 3.68 (t, 2H, J 7.4, C*H*_2_-N of N(2) R_1_), 4.25 (s, 2H, C*H*_2_-C_q_-2), 4.56 (s, 2H, C*H*_2_-C_q_-6), 6.53 (bs, 1H, N*H*), 6.72 (bs, 1H, C*H*-3), 6.98 (bt, 1H, J 7.1, C*H*-4 Ph), 7.02 (bs, 1H, C*H*-5), 7.24 (m, 8H, C*H* Ph), 7.30 (m, 6H, C*H* Ph); ^13^C NMR 20.82 (*C*H_3_), 33.2 (*C*H_2_-Ph of N(2) R_1_), 34.6 (*C*H_2_-Ph of N(6) R_1_), 45.4 (*C*H_2_-C_q_-6), 48.3 (*C*H_2_-C_q_-2), 49.8 (*C*H_2_-N of N(6) R_1_), 51.3 (*C*H_2_-N of N(2) R_1_), 117.2 (*C*_q_-2), 128.4 (*C*H-2+6 Ph of R_2_), 122.6 (*C*H-4 Ph of R_2_), 124.4 (*C*_q_-6), 125.1 (*C*H-3), 126.6 (*C*H-4 Ph), 126.7 (*C*H-4 Ph), 128.70 (2 *C*H Ph), 128.71 (2 *C*H Ph), 128.78 (2 *C*H Ph), 128.79 (2 *C*H Ph), 128.97 (2 *C*H Ph), 129.03 (*C*H-5), 134.0 (*C*_q_-4), 138.3 (*C*_q_-1 Ph of N(2) R_1_), 139.3 (*C*_q_-1 Ph of N(6) R_1_ and *C*_q_-1 Ph of R_2_), 145.5 (*C*_q_-1), 150.1 (O=*C*-O), 155.8 (O=*C*-N); ESI MS *m*/*z* 520 [M + 1]^+^ (11), 542 [M + Na]^+^ (54), 558 [M + K]^+^ (4), 1062 [2M + Na]^+^ (100).
*Ligand **7cb**:* R_f_ 0.39 (3% MeOH/DCM); colourless oil; ^1^H NMR 2.236 (s, 3H, C*H*_3_), 2.90 (dd, 2H, J 7.6, 7.4, C*H*_2_-Ph of N(6) R_1_), 2.98 (dd, 2H, J 7.6, 7.4, C*H*_2_-Ph of N(2) R_1_), 3.57 (dd, 2H, J 7.6, 7.4, C*H*_2_-N of N(6) R_1_), 3.66 (dd, 2H, J 7.6, 7.4, C*H*_2_-N of N(2) R_1_), 4.23 (s, 2H, C*H*_2_-C_q_-2), 4.36 (s, 2H, C*H*_2_ of R_2_), 4.49 (s, 2H, C*H*_2_-C_q_-6), 4.71 (bs, 1H, N*H*), 6.68 (bs, 1H, C*H*-3), 6.95 (bs, 1H, C*H*-5), 7.19 (m, 5H, C*H* Ph), 7.24 (m, 5H, C*H* Ph), 7.28 (m, 5H, C*H* Ph); ^13^C NMR 20.8 (*C*H_3_), 33.2 (*C*H_2_-Ph of N(2) R_1_), 34.8 (*C*H_2_-Ph of N(6) R_1_), 44.8 (*C*H_2_ of R_2_), 46.0 (*C*H_2_-C_q_-6), 48.2 (*C*H_2_-C_q_-2), 49.9 (*C*H_2_-N of N(6) R_1_), 51.3 (*C*H_2_-N of N(2) R_1_), 117.1 (*C*_q_-2), 124.6 (*C*_q_-6), 124.7 (*C*H-3), 126.4 (*C*H-4 Ph), 126.7 (*C*H-4 Ph), 127.0 (*C*H-4 Ph), 127.5 (2 *C*H Ph), 128.1 (*C*H-5), 128.4 (2 *C*H Ph), 128.6 (2 *C*H Ph), 128.7 (2 *C*H Ph), 128.8 (2 *C*H Ph), 128.9 (2 *C*H Ph), 133.9 (*C*_q_-4), 138.4 (*C*_q_-1 Ph of R_2_), 139.1 (*C*_q_-1 Ph of N(2) R_1_), 139.6 (*C*_q_-1 Ph of N(6) R_1_), 145.3 (*C*_q_-1), 150.1 (O=*C*-O), 158.0 (O=*C*-N); ESI MS *m*/*z* 534 [M+1]^+^ (14), 556 [M + Na]^+^ (83), 572 [M + K]^+^ (5), 1090 [2M + Na]^+^ (100).
*Ligand **7cc**:* R_f_ 0.40 (3% MeOH/DCM); colourless oil; ^1^H NMR 2.237 (s, 3H, C*H*_3_), 2.74 (t, 2H, J 7.0, C*H*_2_-Ph of R_2_), 2.82 (dd, 2H, J 7.7, 7.4, C*H*_2_-Ph of N(6) R_1_), 3.00 (dd, 2H, J 7.7, 7.3, C*H*_2_-Ph of N(2) R_1_), 3.44 (dd, 2H, J 7.0, 6.8, C*H*_2_-N of R_2_), 3.46 (dd, 2H, J 7.8, 7.5, C*H*_2_-N of N(6) R_1_), 3.68 (dd, 2H, J 7.7, 7.2, C*H*_2_-N of N(2) R_1_), 4.24 (s, 2H, C*H*_2_-C_q_-2), 4.40 (bs, 1H, N*H*), 4.42 (s, 2H, C*H*_2_-C_q_-6), 6.68 (bs, 1H, C*H*-3), 6.91 (bs, 1H, C*H*-5), 7.14 (m, 4H, C*H* Ph), 7.19 (m, 2H, 2 C*H*-4 Ph), 7.24 (m, 5H, C*H* Ph), 7.29 (m, 4H, C*H* Ph); ^13^C NMR 20.8 (*C*H_3_), 33.3 (*C*H_2_-Ph of N(2) R_1_), 34.7 (*C*H_2_-Ph of N(6) R_1_), 36.2 (*C*H_2_-Ph of R_2_), 42.0 (*C*H_2_-N of R_2_), 44.8 (*C*H_2_-C_q_-6), 48.3 (*C*H_2_-C_q_-2), 49.7 (*C*H_2_-N of N(6) R_1_), 51.3 (*C*H_2_-N of N(2) R_1_), 117.0 (*C*_q_-2), 124.6 (*C*H-3), 124.8 (*C*_q_-6), 126.2 (*C*H-4 Ph), 126.4 (*C*H-4 Ph), 126.7 (*C*H-4 Ph), 128.2 (*C*H-5), 128.5 (2 *C*H Ph), 128.6 (2 *C*H Ph), 128.7 (2 *C*H Ph), 128.77 (2 *C*H Ph), 128.80 (2 *C*H Ph), 128.85 (2 *C*H Ph), 133.8 (*C*_q_-4), 138.4 (*C*_q_-1 Ph of N(2) R_1_), 139.0 (*C*_q_-1 Ph of N(6) R_1_), 139.4 (*C*_q_-1 Ph of R_2_), 145.3 (*C*_q_-1), 150.16 (O=*C*-O), 158.0 (O=*C*-N); ESI MS *m*/*z* 548 [M + 1]^+^ (18), 570 [M + Na]^+^ (100), 586 [M + K]^+^ (7), 1118 [2M + Na]^+^ (99).

### 3.5. Crystallography

Colorless (compounds **2ab**, **2ac**, **2bc**, **3ba**, **4**-solvate, **5**, **6a**, **7aa**, **7ab**, **7bb** and **8a**), yellow (compound **2ba**) or reddish (compound **4**) crystal blocks (compounds **2ab**, **4**-solvate, **7ab**, **7bb** and **8a**), plates (compounds **2ba**, **3ba**, **4**, **5** and **6a**) or prisms (compounds **2ac**, **2bc** and **7aa**) were obtained via recrystallization from suitable solvents. Single crystals with an appropriate size ((0.2 − 0.3) × (0.15 − 0.3) × (0.05 − 0.12) mm^3^) and diffraction quality were carefully selected and mounted on a nylon loop or glass capillary using cryoprotectant oil (Paratone) or epoxy glue. Diffraction data were collected on a SupernovaDual diffractometer equipped with an Atlas CCD detector using micro-focus Mo*K*α radiation (λ = 0.71073 Å). The data were processed using CryAlisPro software 41.117a-64bit [48]. The structures were solved with direct or intrinsic phasing methods and refined using the full-matrix least-squares method on *F*^2^ (ShelxS, ShelxT and ShelxL program packages [49,50] integrated in OLEX v.1.5 software [51]). All non-hydrogen atoms were located successfully from the Fourier map and were refined anisotropically. All hydrogen atoms riding on a parent carbon atom were placed on calculated positions using the following scheme: *U*_eq_ = 1.2 for C-H_aromatic_ = 0.93 Å, C-H_methyl_ = 0.96 Å and C-H_methylenic_ = 0.97 Å. The hydrogen atoms bonded to a heteroatom (e.g., nitrogen or oxygen) were located using the electron density maps. ORTEP-3v2 software [52] was used to illustrate the molecules in the asymmetric unit (ASU). The three-dimensional packing visualization of the molecules was performed using CCDC Mercury [53]. The most important data collection and crystallographic refinement parameters are given in Tables S1–S5. The complete crystallographic data for the reported structures have been deposited in the CIF format with the CCDC as 2287291 to 2287303. These data can be obtained free of charge from The Cambridge Crystallographic Data Centre via www.ccdc.cam.ac.uk/structures (accessed on 7 August 2023).

### 3.6. Isothermal Titration Calorimetry (ITC)

All ITC experiments were performed at 298.15 K (25 °C) using an Affinity ITC isothermal titration calorimeter (TA Instruments, Northampton, MA, USA). The calibration of the Affinity ITC calorimeter was carried out electrically by using electrically generated heat pulses. The active cell volume of the calorimeter was 0.19 mL, with a syringe volume up to 0.2 mL. The reference cell was filled with the nanopure water, with a conductivity not exceeding 0.18 µS cm^−1^ (Adrona, Riga, Latvia). The data, specifically those regarding the heat normalized per mole of injectant, were processed with nanoAnalylze (TA Instruments). A CaCl_2_–EDTA titration (test kit TA Instruments) was performed in order to check the apparatus and processing of the results (*n*—stoichiometry, *K*d, Δ*H*). The typical experiment for assessing polydentate *N*,*O*-ligands includes 30 injections of 2.0 µL into the reaction cell. The reaction cell contains the polydentate ligand at a concentration of 0.15–0.4 mM, whereas the syringe contains 1.0–1.5 mM of buffered solution of chloride or nitrate Ca^2+^, Pb^2+^, K^+^ salts. The first (initial) 2 µL injection was discarded from each data set to remove the effect of titrant diffusion and enable the equilibration process. All reagents, e.g., compounds **2aa** to **7cc** (Table S6), were dissolved directly into the 0.1 M buffer solution with pH 5.0, 7.0 or 8.5. The buffer pH was adjusted with HCl or NaOH, and all solutions were degassed prior to titration. A background titration, consisting of an identical titrant solution but with the buffer solution in the reaction cell only, was removed from each experimental titration to account for the heat of dilution. The titrant was injected at 200–300 s intervals to ensure that the titration peak returned to the baseline before the next injection. For homogeneous mixing in the cell, the stirrer speed was kept constant at 125 rpm.

## 4. Conclusions

In this study, three series of polydentate *N*,*O*-ligands possessing unsymmetrical urea fragments attached to a *p*-cresol scaffold are obtained; mono- and bi-substituted open chain aromatics and fused aryloxazinones. The open-chain compounds **2** and **3** are obtained together via a two-step protocol and separated using column chromatography. The conditions are optimized and individual procedures are developed for each product due to the observed strong dependence of the reaction output on both the bis-amine and carbamoyl chloride substituents. On the contrary, oxazinones **7** are effectively obtained via a common protocol. The products are characterized via 1D and 2D NMR spectra in solution and via single-crystal XRD in solid state. It is shown that the open-chain substituted compounds **2** and **3** are oriented towards the optimal intramolecular H-bonding of the urea’s heteroatoms, while the preferred geometry of oxazinones **7** is driven by intermolecular bonding. A preliminary study on the coordination properties of the products at an approximately neutral pH range is performed using ICT. The results show that most of the compounds do not interact with the particular metal ions tested and it is suggested that the ligands need more acidic or more basic media to bind metal ions.

The protocols offer unlimited possibilities for the preparation of libraries of targets by varying the amines used and via further *N*-substitution as well.

## Figures and Tables

**Table 1:** Synthesis of ligands **2** and **3**.

Entry	Conditions ^a^	Products				
		2	Yield, %	3	Yield, %	Total, %
1	DIPEA, 1a:CC 1:1.5, toluene, 24 h	**2aa**	15	**3aa**	-	51 ^b^
2	**1a**:CC 1:1, benzene, 2 h		19		traces	19
3	**1a**:CC 1:2, toluene, 24 h		39		13	52
4	**1a**:CC 1:3, toluene, 24 h		35		22	57
5	**1a**:CC 1:1, toluene, 2 h	**2ab**	30	**3ab**	traces	30
6	**1a**:CC 1:2, toluene, 24 h		32		23	55
7	**1a**:CC 1:1, benzene, 2 h	**2ac**	traces	**3ac**	-	-
8	**1a**:CC 1:2, benzene, 2 h		17		traces	17
9	**1a**:CC 1:2, toluene, 2 h		39		8	47
10	**1a**:CC 1:2, DCE, 2 h		30		32	62
11	**1b**:CC 1:2, toluene, 24 h	**2ba** ^c^	21	**3ba**	-	21
12	**1b**:CC 1:2, benzene, 24 h		37		7	44
13	**1b**:CC 1:4, toluene, 24 h		19		17	36
14	**1b**:CC 1:1, toluene, 2 h	**2bb**	34	**3bb**	traces	34
15	**1b**:CC 1:2, toluene, 24 h		-		46	46
16	**1b**:CC 1:1, benzene, 2 h	**2bc**	26	**3bc**	-	26
17	**1b**:CC 1:2, benzene, 2 h		16		-	16
18	**1b**:CC 1:2, toluene, 24 h		17		28	45
19	**1b**:CC 1:2, DCE, 2 h		8		27	35
20	**1c**:CC 1:1.5, toluene, 24 h	**2ca**	44	**3ca**	17	61
21	**1c**:CC 1:2, toluene, 24 h		20		43	63
22	**1c**:CC 1:2, toluene, 24 h	**2cb**	15	**3cb**	43	58
23	**1c**:CC 1:3, toluene, 4 h		-		43	43
24	**1c**:CC 1:1, DCE, 2 h		7		traces	7
25	**1c**:CC 1:2, DCE, 2 h		18		16	34
26	**1c**:CC 1:1, benzene, 2 h	**2cc**	42	**3cc**	21	63
27	**1c**:CC 1:2, benzene, 2 h		-		19	19
28	**1c**:CC 1:2, toluene, 24 h		20		39	59
29	**1c**:CC 1:2, DCE, 2 h		-		38	38

^a^ Common conditions: pyridine (unless anything else noted), solvent, rt; CC means carbamic chloride; ^b^ Other isolated products: 25% **4**, 11% **5**; ^c^ Equal to **S1** on Figure 1.

**Table 2:** Selected ^1^H and ^13^C NMR resonances (ppm) of ligands **2** and **3**.

Lig.	C*H*-3 *C*H-3	C*H*-5 *C*H-5	C*H*_2_-C_q_-2 *C*H_2_-C_q_-2	C*H*_2_-C_q_-6 *C*H_2_-C_q_-6
**2aa**	6.372 130.90	7.042 130.14	4.773 50.47	4.363 44.54
**3aa**	6.667/131.03		4.876/49.40	
**2ab**	6.344 130.90	7.042 130.08	4.723 50.81	4.372 44.43
**3ab**	6.678/130.23		4.828/49.63	
**2ac**	6.327 130.83	7.038 129.99	4.667 50.51	4.374 44.36
**3ac**	6.647/130.07		4.778/49.29	
2ba *	6.843 130.79	6.800 129.16	4.362 45.21	4.015 51.62
**3ba**	6.900/132.16		4.501 (br)/49.72	
**2bb**	6.822 129.46	6.741 128.72	4.336 44.76	3.869 51.46
**3bb**	6.833/130.94		4.416/46.94	
**2bc**	6.802 129.43	6.770 128.93	4.295 45.21	3.922 51.44
**3bc**	6.879/130.64		4.893/46.64	
**2ca**	6.939 130.11	6.771 129.08	4.349 46.39	3.952 52.00
**3ca**	6.989/131.93		4.446/46.66	
**2cb**	6.886 129.06	6.726 128.71	4.297 45.80	3.861 52.00
**3cb**	6.879/130.64		4.335/46.64	
**2cc**	6.840 129.36	6.769 129.04	4.244 45.87	3.925 51.62
**3cc**	6.844/130.46		4.284/46.57	

* Published in ref. [43], **S1** in Figure 1.

**Table 3:** Synthesis of ligands **7**.

Starting Chloride	Products					
	Compd.	Yield, %	Compd.	Yield, %	Compd.	Yield, %
**6a**	**7aa**	73	**7ab**	84	**7ac**	84
**6b**	**7ba** ^a^	87	**7bb**	79	**7bc**	71
**6c**	**7ca**	72	**7cb**	71	**7cc**	68

^a^ Equal to **S2** on Figure 1.

**Table 4:** Selected ^1^H and ^13^C NMR resonances (ppm) for the ligands **7**.

Lig.	C*H*-3 *C*H-3	C*H*-5 *C*H-5	C*H_2_*-C_q_-2 *C*H_2_-C_q_-2	C*H_2_*-C_q_-6 *C*H_2_-C_q_-6
**7aa**	6.798 124.56	7.326 129.30	4.710 50.43	5.120 46.86
**7ab**	6.791 124.35	7.298 129.15	4.715 50.49	5.084 47.05
**7ac**	6.777 124.26	7.260 129.09	4.704 50.45	5.037 46.74
7ba *	6.749 125.33	7.015 129.30	4.279 46.56	4.595 45.33
**7bb**	6.709 124.87	6.978 128.11	4.240 46.54	4.537 44.92
**7bc**	6.707 124.80	6.938 128.13	4.260 46.57	4.464 44.87
**7ca**	6.720 125.10	7.020 129.03	4.251 48.26	4.556 45.38
**7cb**	6.685 124.69	6.948 128.09	4.229 48.24	4.490 44.95
**7cc**	6.681 124.62	6.907 128.16	4.244 48.26	4.425 44.80

* Published in ref. [43], **S2** on Figure 1.

**Table 5:** Summary of the ITC results for the titration of either Ca(II) chloride, Pb(II) chloride or KCl with **2ba**, **2bc**, **2cb**, **3bb**, **3cc** derivatives; all interactions are for pH 5.0 and are conducted at 25 °C with 125 rpm continuous stirring; Ka is the association constant; n represents the stoichiometry, e.g., the number of molecules that associate to a metal ion.

Cell Contents [mM]	Syringe Contents [mM]	Δ*H* [kJ·mol^−1^]	Ka.10^6^ [M^−1^]	*n*
EDTA 0.15	CaCl_2_ 0.95	−16.54 ± 0.36	1.30 ± 4.91	0.886
**2ba** 0.20	PbCl_2_ 1.20	62.95 ± 9.85	0.31 ± 0.25	0.869
**2ba** 0.20	CaCl_2_ 1.20	−100.00 ± 7.72	0.024 ± .059	1.856
**2bc** 0.20	PbCl_2_ 1.10	−16.62 ± 0.44	0.14 ± 0.33	0.545
**2cb** 0.30	PbCl_2_ 1.50	−37.27 ± 20.16	0.17 ± 0.60	0.201 *
**3bb** 0.25	KCl 1.10	−13.48 ± 0.45	1.00 ± 0.58	0.444
**3cc** 0.20	KCl 1.20	−16.06 ± 1.48	0.54 ± 0.71	0.424

* Independent model used, but the formation of the complex is likely a combination of models.

## Data Availability

The data of the current study are available from the corresponding authors upon reasonable request.

## Supplementary Materials

The following supporting information can be downloaded at: https://www.mdpi.com/article/10.3390/molecules28186540/s1, Crystallographic data (Tables S1–S13 and Figures S1–S4), ITC study (Table S14 and Figures S5–S10) and Original NMR spectra (Figures S11–S180).

## Sample Availability

Samples of the compounds are not available from the authors.
