# Supplementary material for: Polydentate N,O-Ligands Possessing Unsymmetrical Urea Fragments Attached to a p-Cresol Scaffold
Source: Molecules. 2023 Sep 9;28(18):6540. doi: 10.3390/molecules28186540 (PMC10536015; doi:10.3390/molecules28186540)
Supplement: Supplementary file 1 [file molecules-28-06540-s001.zip › molecules-2584011-supplementary.pdf]

SUPPLEMANTARY MATERIAL

Novel polydentate *N,O*-ligands possessing unsymmetrical urea fragments attached to a p-cresol scaffold

Stanislava E. Todorova,[a] Rusi I. Rusew,[b] Boris L. Shivachev,[b,*] Vanya B. Kurteva[a,*]

[a]*Institute of Organic Chemistry with Centre of Phytochemistry, Bulgarian Academy of Sciences, Acad. G. Bonchev str., bl. 9, 1113 Sofia, Bulgaria.*

[b]*Institute of Mineralogy and Crystallography "Acad. Ivan Kostov", Bulgarian Academy of Sciences, Acad. G. Bonchev str., bl. 107, 1113 Sofia, Bulgaria.*

Table of Contents

Content	Page
Crystallographic data	2
ITC study	10
Original NMR spectra	14

Crystallographic data

Table S1. Most important data collection and crystallographic refinement parameters for compounds **4** and **5**.

compound	**4**	**5**
Empirical formula	$C_{28}H_{27}N_3O_2$	$C_{75}H_{76}BF_4N_8O_7$
Formula weight	437.52	1288.24
Temperature/K	150	150
Crystal system	monoclinic	triclinic
Space group	$P2_1/c$	P-1
a/Å	19.9424(5)	13.8828(5)
b/Å	5.18270(10)	15.2333(6)
c/Å	21.9576(6)	18.0451(8)
α/°	90	77.953(4)
β/°	102.503(3)	75.414(4)
γ/°	90	86.314(3)
Volume/Å^3	2215.62(10)	3611.6(3)
Z	4	2
ρ_{calc}g/cm^3	1.312	1.185
μ/mm^{-1}	0.661	0.683
F(000)	928.0	1358.0
Crystal size/mm^3	0.3 × 0.15 × 0.15	0.2 × 0.2 × 0.1
Radiation	Cu Kα (λ = 1.54184)	Cu Kα (λ = 1.54184)
2Θ range for data collection/°	8.25 to 148.734	6.58 to 148.814
Index ranges	-20 ≤ h ≤ 24, -4 ≤ k ≤ 6, -25 ≤ l ≤ 26	-15 ≤ h ≤ 17, -16 ≤ k ≤ 18, -19 ≤ l ≤ 22
Reflections collected	7691	25508
Independent reflections	4379 [R_{int} = 0.0205, R_{sigma} = 0.0258]	14095 [R_{int} = 0.0391, R_{sigma} = 0.0487]
Data/restraints/parameters	4379/0/311	14095/0/886
Goodness-of-fit on F^2	1.035	1.056
Final R indexes [I>=2σ (I)]	R_1 = 0.0393, wR_2 = 0.0994	R_1 = 0.0722, wR_2 = 0.2121
Final R indexes [all data]	R_1 = 0.0481, wR_2 = 0.1074	R_1 = 0.1005, wR_2 = 0.2307
Largest diff. peak/hole / e Å^{-3}	0.19/-0.18	0.71/-0.46
COD number		

Table S2. Most important data collection and crystallographic refinement parameters for compounds **2ab**, **2ac** and **2ba**.

Compound	**2ab**	**2ac**	**2ba**
Empirical formula	$C_{29}H_{29}N_3O_2$	$C_{30}H_{31}N_3O_2$	$C_{30}H_{31}N_3O_2$
Formula weight	451.55	465.58	465.58
Temperature/K	290	290	290
Crystal system	monoclinic	orthorhombic	orthorhombic
Space group	Cc	$Pca2_1$	Pbca
a/Å	20.7696(6)	9.38080(10)	21.5979(12)
b/Å	25.1669(7)	11.95160(10)	8.3972(5)
c/Å	9.4602(3)	23.8646(3)	28.0580(15)
α/°	90	90	90
β/°	95.402(3)	90	90
γ/°	90	90	90
Volume/Å^3	4922.9(3)	2675.59(5)	5088.7(5)
Z	8	4	8
ρ_{calc}g/cm^3	1.218	1.156	1.215
μ/mm^{-1}	0.610	0.575	0.077
F(000)	1920.0	992.0	1984.0
Crystal size/mm^3	0.2 × 0.15 × 0.15	0.3 × 0.25 × 0.12	0.15 × 0.15 × 0.05
Radiation	Cu Kα (λ = 1.54184)	Cu Kα (λ = 1.54184)	Mo Kα (λ = 0.71073)
2Θ range for data collection/°	8.552 to 148.784	7.396 to 134.156	5.808 to 50.05
Index ranges	-24 ≤ h ≤ 25, -30 ≤ k ≤ 25, -8 ≤ l ≤ 11	-11 ≤ h ≤ 11, -14 ≤ k ≤ 14, -28 ≤ l ≤ 28	-25 ≤ h ≤ 25, -9 ≤ k ≤ 9, -33 ≤ l ≤ 29
Reflections collected	9334	10177	28130
Independent reflections	5883 [R_{int} = 0.0169, R_{sigma} = 0.0190]	4347 [R_{int} = 0.0127, R_{sigma} = 0.0119]	4480 [R_{int} = 0.1257, R_{sigma} = 0.0777]
Data/restraints/parameters	5883/2/639	4347/250/398	4480/0/329
Goodness-of-fit on F^2	1.045	1.064	1.013
Final R indexes [I>=2σ (I)]	R_1 = 0.0374, wR_2 = 0.1031	R_1 = 0.0455, wR_2 = 0.1257	R_1 = 0.0685, wR_2 = 0.1549
Final R indexes [all data]	R_1 = 0.0439, wR_2 = 0.1098	R_1 = 0.0484, wR_2 = 0.1323	R_1 = 0.1431, wR_2 = 0.1881
Largest diff. peak/hole / e Å^{-3}	0.19/-0.12	0.21/-0.20	0.17/-0.17
Flack parameter	0.10(16)	-0.08(9)	

Table S3. Most important data collection and crystallographic refinement parameters for compounds **2ba**, **3ba** and **8a**.

Identification code	**2bc**	**3ba**	**8a**
Empirical formula	$C_{32}H_{35}N_3O_2$	$C_{37}H_{36}N_4O_3$	$C_{25}H_{23}N_2O_2$
Formula weight	493.63	584.70	383.45
Temperature/K	290	290	150
Crystal system	monoclinic	monoclinic	monoclinic
Space group	$P2_1/c$	$P2_1/c$	$P2_1/c$
a/Å	14.0454(2)	9.2587(4)	16.3384(2)
b/Å	11.9778(2)	15.1515(7)	9.67740(10)
c/Å	16.5080(2)	22.7517(11)	13.0491(2)
α/°	90	90	90
β/°	92.8190(10)	95.249(5)	100.9390(10)
γ/°	90	90	90
Volume/Å^3	2773.83(7)	3178.3(3)	2025.75(5)
Z	4	4	4
ρ_{calc}g/cm^3	1.182	1.222	1.257
μ/mm^{-1}	0.581	0.079	0.635
F(000)	1056.0	1240.0	812.0
Crystal size/mm^3	0.3 × 0.25 × 0.12	0.3 × 0.25 × 0.12	0.2 × 0.2 × 0.1
Radiation	Cu Kα (λ = 1.54184)	Mo Kα (λ = 0.71073)	Cu Kα (λ = 1.54184)
2Θ range for data collection/°	6.3 to 148.606	4.418 to 50.372	10.676 to 148.712
Index ranges	$-17 \le h \le 14$, $-14 \le k \le 10$, $-17 \le l \le 20$	$-10 \le h \le 11$, $-18 \le k \le 17$, $-27 \le l \le 22$	$-19 \le h \le 20$, $-11 \le k \le 8$, $-16 \le l \le 16$
Reflections collected	10962	14781	7818
Independent reflections	5485 [R_{int} = 0.0166, R_{sigma} = 0.0168]	5710 [R_{int} = 0.0629, R_{sigma} = 0.0789]	4004 [R_{int} = 0.0130, R_{sigma} = 0.0165]
Data/restraints/parameters	5485/0/347	5710/0/410	4004/0/267
Goodness-of-fit on F^2	1.005	1.059	1.030
Final R indexes [I>=2σ (I)]	R_1 = 0.0427, wR_2 = 0.1226	R_1 = 0.0857, wR_2 = 0.2065	R_1 = 0.0370, wR_2 = 0.0952
Final R indexes [all data]	R_1 = 0.0550, wR_2 = 0.1301	R_1 = 0.1533, wR_2 = 0.2522	R_1 = 0.0410, wR_2 = 0.0984
Largest diff. peak/hole / e Å^{-3}	0.15/-0.20	0.35/-0.26	0.25/-0.15
Flack parameter			

Table S4. Most important data collection and crystallographic refinement parameters for compounds **6a**, **7aa**, **7ba** and **7bb**.

Identification code	**6a**	**7aa**	**7ab**	**7bb**
Empirical formula	$C_{23}H_{19}ClN_2O_3$	$C_{29}H_{25}N_3O_3$	$C_{30}H_{27}N_3O_3$	$C_{32}H_{31}N_3O_3$
Formula weight	406.85	463.52	477.54	505.60
Temperature/K	290	290	290	290
Crystal system	triclinic	orthorhombic	orthorhombic	monoclinic
Space group	P-1	$P2_12_12_1$	$P2_12_12_1$	$P2_1/n$
a/Å	11.7034(5)	8.9403(15)	7.9570(5)	9.9223(5)
b/Å	13.0940(5)	14.870(2)	10.9211(5)	16.4555(8)
c/Å	14.2002(7)	17.283(3)	28.2812(13)	16.3796(10)
α/°	100.903(4)	90	90	90
β/°	95.857(4)	90	90	99.384(5)
γ/°	103.816(3)	90	90	90
Volume/Å^3	2050.12(16)	2297.6(6)	2457.6(2)	2638.6(2)
Z	4	4	4	4
ρ_{calc}g/cm^3	1.318	1.340	1.291	1.273
μ/mm^{-1}	0.213	0.706	0.084	0.656
F(000)	848.0	976.0	1008.0	1072.0
Crystal size/mm^3	0.2 × 0.2 × 0.05	0.25 × 0.2 × 0.18	0.2 × 0.2 × 0.1	0.2 × 0.2 × 0.15
Radiation	Mo Kα (λ = 0.71073)	Cu Kα (λ = 1.54184)	Mo Kα (λ = 0.71073)	Cu Kα (λ = 1.54184)
2Θ range for data collection/°	4.202 to 50.246	7.844 to 146.196	5.708 to 50.37	7.668 to 151.16
Index ranges	-13 ≤ h ≤ 13, -14 ≤ k ≤ 15, -16 ≤ l ≤ 16	-7 ≤ h ≤ 10, -12 ≤ k ≤ 18, -11 ≤ l ≤ 21	-7 ≤ h ≤ 9, -13 ≤ k ≤ 12, -33 ≤ l ≤ 33	-11 ≤ h ≤ 10, -19 ≤ k ≤ 14, -16 ≤ l ≤ 20
Reflections collected	18346	5671	9096	8747
Independent reflections	7299 [R_{int} = 0.0420, R_{sigma} = 0.0534]	3747 [R_{int} = 0.0287, R_{sigma} = 0.0397]	4317 [R_{int} = 0.0270, R_{sigma} = 0.0346]	5122 [R_{int} = 0.0772, R_{sigma} = 0.0617]
Data/restraints/parameters	7299/0/526	3747/0/321	4317/0/368	5122/0/394
Goodness-of-fit on F^2	1.046	1.229	1.094	1.076
Final R indexes [I>=2σ (I)]	R_1 = 0.0626, wR_2 = 0.1544	R_1 = 0.0631, wR_2 = 0.1455	R_1 = 0.0512, wR_2 = 0.1272	R_1 = 0.0843, wR_2 = 0.2241
Final R indexes [all data]	R_1 = 0.0999, wR_2 = 0.1775	R_1 = 0.0765, wR_2 = 0.1522	R_1 = 0.0696, wR_2 = 0.1403	R_1 = 0.1044, wR_2 = 0.2350
Largest diff. peak/hole / e Å^{-3}	0.25/-0.36	0.26/-0.22	0.14/-0.14	0.32/-0.34
Flack parameter		0.0(3)	-0.7(8)	

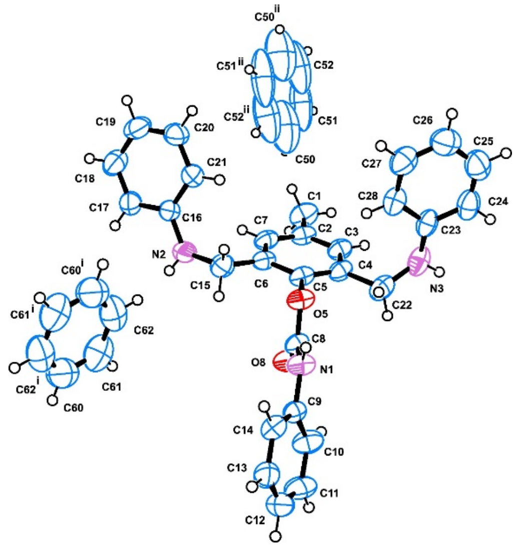

Figure S1. ORTEP view of the *O*-acylated product **4** solvate; symmetry operation: (i)*1–x, 1–y, –z*; (ii) *–x, 1–y, –z*.

Table S5. Most important data collection and crystallographic refinement parameters for compound **2aa** solvate.

Identification code	mo_B_0027_0ma_a_c
Empirical formula	$C_{34}H_{33}N_3O_2$
Formula weight	515.63
Temperature/K	290
Crystal system	monoclinic
Space group	$P2_1/c$
a/Å	11.0093(13)
b/Å	27.623(3)
c/Å	9.7139(8)
α/°	90
β/°	99.384(4)
γ/°	90
Volume/Å^3	2914.6(5)
Z	4
ρ_{calc}g/cm^3	1.175
μ/mm^{-1}	0.073
F(000)	1096.0
Crystal size/mm^3	0.2 × 0.1 × 0.1
Radiation	MoKα (λ = 0.71073)
2Θ range for data collection/°	4.03 to 50.854
Index ranges	-13 ≤ h ≤ 13, -33 ≤ k ≤ 33, -11 ≤ l ≤ 11
Reflections collected	36282
Independent reflections	5104 [R_{int} = 0.1007, R_{sigma} = 0.0535]

Data/restraints/parameters	5104/4/365
Goodness-of-fit on F^2	1.023
Final R indexes [I>=2σ (I)]	R_1 = 0.0574, wR_2 = 0.1247
Final R indexes [all data]	R_1 = 0.0930, wR_2 = 0.1429
Largest diff. peak/hole / e $Å^{-3}$	0.18/-0.20

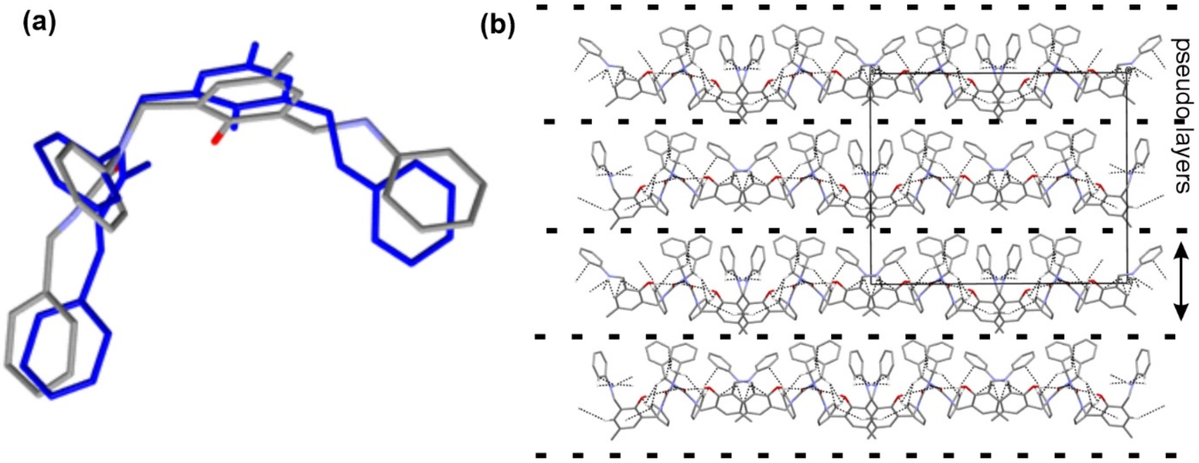

Figure S2. Overlay of the molecules in a **2ab**; rmsd 1.0635 Å (a) and visualization of the pseudo-layers (b).

Table S6. Detected hydrogen bonds and weak interactions in **2ab**.

D	H	A	d(D-H)/Å	d(H-A)/Å	d(D-A)/Å	D-H-A/°
N21	H21	O92[1]	0.81(3)	2.21(3)	2.937(3)	150(3)
N22	H22	O91	0.82(3)	2.15(3)	2.930(3)	159(3)
O52	H52	O92	0.93(5)	1.69(5)	2.612(3)	172(4)
O51	H51	O91	1.01(5)	1.63(5)	2.635(3)	175(4)
O51	H51	N11	1.01(5)	2.67(5)	3.378(3)	128(3)

[1] *1+x,+y,1+z*

Table S7. Detected hydrogen bonds in **2ac**.

D	H	A	d(D-H)/Å	d(H-A)/Å	d(D-A)/Å	D-H-A/°
N2	H2B	O9[1]	0.86	2.11(3)	2.879(3)	148.1
O5	H5	O9	0.80(5)	1.87(5)	2.655(3)	167(4)

[1] *1-1/2+x,1–y,+z*

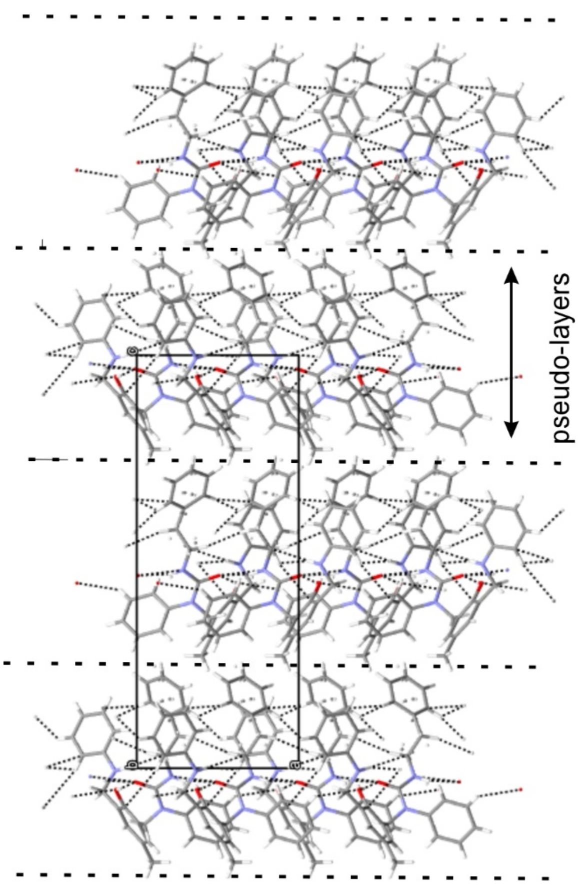

Figure S3. Visualization of the pseudo-layers produced by weak interaction in **2ac**.

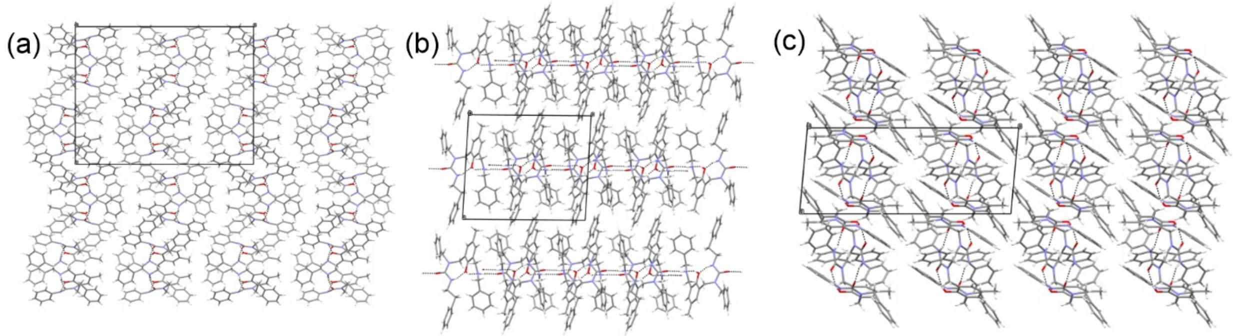

Figure S4. The three-dimensional arrangement of the molecules of (a) **2ba**, (b) **2bc** and (c) **3ba** producing layered motifs.

Table S8. Detected hydrogen bonds in **2ba**.

D	H	A	d(D-H)/Å	d(H-A)/Å	d(D-A)/Å	D-H-A/°
N2	H2	O5	0.85(3)	1.95(3)	2.786(4)	165(3)
O5	H5	N3	0.92(4)	1.69(4)	2.549(3)	154(3)
N3	H3	O9[1]	0.96(4)	1.99(4)	2.932(4)	164(3)

[1] *1+x,1+y,+z*

Table S9. Detected hydrogen bonds in **2bc**.

D	H	A	d(D-H)/Å	d(H-A)/Å	d(D-A)/Å	D-H-A/°
N2	H2	O5	0.894(17)	2.025(17)	2.8628(14)	155.4(14)
N3	H3	O9[1]	0.872(17)	2.082(17)	2.9495(14)	173.5(15)
O5	H5	N3	0.90(3)	1.72(3)	2.5637(13)	157(2)

[1] *1+x,3/2-y,1/2+z*

Table S10. Detected hydrogen bonds in **3ba**.

D	H	A	d(D-H)/Å	d(H-A)/Å	d(D-A)/Å	D-H-A/°
C19	H19	O9	0.93	2.55	3.448(5)	161.4
N4	H4	O5	0.96(4)	1.95(4)	2.901(4)	173(3)
O5	H5	O9	0.86(5)	1.76(6)	2.580(4)	158(5)

Table S11. Detected hydrogen bonds and weak interactions in **7aa**.

D	H	A	d(D-H)/Å	d(H-A)/Å	d(D-A)/Å	D-H-A/°
N3	H3	O9[1]	0.84(7)	2.29(7)	3.051(8)	150(6)
C23	H23	O17	0.93	2.33	2.873(9)	116.7

[1] *3/2-x,1-y,-1/2+z*

Table S12. Detected hydrogen bonds and weak interactions in **7ab**.

D	H	A	d(D-H)/Å	d(H-A)/Å	d(D-A)/Å	D-H-A/°
C11	H11	O17[1]	0.93	2.60	3.484(6)	159.8
C8	H8A	O17[1]	0.97	2.57	3.473(6)	155.7
N3	H3	O9[2]	0.93(5)	2.62(5)	3.373(5)	138(4)

[1] *-1/2+x,1/2-y,1-z;* [2] *1/2+x,3/2-y,1-z*

Table S13. Detected hydrogen bonds and weak interactions in **7bb**.

D	H	A	d(D-H)/Å	d(H-A)/Å	d(D-A)/Å	D-H-A/°
C26	H26A	O5	0.97	2.52	3.144(4)	122.3
C26	H26B	O9[1]	0.97	2.52	3.339(4)	142.0
N3	H3	O9[1]	0.81(5)	2.11(5)	2.873(4)	157(4)

[1] *1-x,1-y,1-z*

ITC study

Table S14. ITC sensed interaction between **2**-**7** and metal salts at different pH.

Ligand	pH 5.0	pH 7.0	pH 8.5
**2aa**	no	no	no
**2ab**	no	no	no
**2ac**	no	no	no
**3aa**	no	no	no
**3ab**	no	no	no
**3ac**	no	no	no
**2ba**	Pb/Ca	no	no
**2bb**	no	no	no
**2bc**	Pb	no	no
**3ba**	no	no	no
**3bb**	K	no	no
**3bc**	no	no	no
**2ca**	no	no	no
**2cb**	Pb	no	no
**2cc**	no	no	no
**3ca**	no	no	no
**3cb**	no	no	no
**3cc**	K	no	no
**7aa**	no	no	no
**7ac**	no	no	no
**7ba**	no	no	no
**7bb**	no	no	no
**7bc**	no	no	no
**7ca**	no	no	no
**7cb**	no	no	no

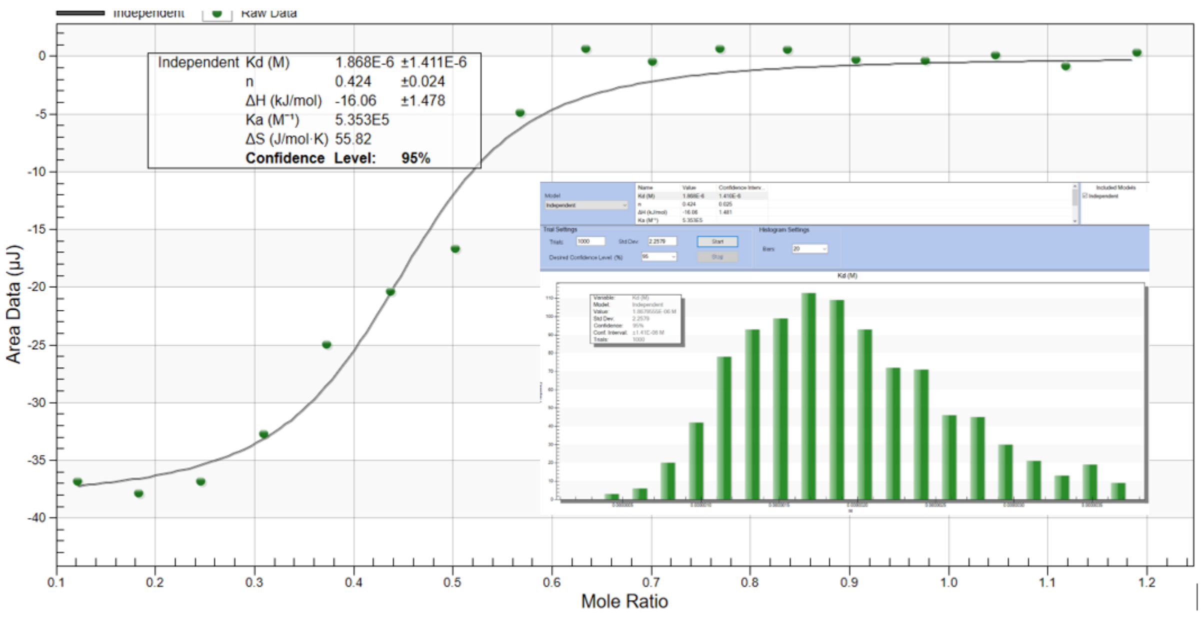

Figure S5. Visualization of ITC fit using a simple one set of identical non-interacting binding site model for **3cc** vs KCl providing K_a, enthalpy and n.

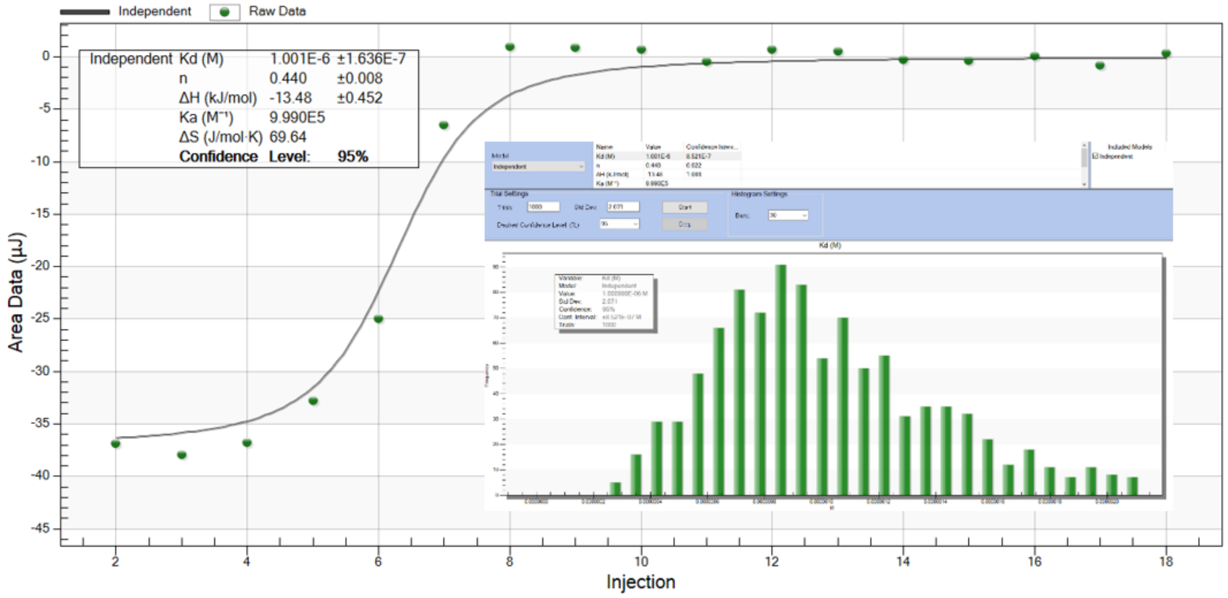

Figure S6. Visualization of ITC fit using a simple one set of identical non-interacting binding site model for **3bb** vs KCl.

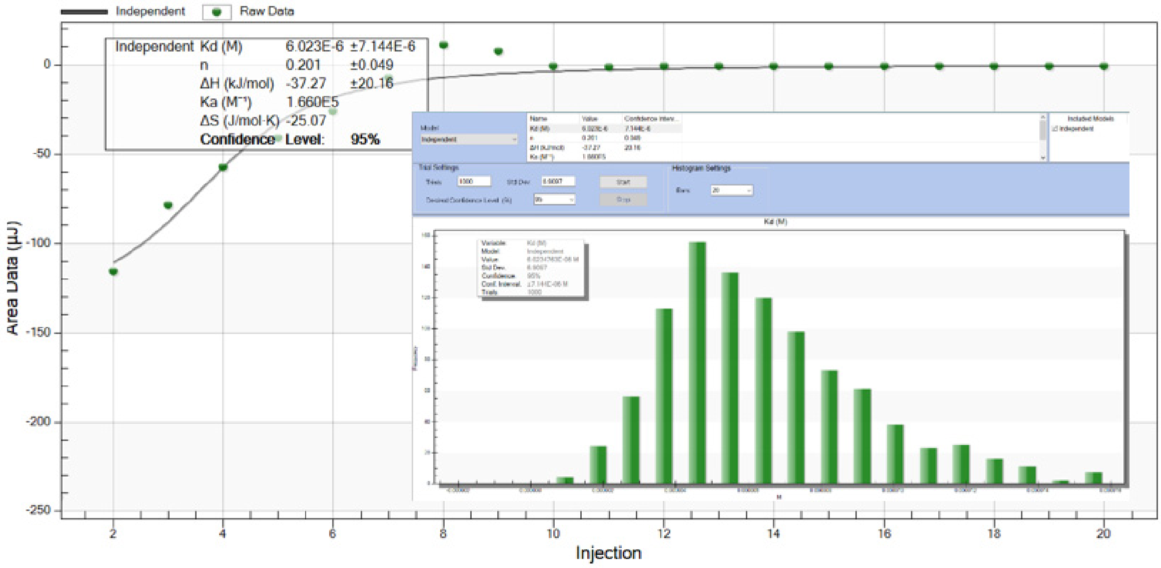

Figure S7. Visualization of ITC fit using a simple one set of identical non-interacting binding site model for **2cb** vs $PbCl_2$; the independent model has been selected as the other tested models (competitive, cooperative, multiples sites, sequential) did not produce a better fit.

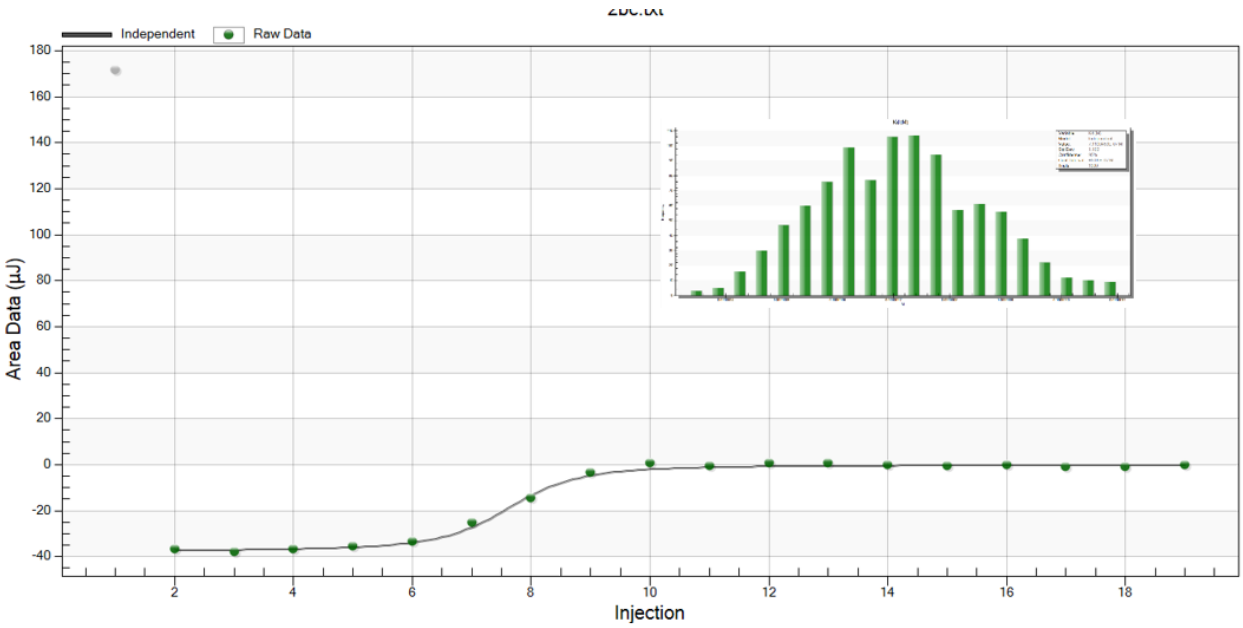

Figure S8. Visualization of ITC fit using a simple one set of identical non-interacting binding site model for **2bc** vs $PbCl_2$.

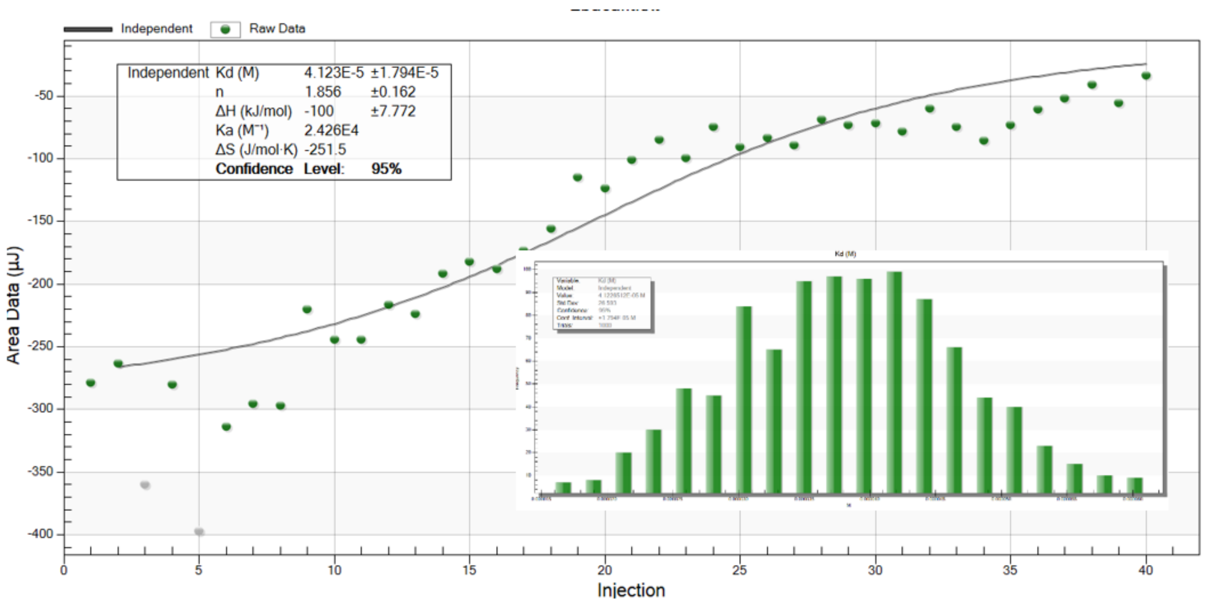

Figure S9. Visualization of ITC fit using a simple one set of identical non-interacting binding site model for **2ba** vs $PbCl_2$.

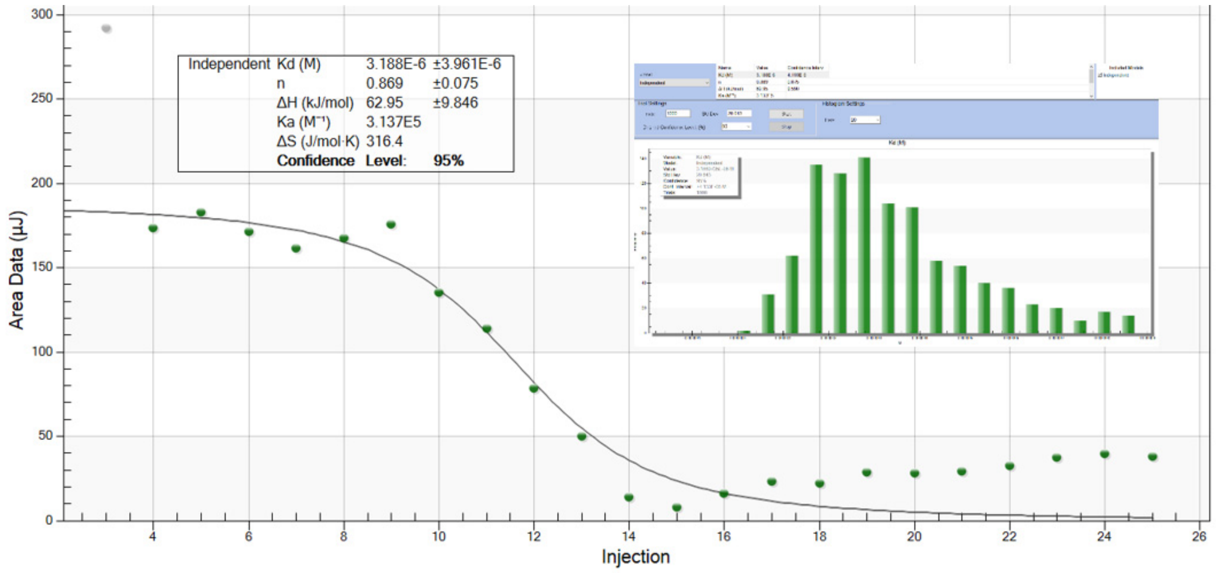

Figure S10. Visualization of ITC fit using a simple one set of identical non-interacting binding site model **2ba** vs $CaCl_2$.

Original NMR spectra

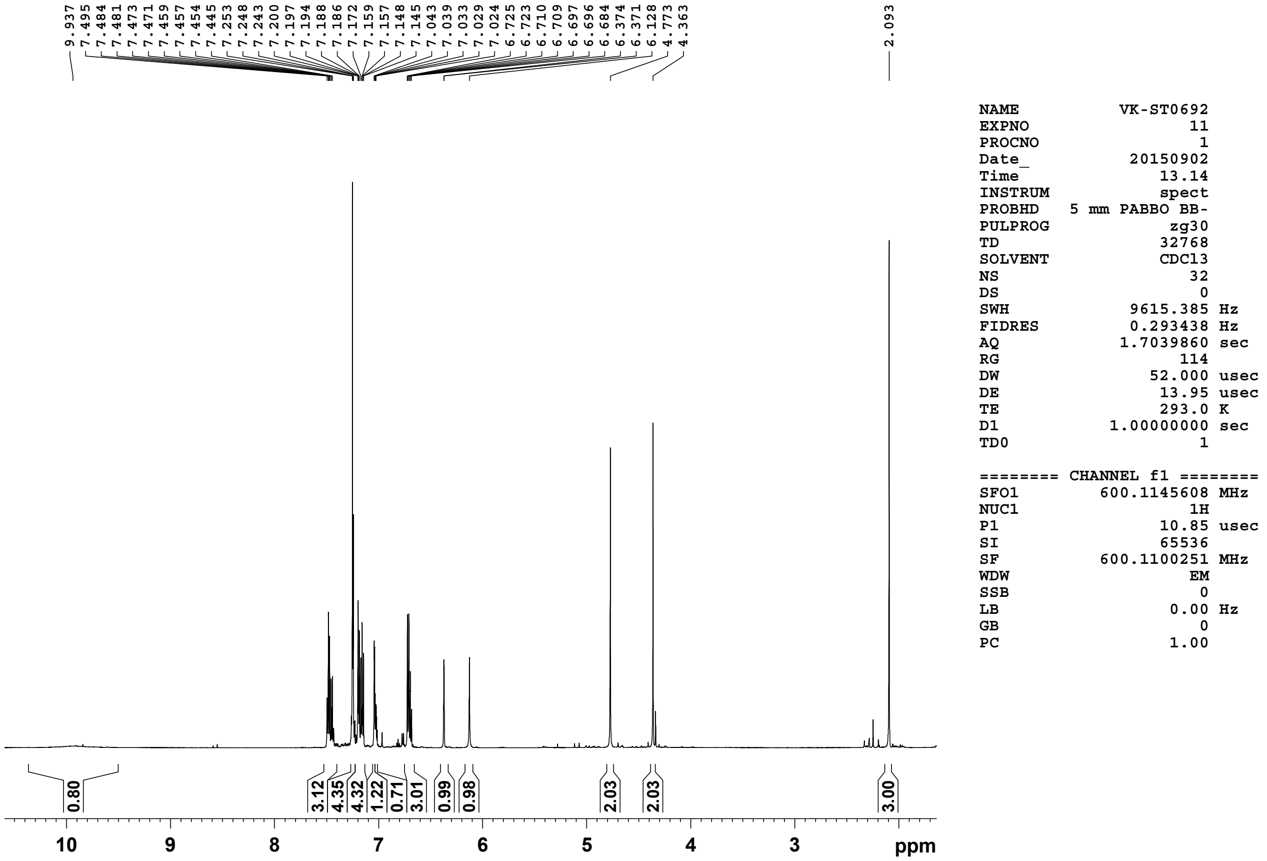

Figure S11. ^{1}H NMR spectrum of compound **2aa**.

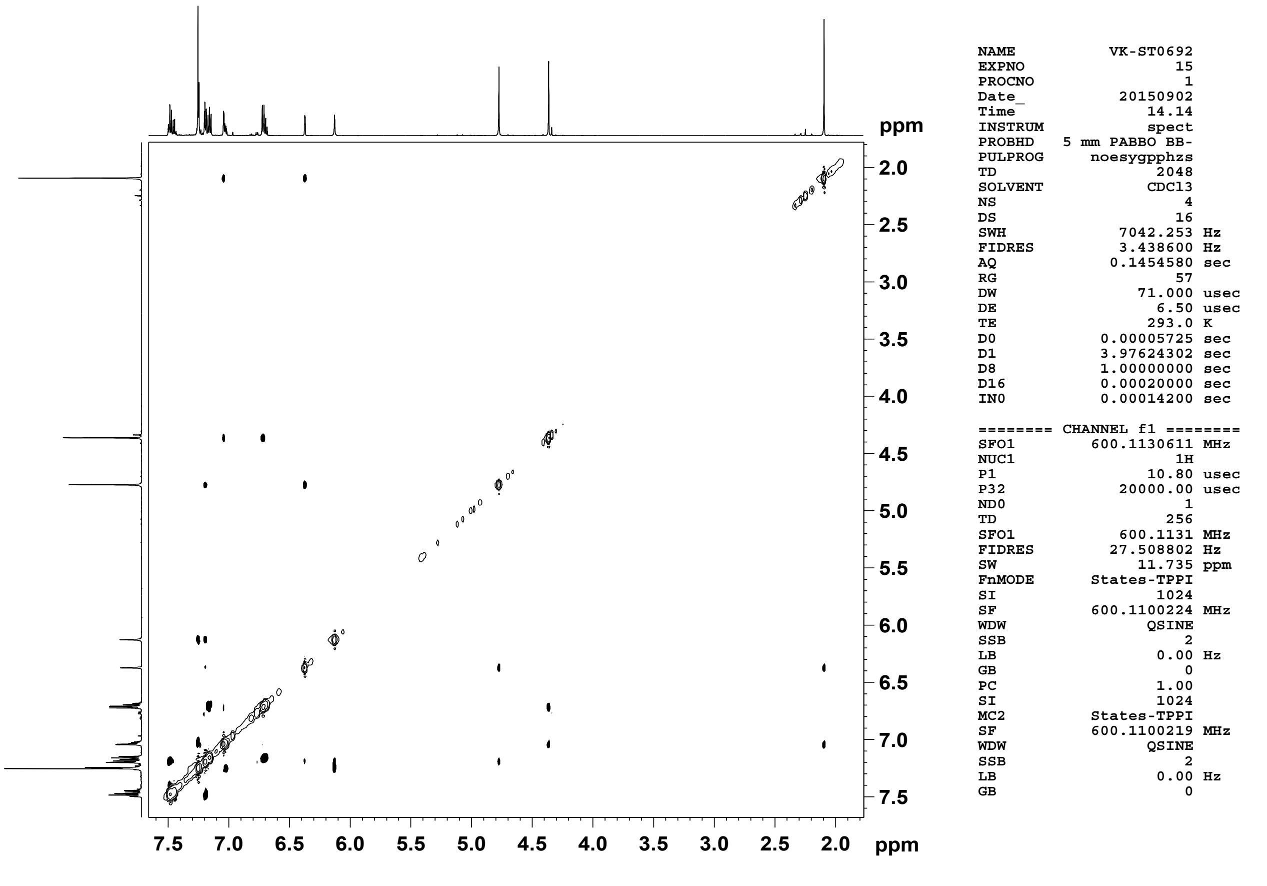

Figure S12. ^{1}H-^{1}H NOESY spectrum of compound **2aa**.

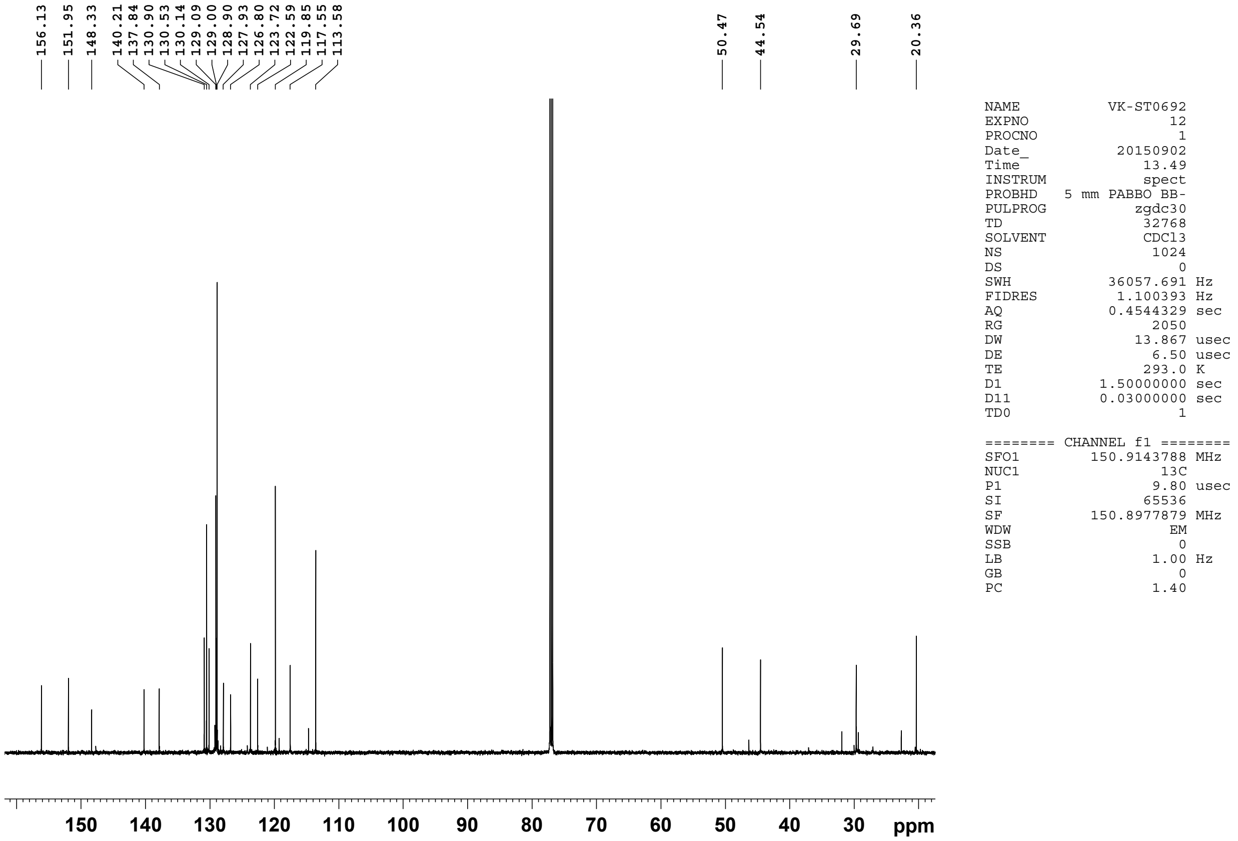

Figure S13. ^{13}C NMR spectrum of compound **2aa**.

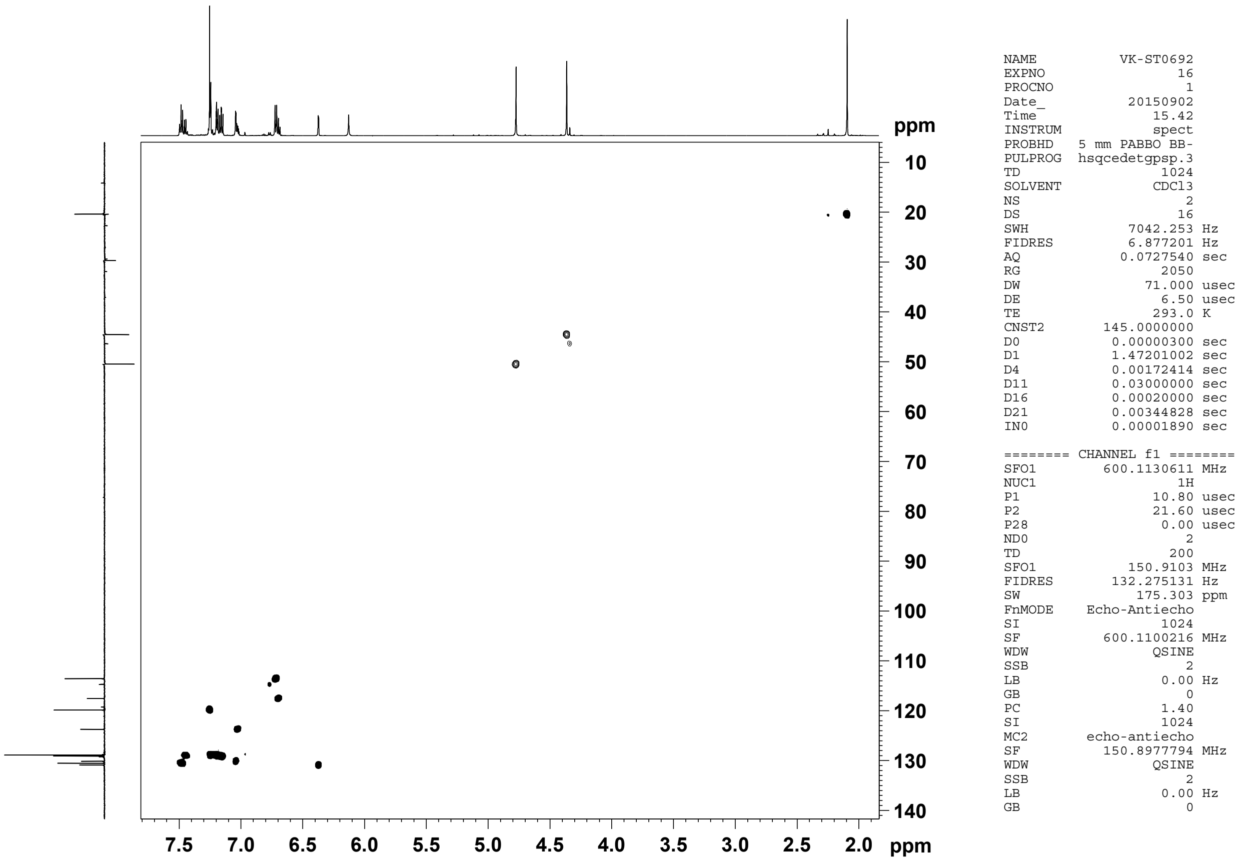

Figure S14. ^{1}H-^{13}C HSQC spectrum of compound **2aa**.

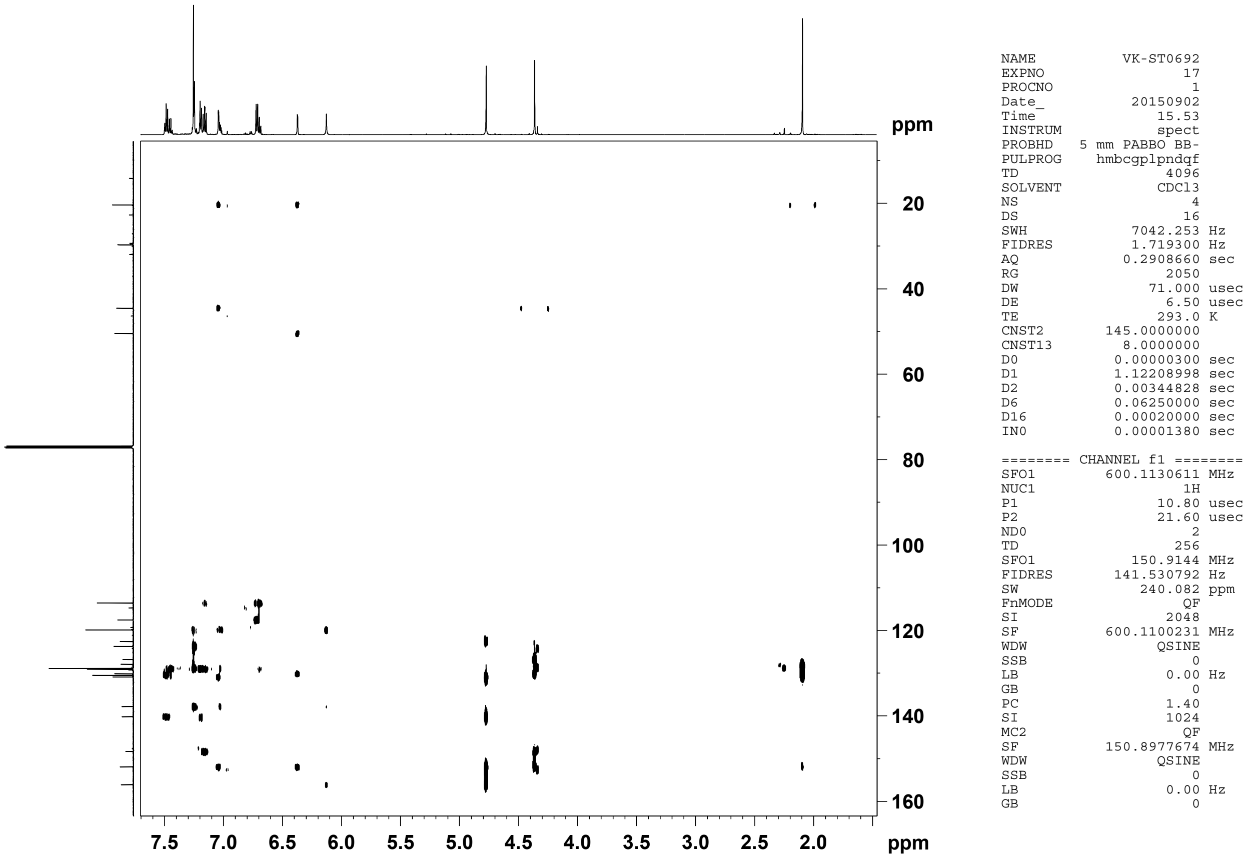

Figure S15. ^{1}H-^{13}C HMBC spectrum of compound **2aa**.

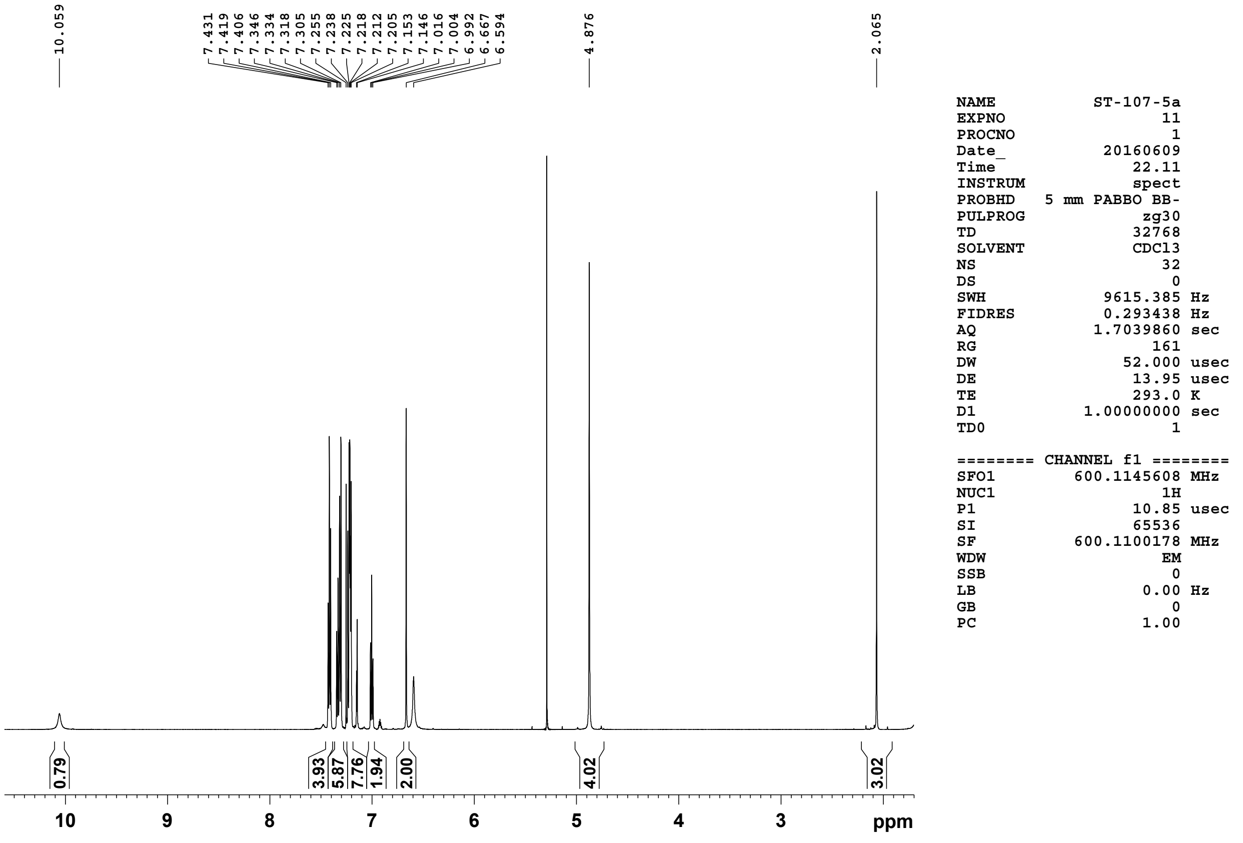

Figure S16. ^{1}H NMR spectrum of compound **3aa**.

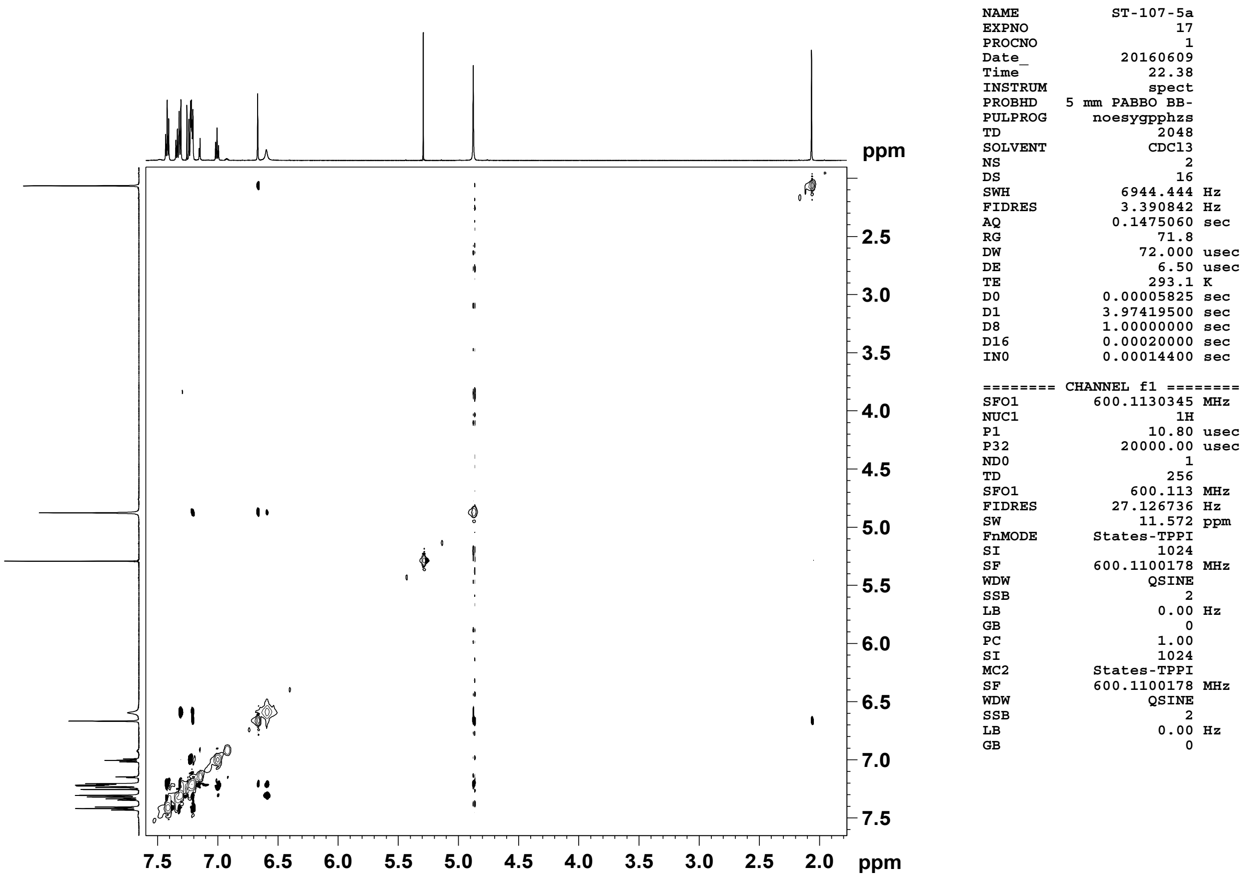

Figure S17. ^{1}H-^{1}H NOESY spectrum of compound **3aa**.

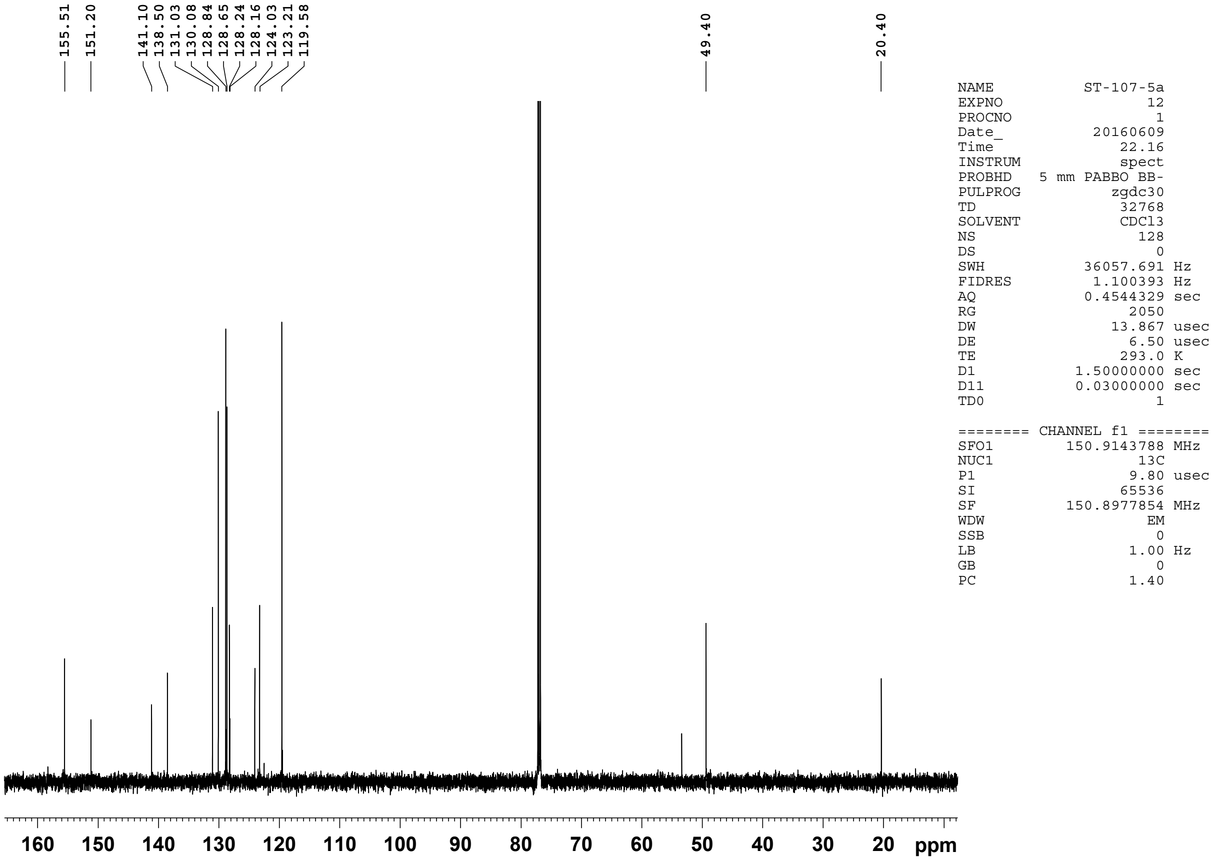

Figure S18. ^{13}C NMR spectrum of compound **3aa**.

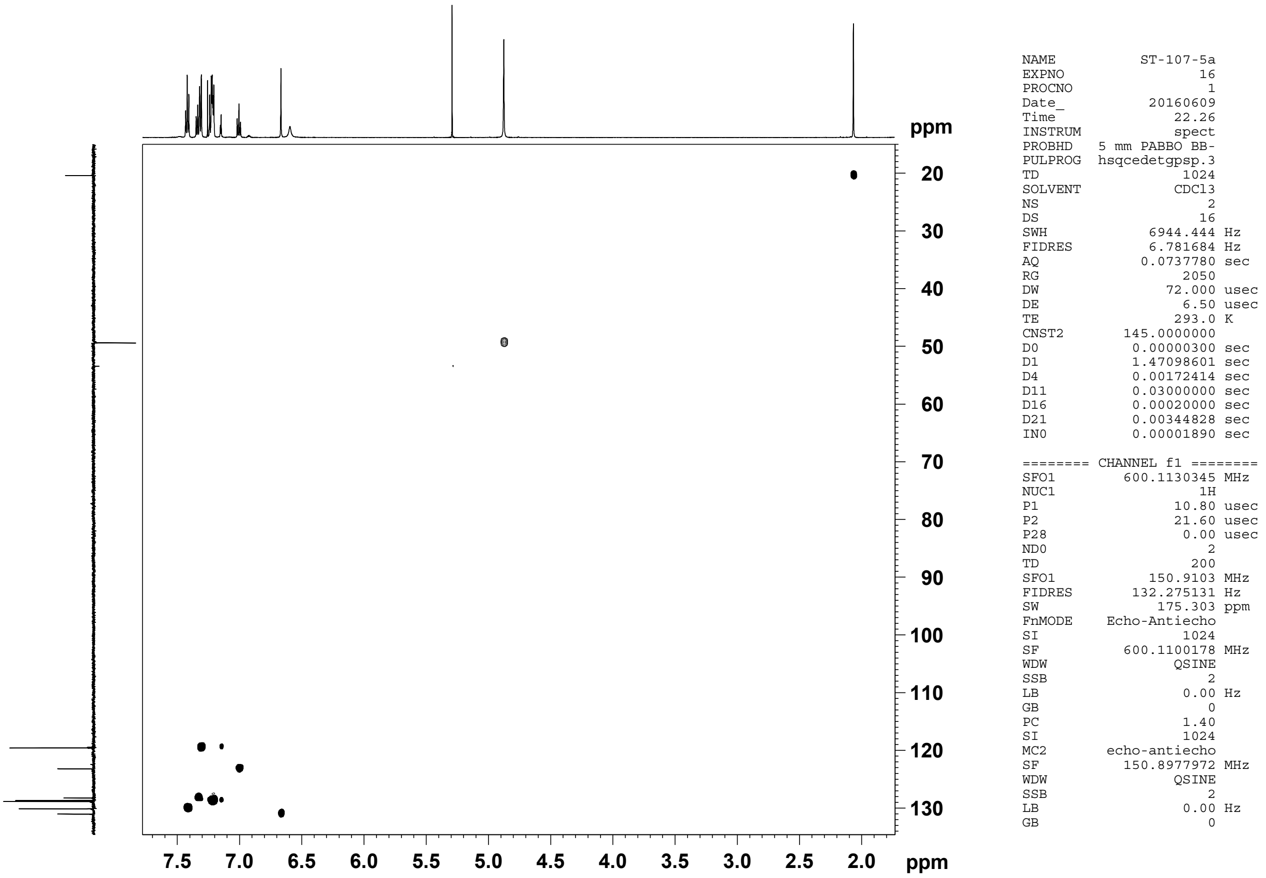

Figure S19. ^{1}H-^{13}C HSQC spectrum of compound **3aa**.

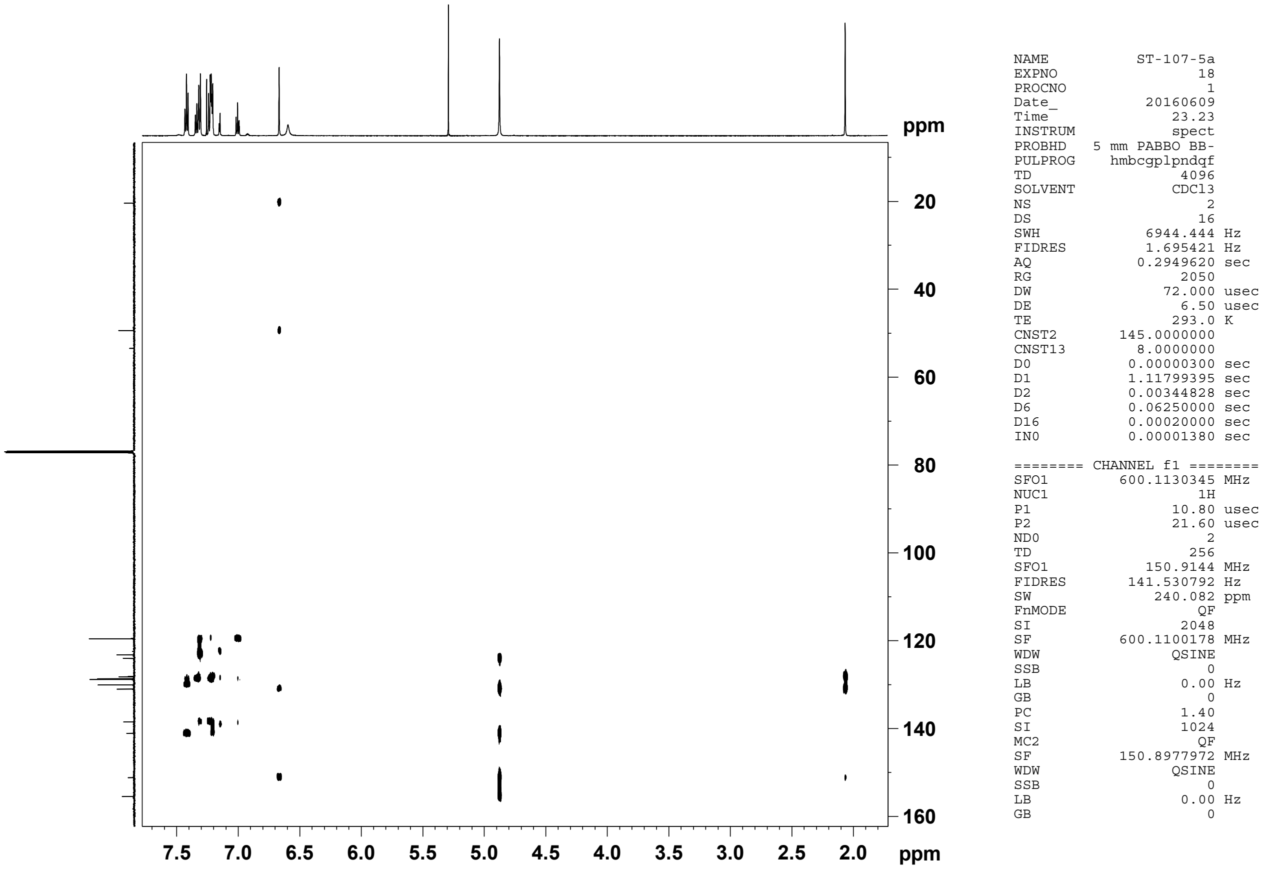

Figure S20. ^{1}H-^{13}C HMBC spectrum of compound **3aa**.

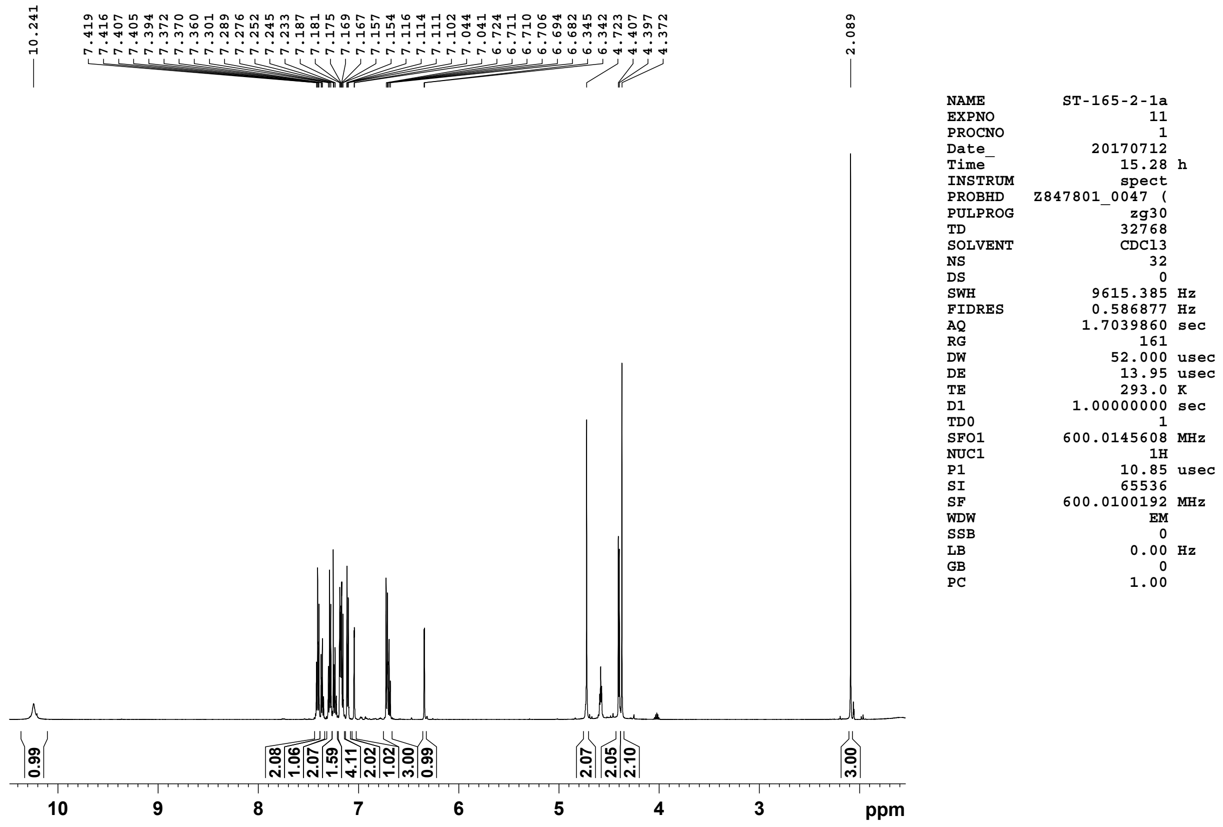

Figure S21. ¹H NMR spectrum of compound **2ab**.

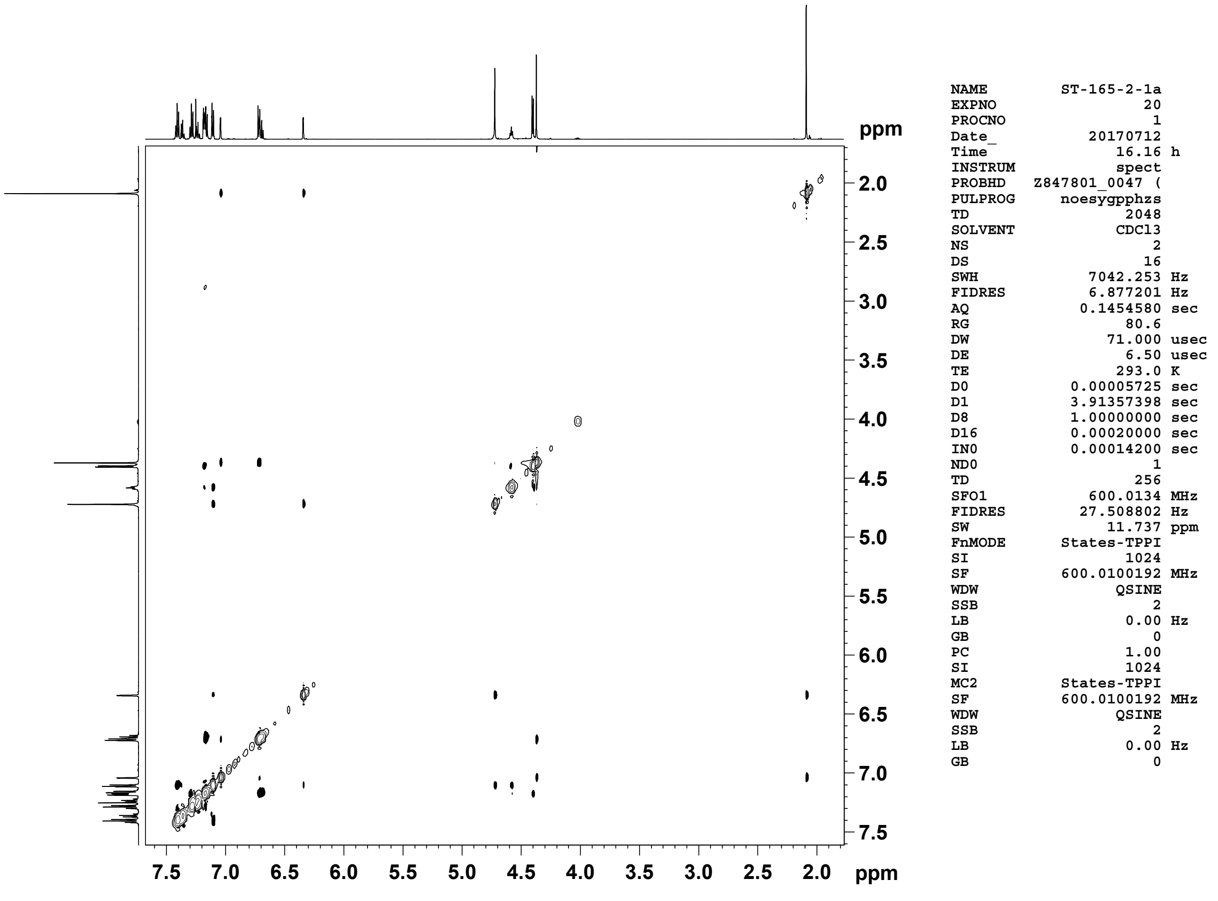

Figure S22. ¹H-¹H NOESY spectrum of compound **2ab**.

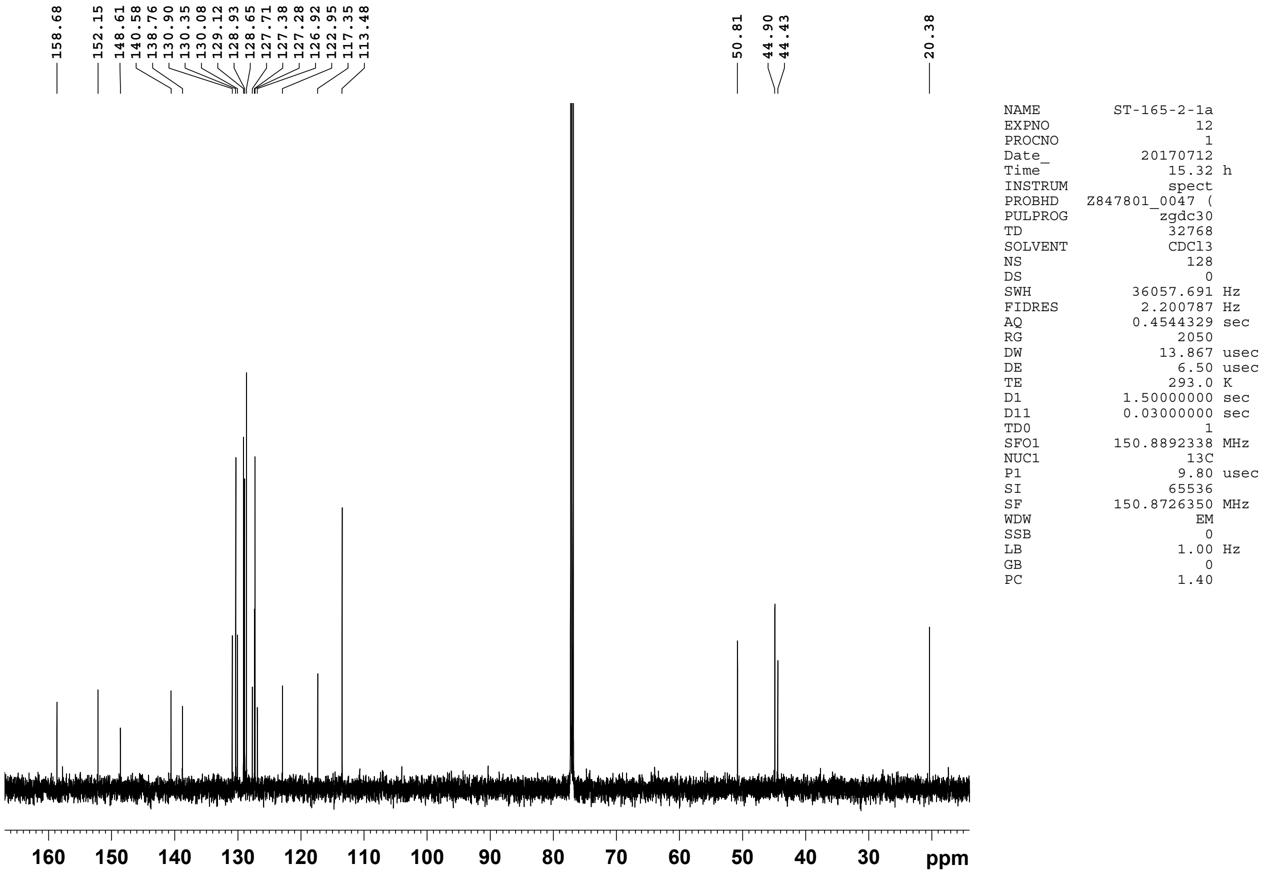

Figure S23. ^{13}C NMR spectrum of compound **2ab**.

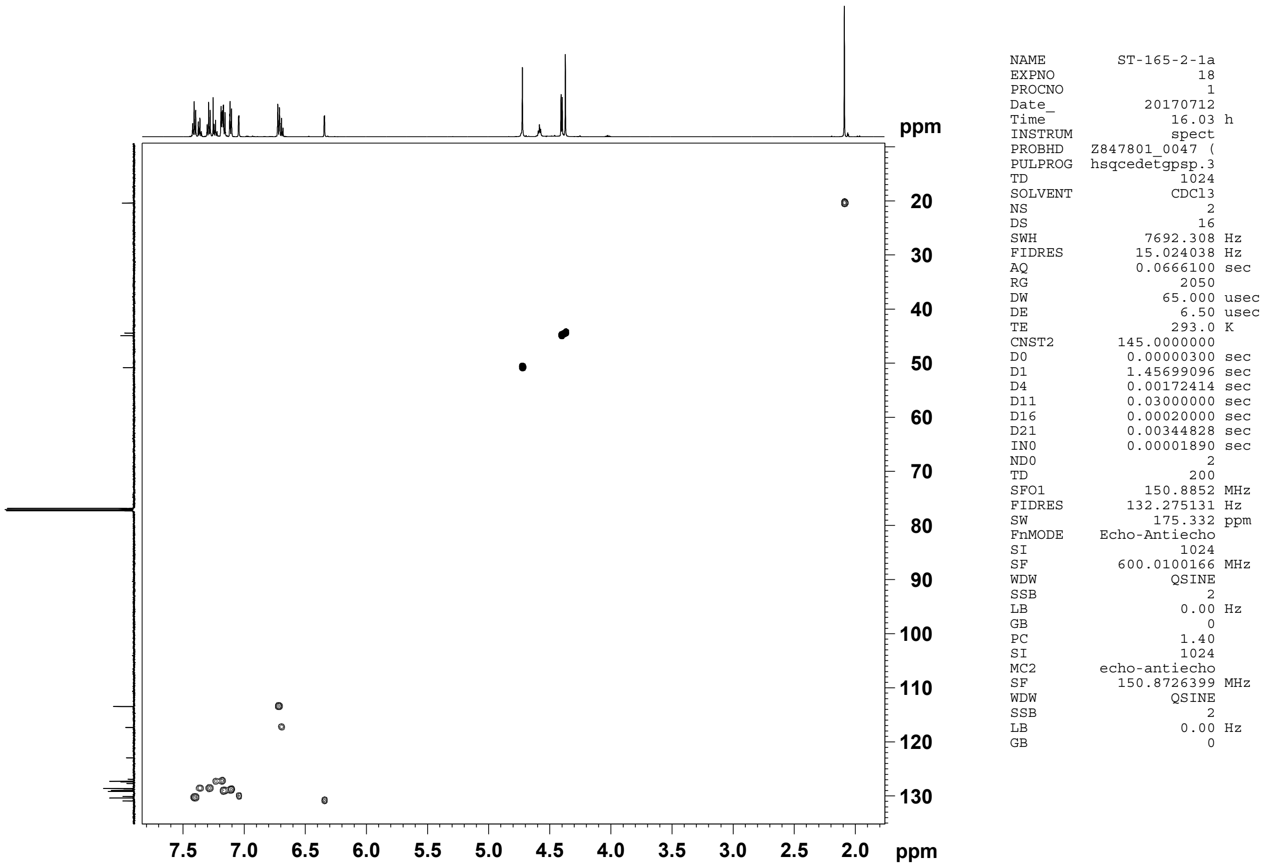

Figure S24. ^{1}H-^{13}C HSQC spectrum of compound **2ab**.

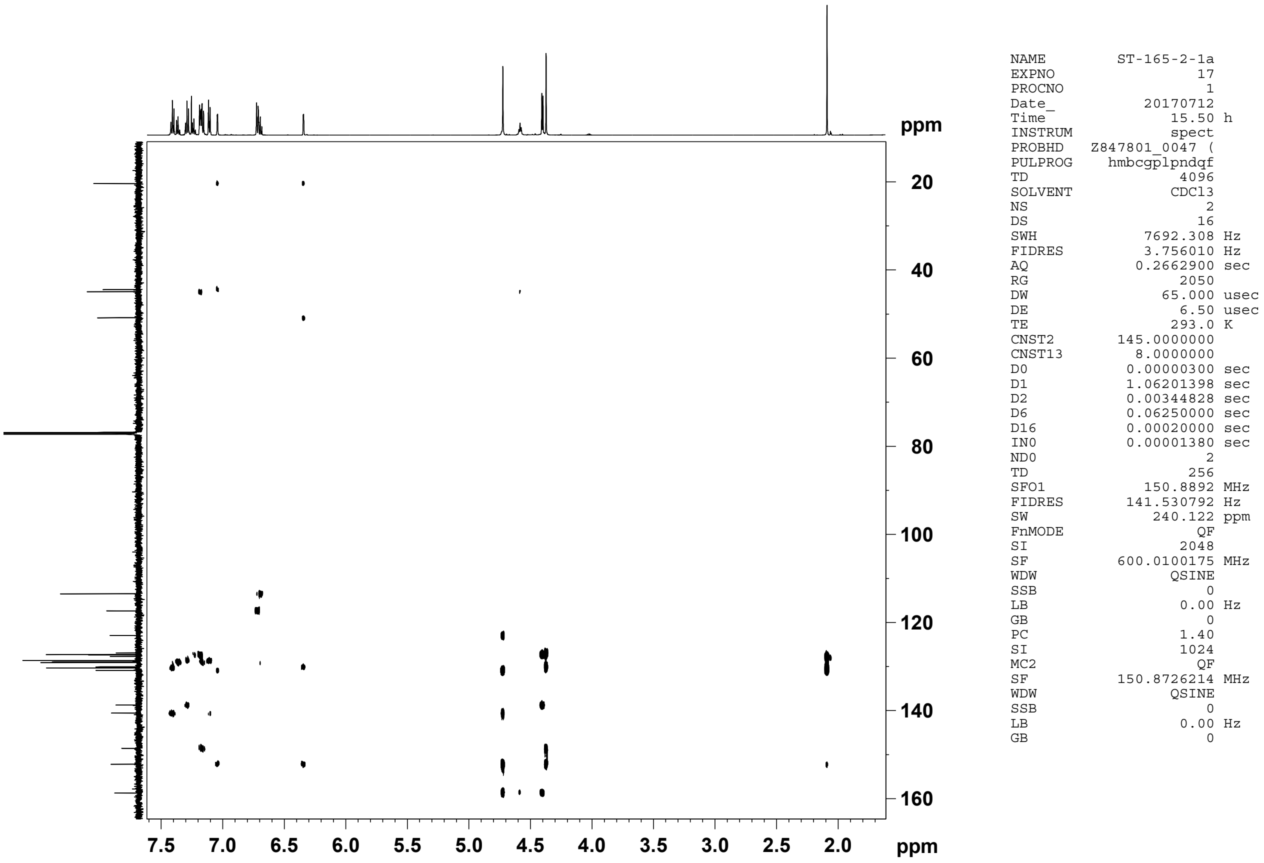

Figure S25. ^1H-^{13}C HMBC spectrum of compound **2ab**.

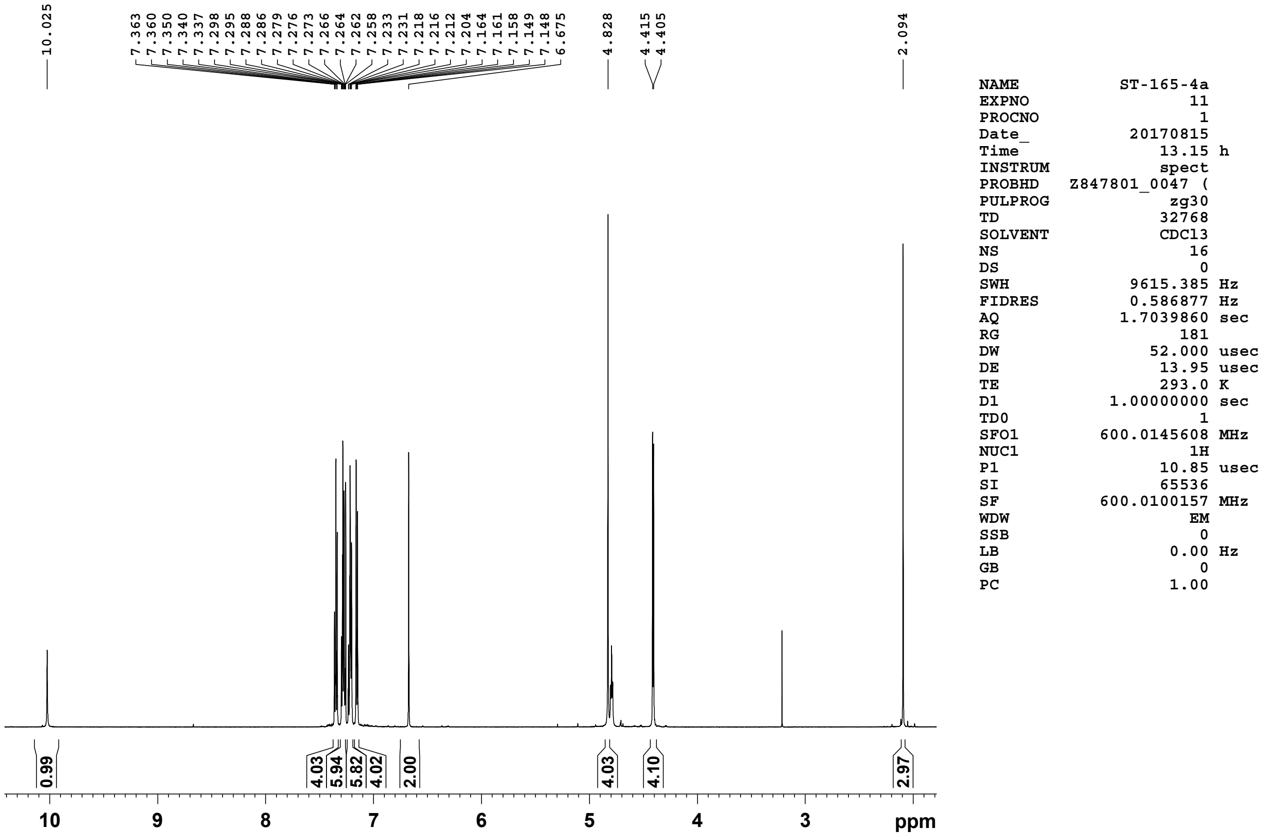

Figure S26. ^1H NMR spectrum of compound **3ab**.

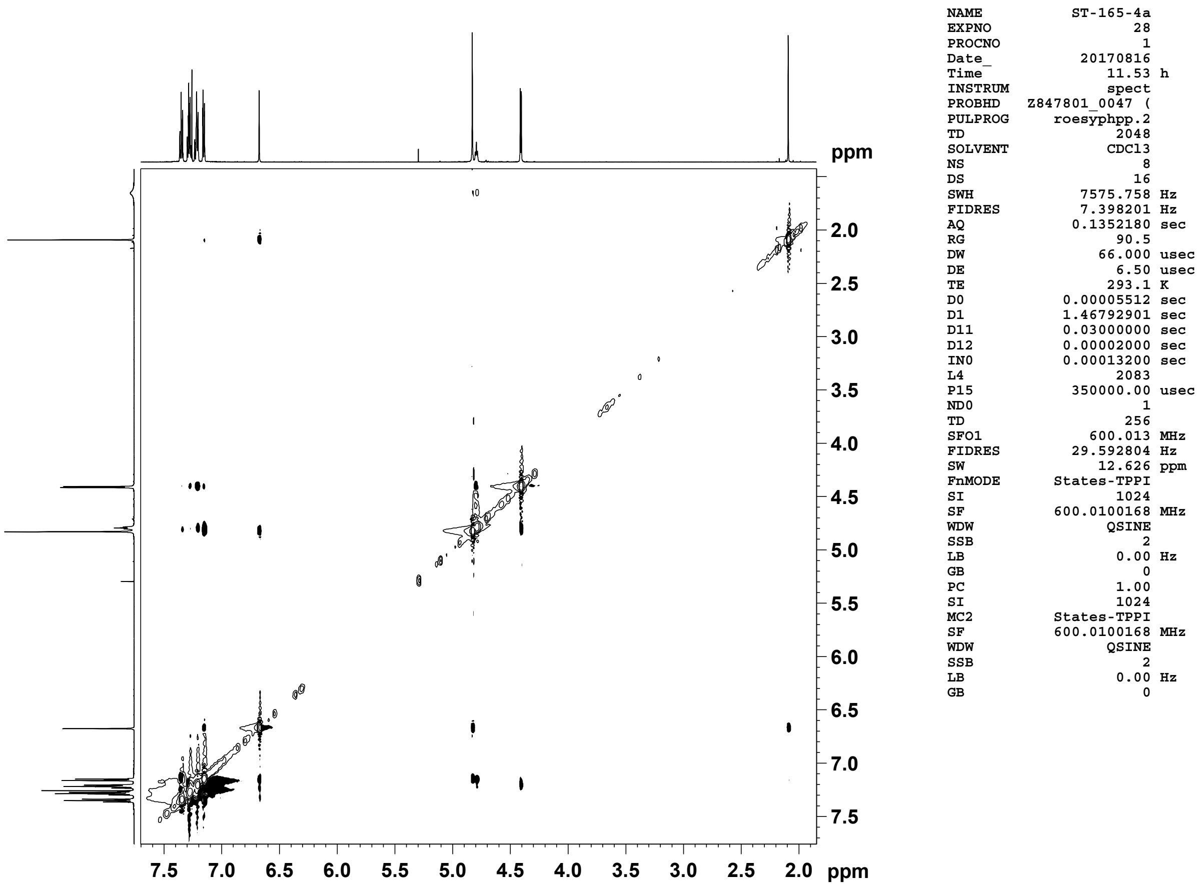

Figure S27. ^1H-^1H NOESY spectrum of compound **3ab**.

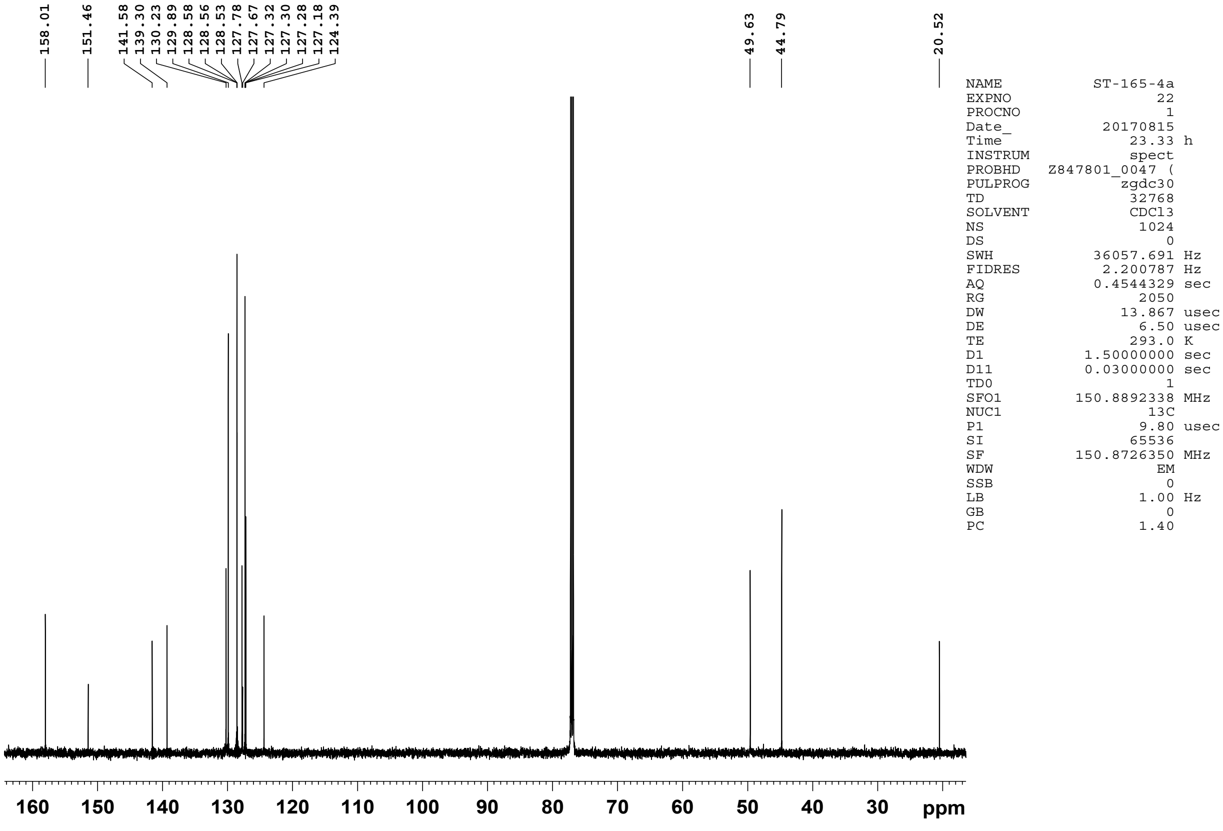

Figure S28. ^{13}C NMR spectrum of compound **3ab**.

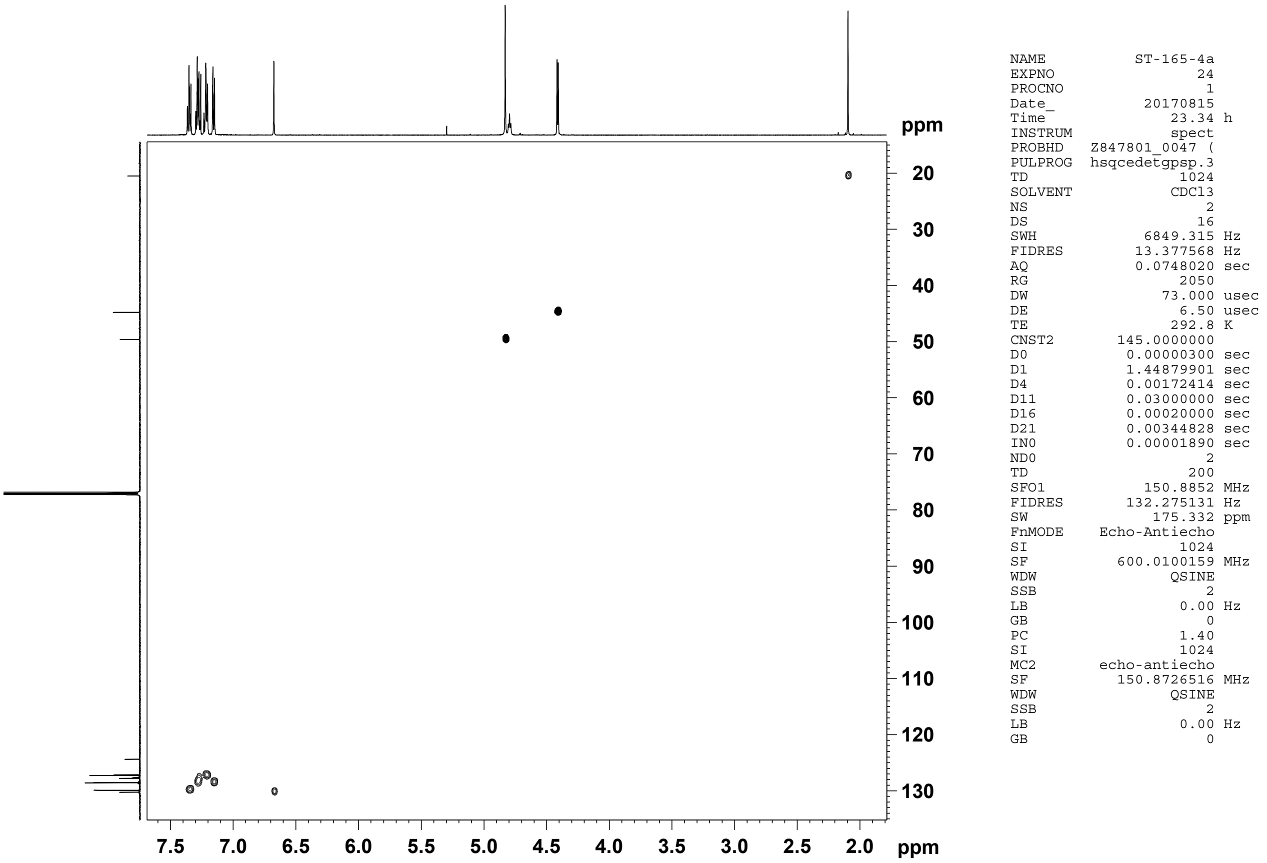

Figure S29. ^{1}H-^{13}C HSQC spectrum of compound **3ab**.

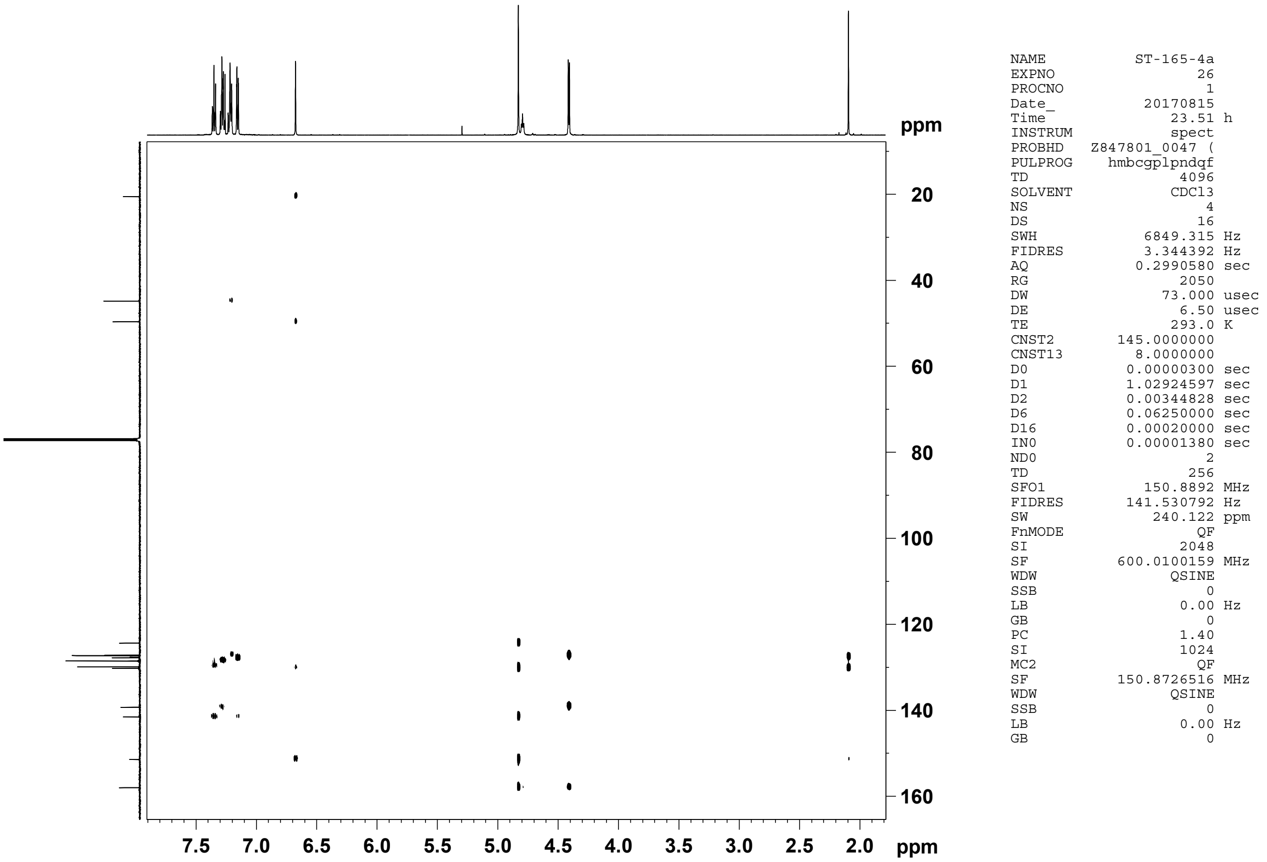

Figure S30. ^{1}H-^{13}C HMBC spectrum of compound **3ab**.

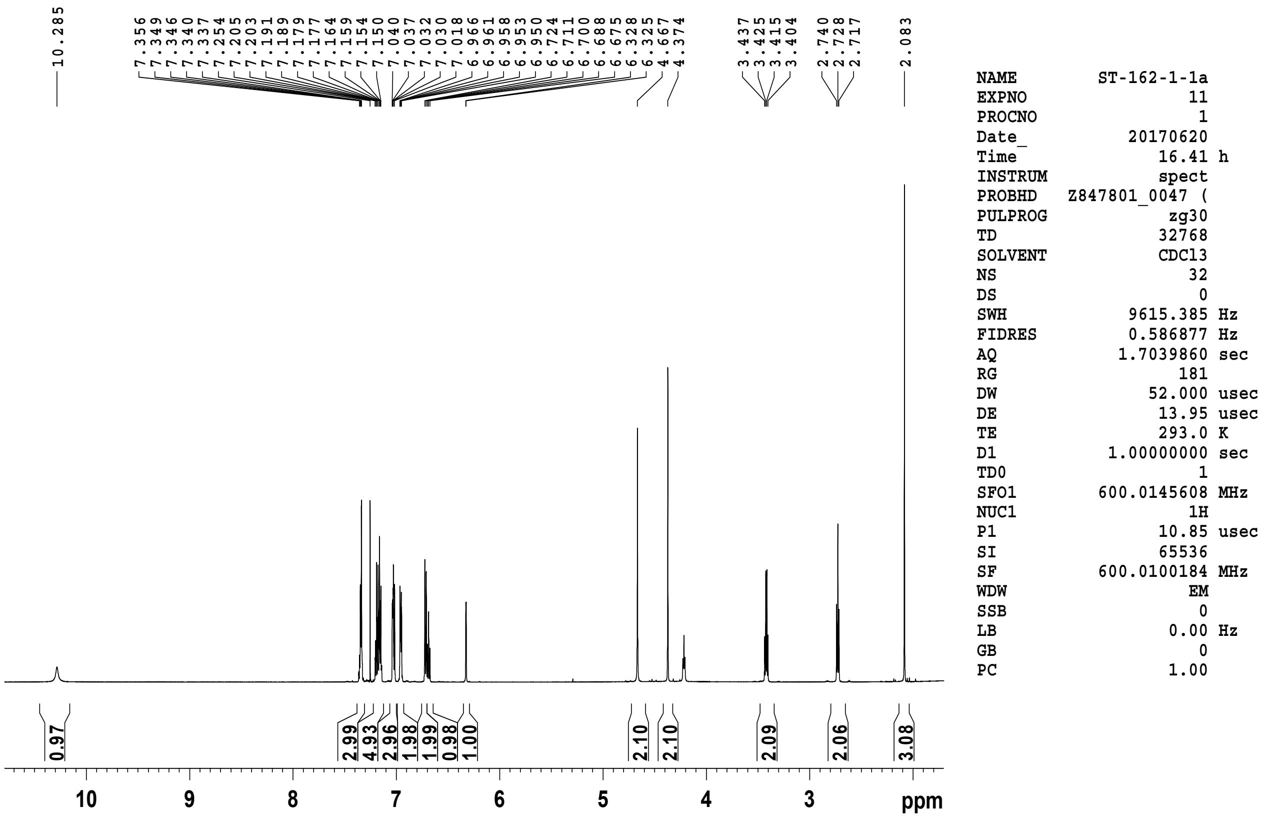

Figure S31. ^{1}H NMR spectrum of compound **2ac**.

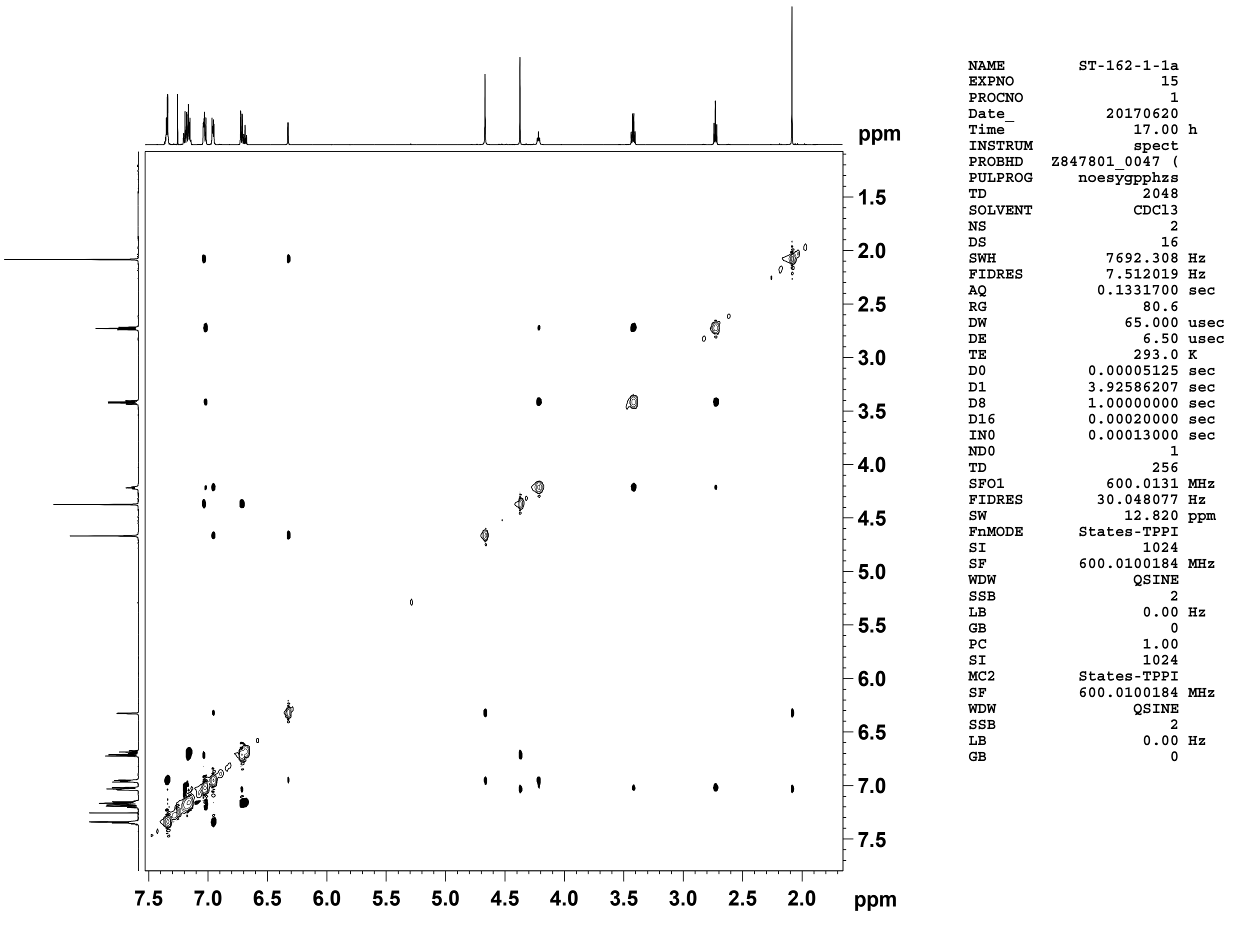

Figure S32. ^{1}H-^{1}H NOESY spectrum of compound **2ac**.

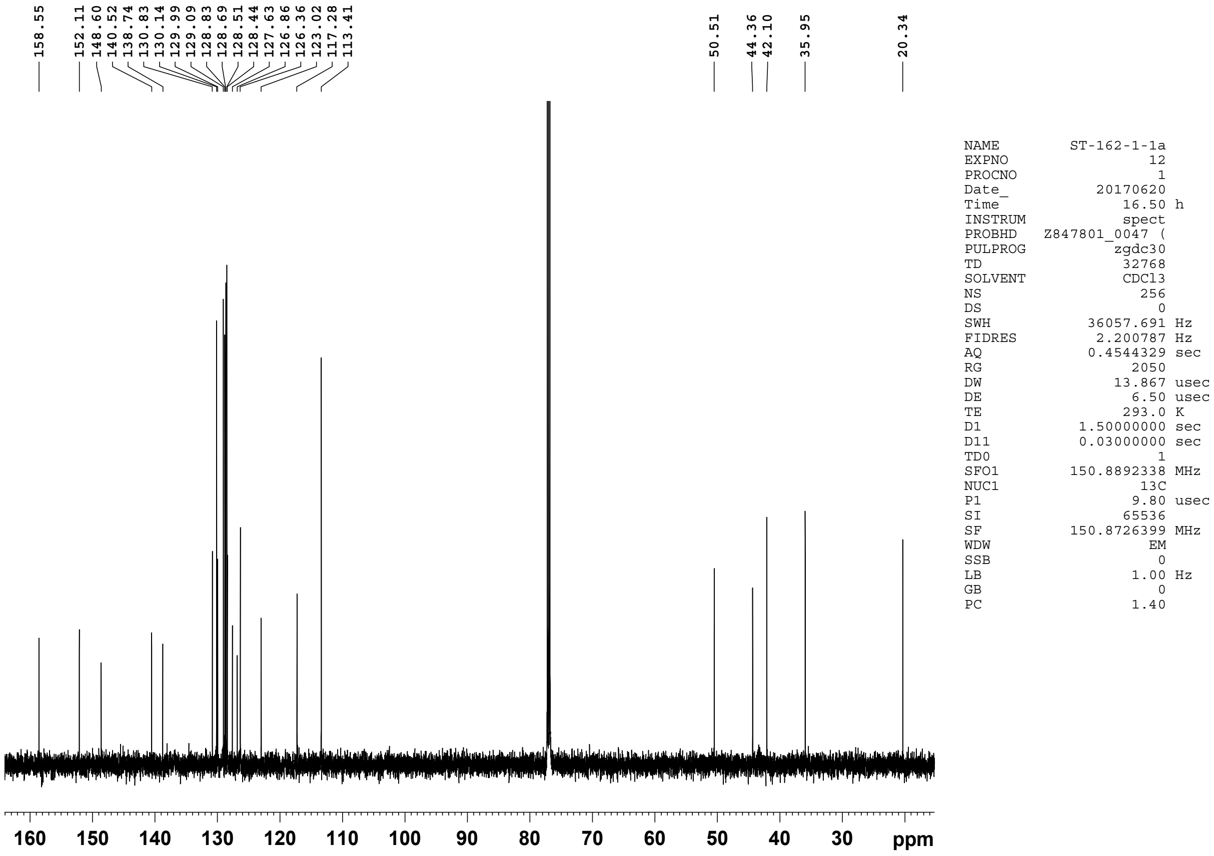

Figure S33. ^{13}C NMR spectrum of compound **2ac**.

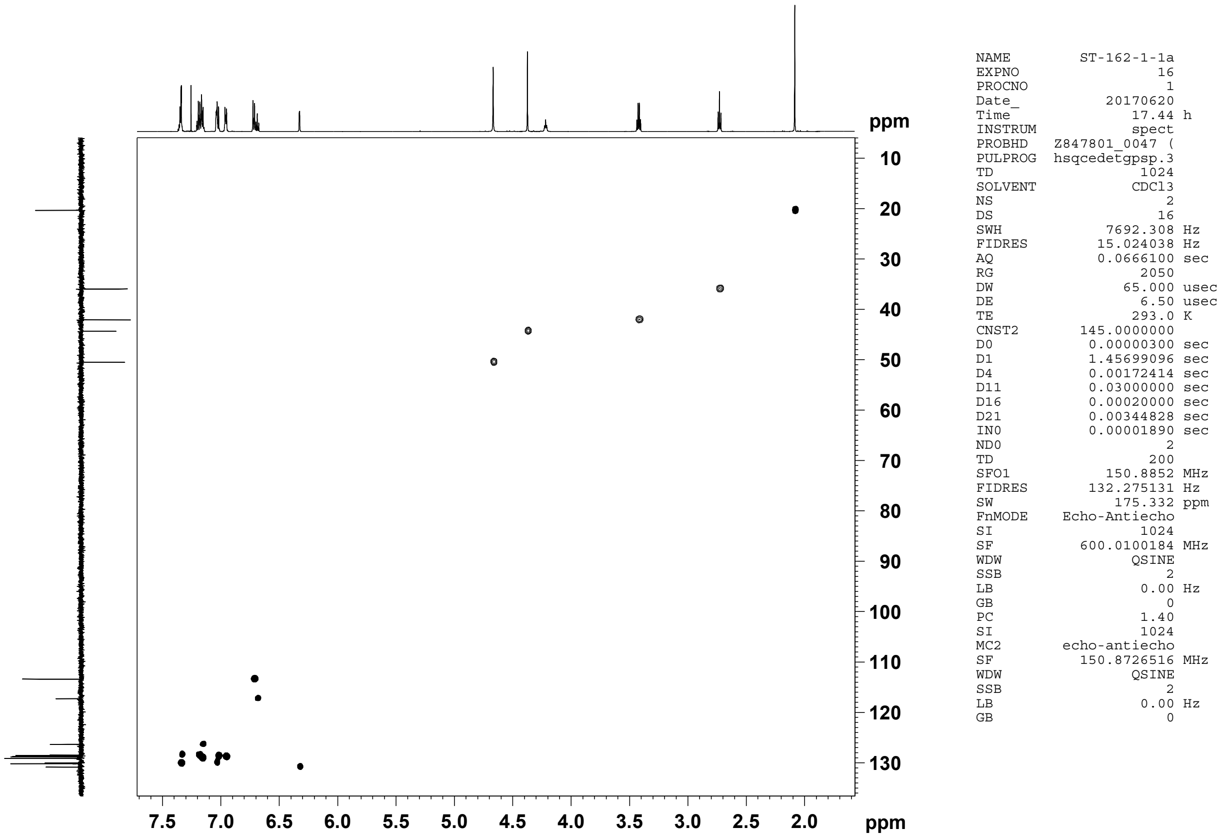

Figure S34. ^{1}H-^{13}C HSQC spectrum of compound **2ac**.

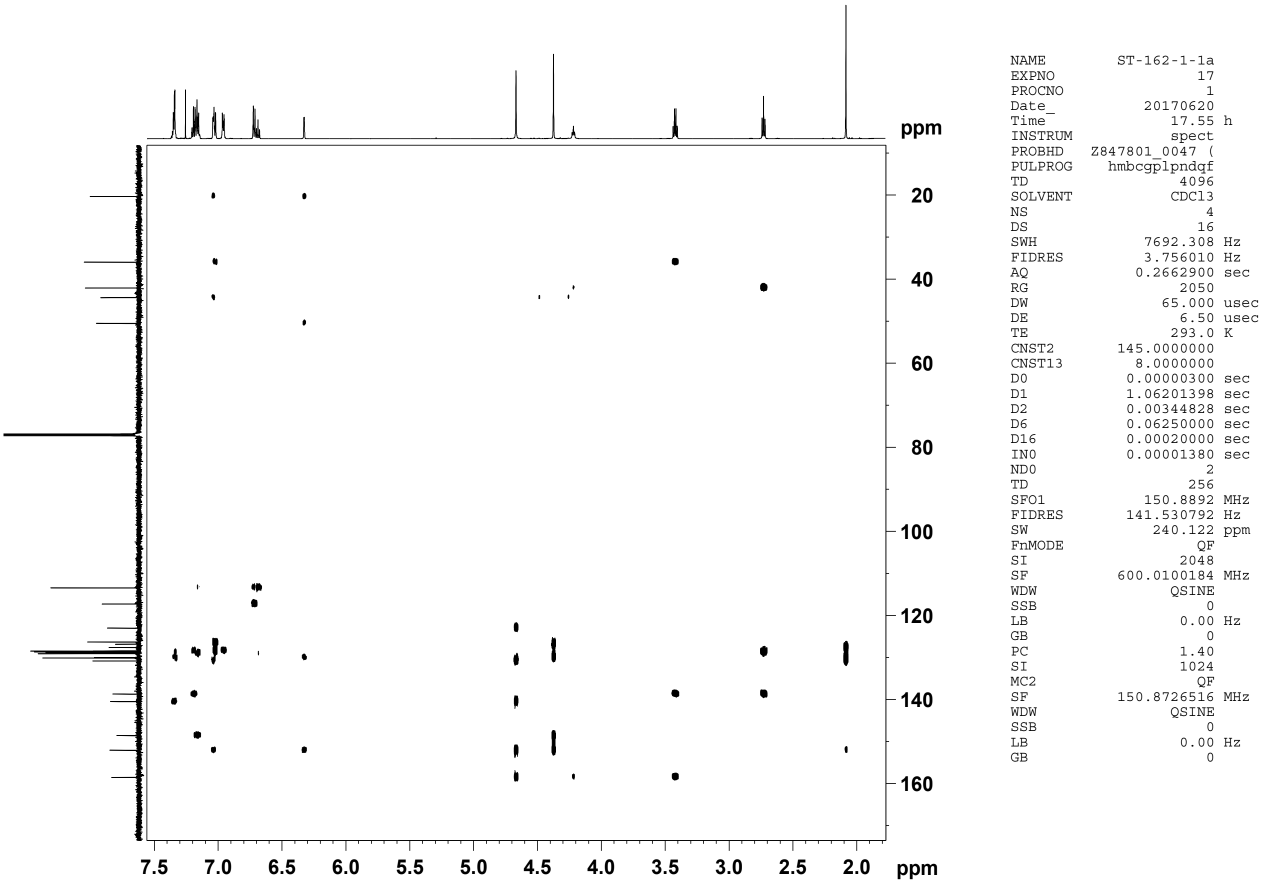

Figure S35. ^{1}H-^{13}C HMBC spectrum of compound **2ac**.

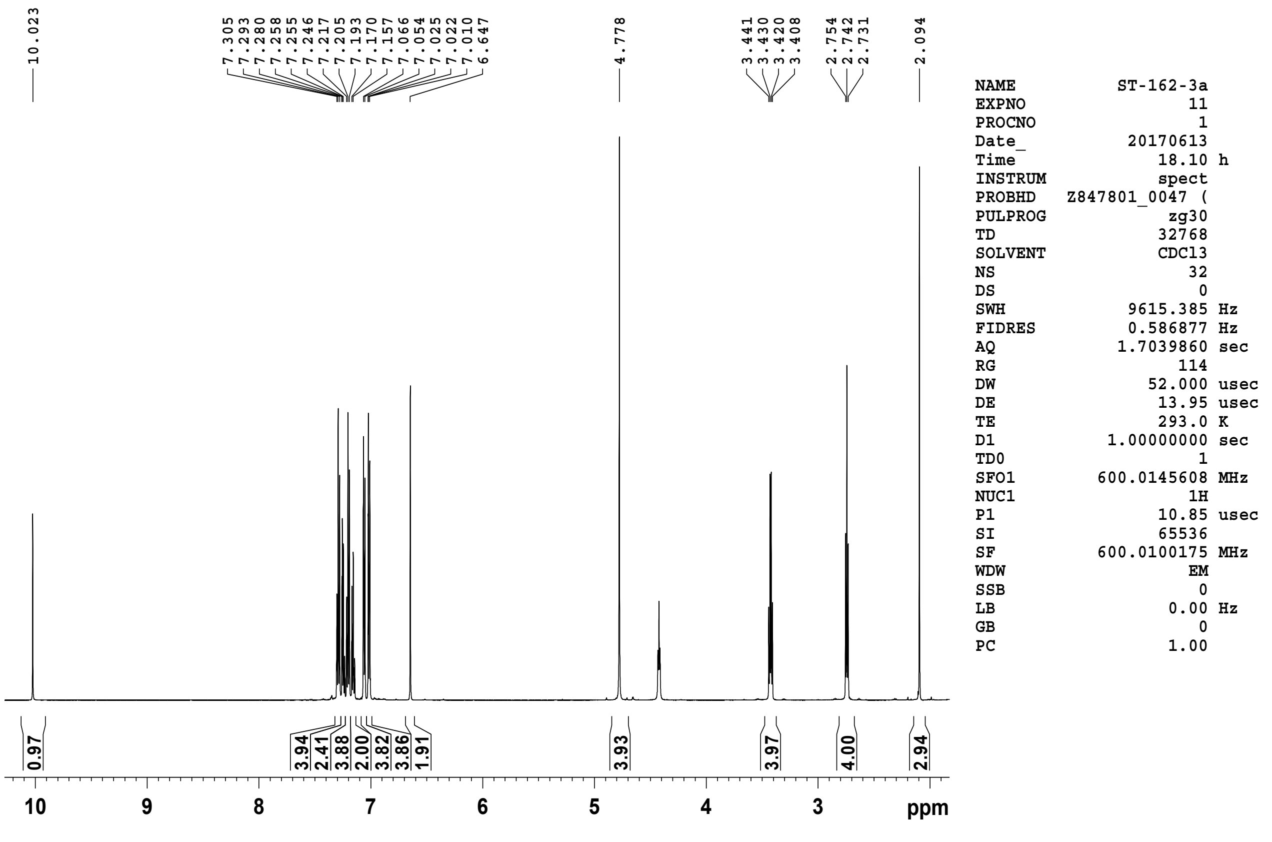

Figure S36. ^{1}H NMR spectrum of compound **3ac**.

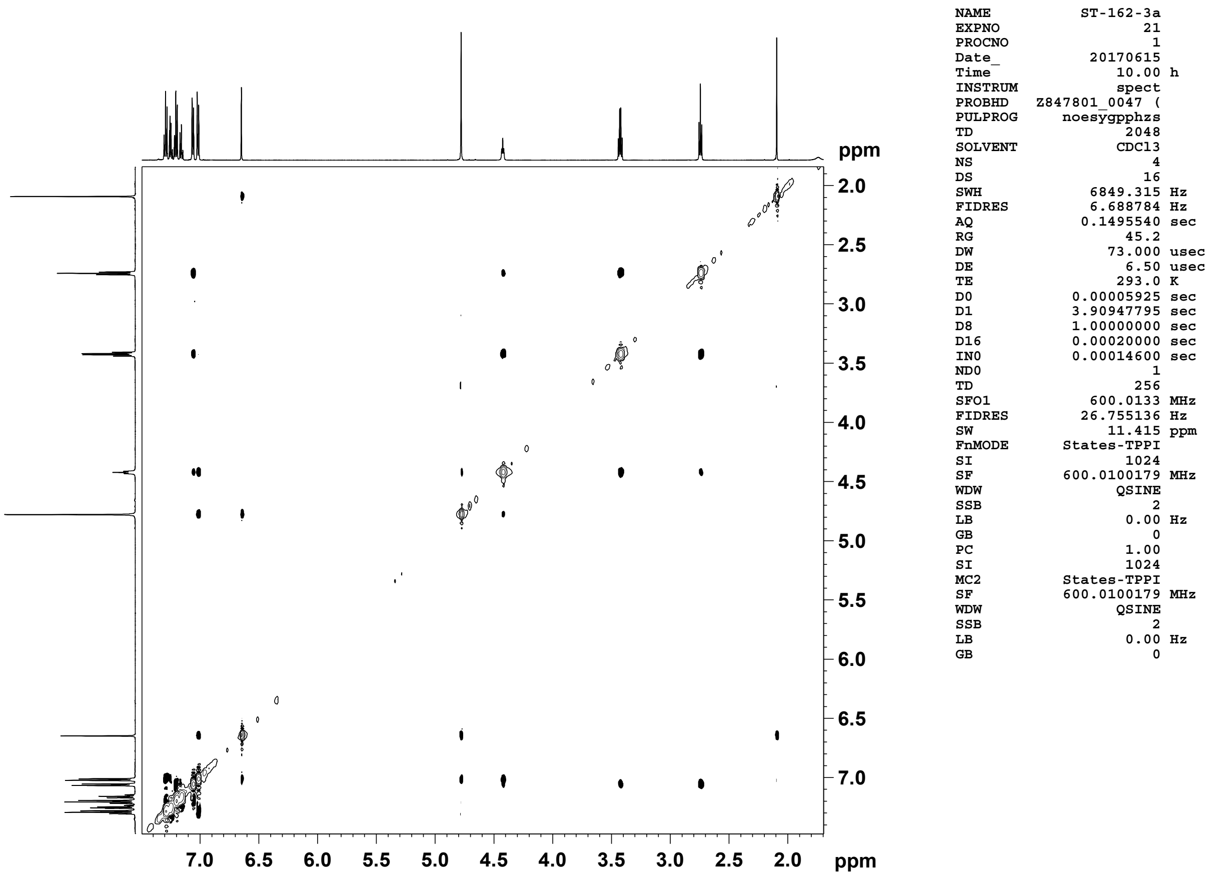

Figure S37. ^{1}H-^{1}H NOESY spectrum of compound **3ac**.

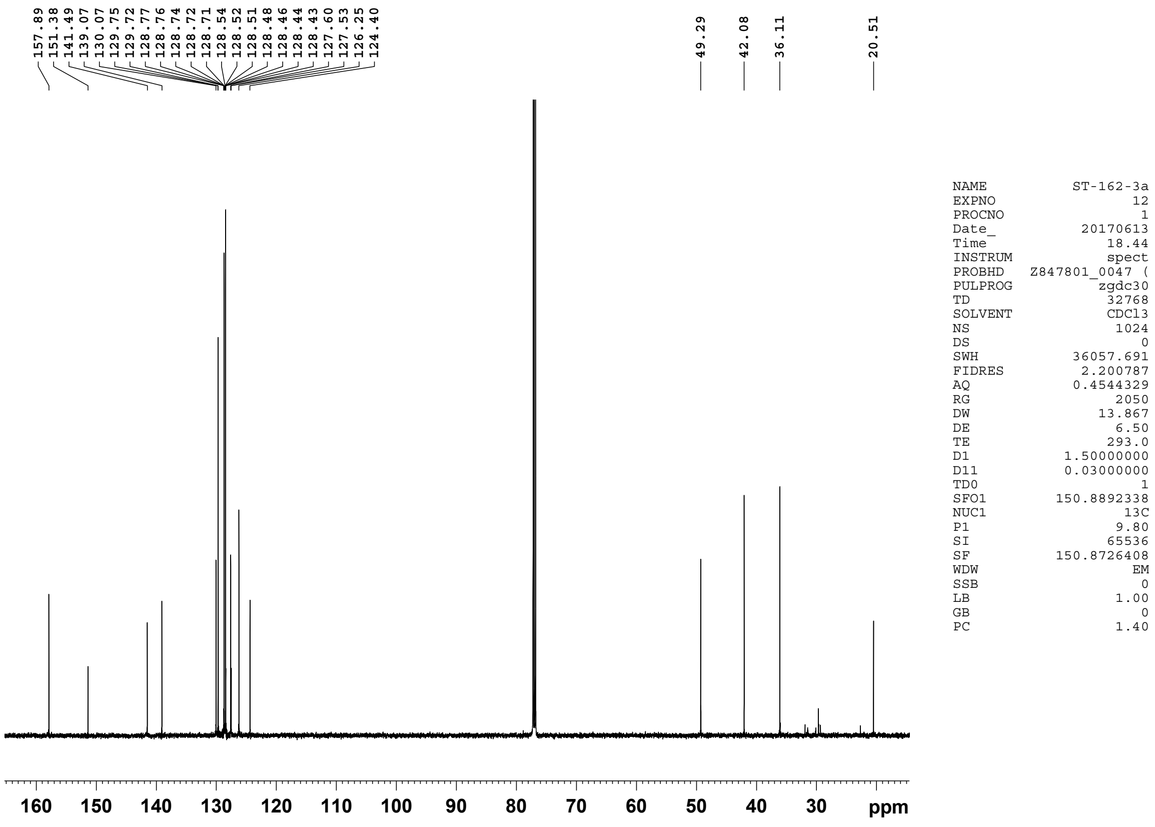

Figure S38. ^{13}C NMR spectrum of compound **3ac**.

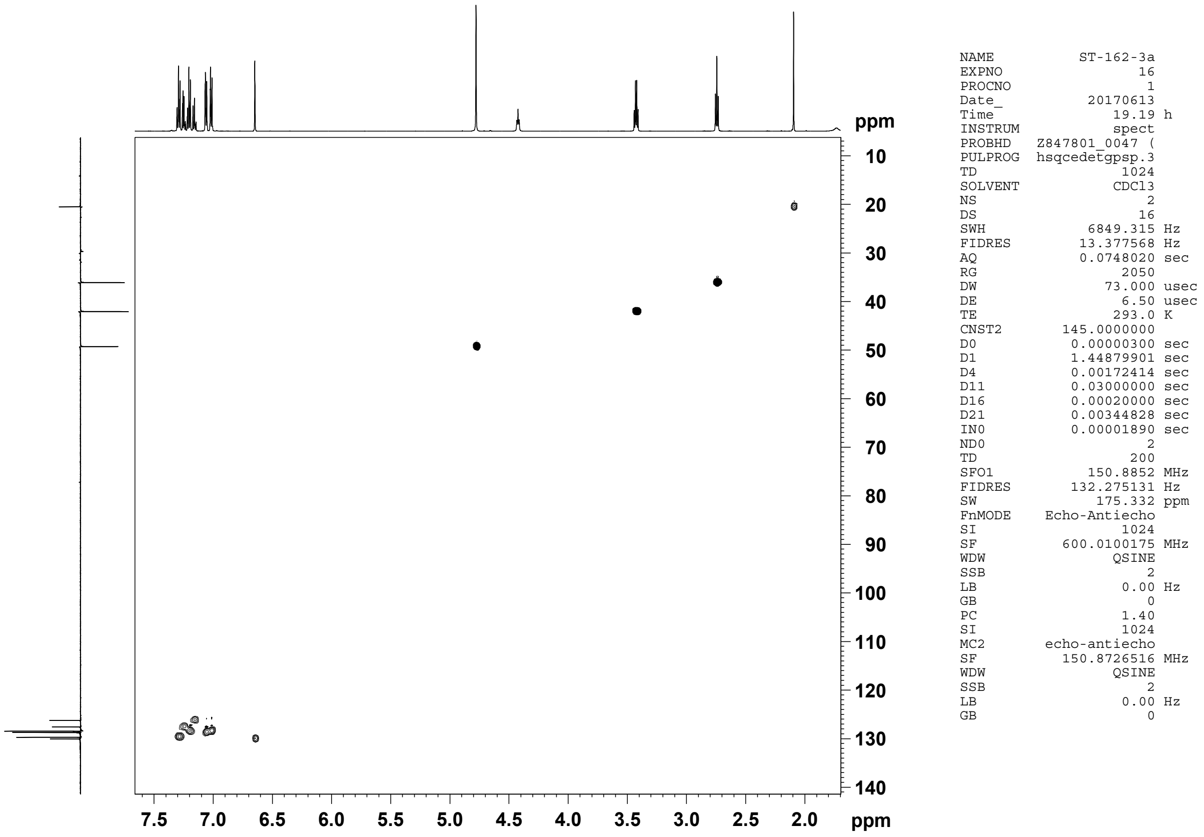

Figure S39. ^{1}H-^{13}C HSQC spectrum of compound **3ac**.

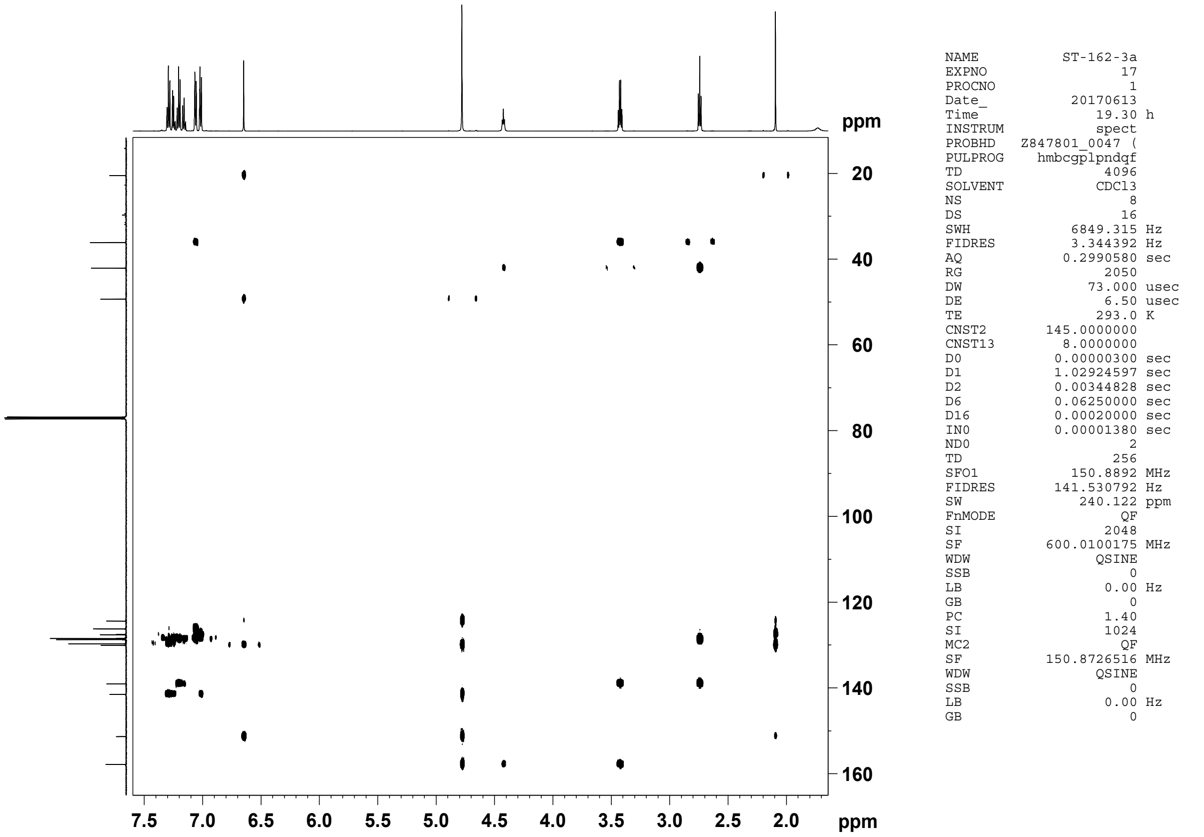

Figure S40. ^{1}H-^{13}C HMBC spectrum of compound **3ac**.

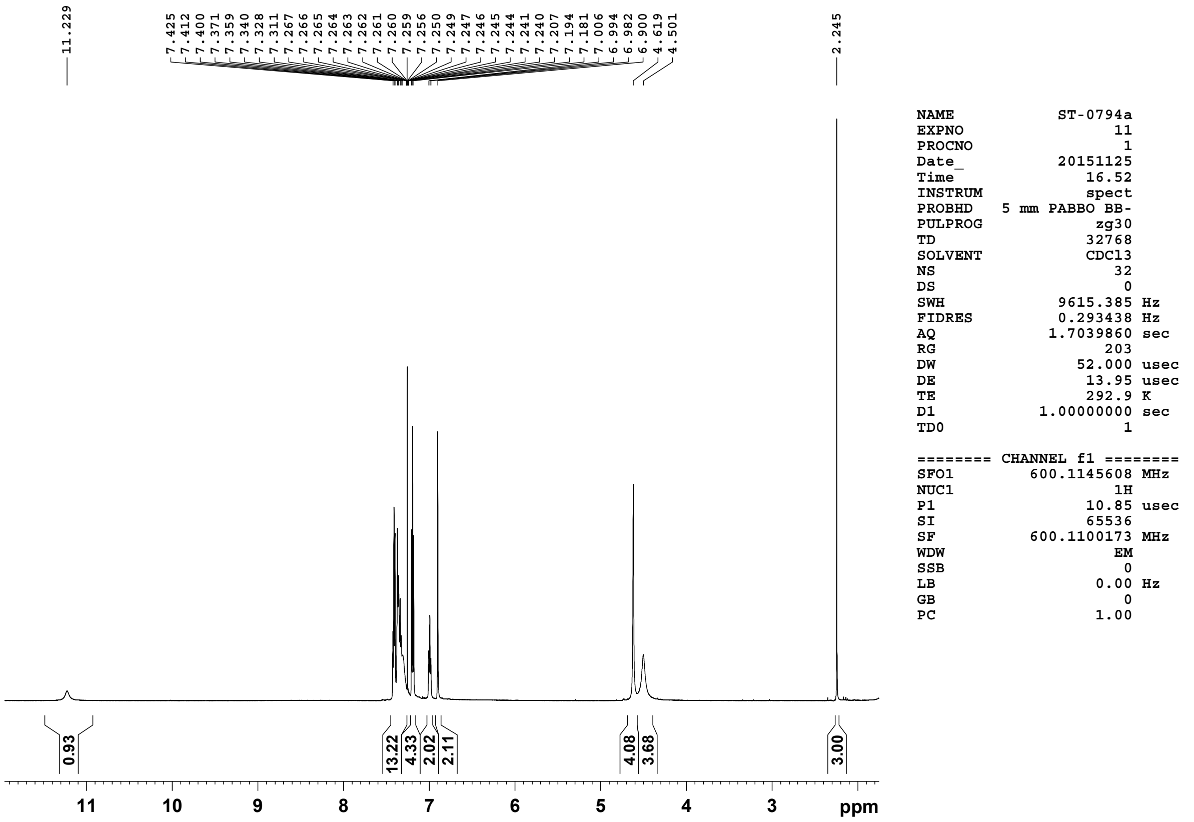

Figure S41. ^{1}H NMR spectrum of compound **3ba**.

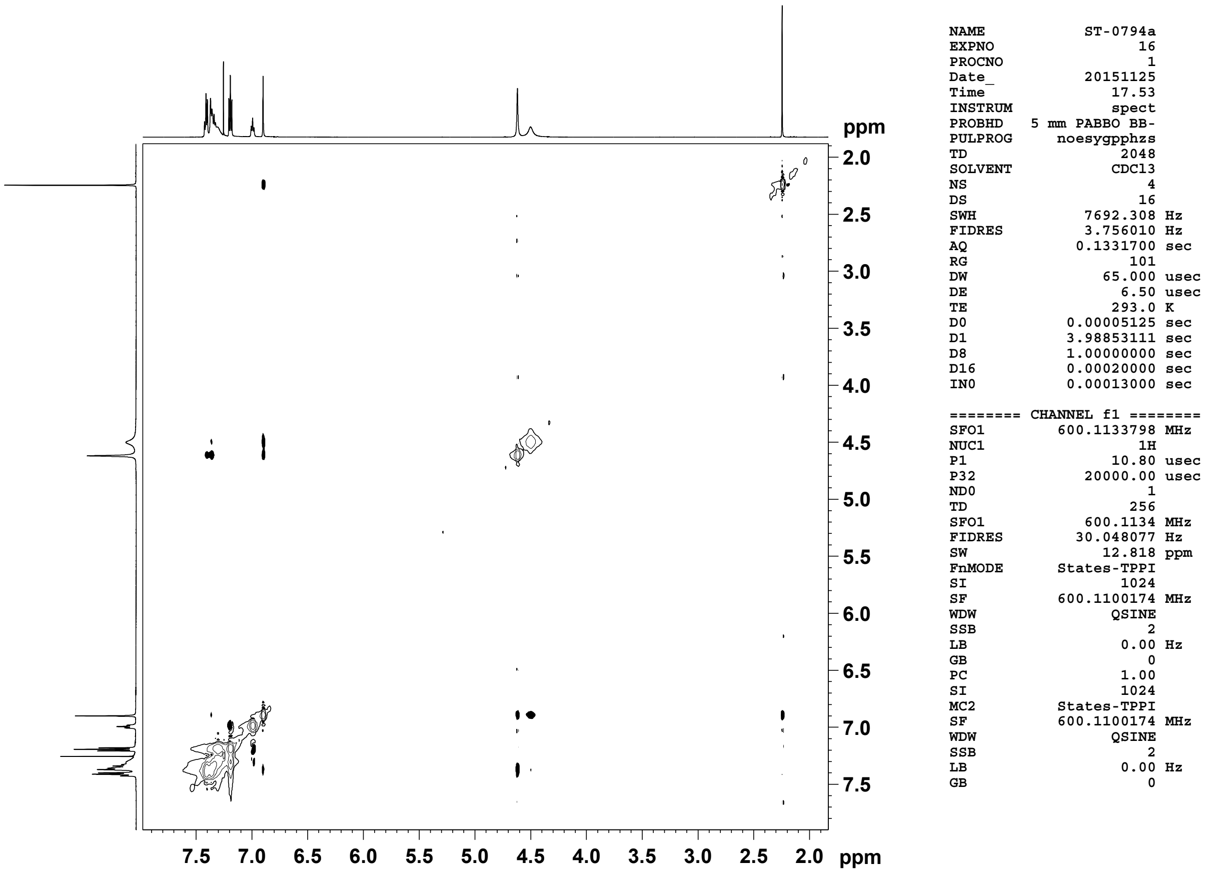

Figure S42. ^{1}H-^{1}H NOESY spectrum of compound **3ba**.

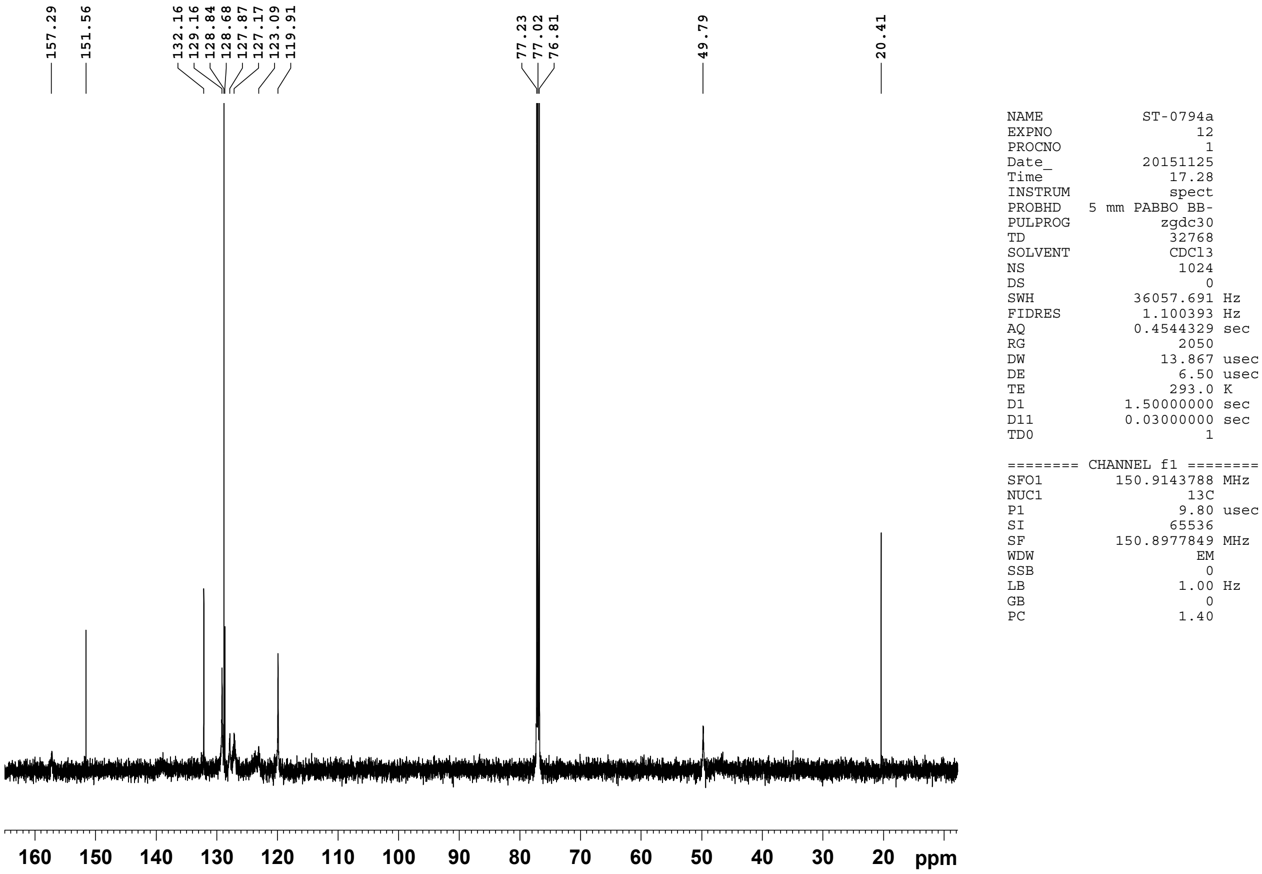

Figure S43. ^{13}C NMR spectrum of compound **3ba**.

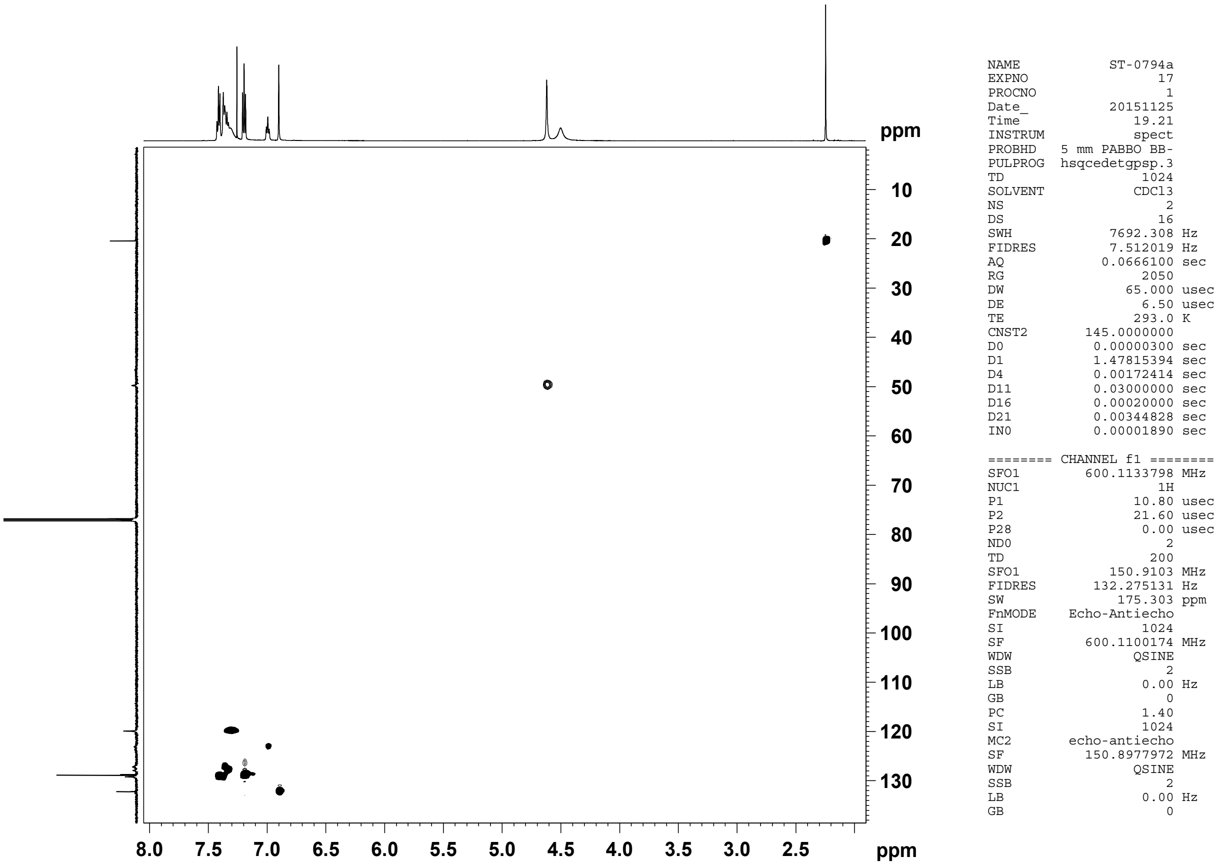

Figure S44. ^{1}H-^{13}C HSQC spectrum of compound **3ba**.

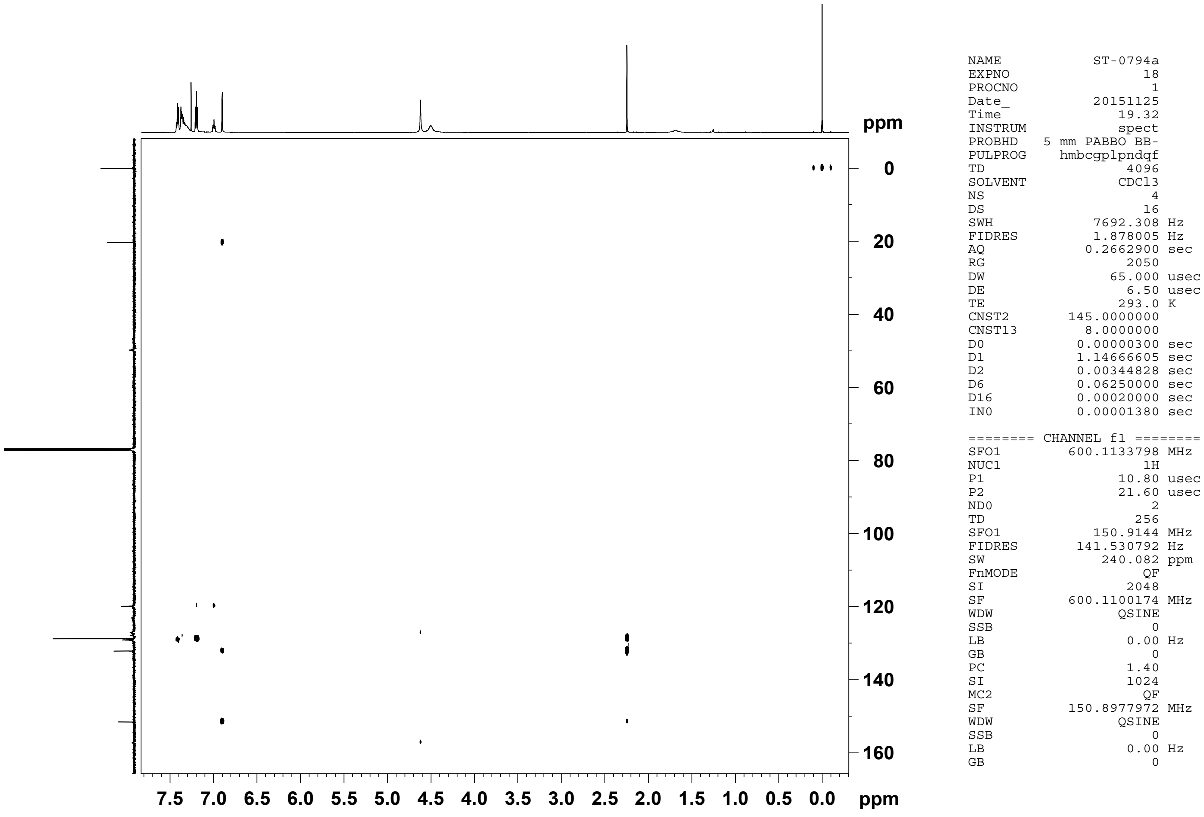

Figure S45. ^{1}H-^{13}C HMBC spectrum of compound **3ba**.

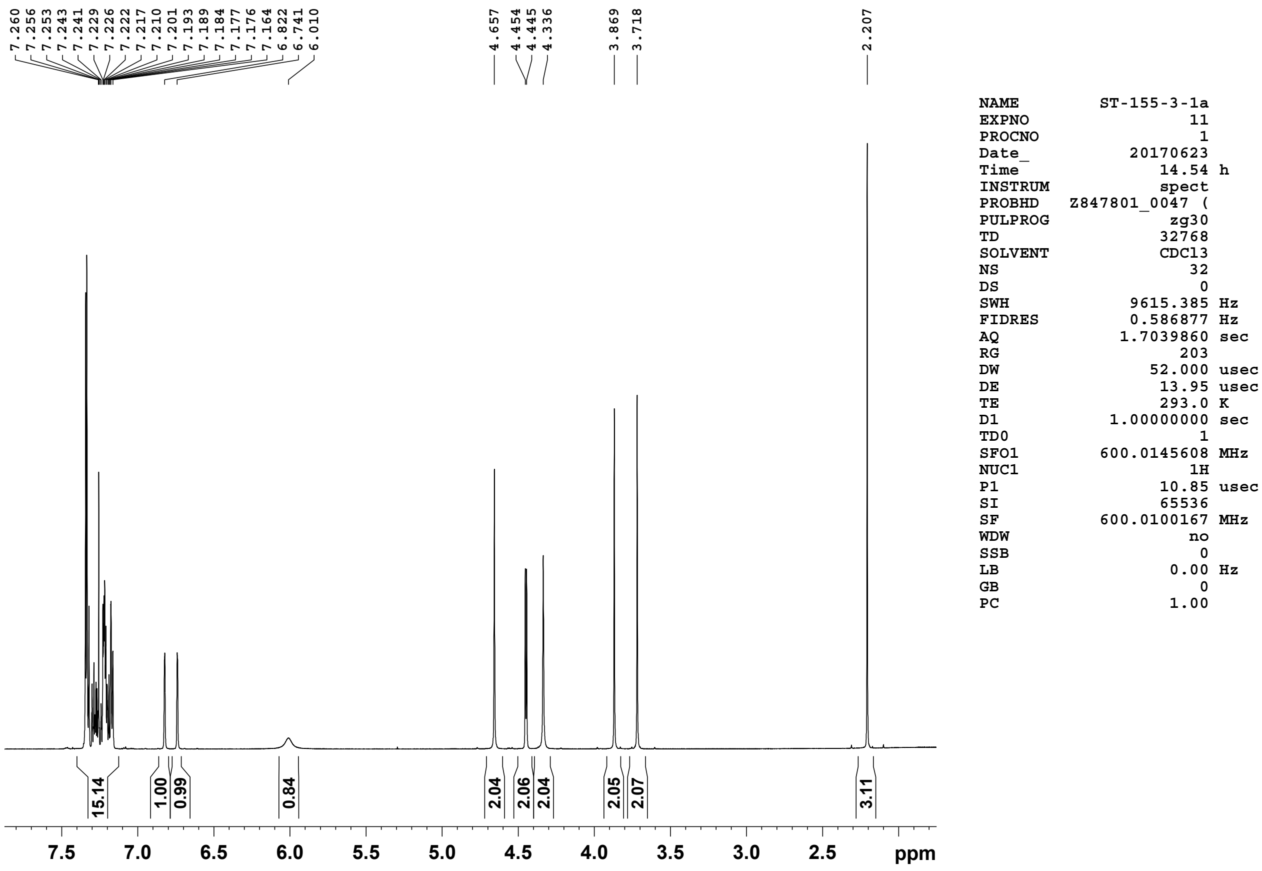

Figure S46. ^{1}H NMR spectrum of compound **2bb**.

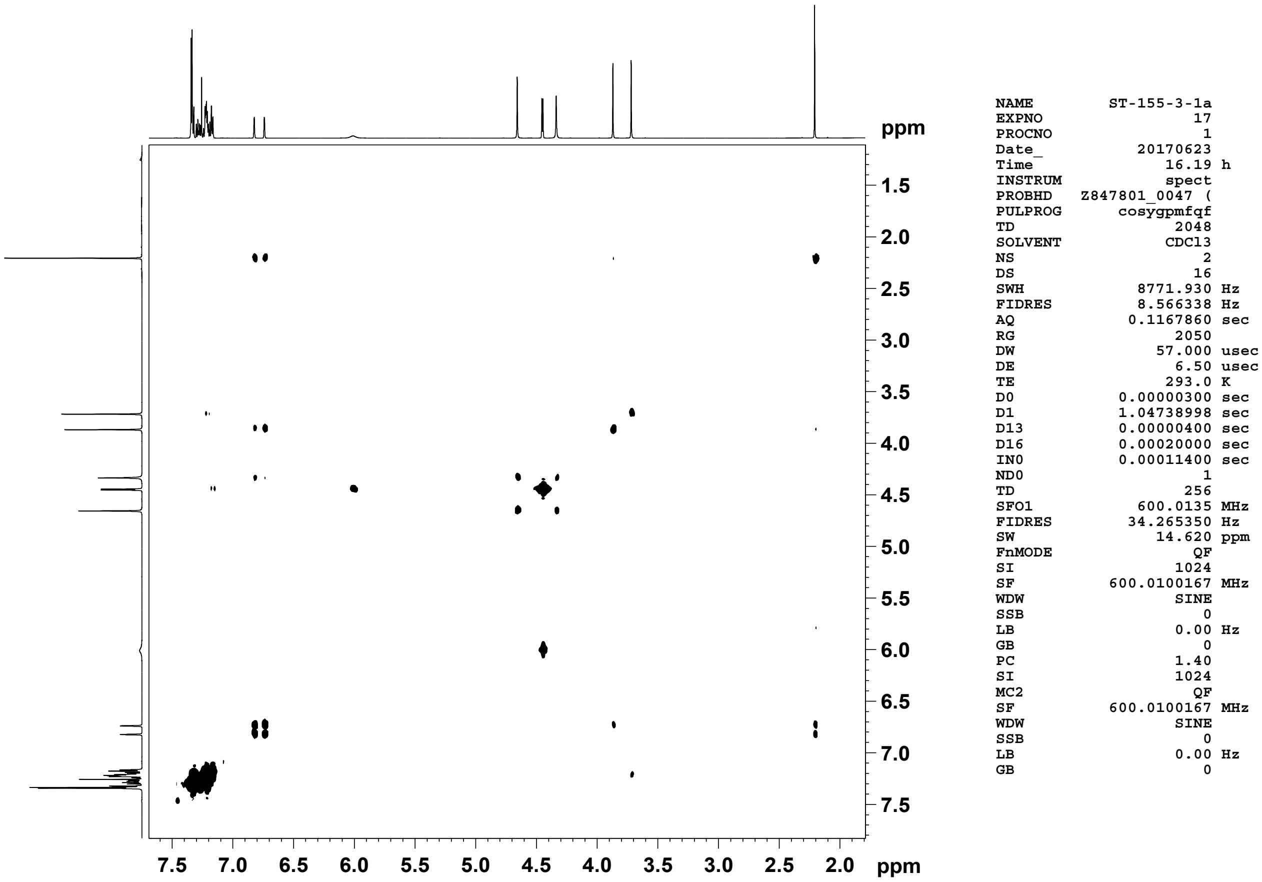

Figure S47. ^{1}H-^{1}H COSY spectrum of compound **2bb**.

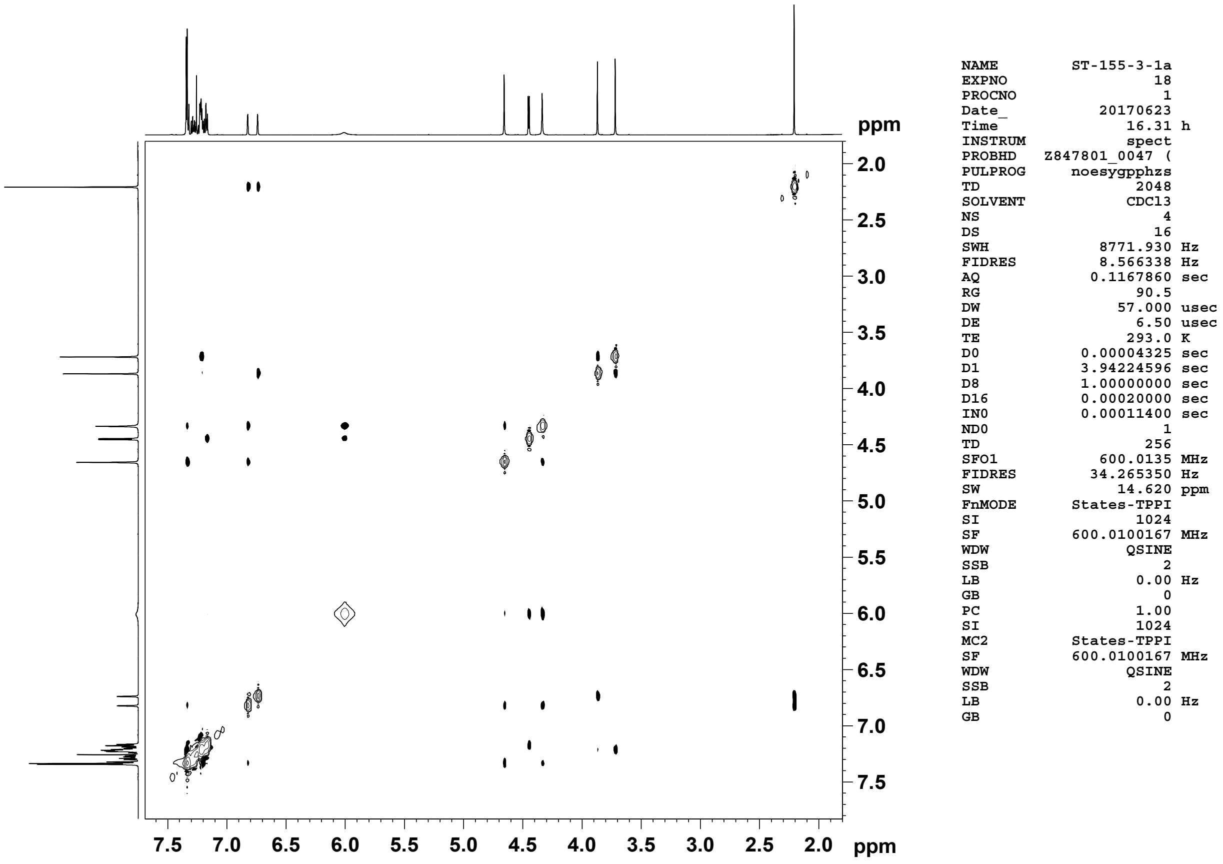

Figure S48. ^{1}H-^{1}H NOESY spectrum of compound **2bb**.

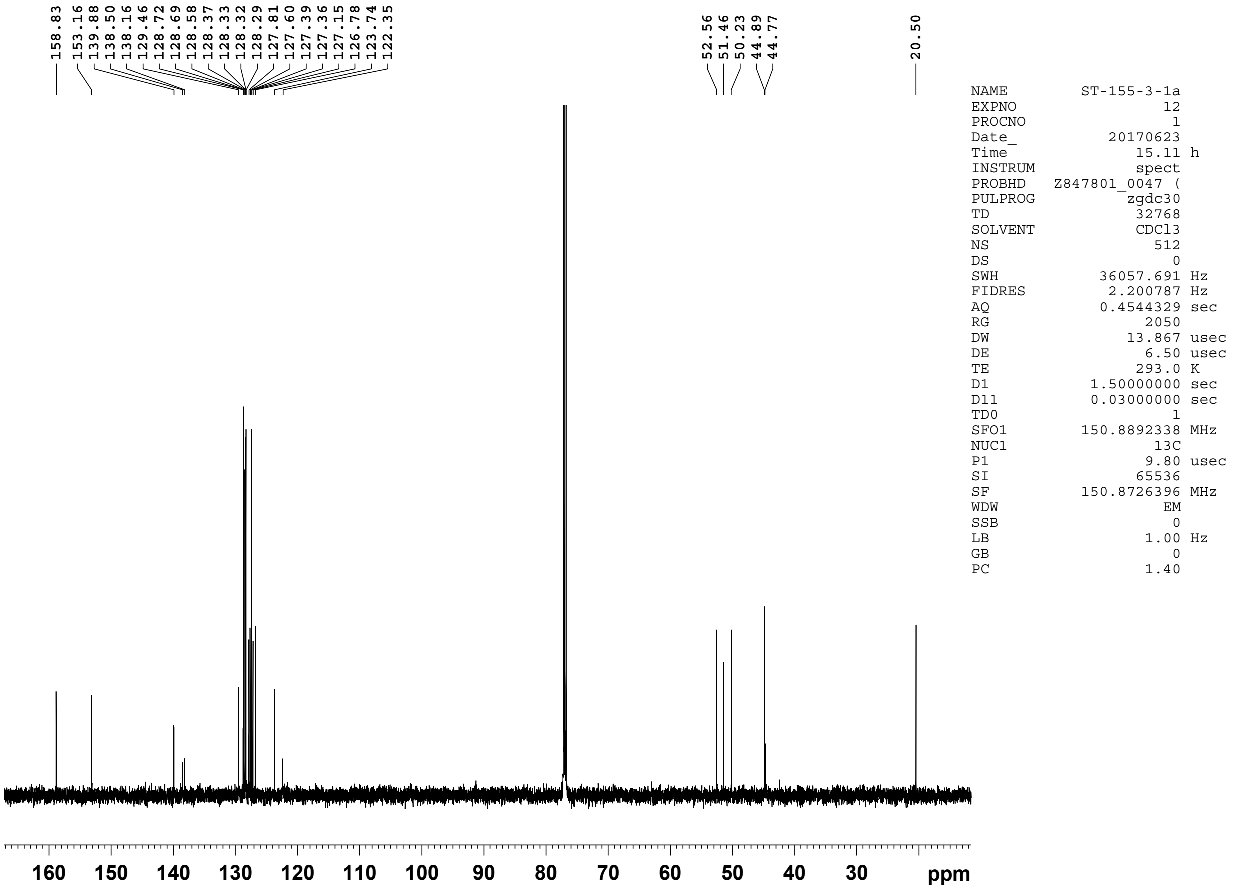

Figure S49. ^{13}C NMR spectrum of compound **2bb**.

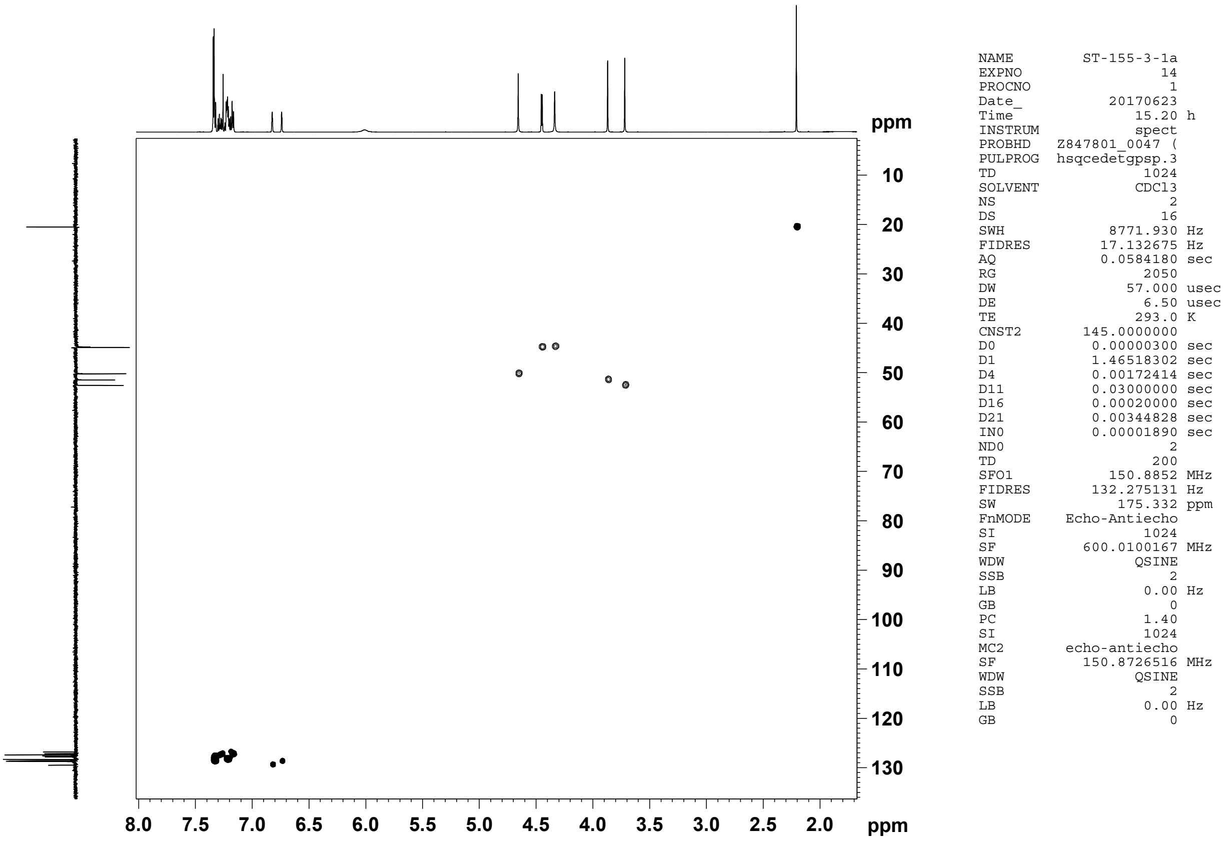

Figure S50. ^{1}H-^{13}C HSQC spectrum of compound **2bb**.

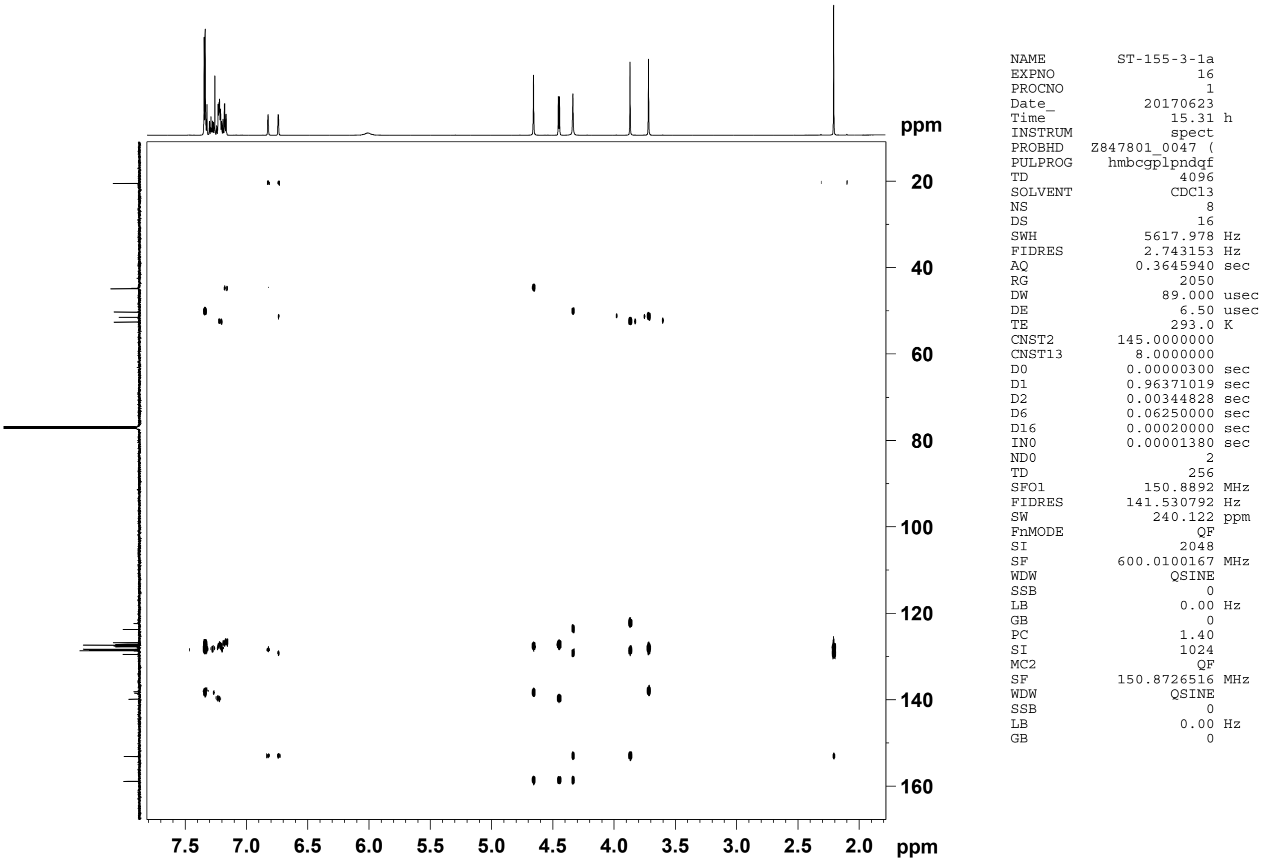

Figure S51. ^{1}H-^{13}C HMBC spectrum of compound **2bb**.

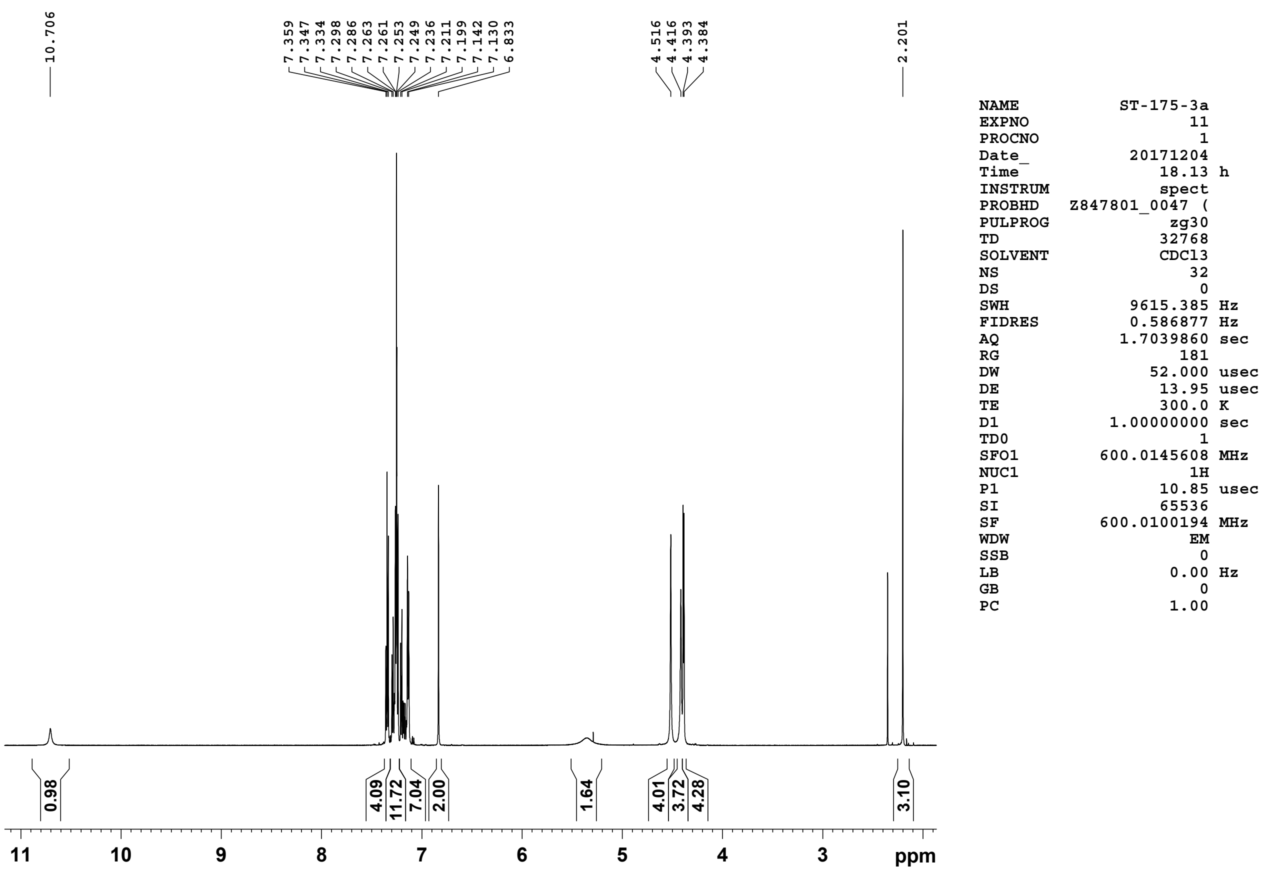

Figure S52. ^{1}H NMR spectrum of compound **3bb**.

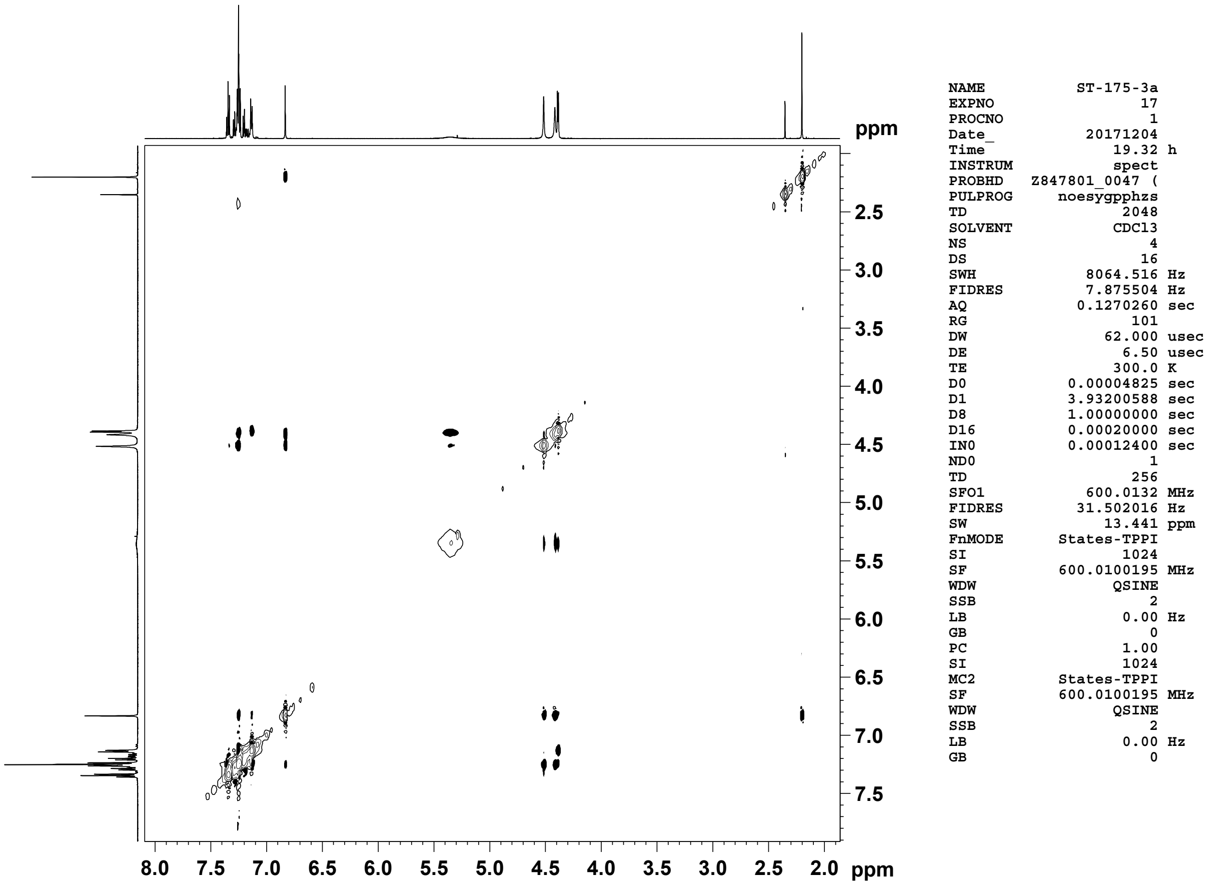

Figure S53. ^{1}H-^{1}H NOESY spectrum of compound **3bb**.

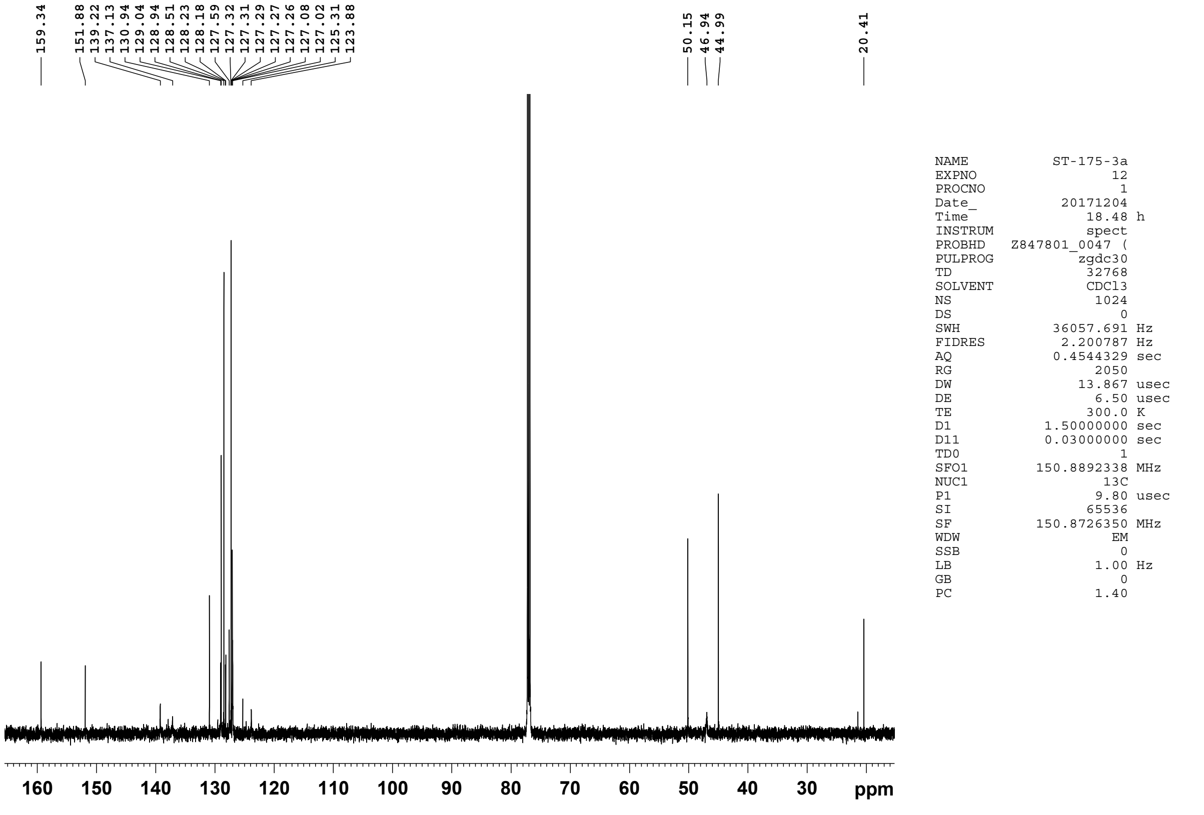

Figure S54. ^{13}C NMR spectrum of compound **3bb**.

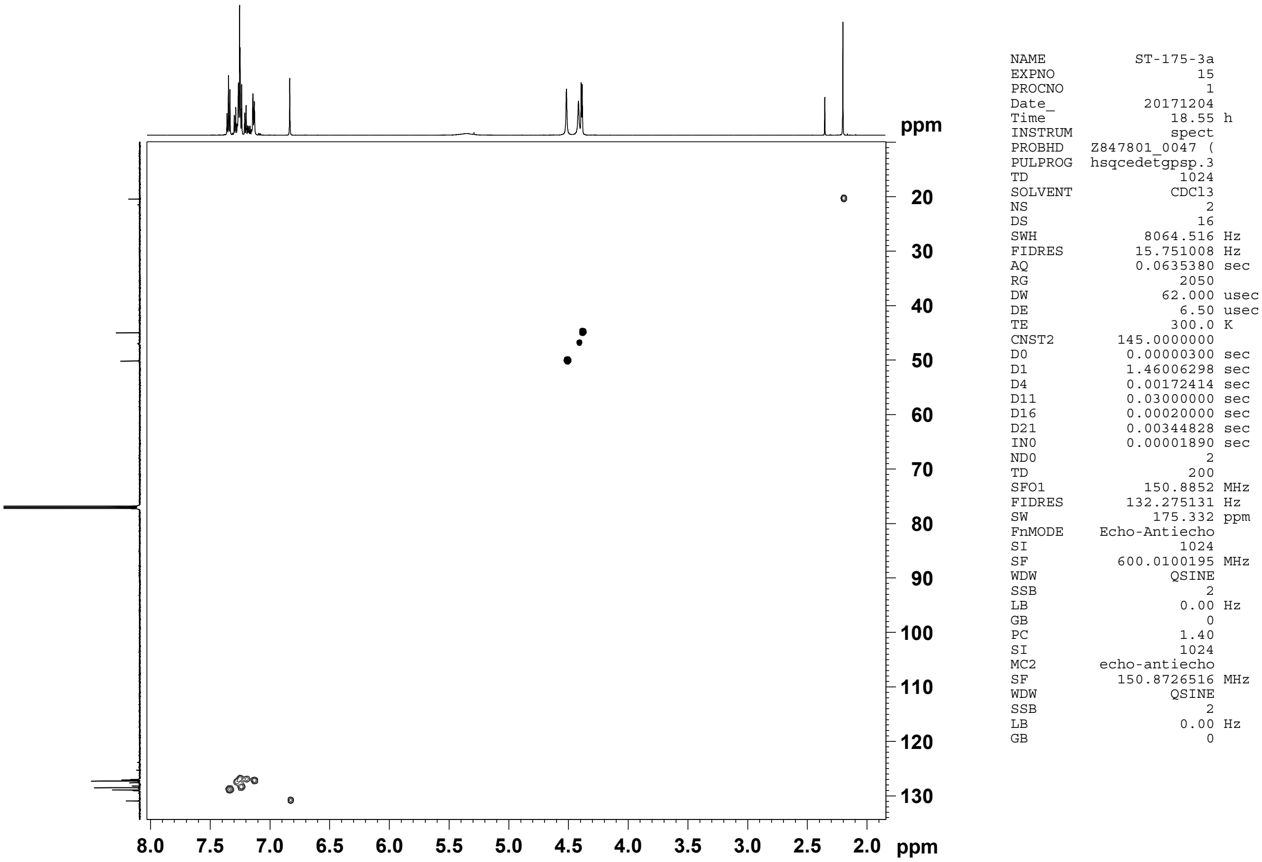

Figure S55. ^{1}H-^{13}C HSQC spectrum of compound **3bb**.

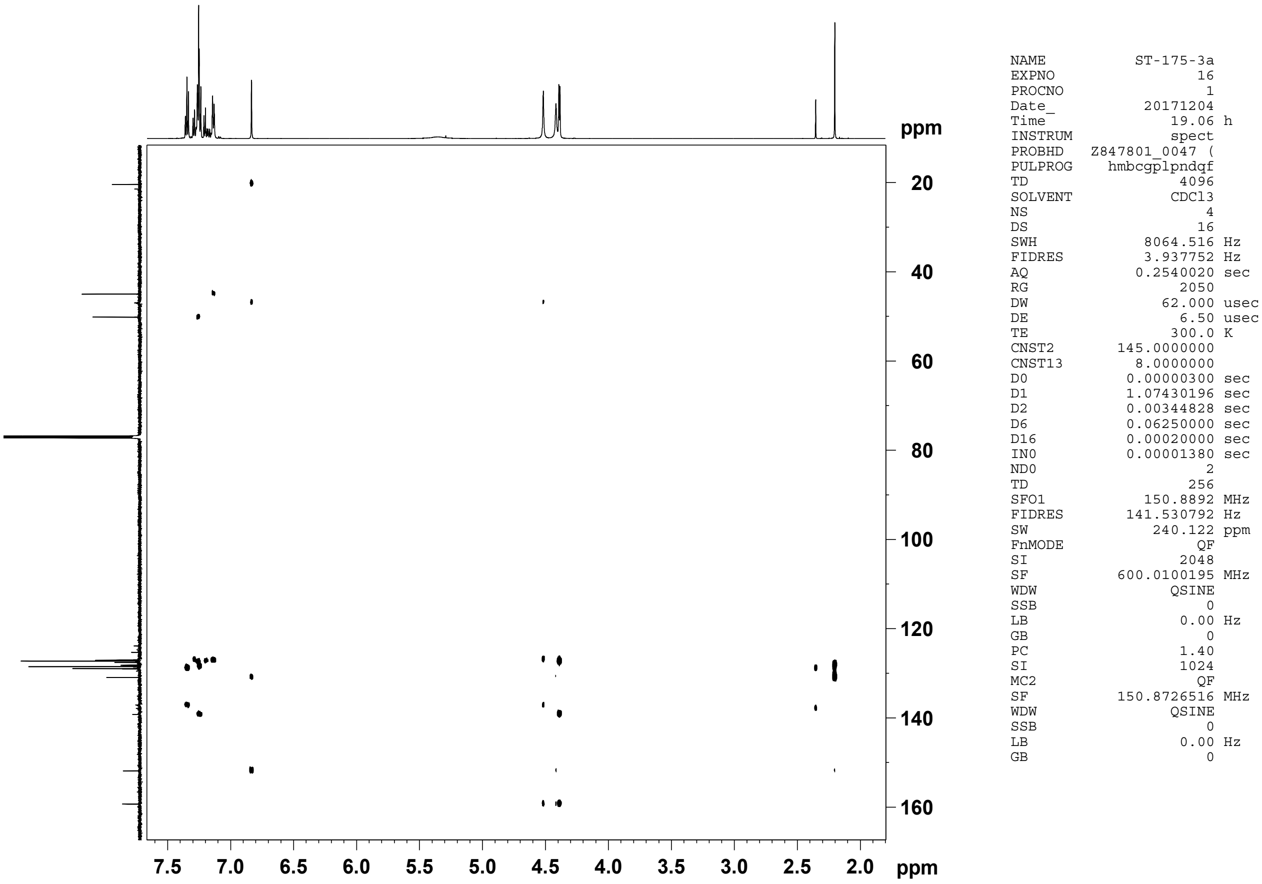

Figure S56. ^{1}H-^{13}C HMBC spectrum of compound **3bb**.

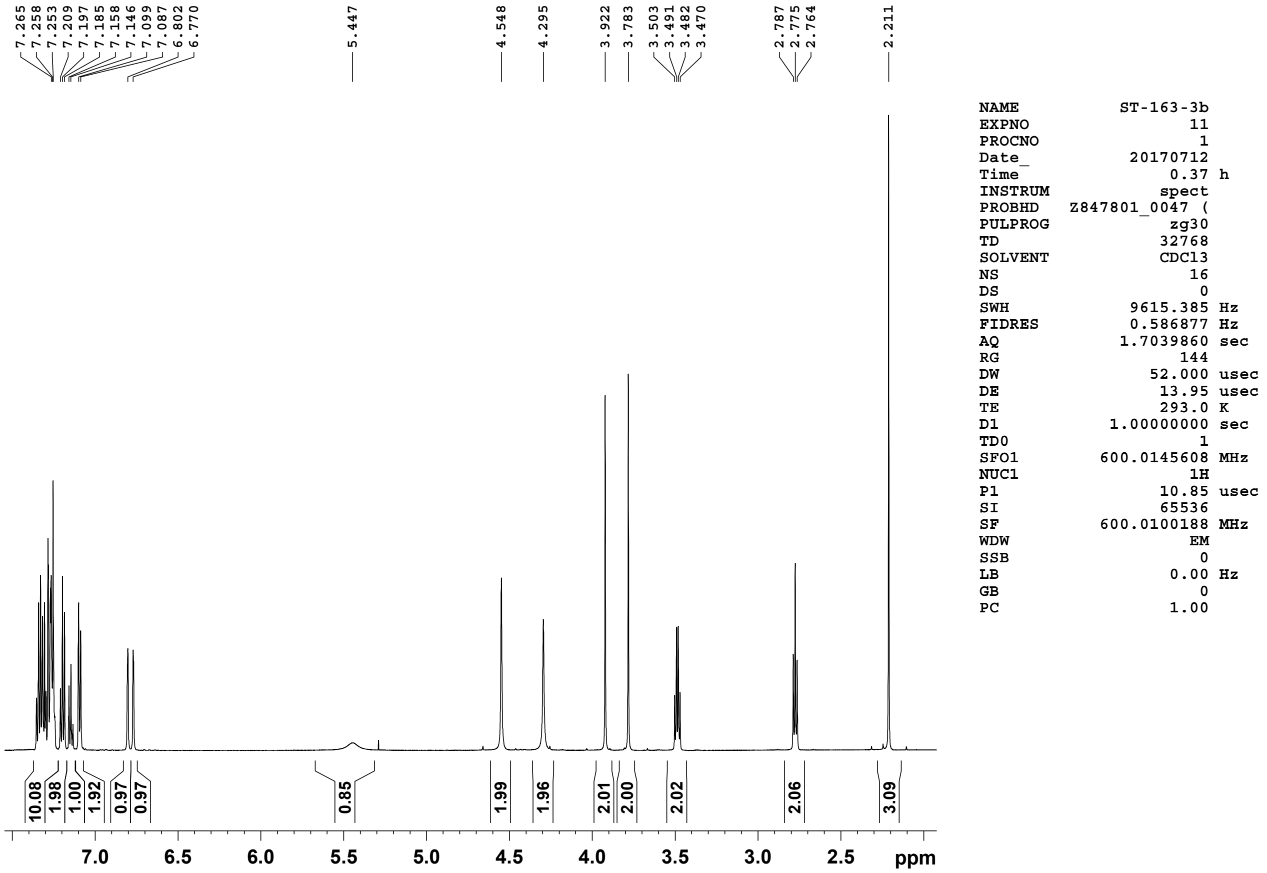

Figure S57. ^{1}H NMR spectrum of compound **2bc**.

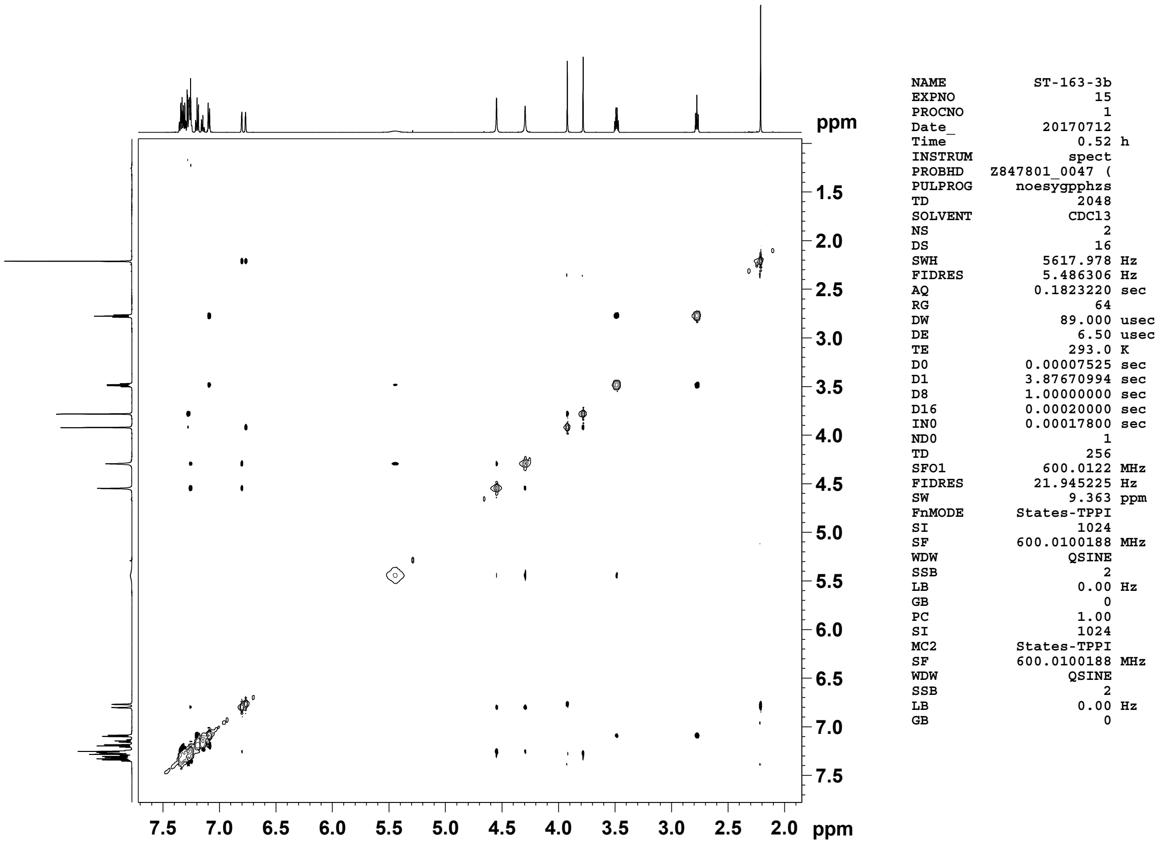

Figure S58. ^{1}H-^{1}H NOESY spectrum of compound **2bc**.

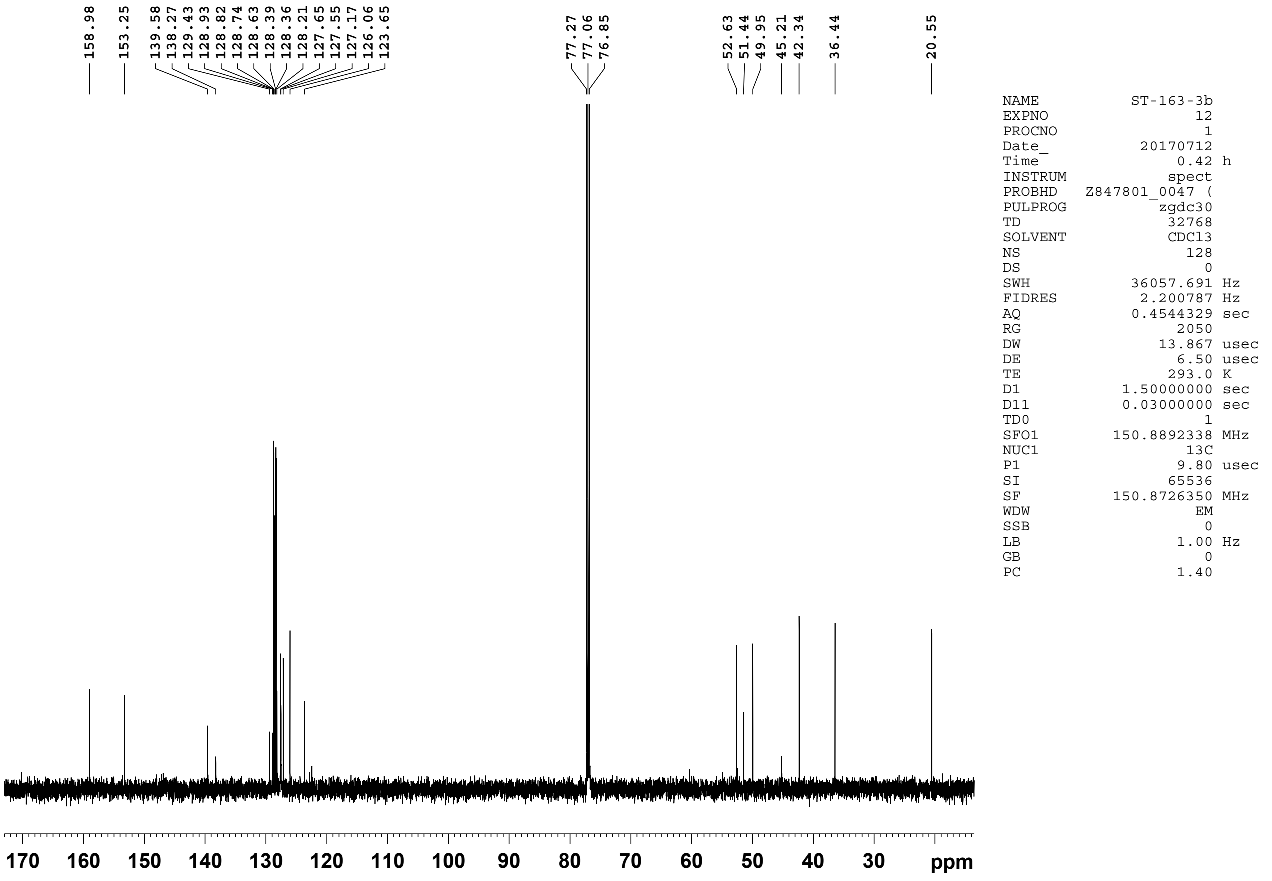

Figure S59. ^{13}C NMR spectrum of compound **2bc**.

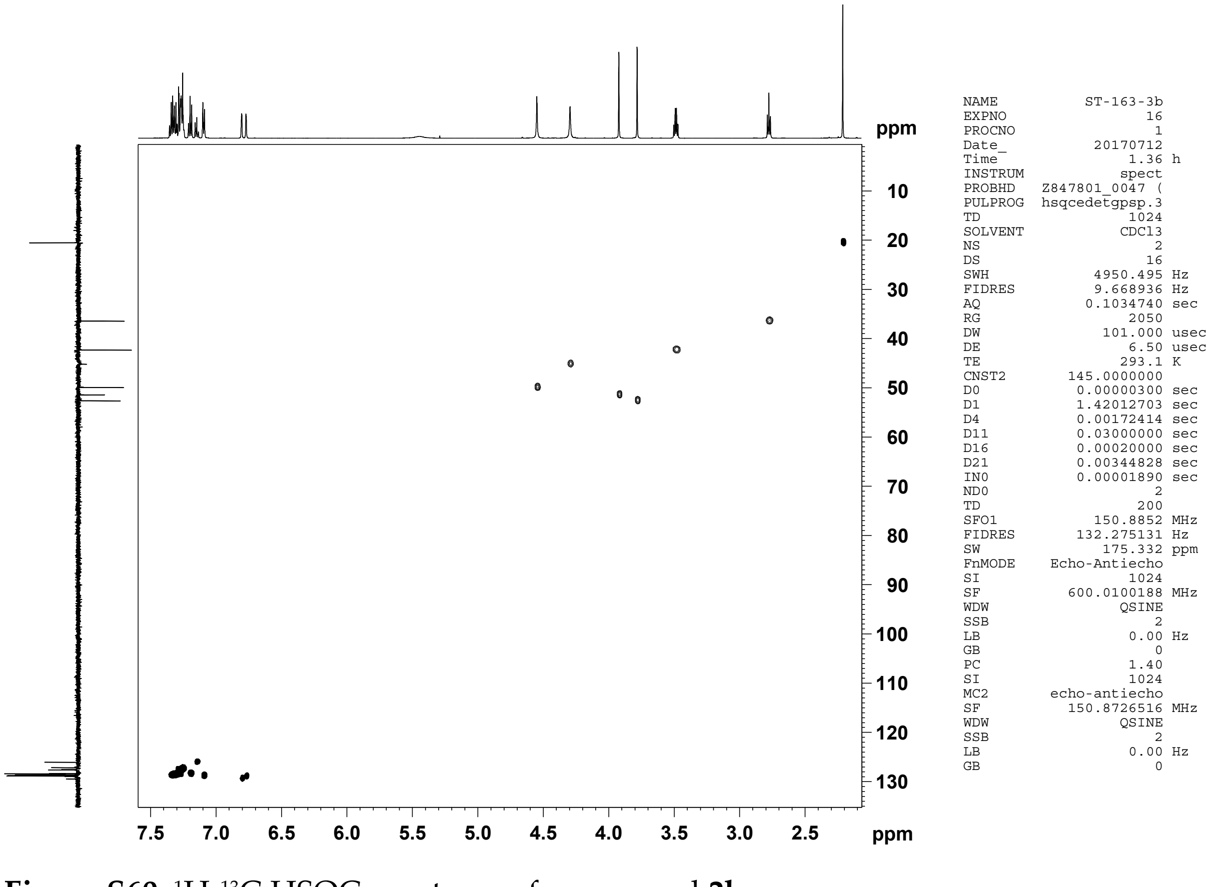

Figure S60. ^{1}H-^{13}C HSQC spectrum of compound **2bc**.

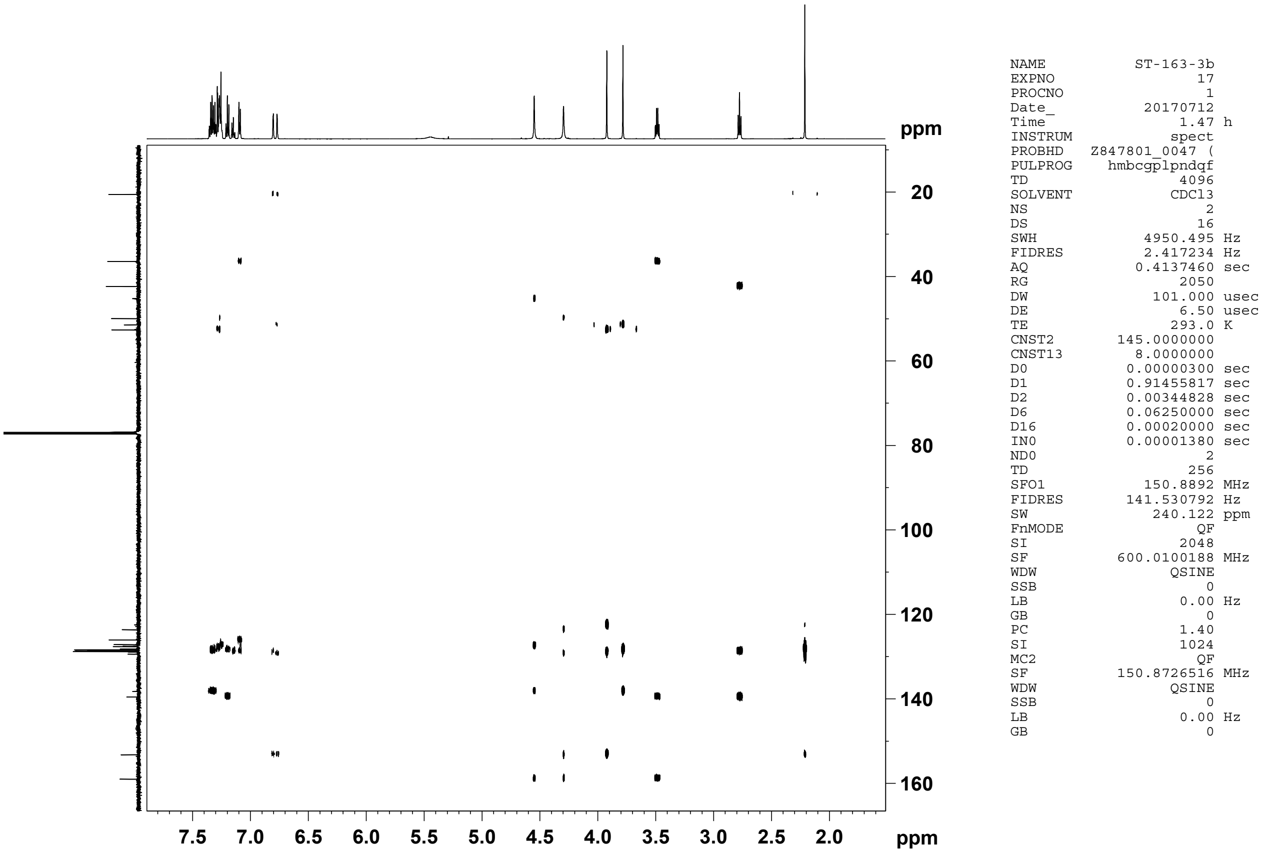

Figure S61. ^{1}H-^{13}C HMBC spectrum of compound **2bc**.

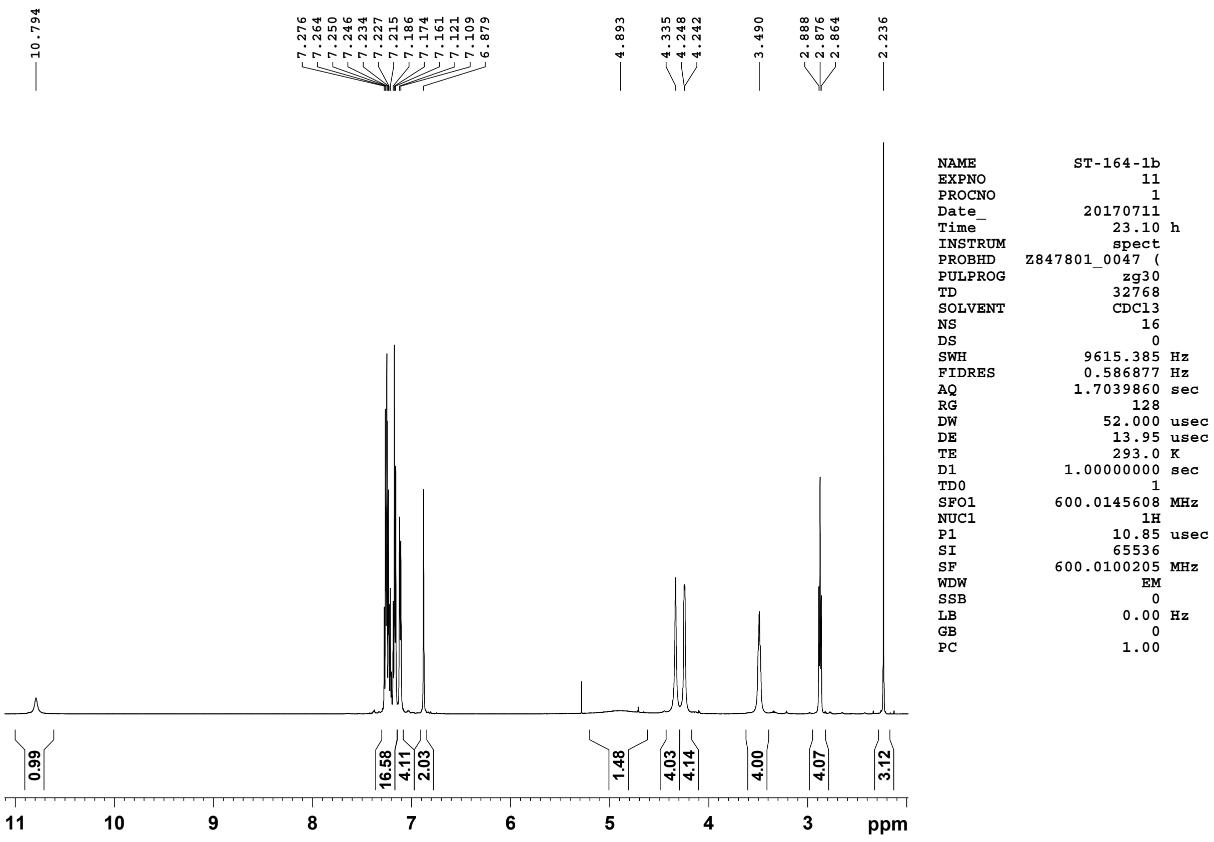

Figure S62. ^{1}H NMR spectrum of compound **3bc**.

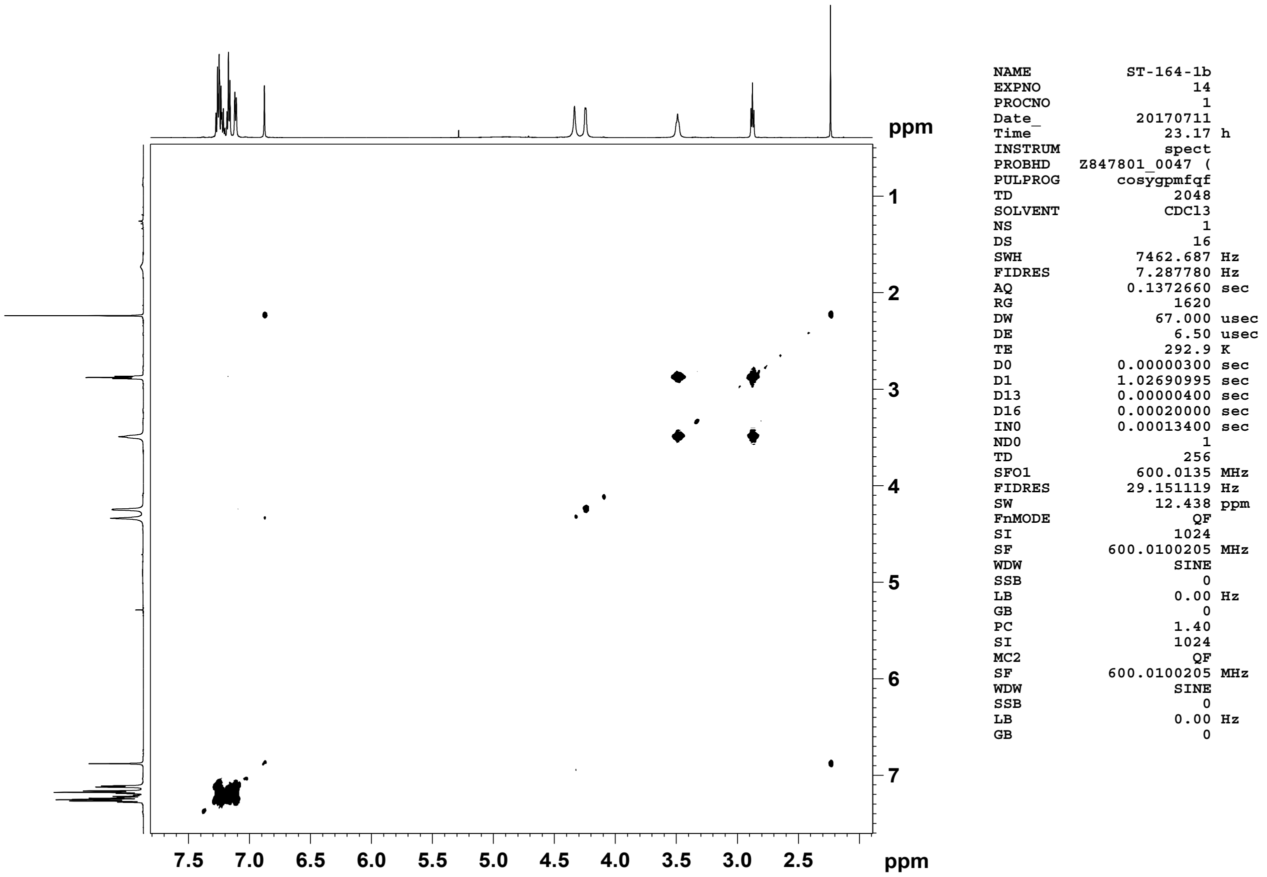

Figure S63. ^1H-^1H COSY spectrum of compound **3bc**.

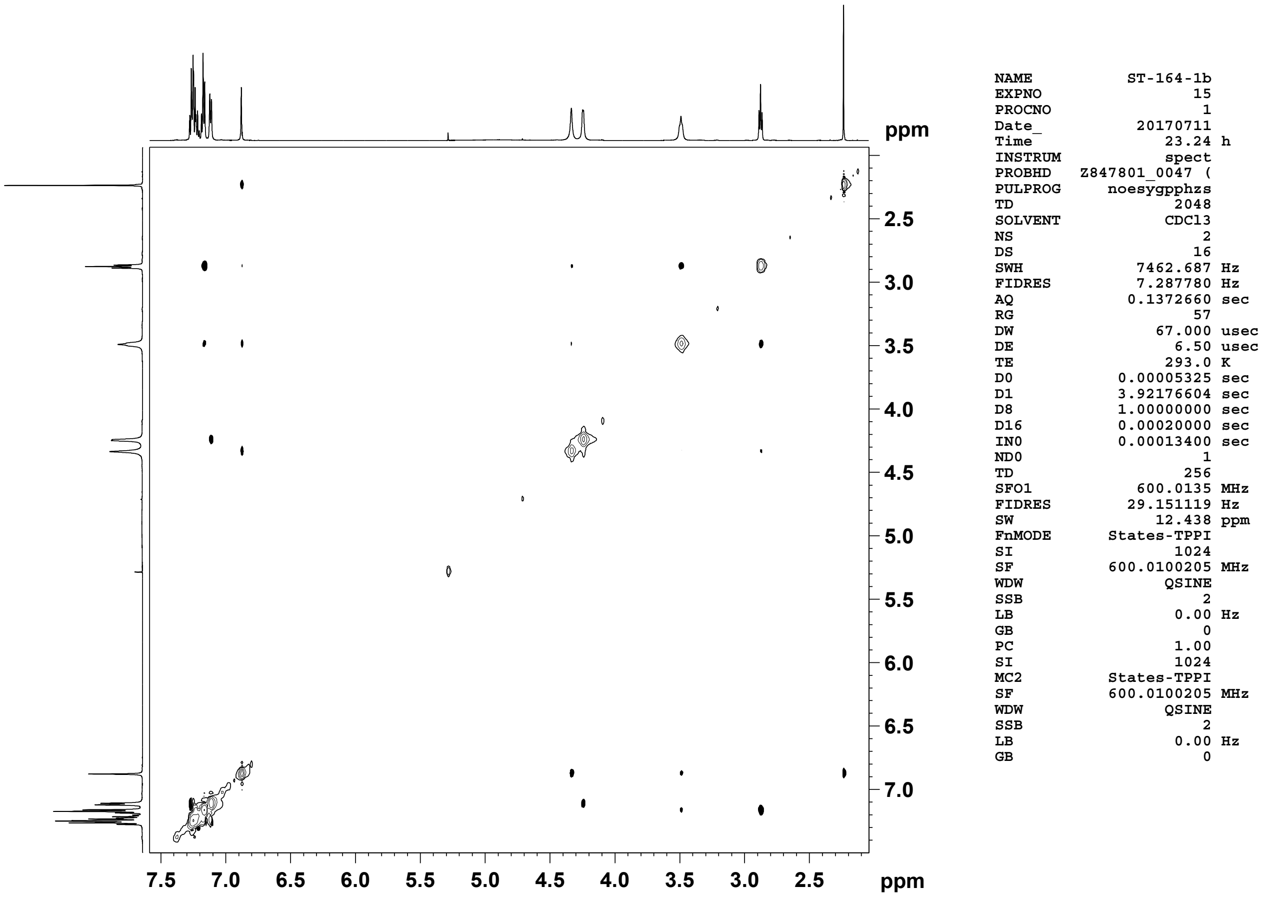

Figure S64. ^1H-^1H NOESY spectrum of compound **3bc**.

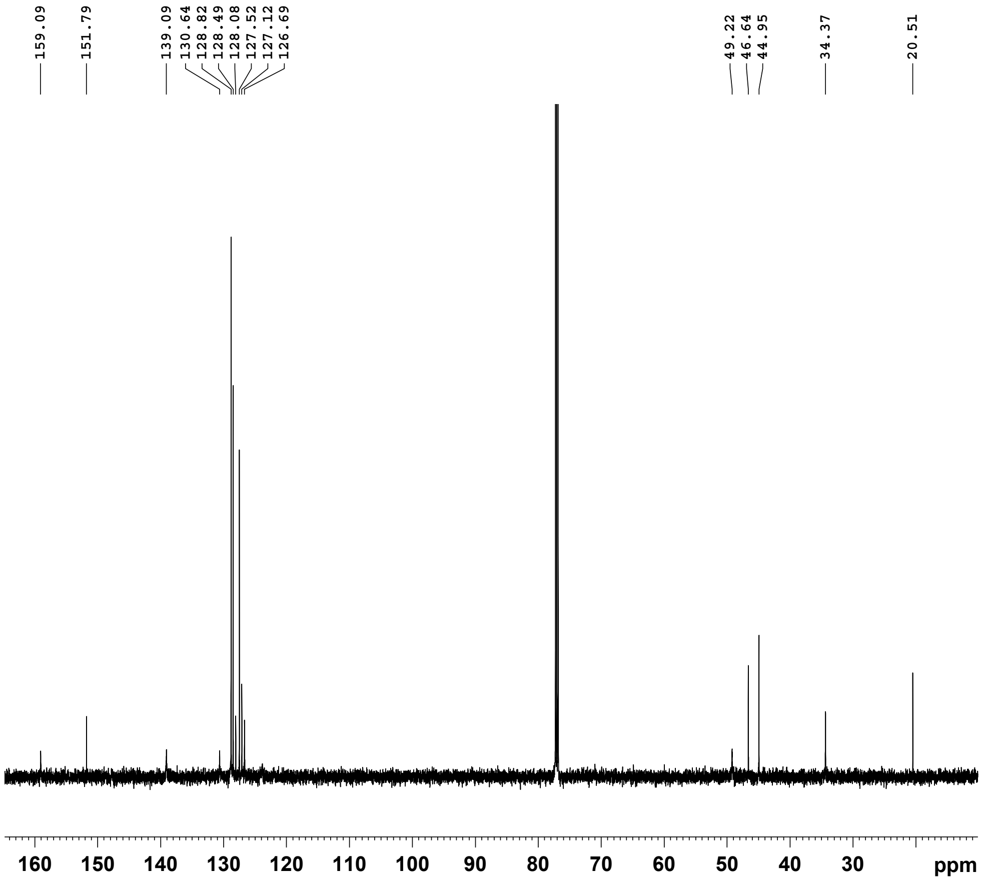

Figure S65. ^{13}C NMR spectrum of compound **3bc**.

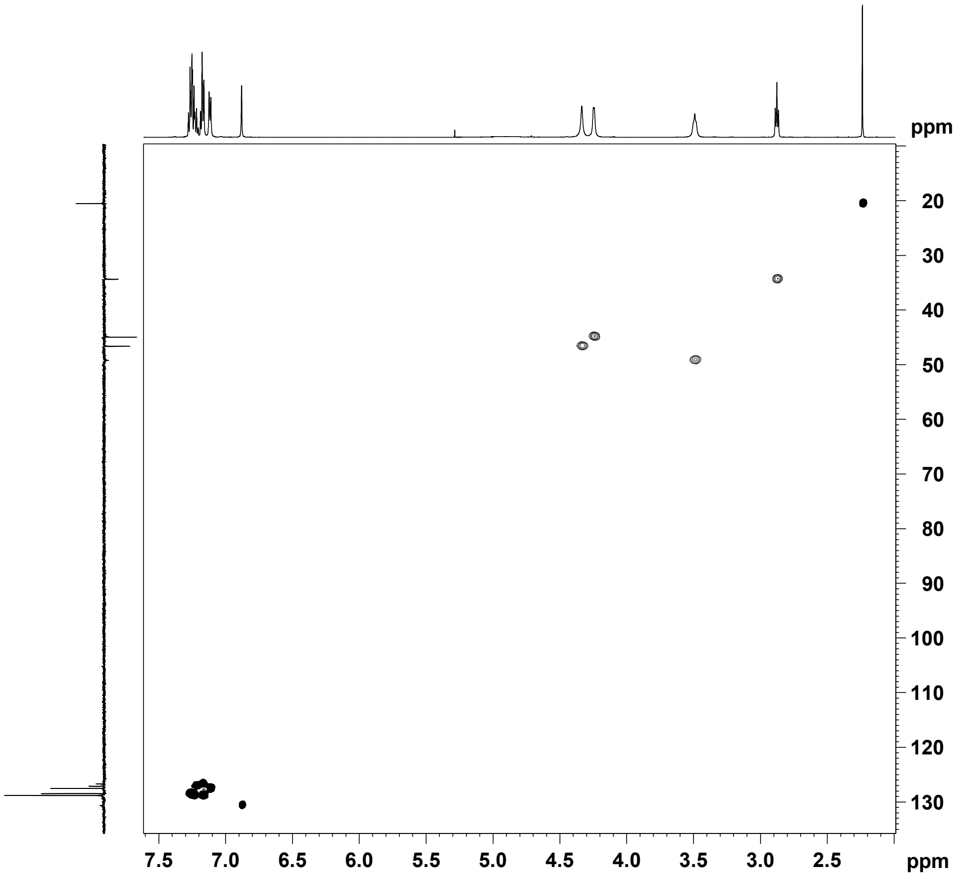

Figure S66. ^{1}H-^{13}C HSQC spectrum of compound **3bc**.

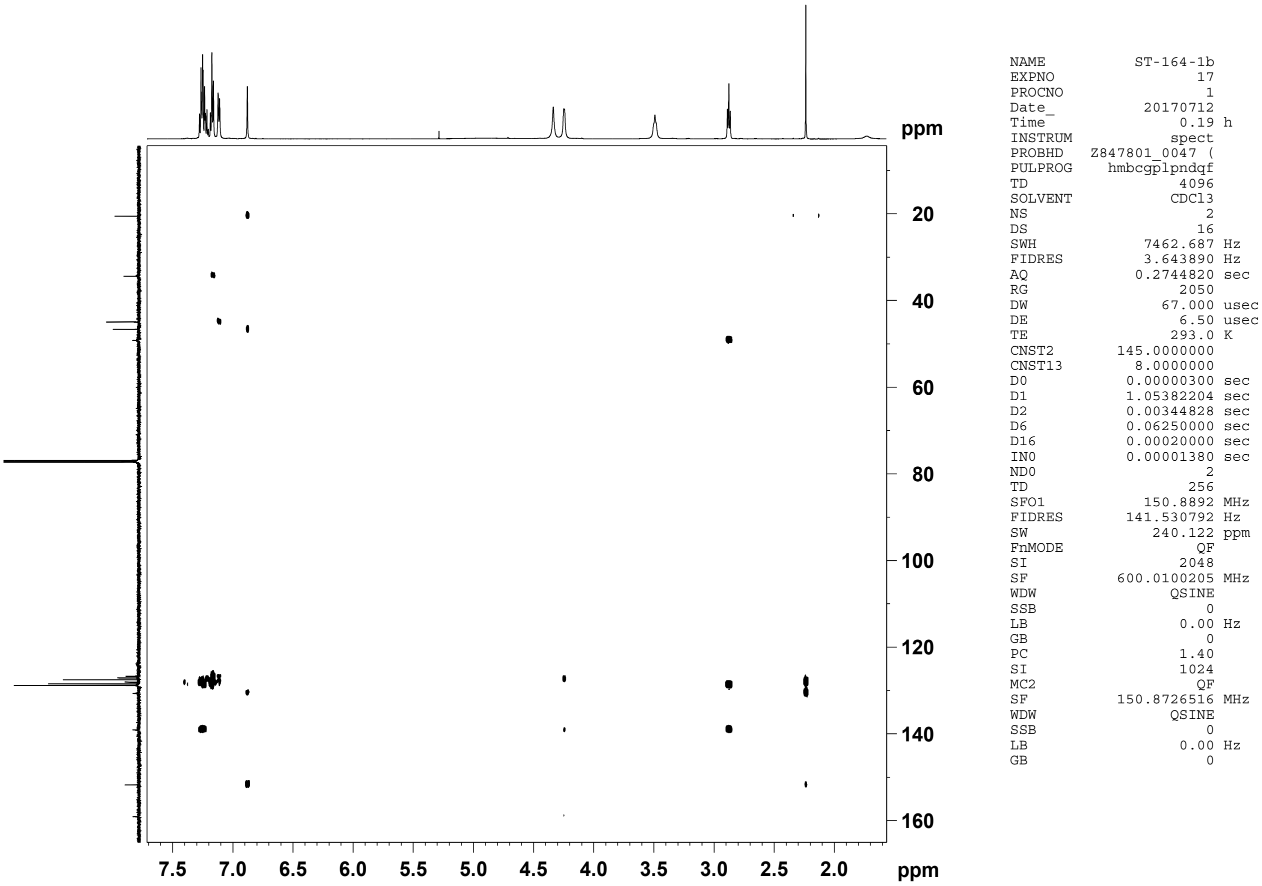

Figure S67. ^{1}H-^{13}C HMBC spectrum of compound **3bc**.

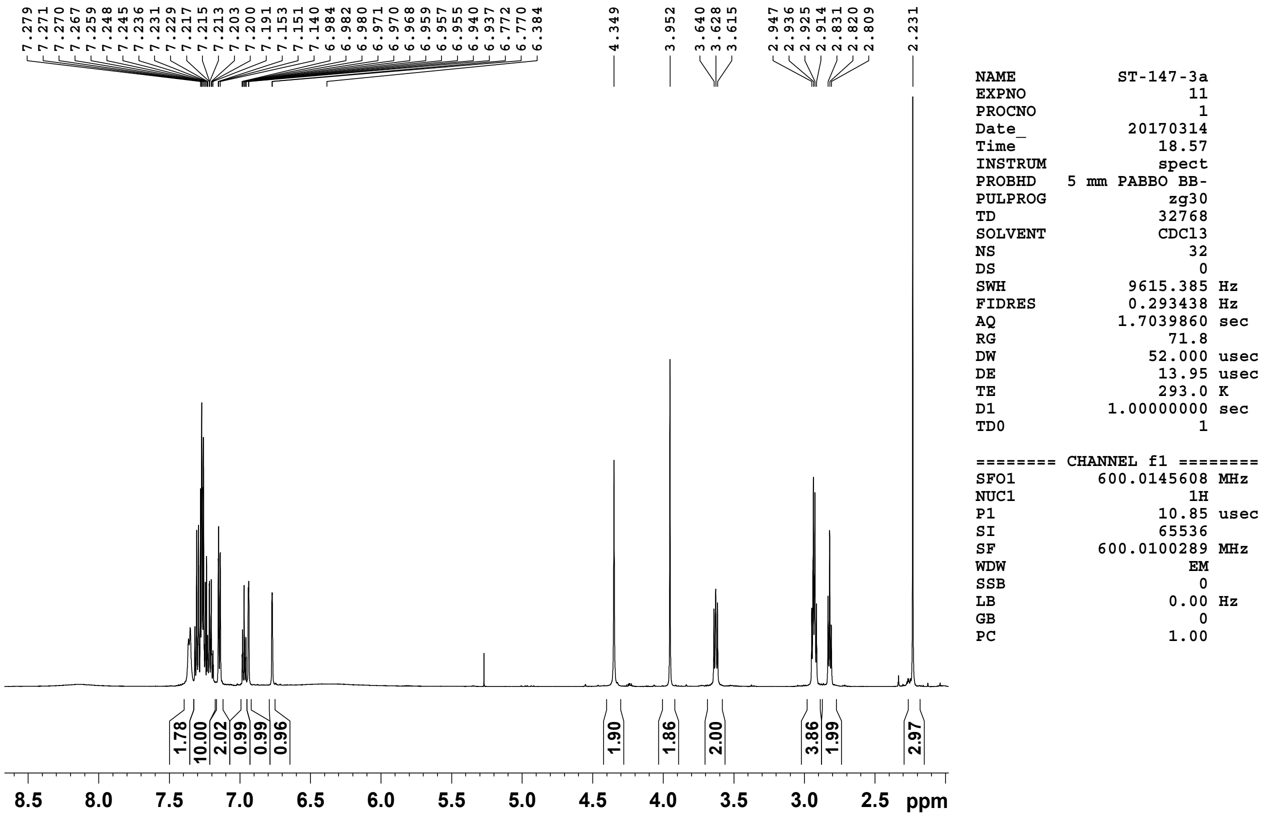

Figure S68. ^{1}H NMR spectrum of compound **2ca**.

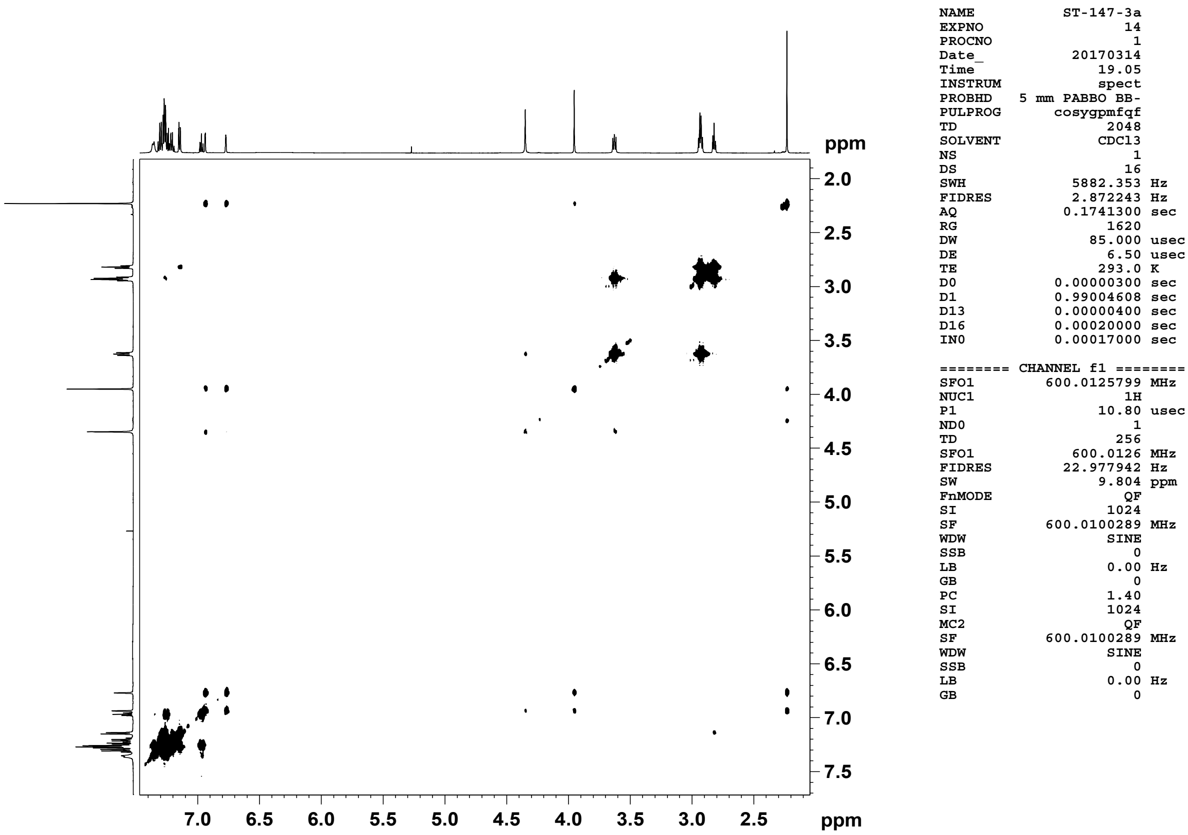

Figure S69. ^1H-^1H COSY spectrum of compound **2ca**.

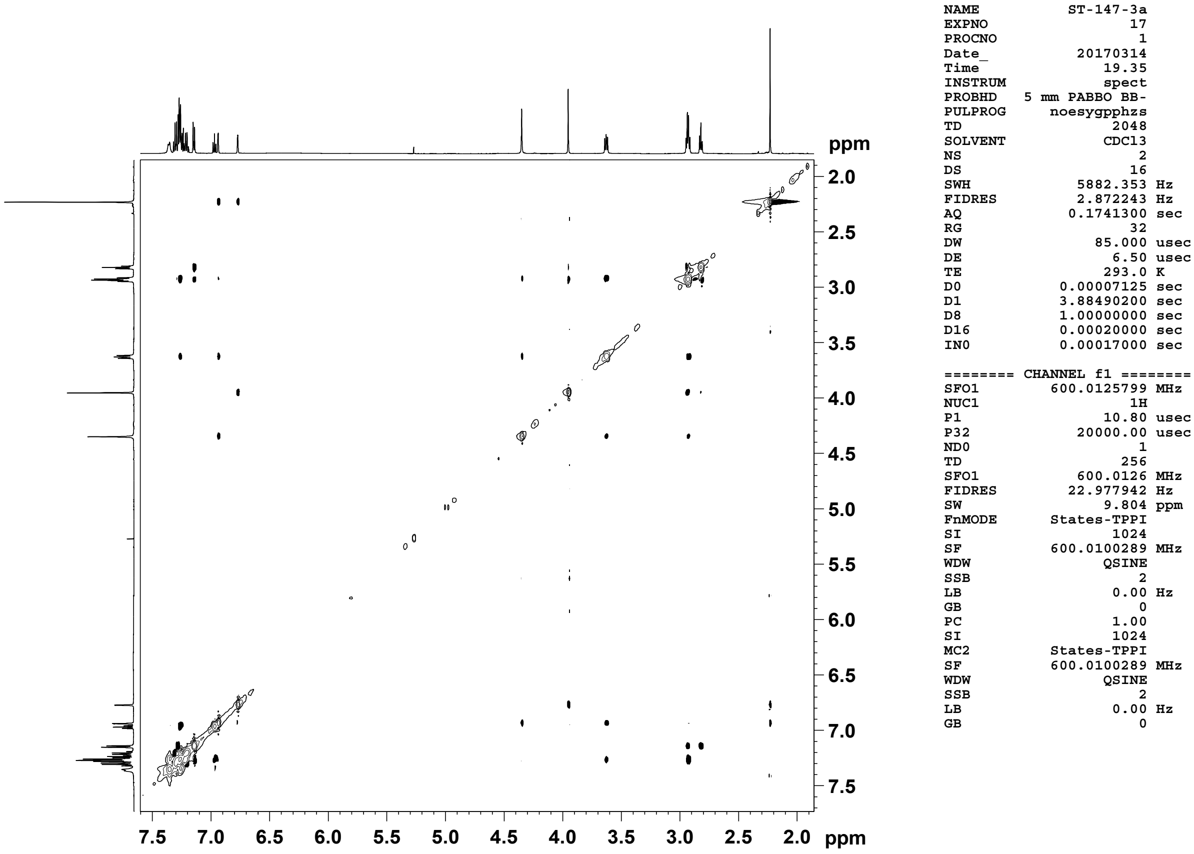

Figure S70. ^1H-^1H NOESY spectrum of compound **2ca**.

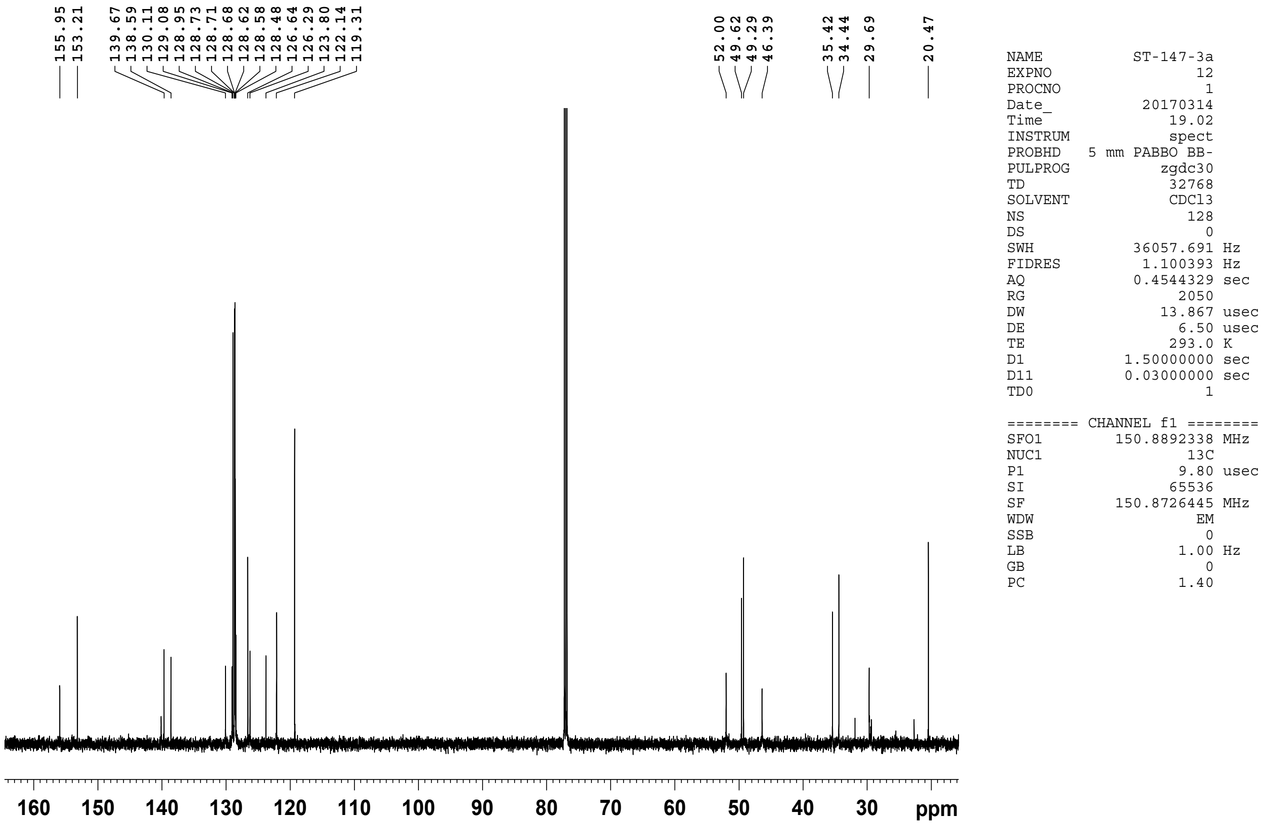

Figure S71. ^{13}C NMR spectrum of compound **2ca**.

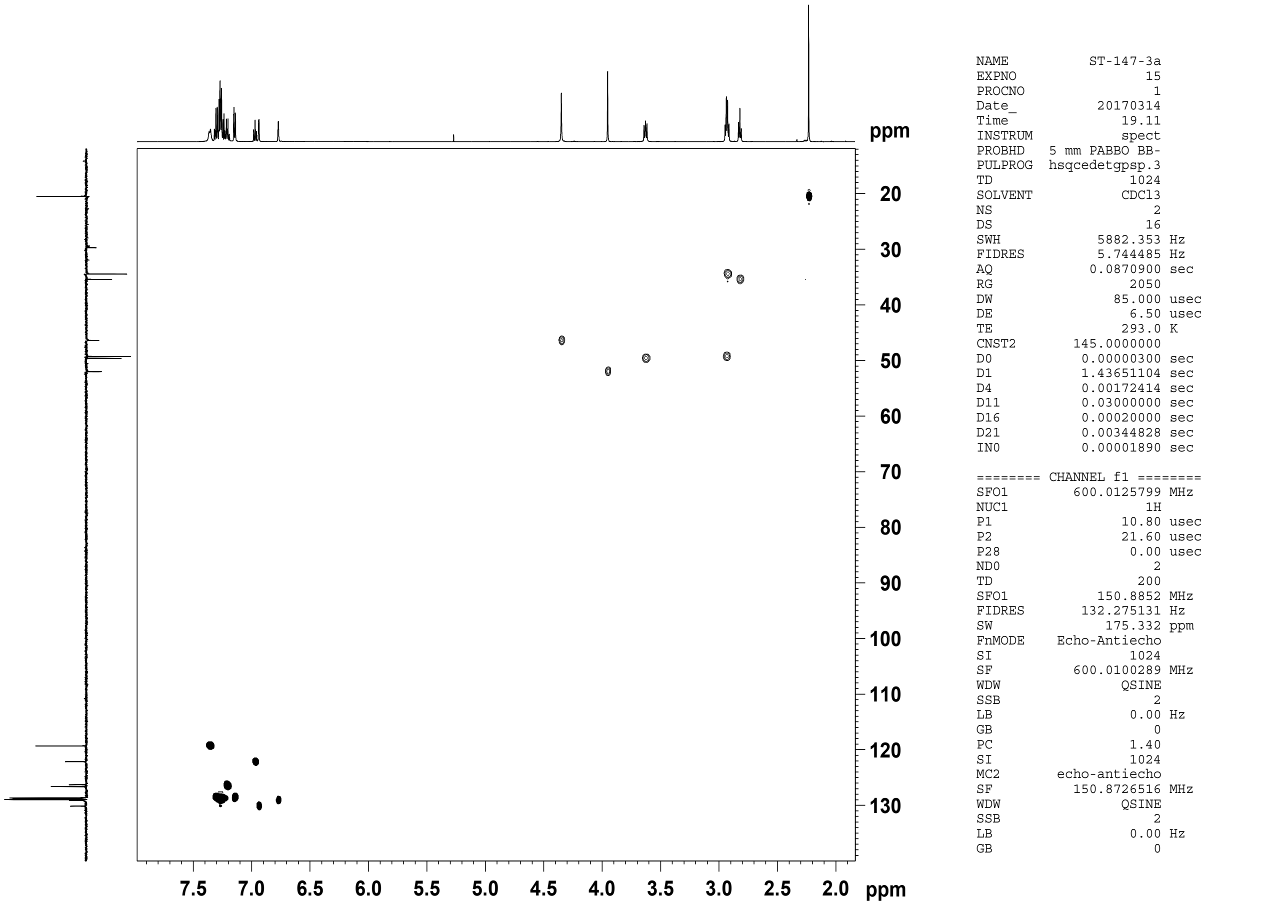

Figure S72. ^{1}H-^{13}C HSQC spectrum of compound **2ca**.

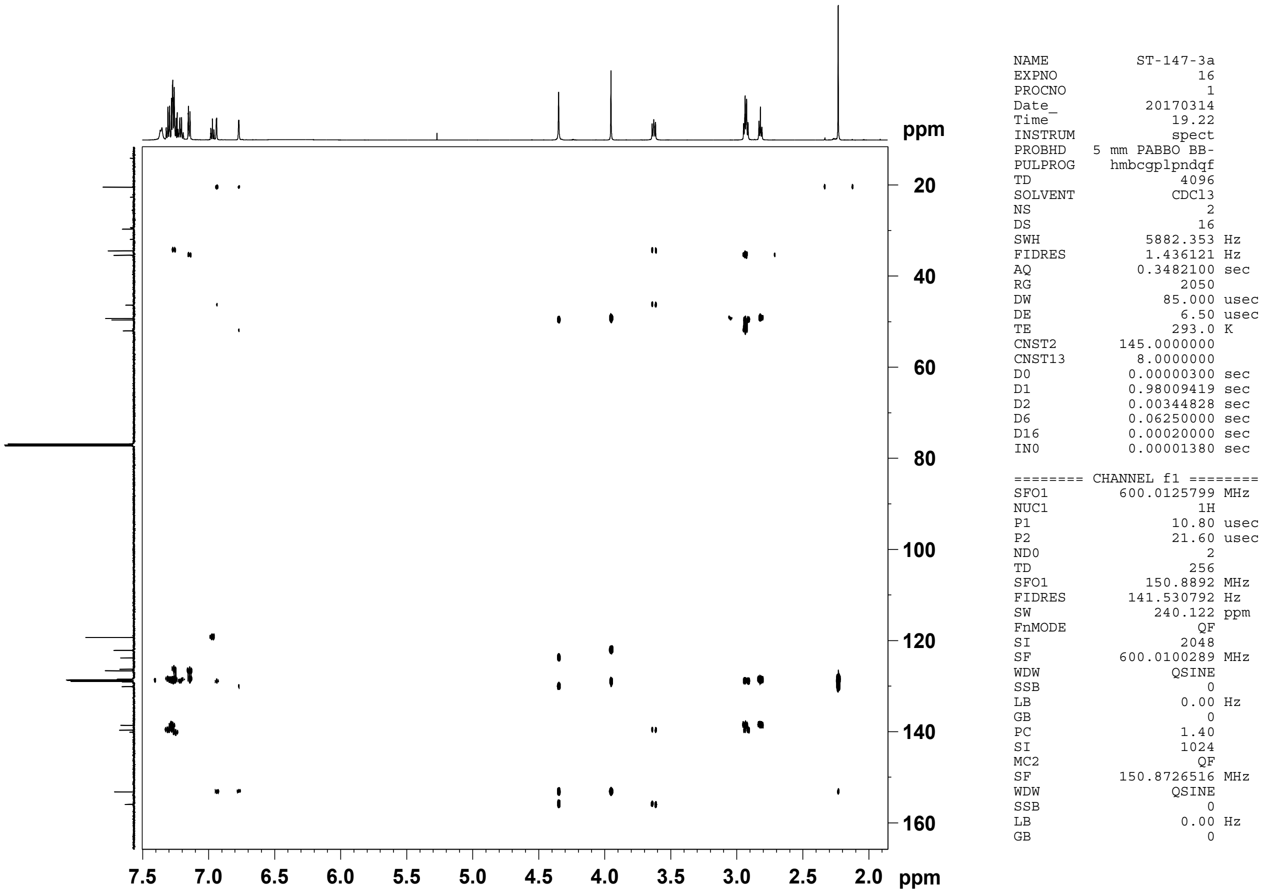

Figure S73. ^{1}H-^{13}C HMBC spectrum of compound **2ca**.

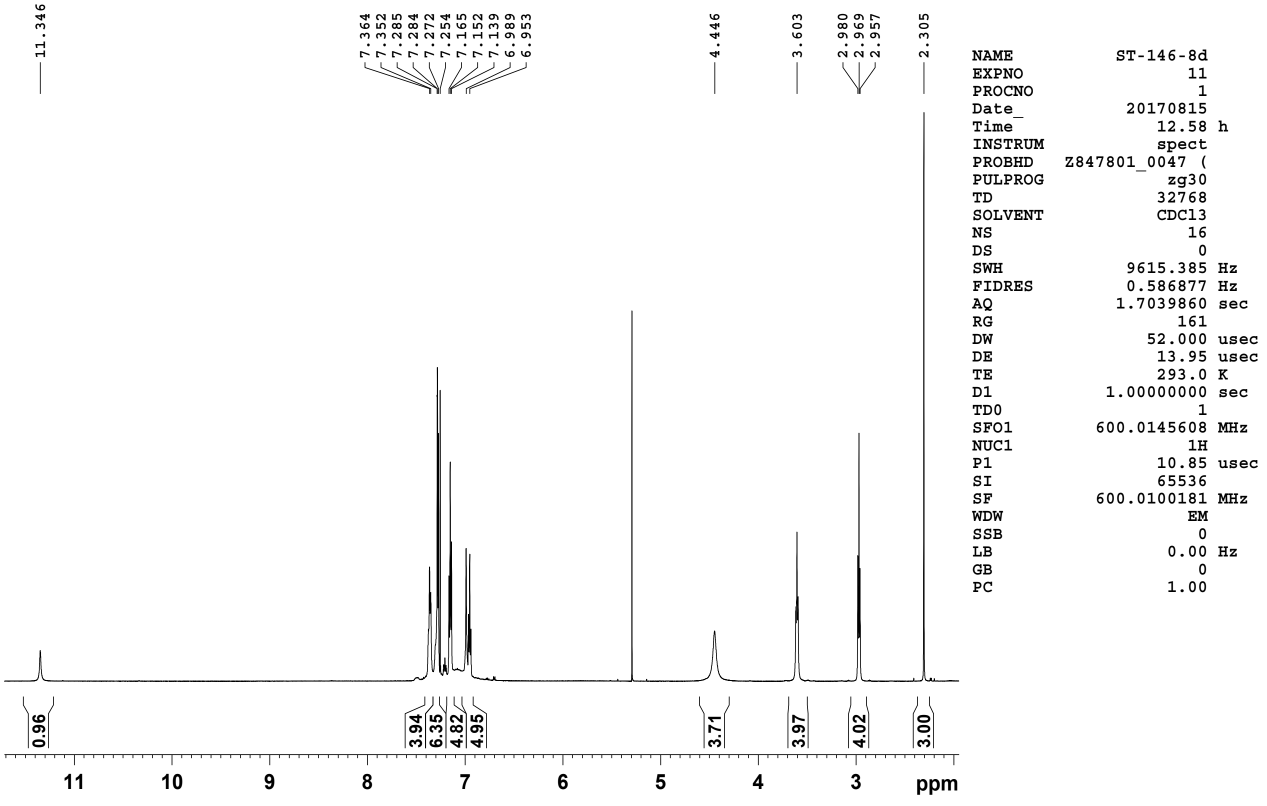

Figure S74. ^{1}H NMR spectrum of compound **3ca**.

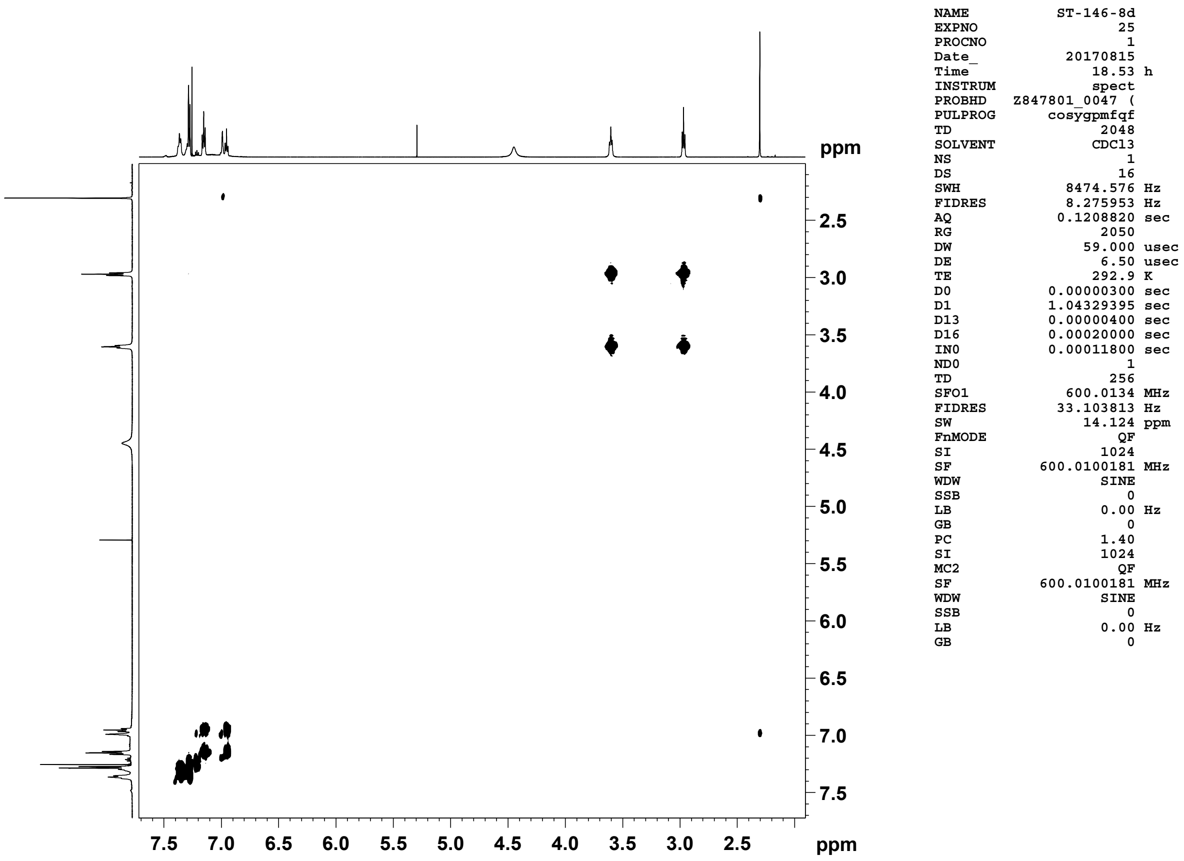

Figure S75. ^{1}H-^{1}H COSY spectrum of compound **3ca**.

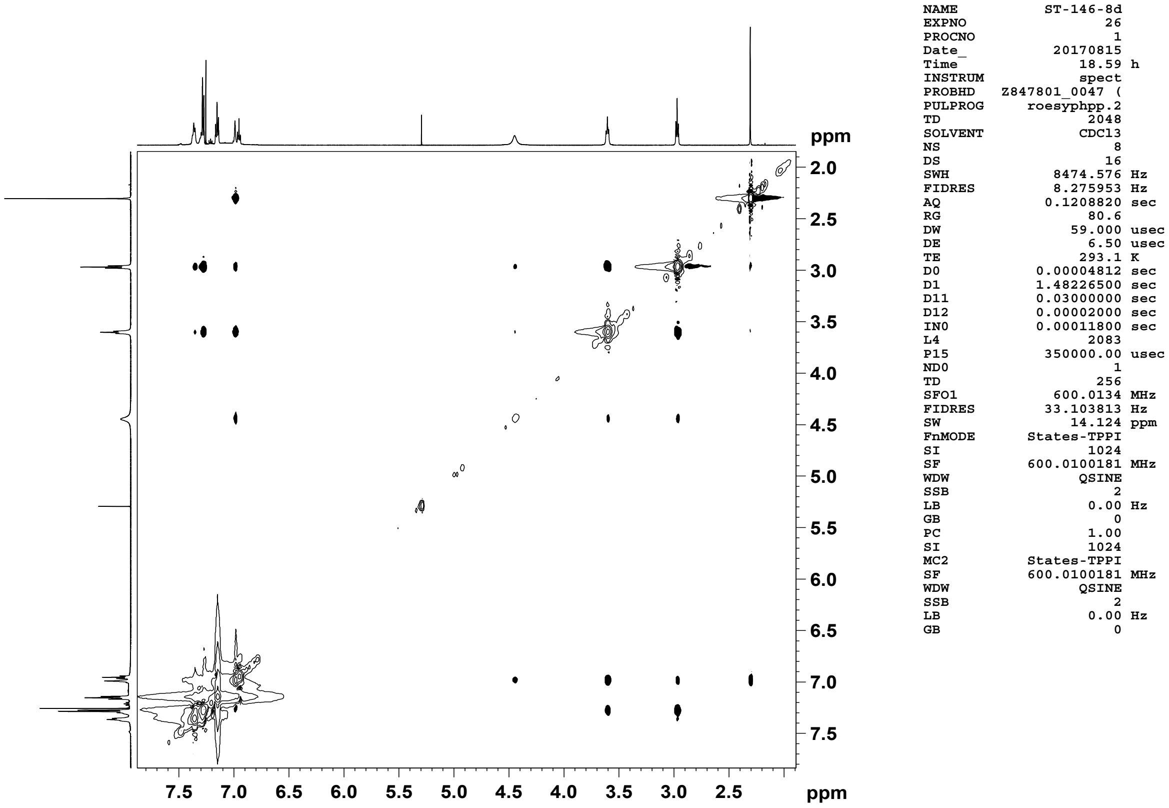

Figure S76. ^{1}H-^{1}H NOESY spectrum of compound **3ca**.

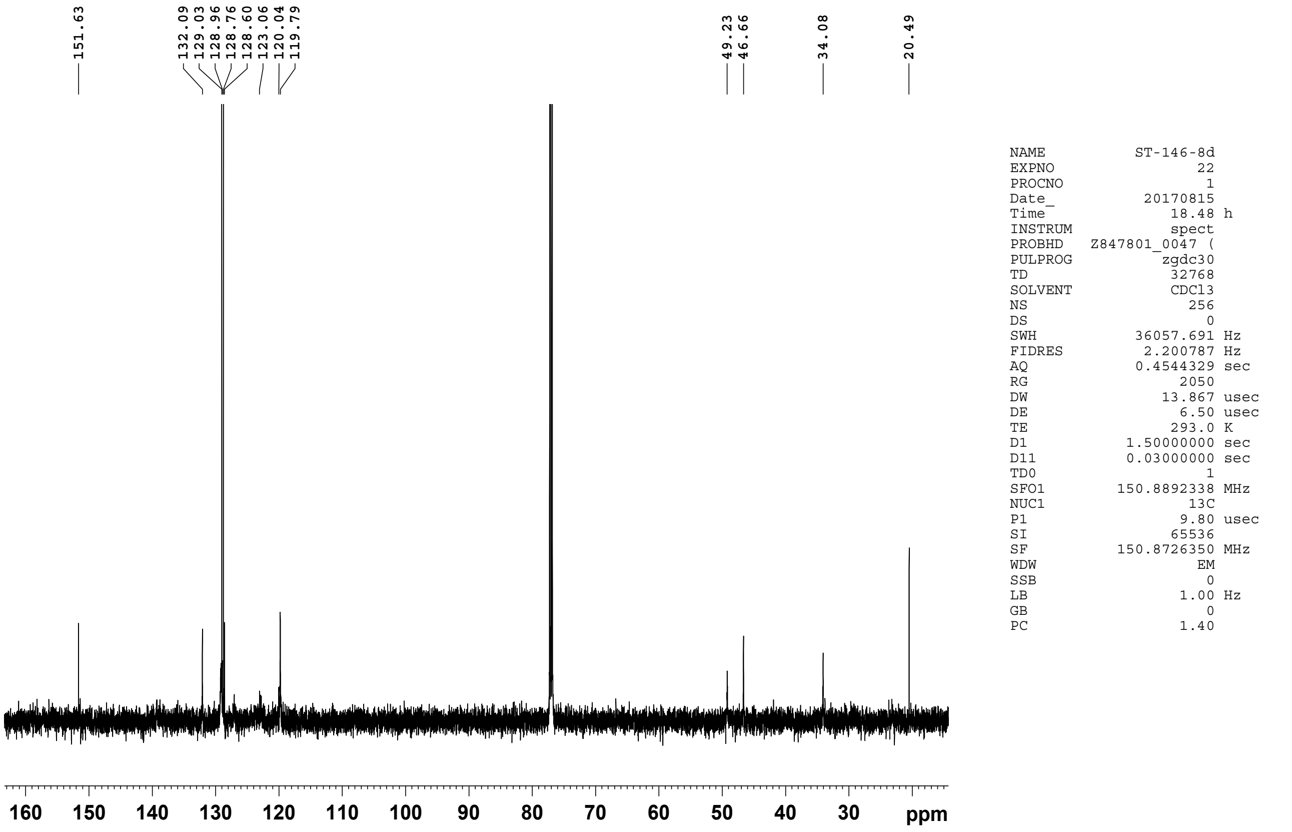

Figure S77. ^{13}C NMR spectrum of compound **3ca**.

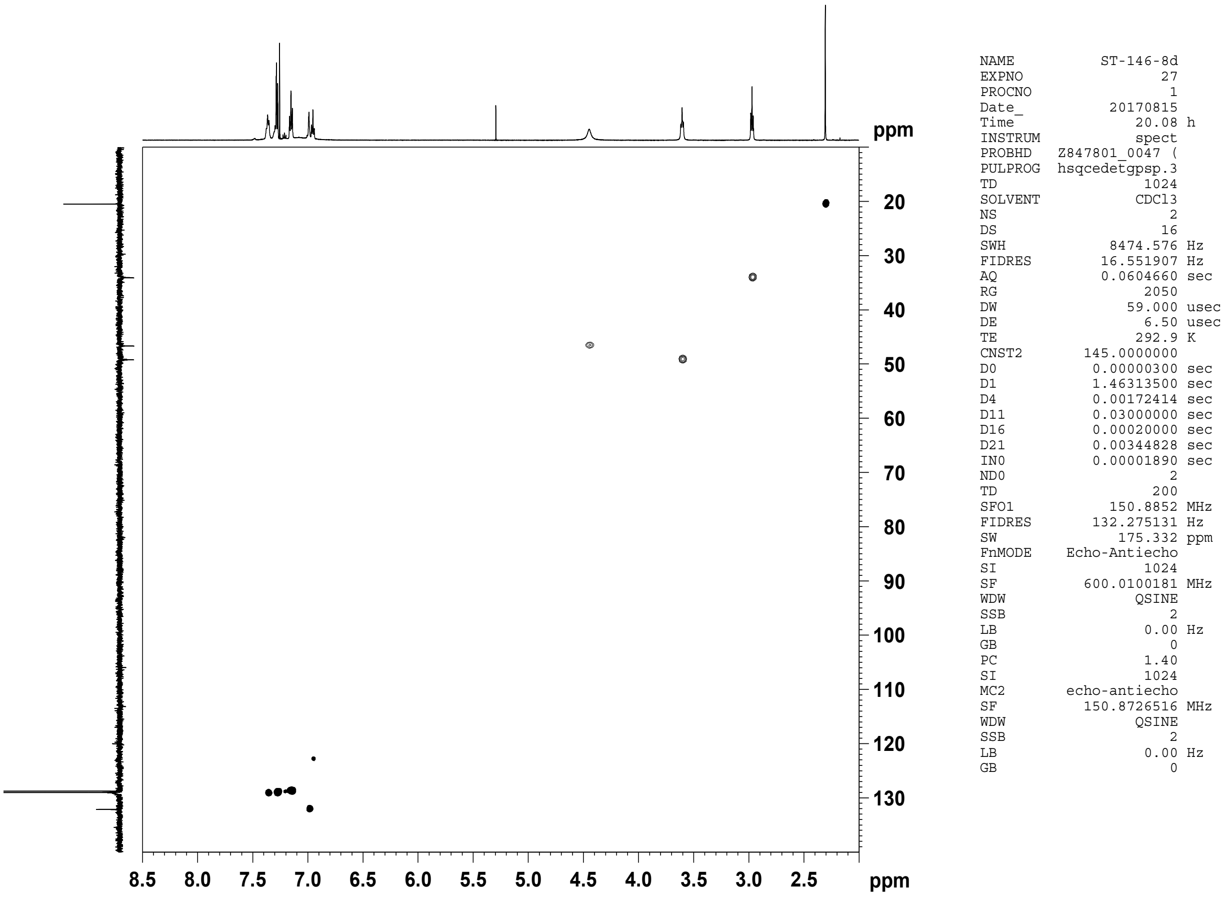

Figure S78. ^{1}H-^{13}C HSQC spectrum of compound **3ca**.

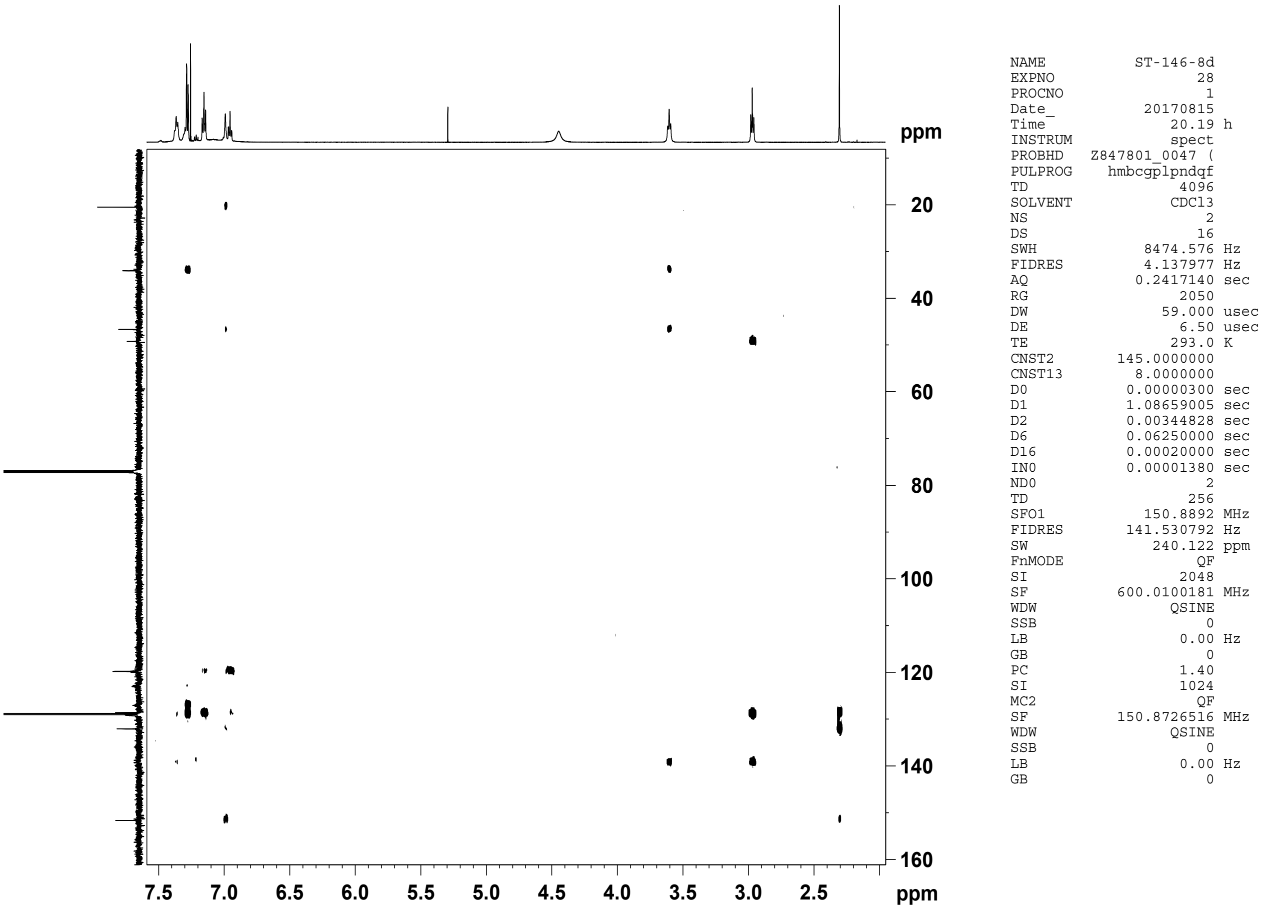

Figure S79. ^{1}H-^{13}C HMBC spectrum of compound **3ca**.

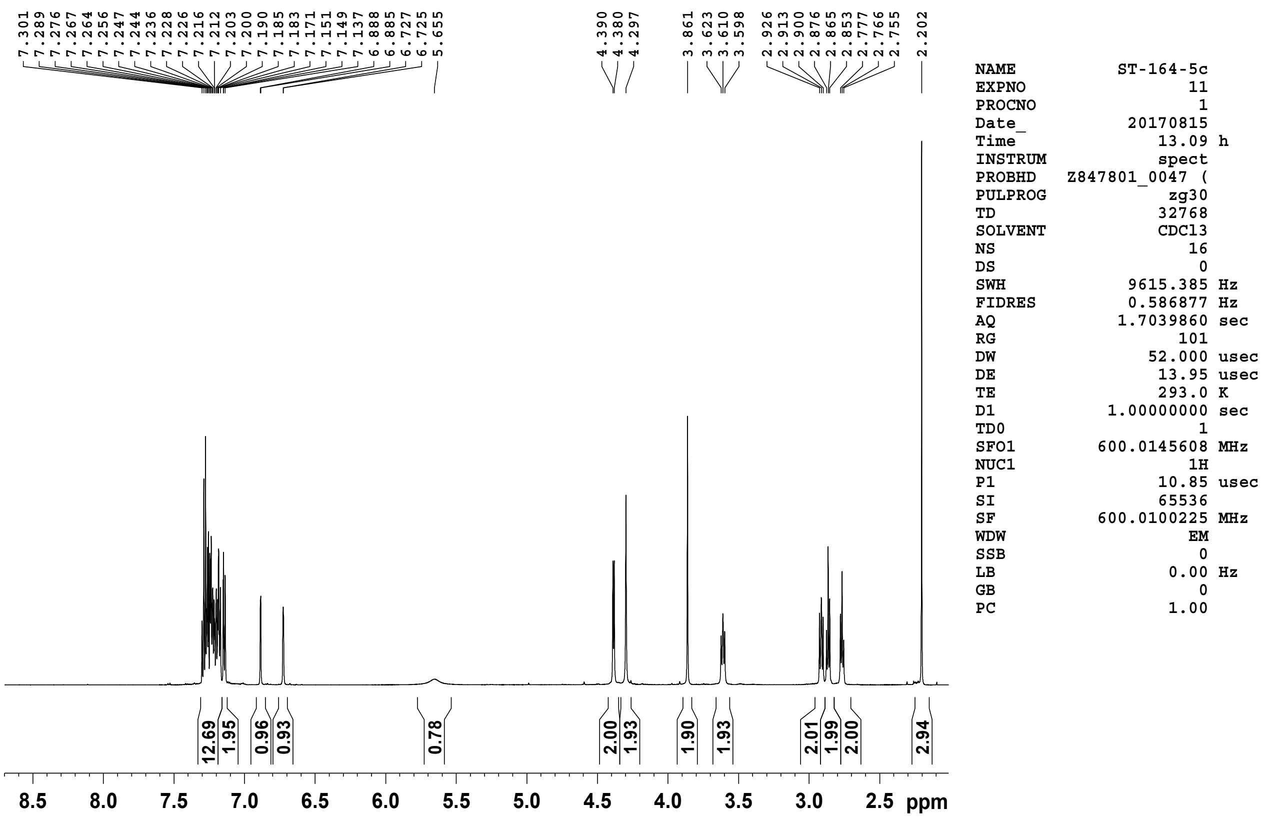

Figure S80. ^{1}H NMR spectrum of compound **2cb**.

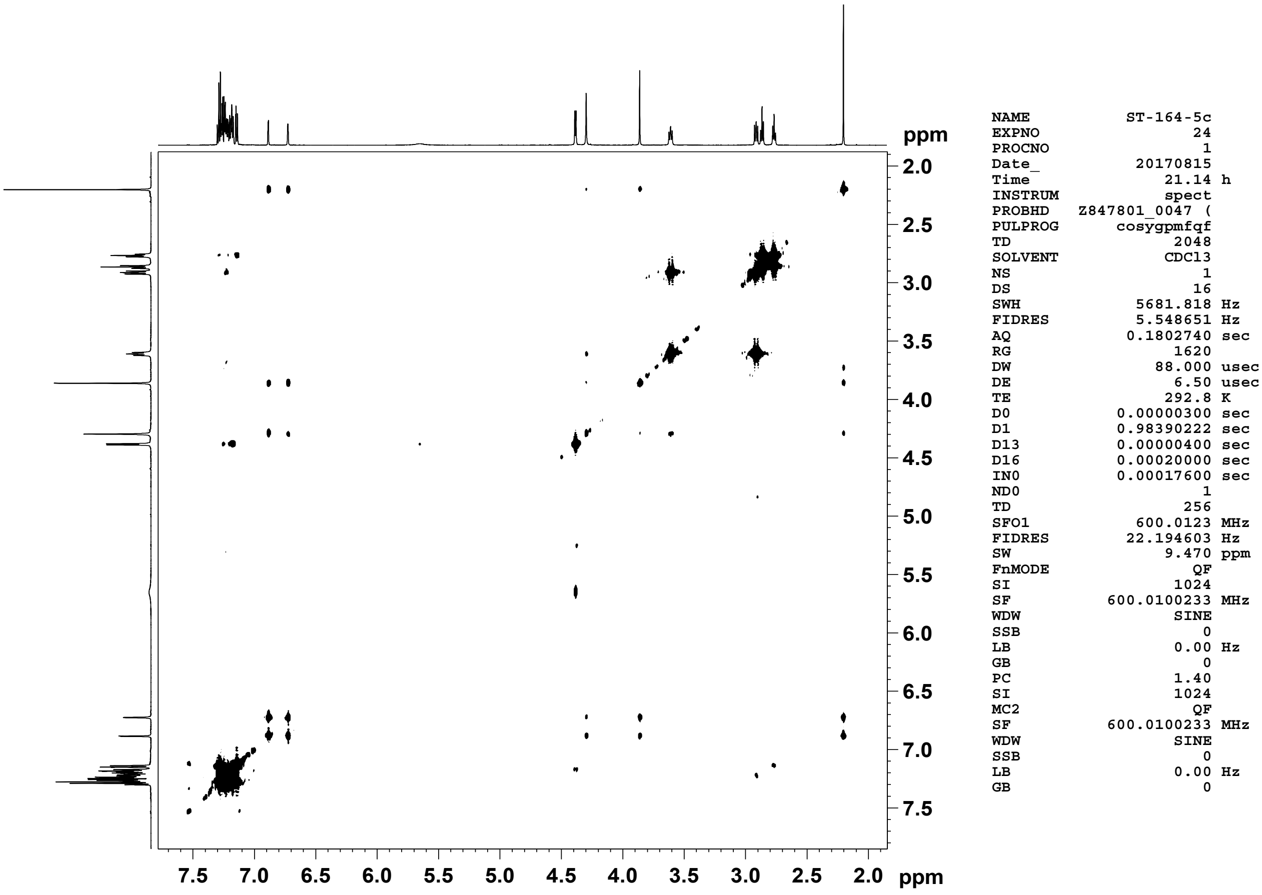

Figure S81. ^1H-^1H COSY spectrum of compound **2cb**.

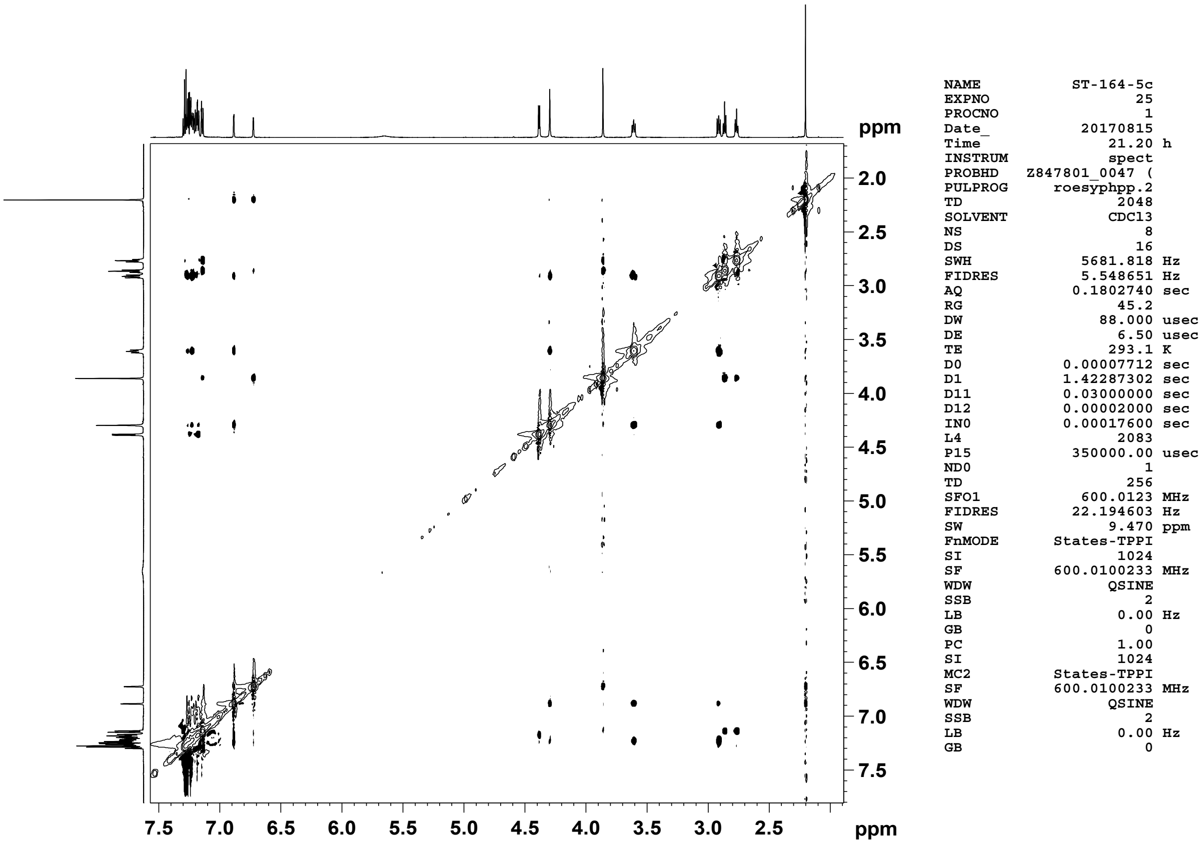

Figure S82. ^1H-^1H NOESY spectrum of compound **2cb**.

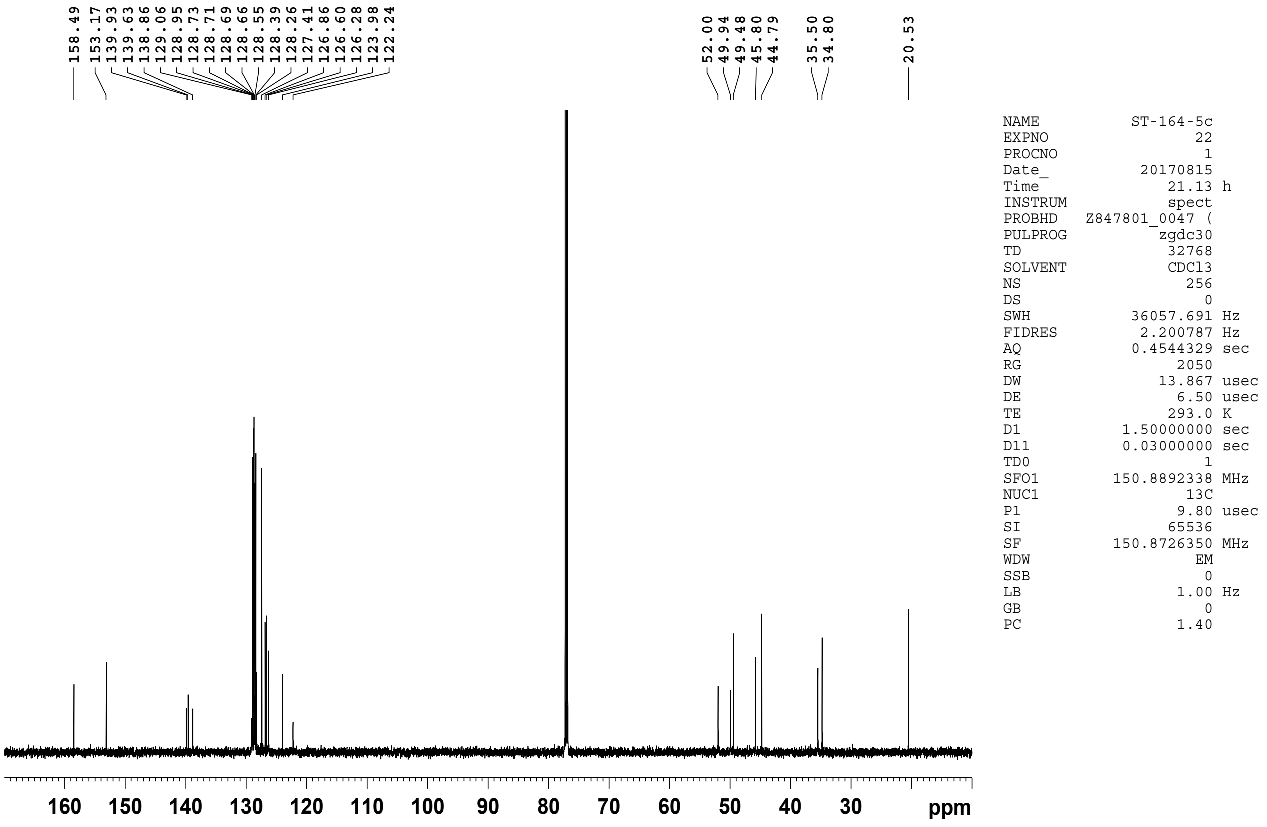

Figure S83. ^{13}C NMR spectrum of compound **2cb**.

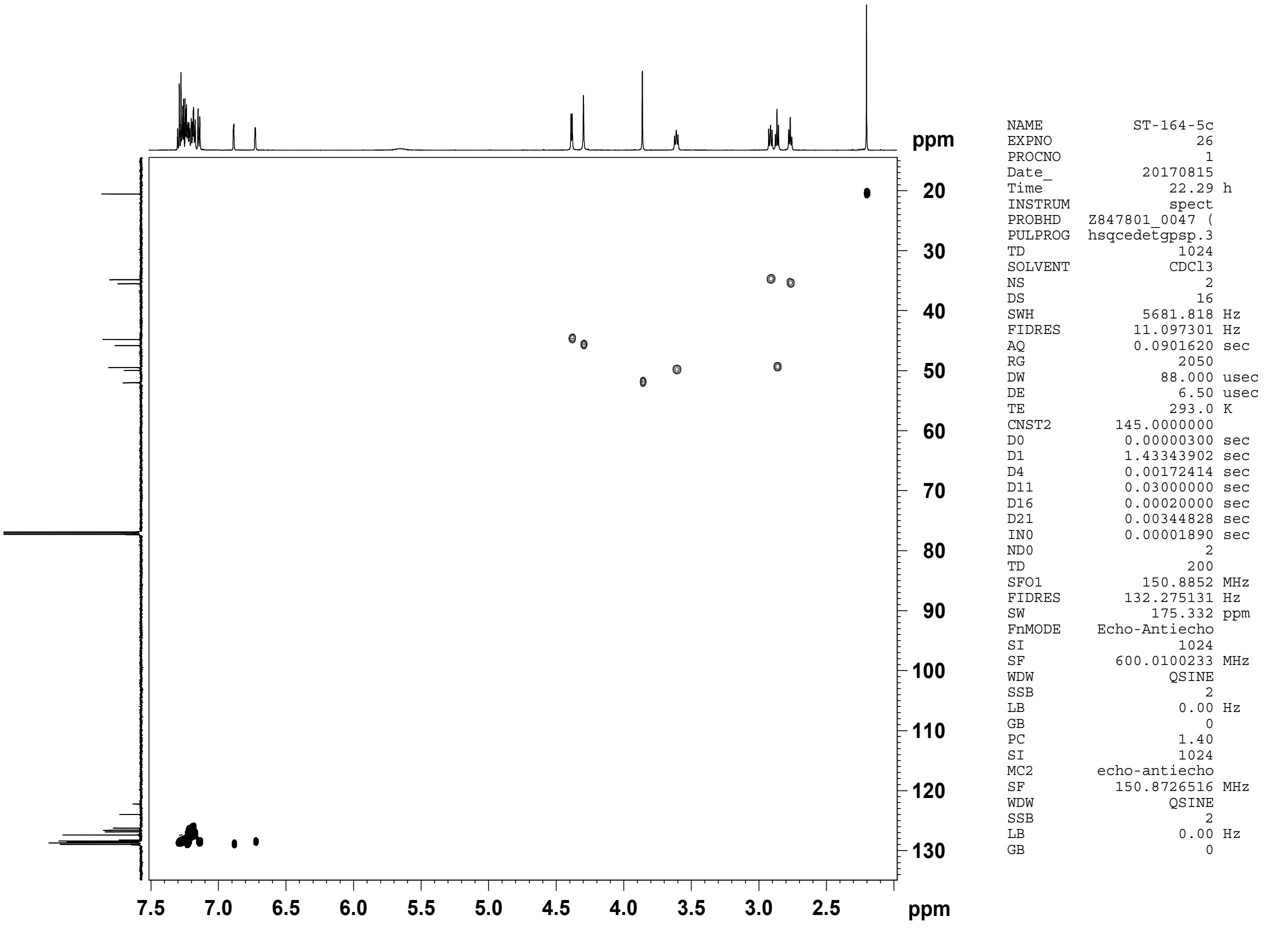

Figure S84. ^{1}H-^{13}C HSQC spectrum of compound **2cb**.

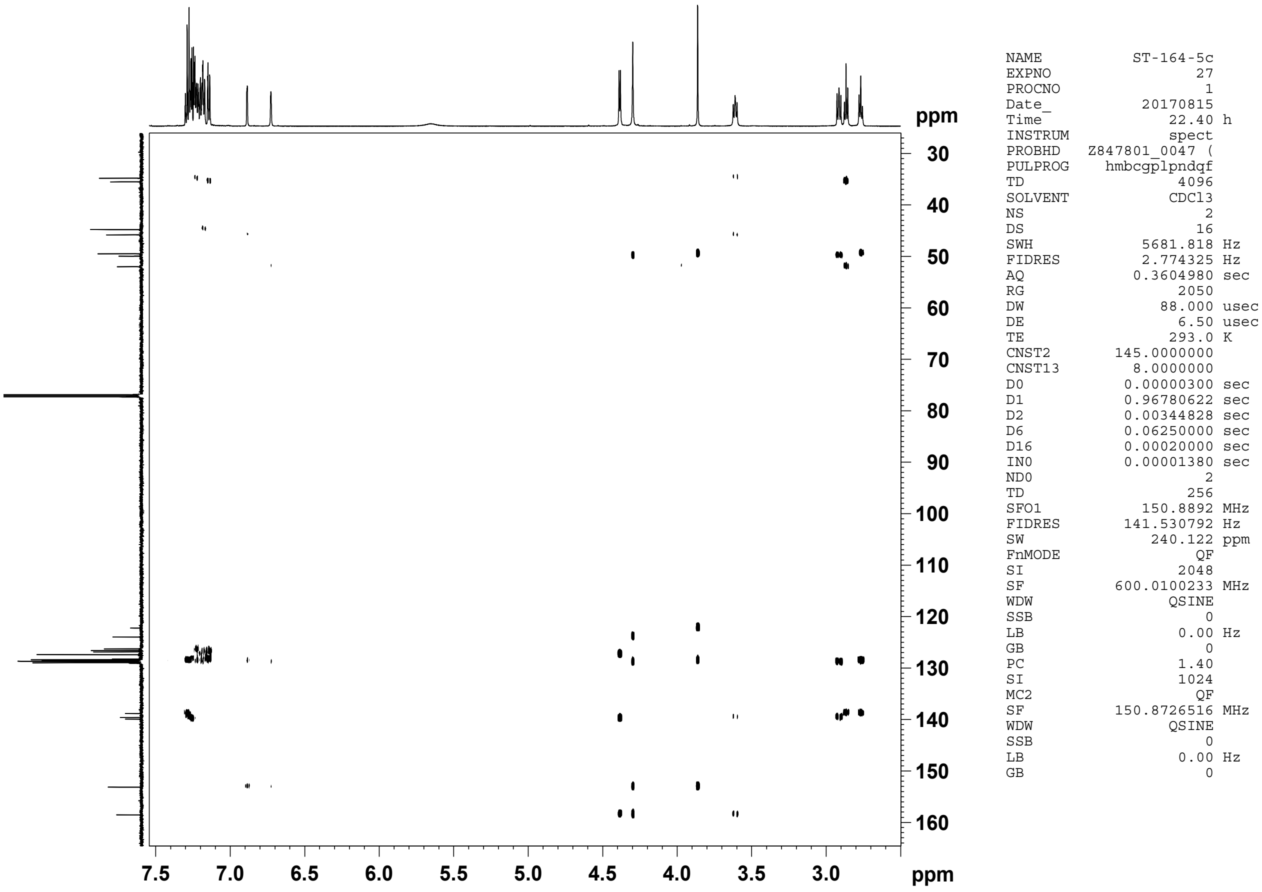

Figure S85. ^{1}H-^{13}C HMBC spectrum of compound **2cb**.

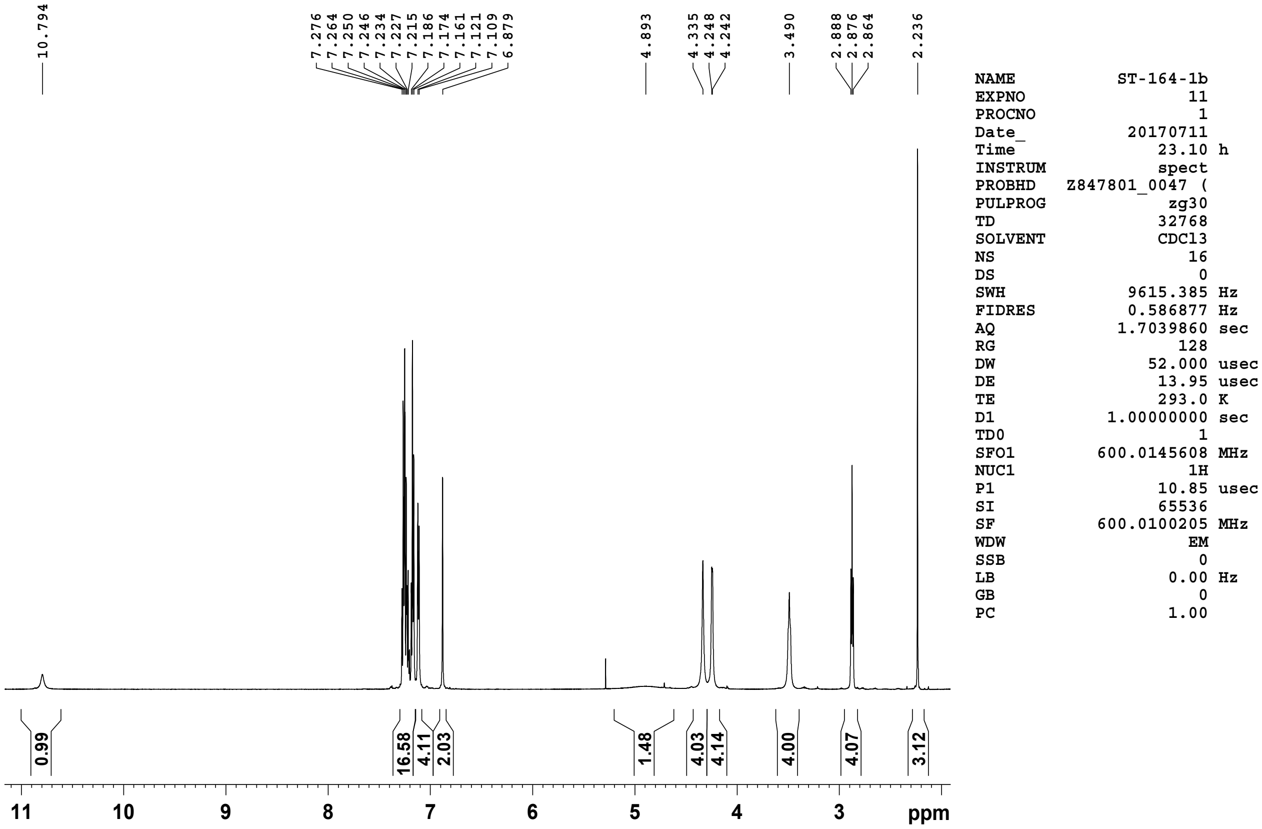

Figure S86. ^{1}H NMR spectrum of compound **3cb**.

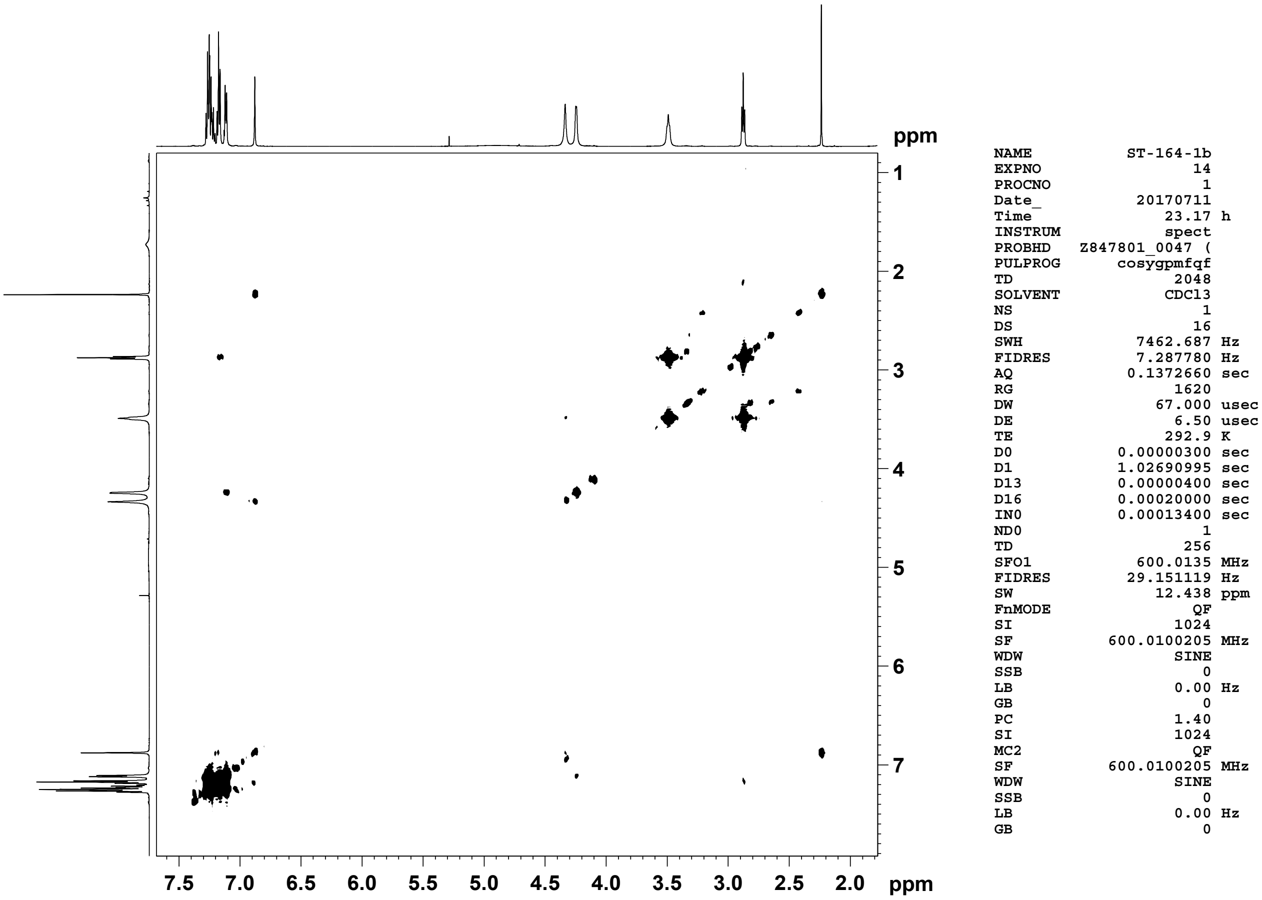

Figure S87. ^1H-^1H COSY spectrum of compound **3cb**.

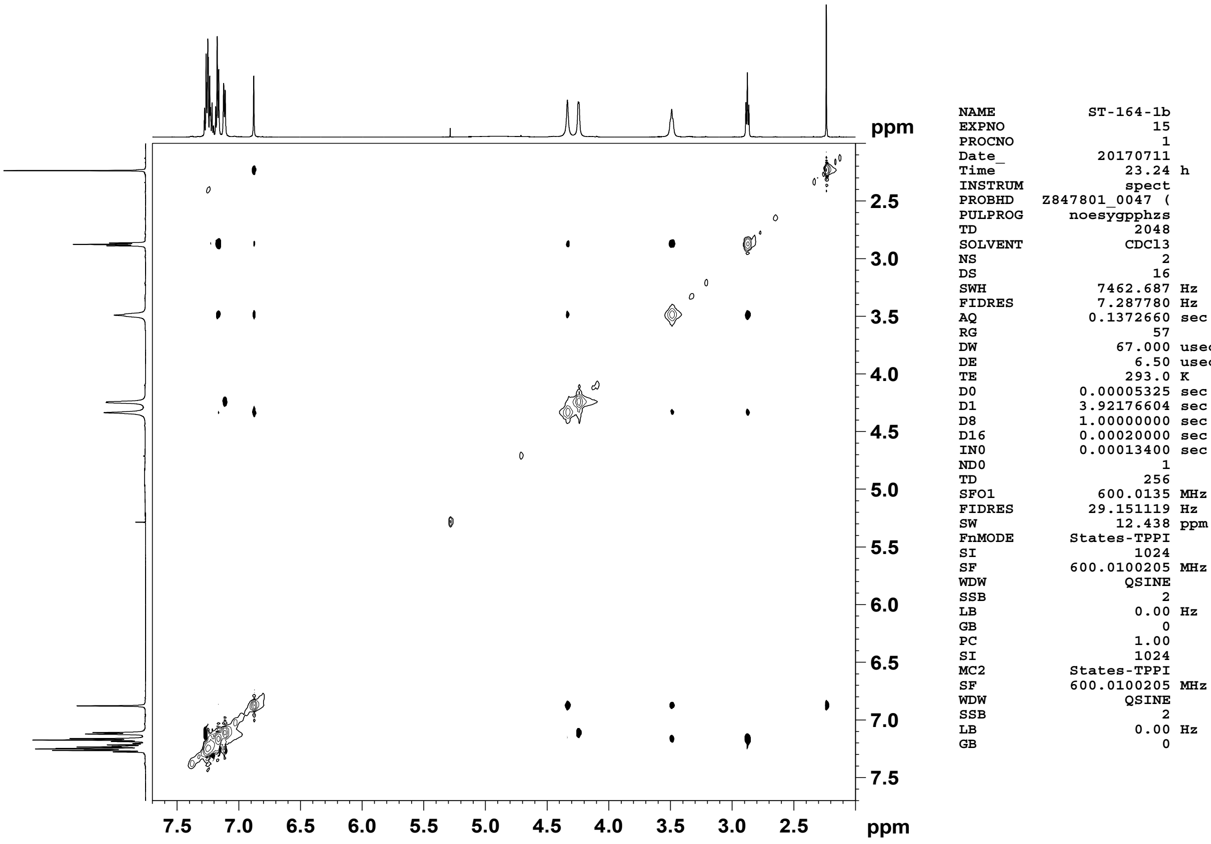

Figure S88. ^1H-^1H NOESY spectrum of compound **3cb**.

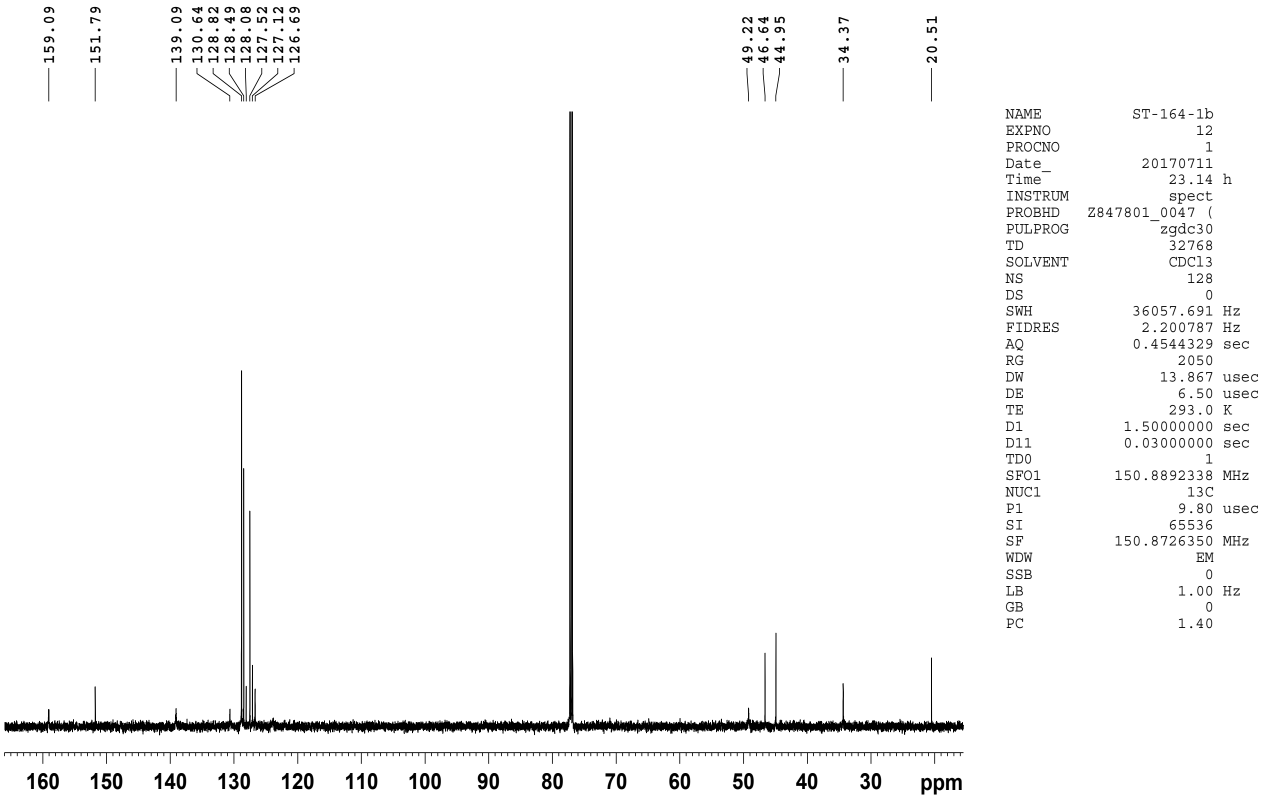

Figure S89. ^{13}C NMR spectrum of compound **3cb**.

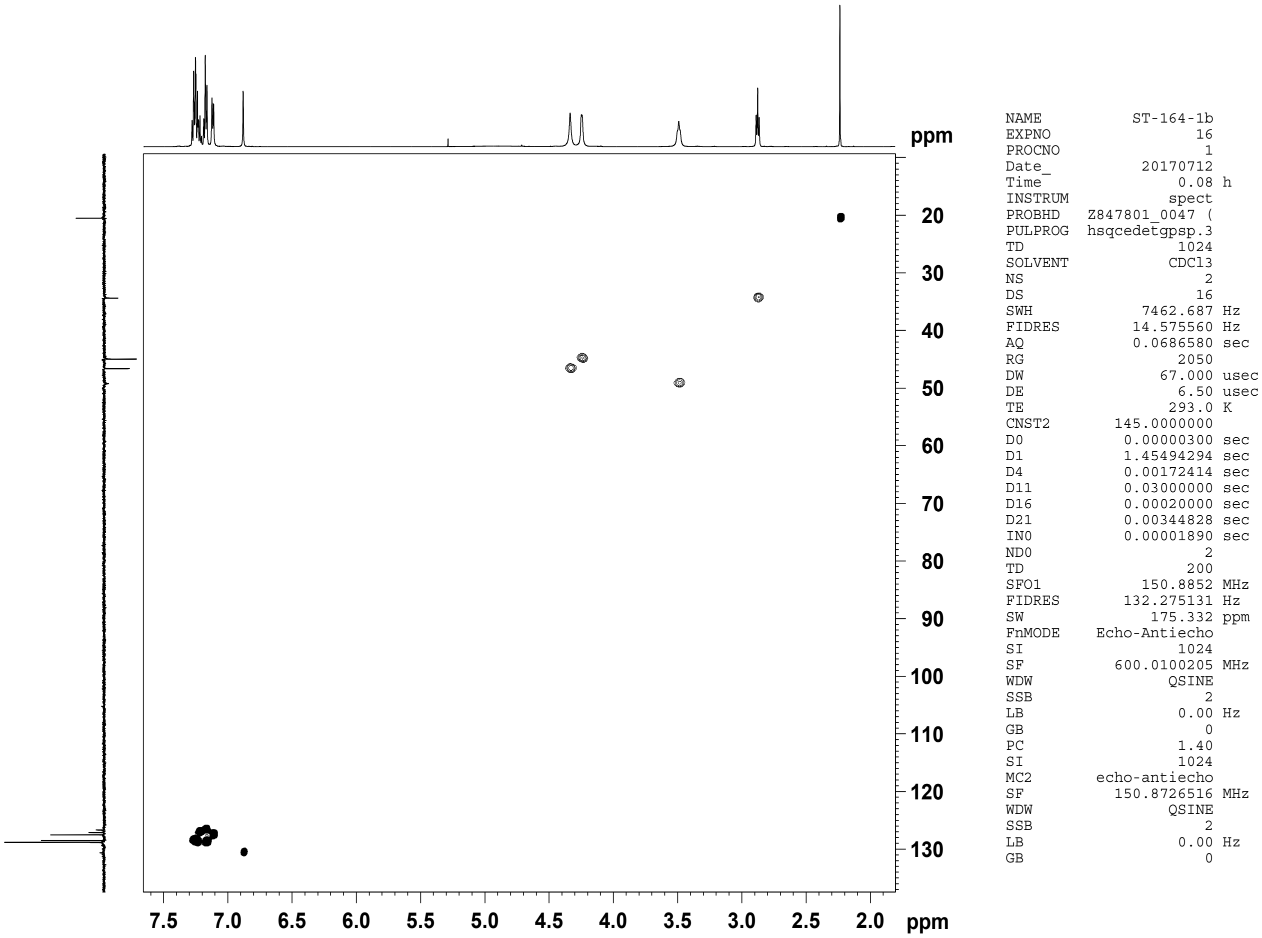

Figure S90. ^{1}H-^{13}C HSQC spectrum of compound **3cb**.

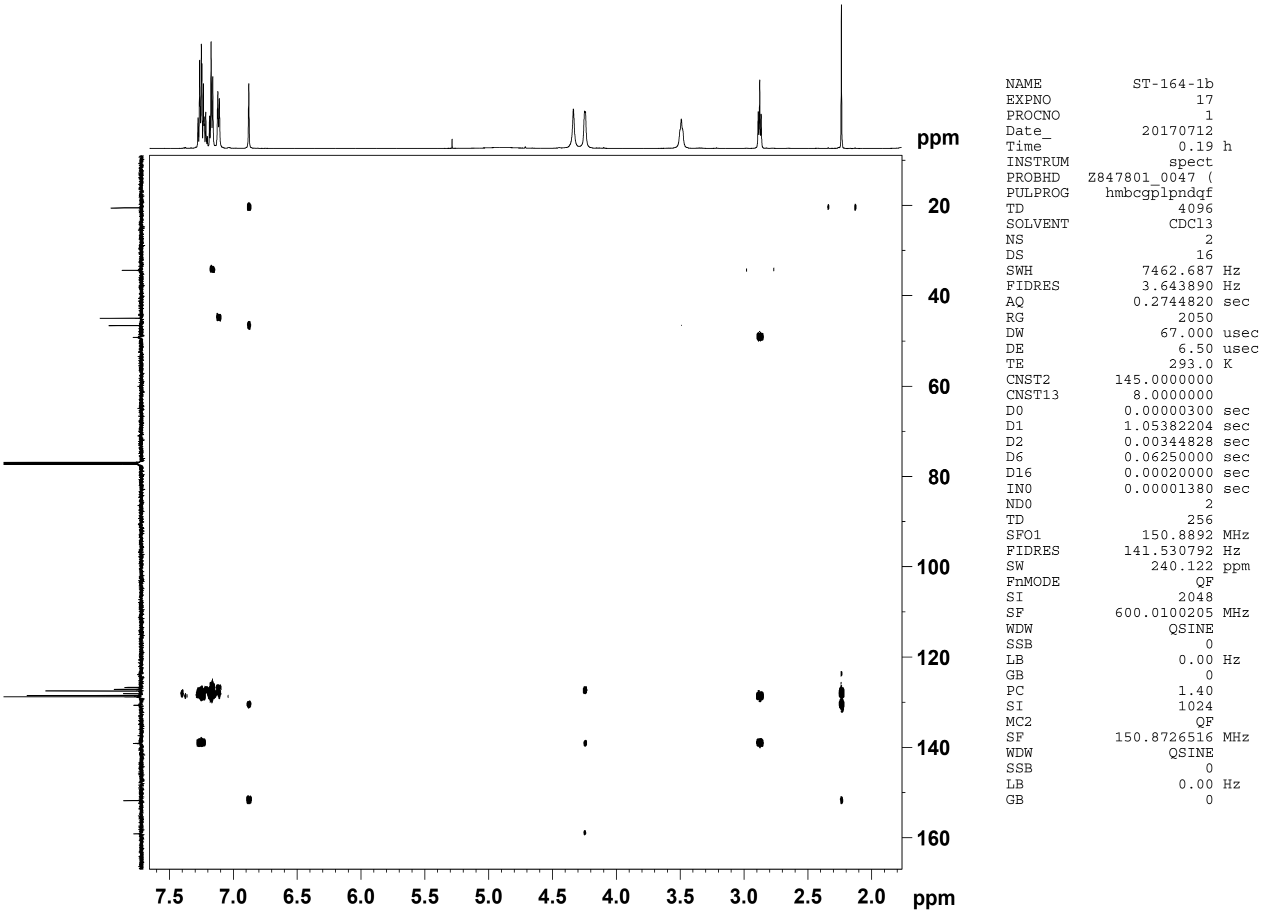

Figure S91. ^{1}H-^{13}C HMBC spectrum of compound **3cb**.

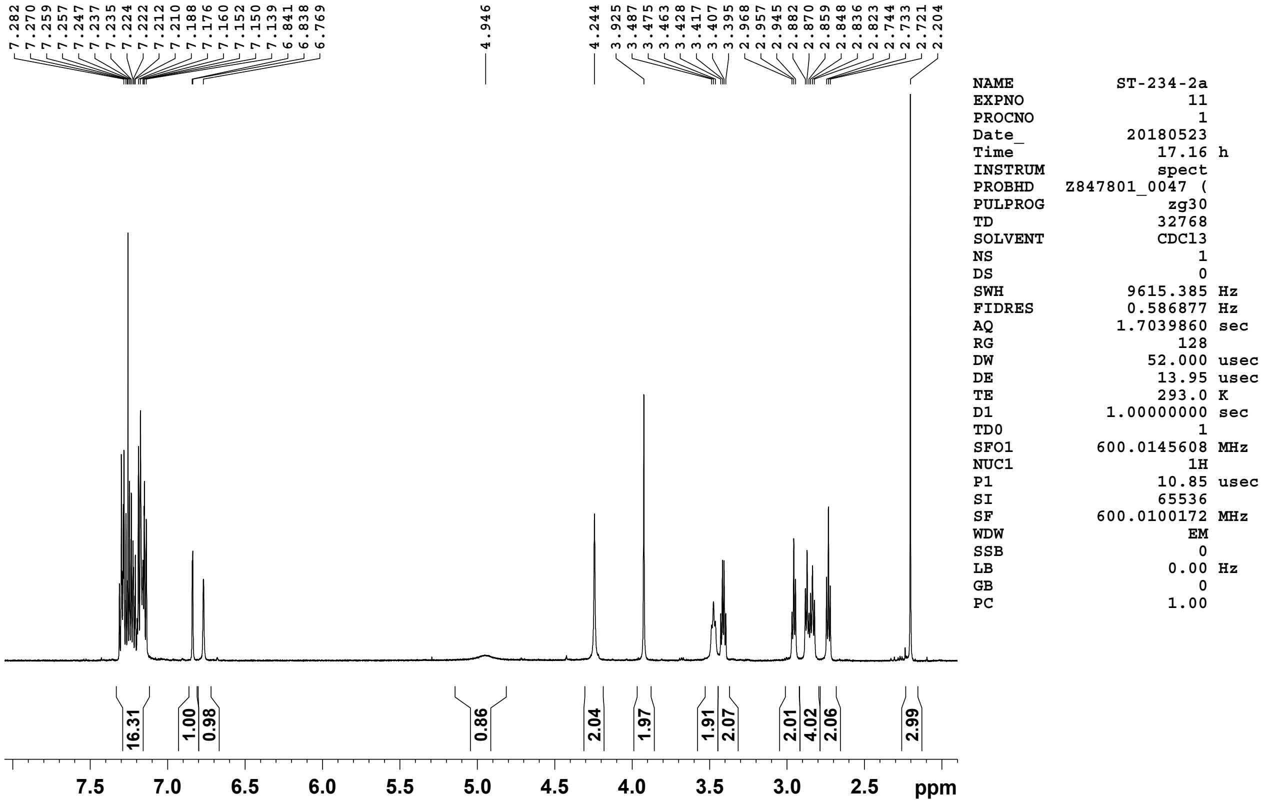

Figure S92. ^{1}H NMR spectrum of compound **2cc**.

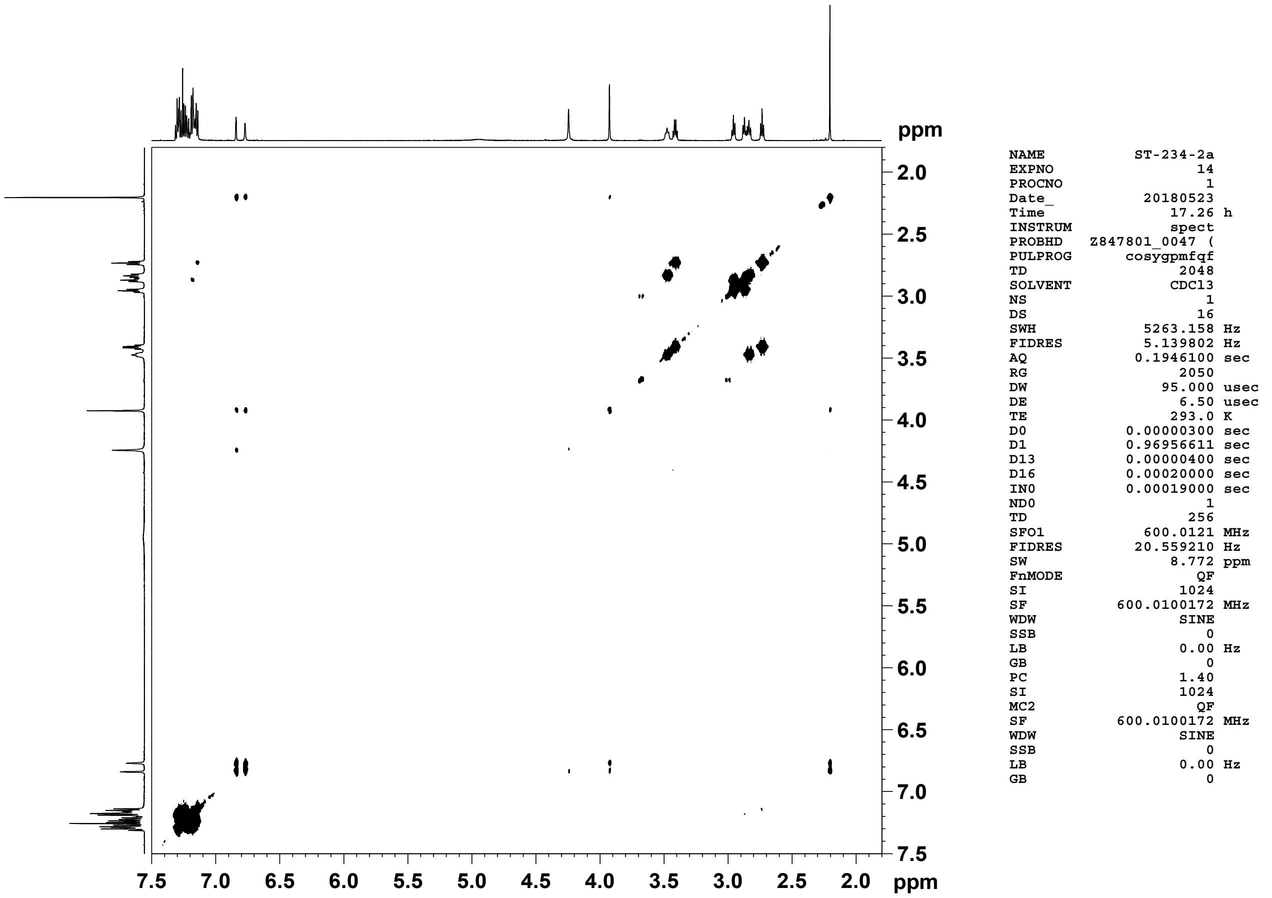

Figure S93. ^{1}H-^{1}H COSY spectrum of compound **2cc**.

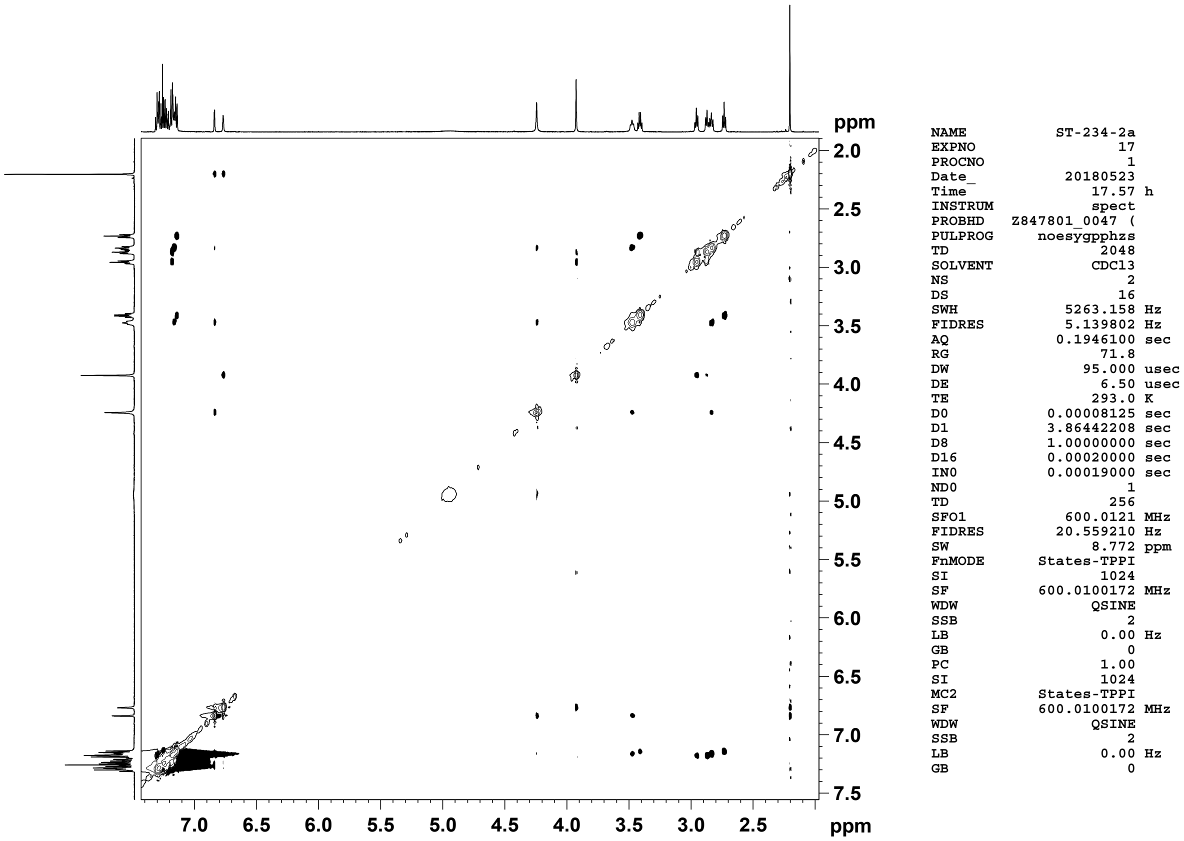

Figure S94. ^{1}H-^{1}H NOESY spectrum of compound **2cc**.

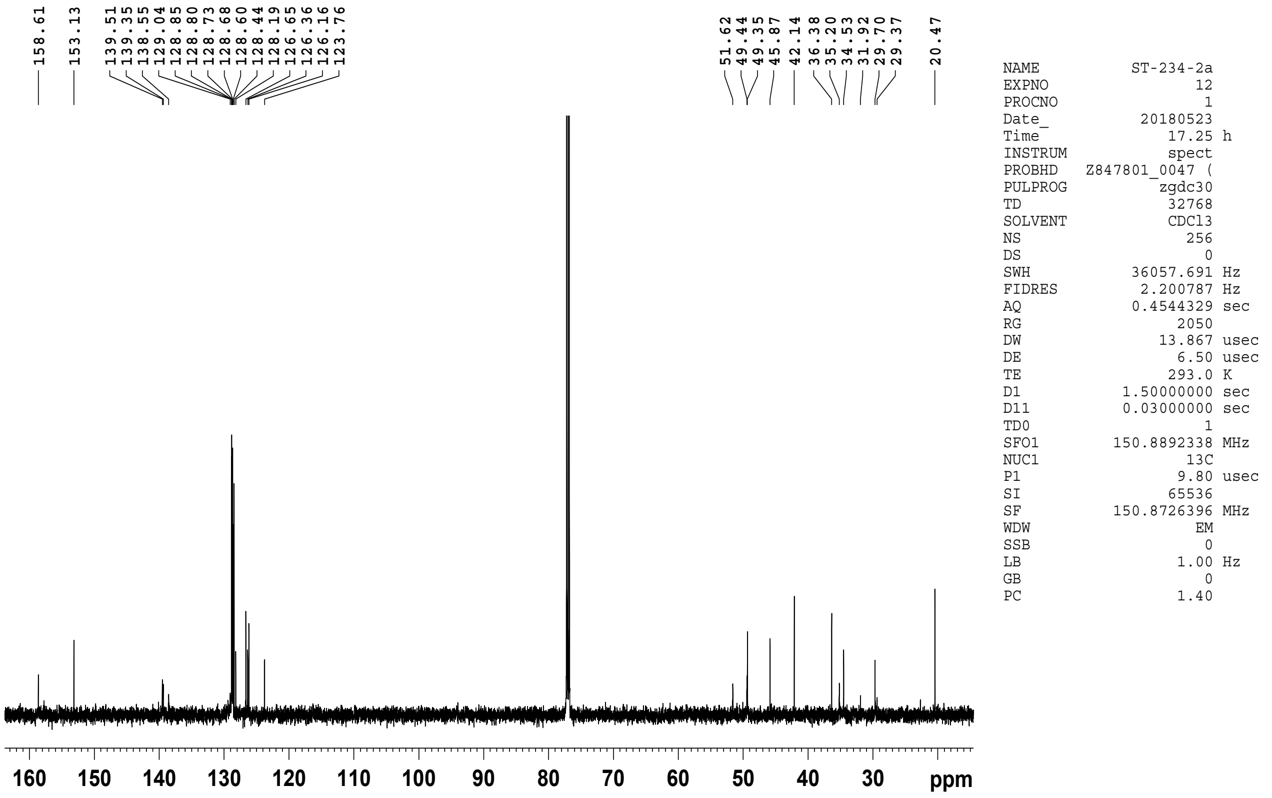

Figure S95. ^{13}C NMR spectrum of compound **2cc**.

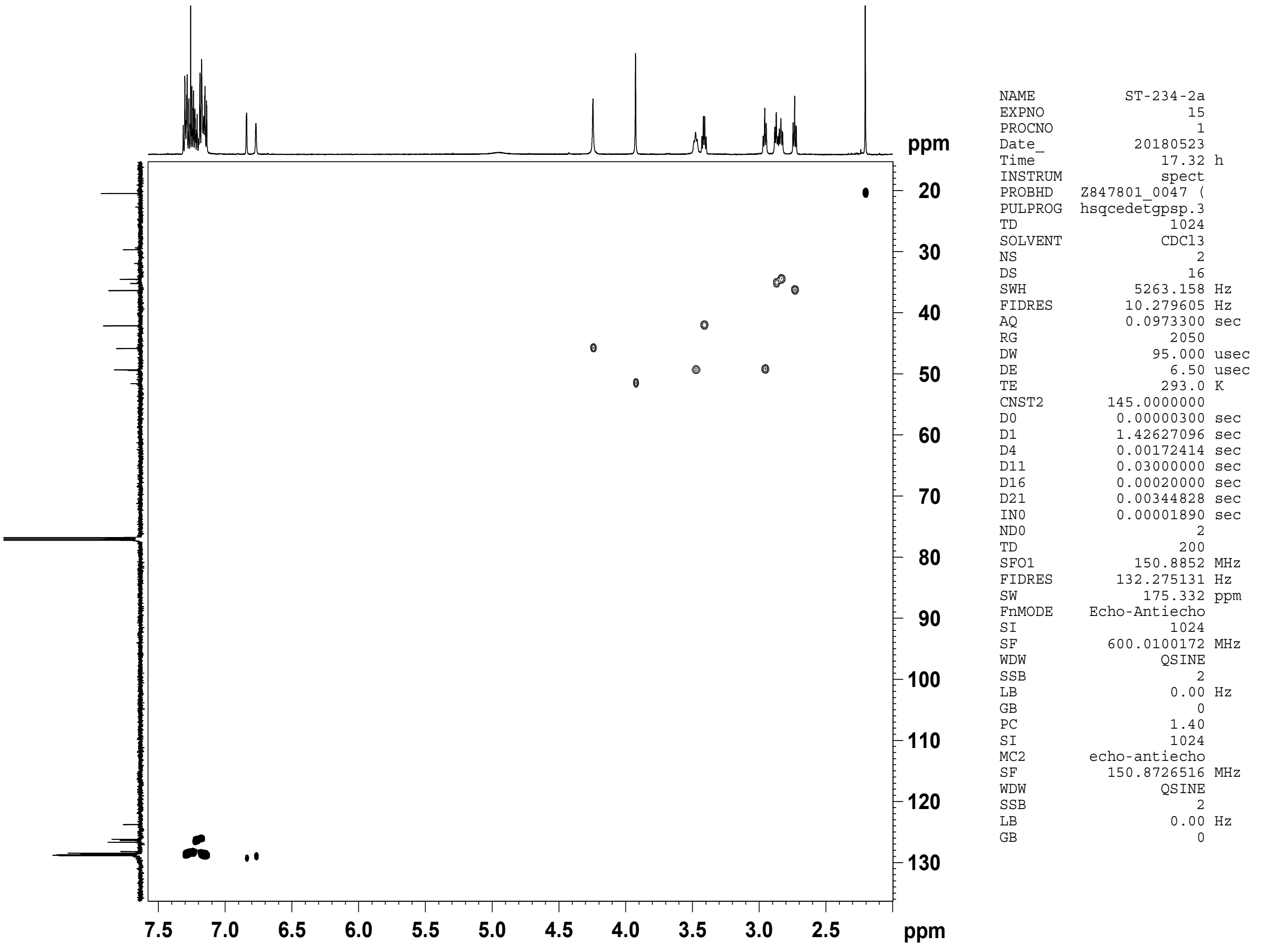

Figure S96. ^{1}H-^{13}C HSQC spectrum of compound **2cc**.

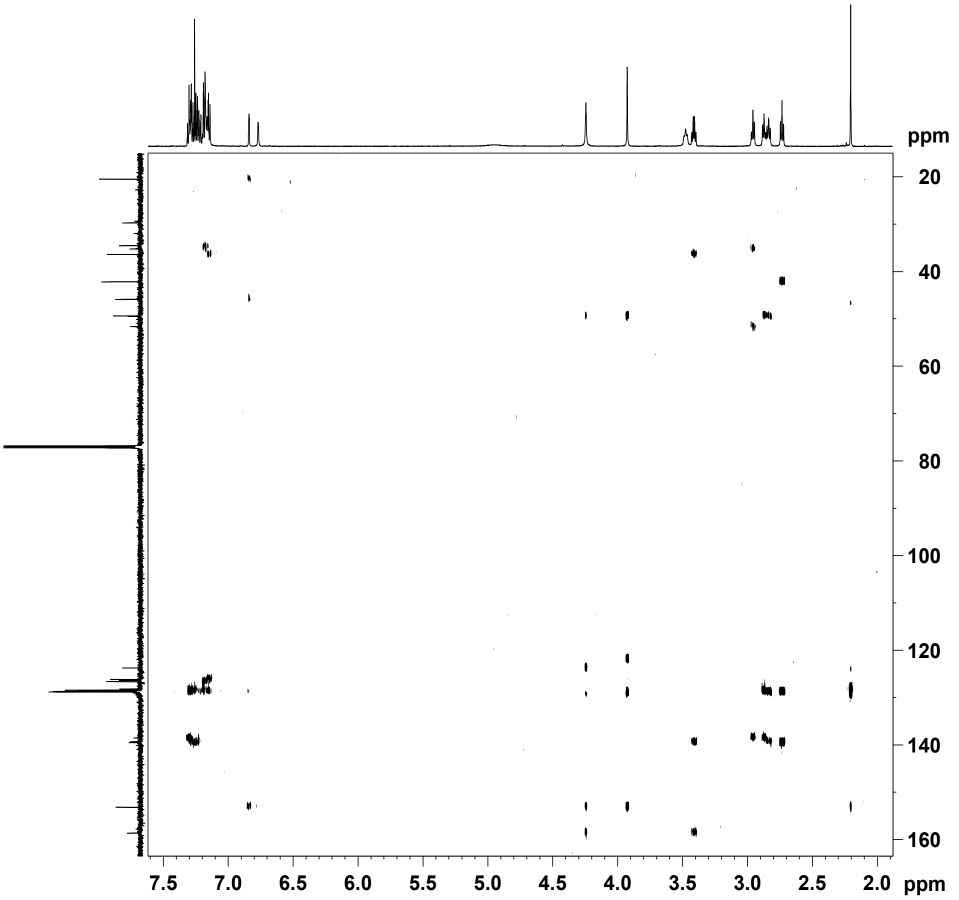

Figure S97. ^{1}H-^{13}C HMBC spectrum of compound **2cc**.

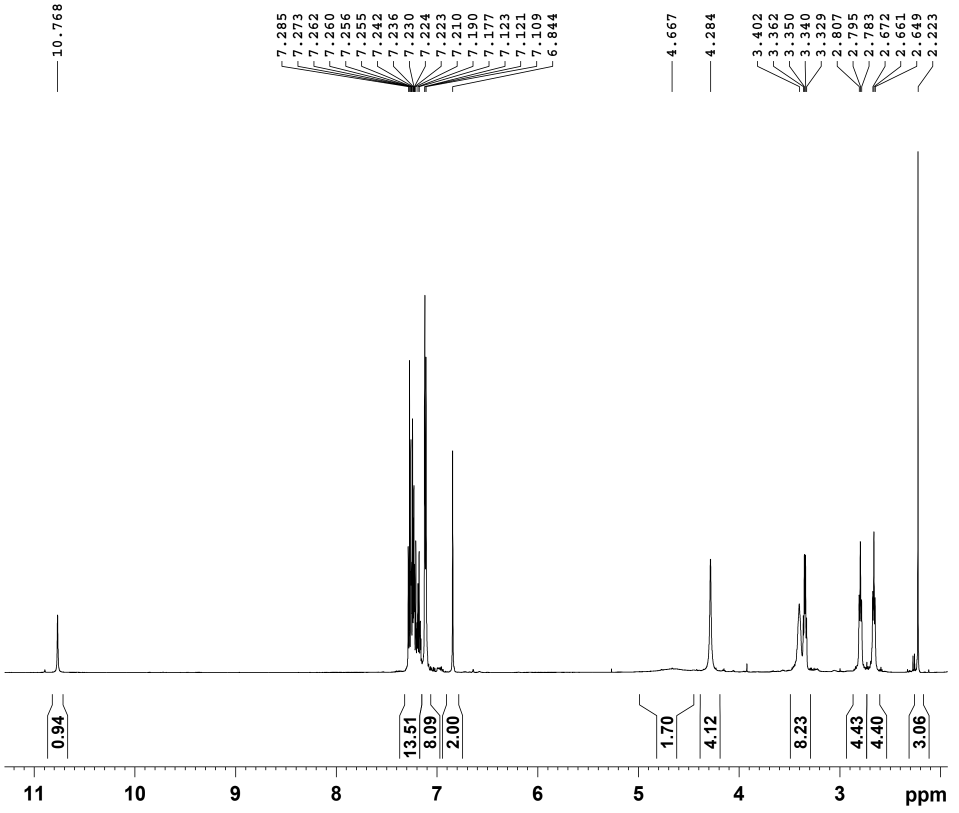

Figure S98. ^{1}H NMR spectrum of compound **3cc**.

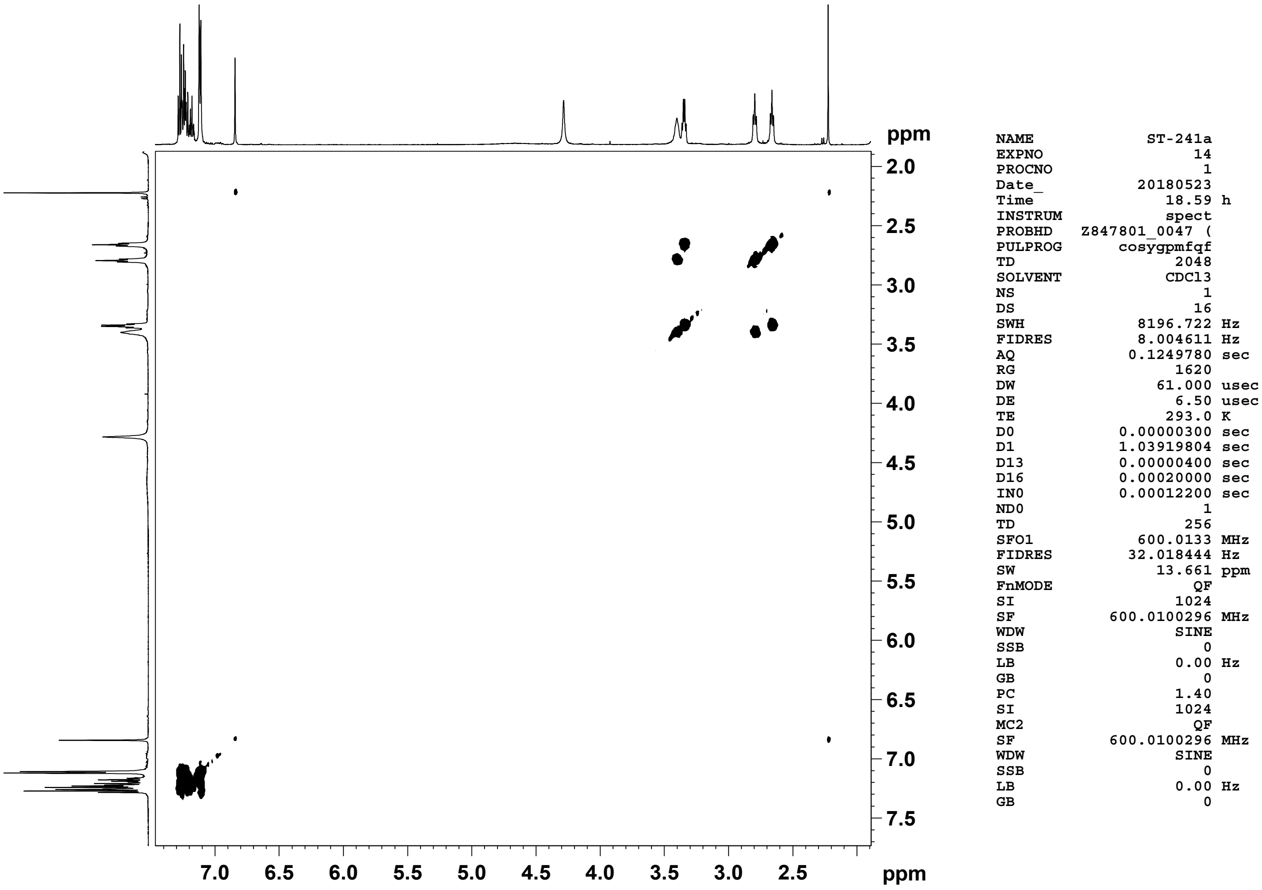

Figure S99. ^{1}H-^{1}H COSY spectrum of compound **3cc**.

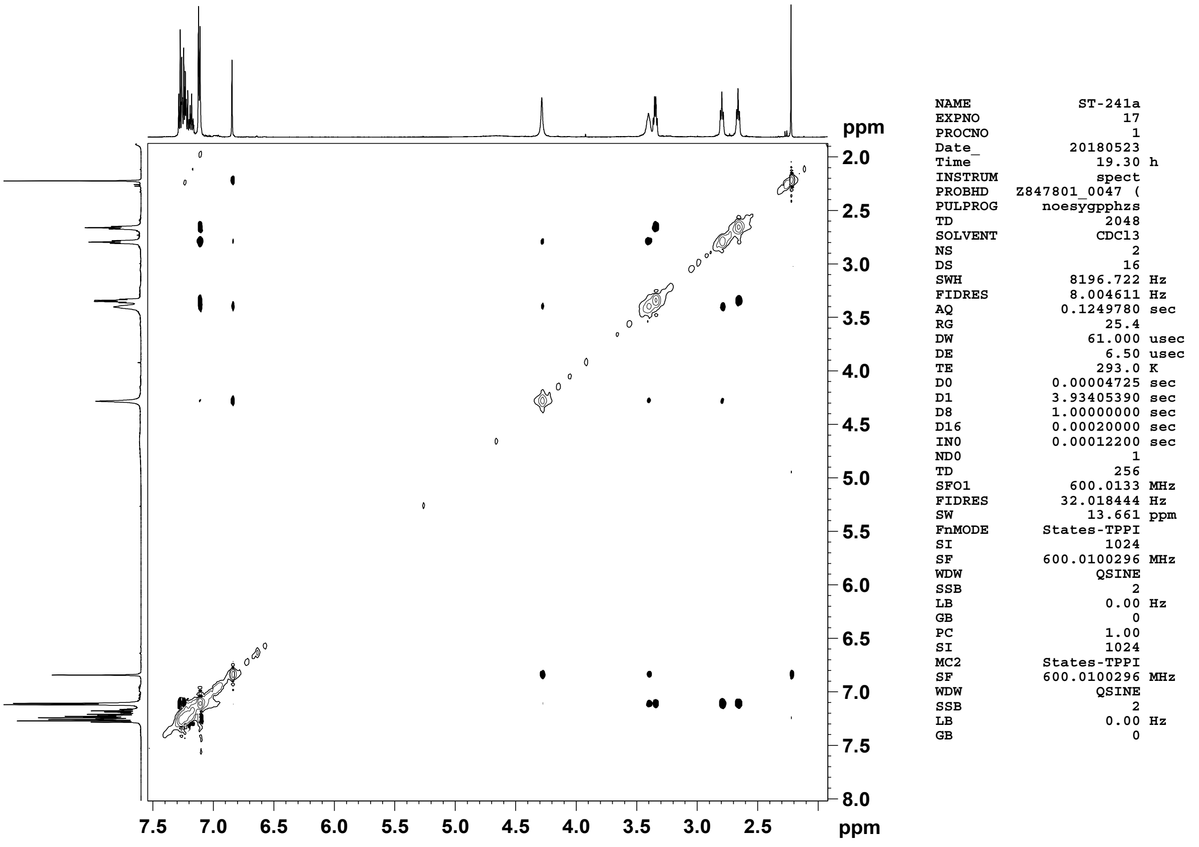

Figure S100. ^{1}H-^{1}H NOESY spectrum of compound **3cc**.

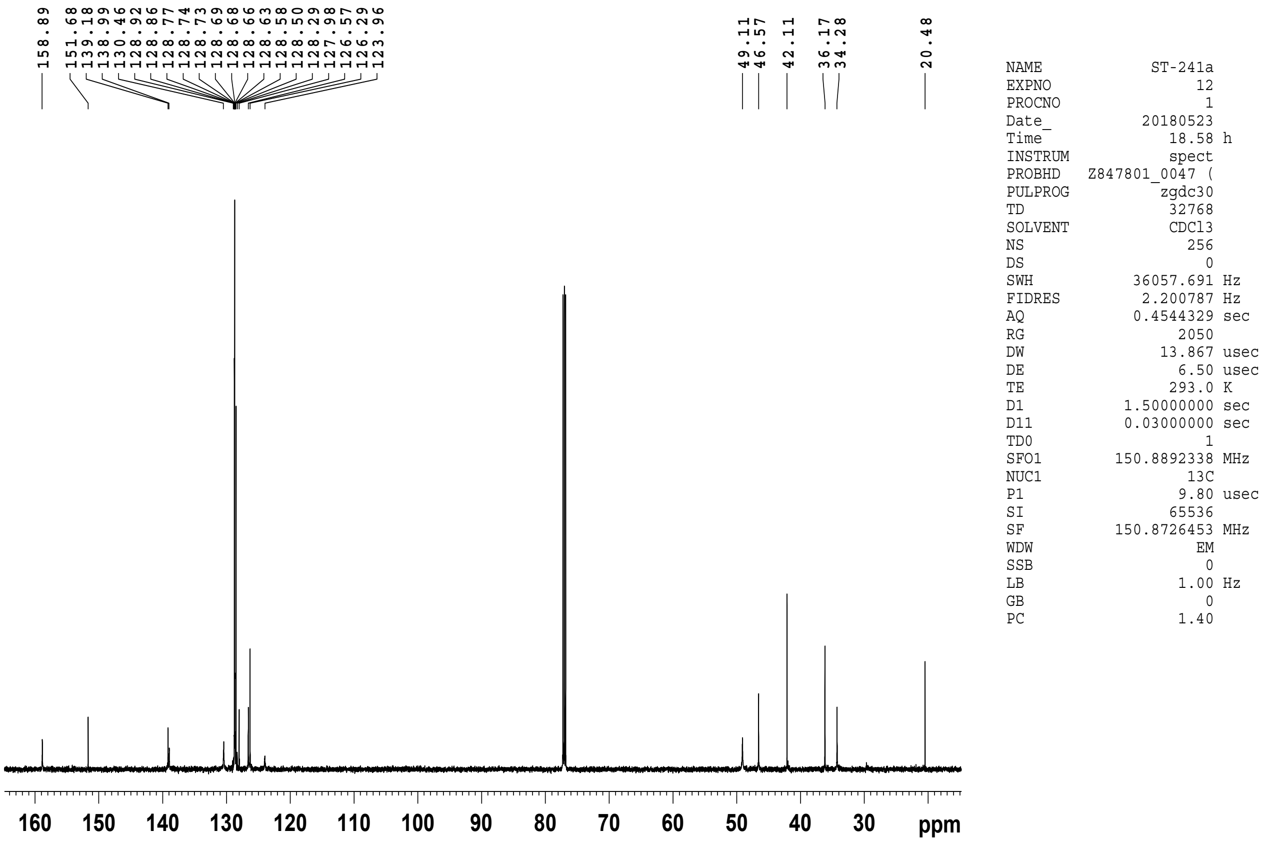

Figure S101. ^{13}C NMR spectrum of compound **3cc**.

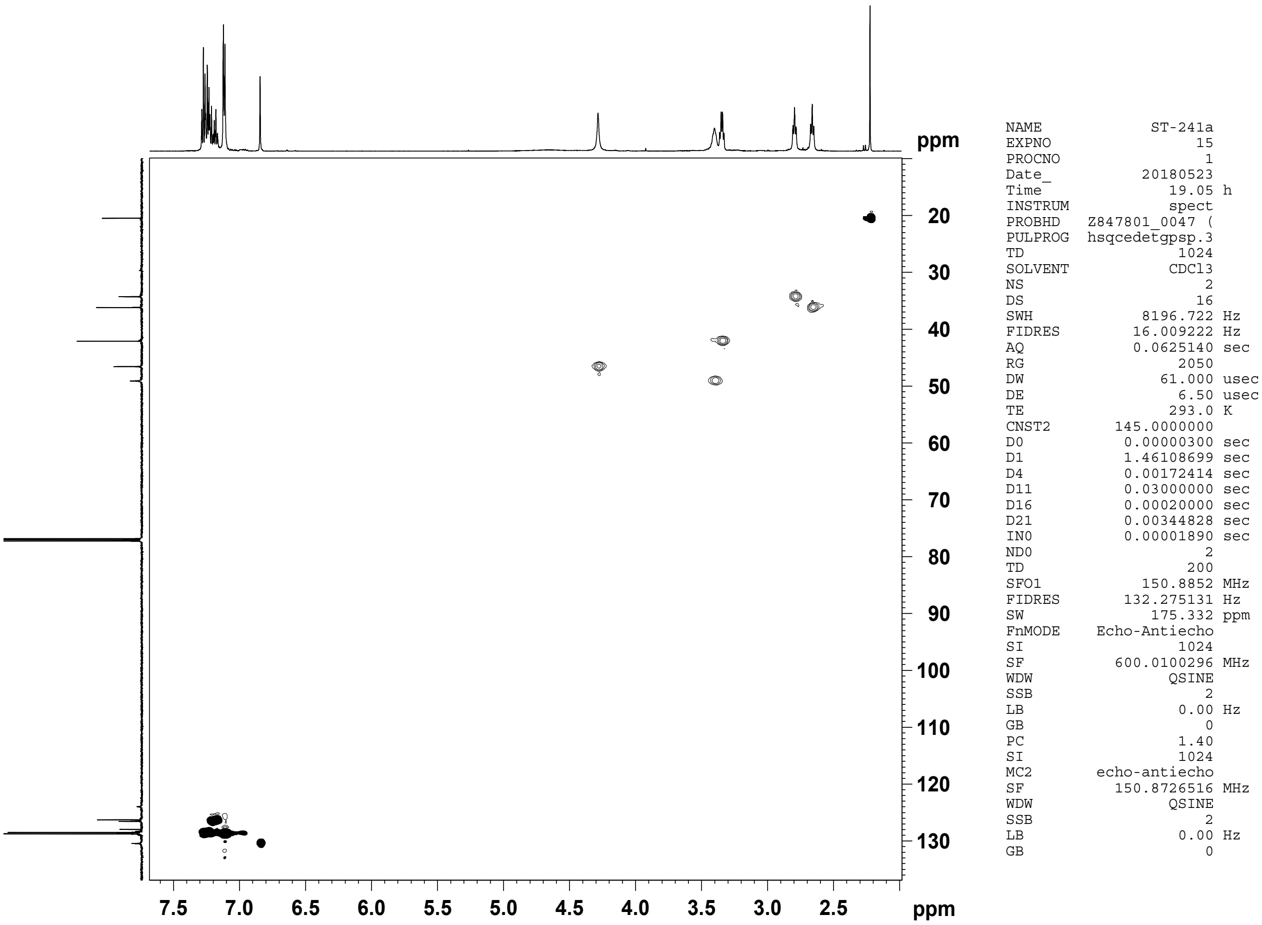

Figure S102. ^{1}H-^{13}C HSQC spectrum of compound **3cc**.

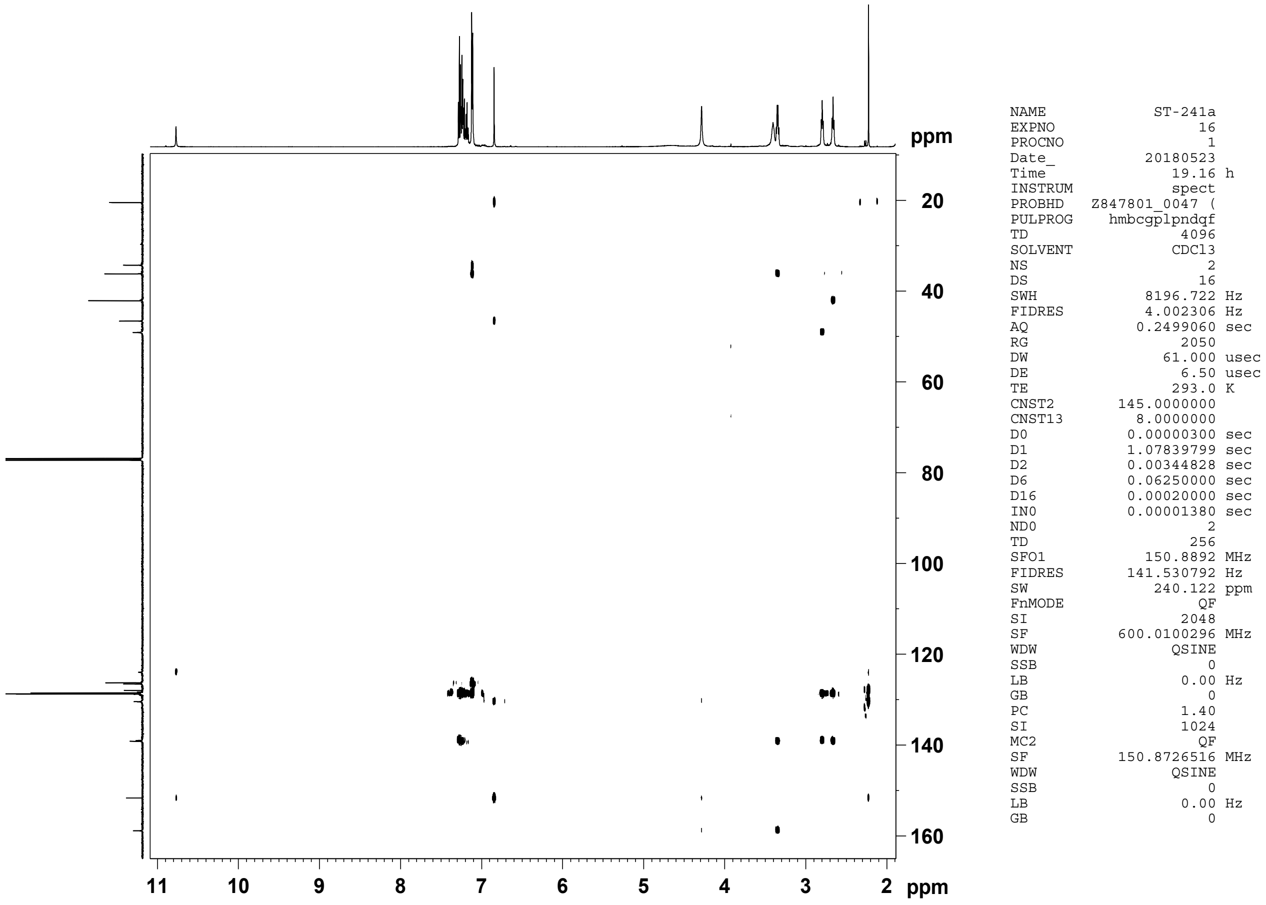

Figure S103. ^{1}H-^{13}C HMBC spectrum of compound **3cc**.

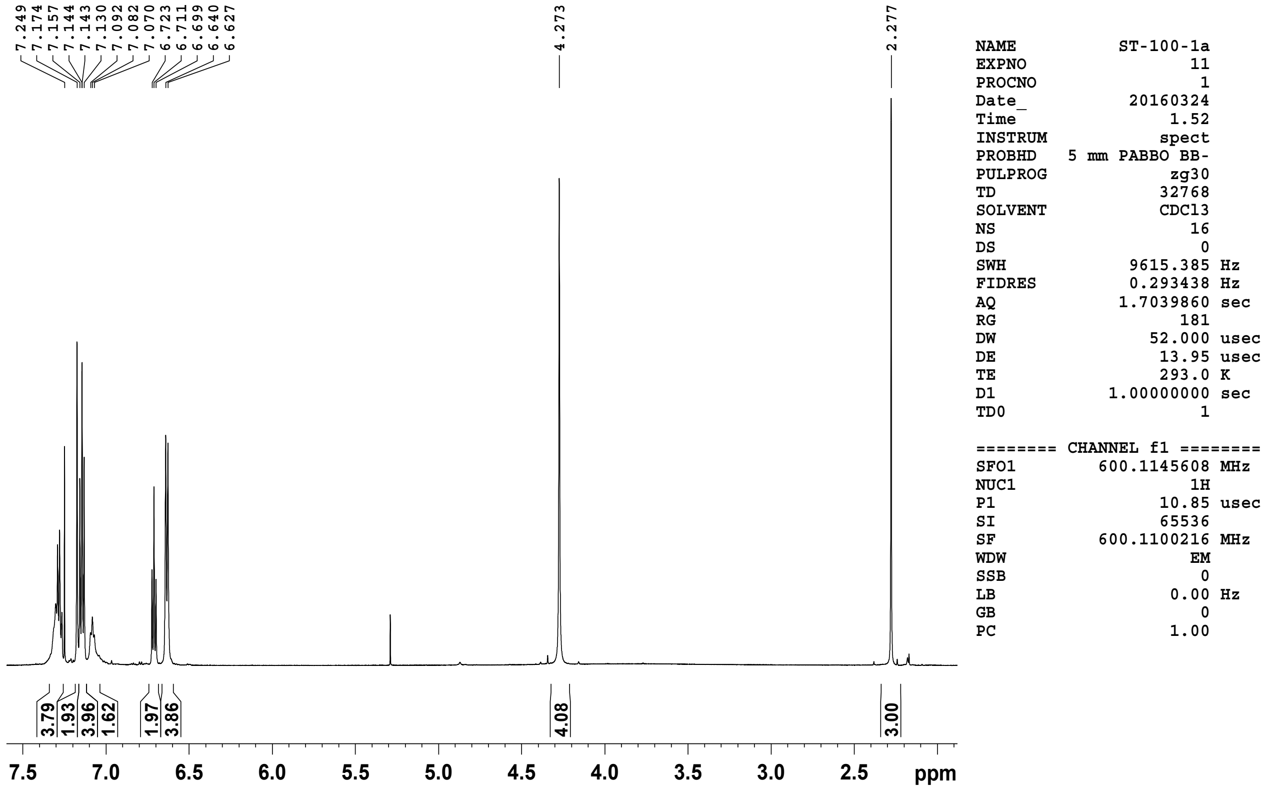

Figure S104. ^{1}H NMR spectrum of compound **4**.

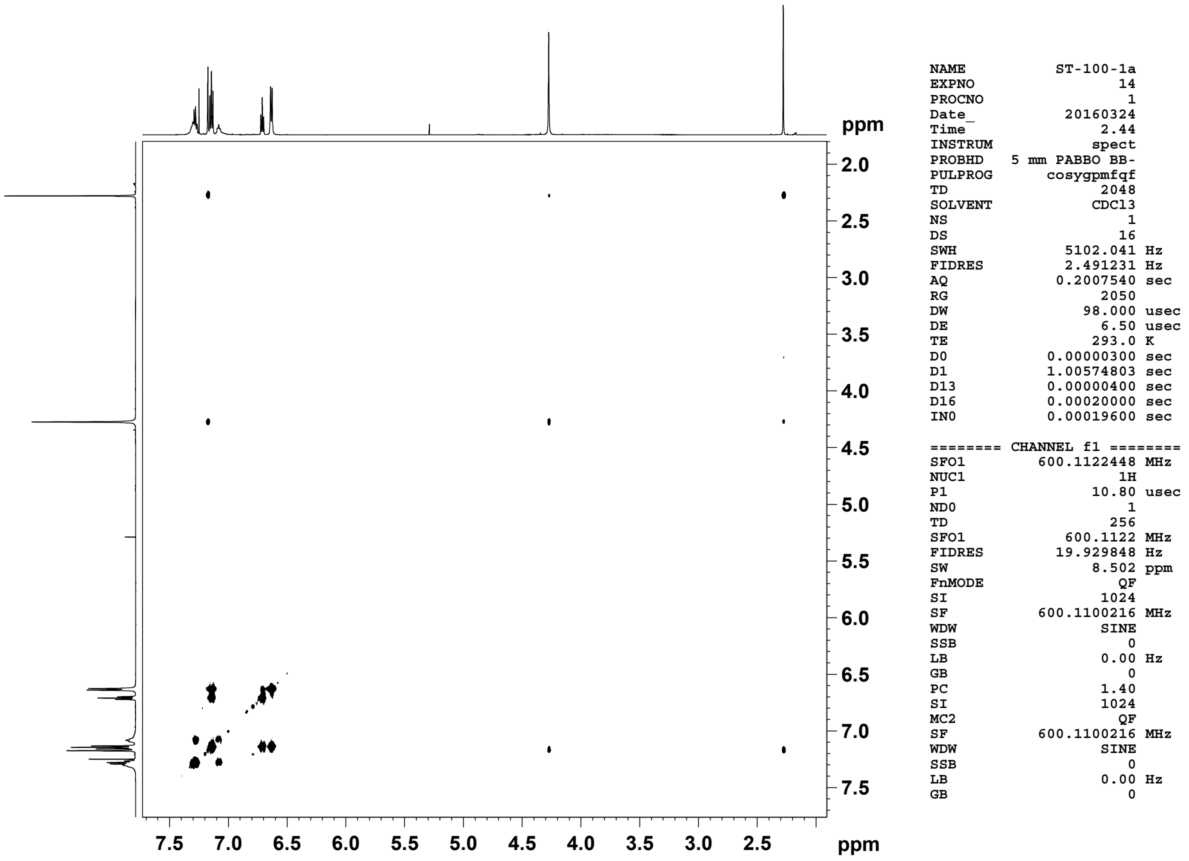

Figure S105. ^{1}H-^{1}H COSY spectrum of compound **4**.

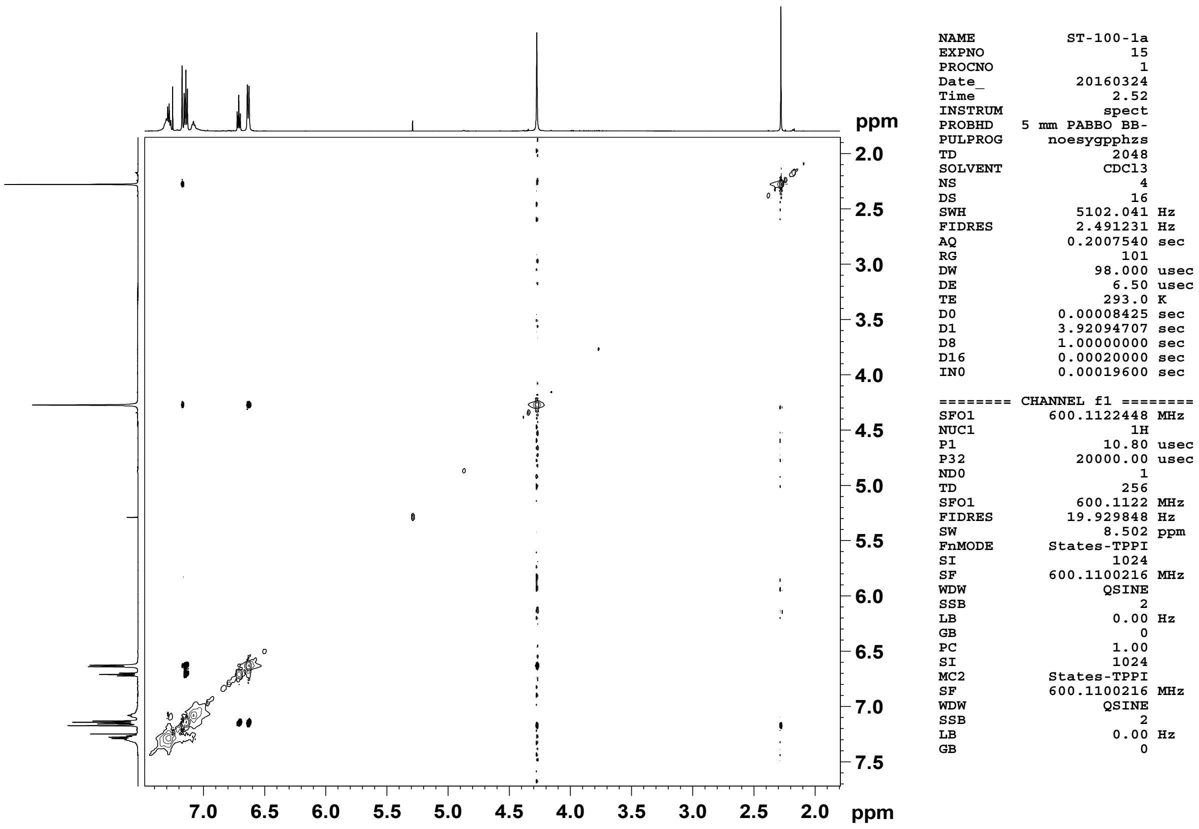

Figure S106. ^{1}H-^{1}H NOESY spectrum of compound **4**.

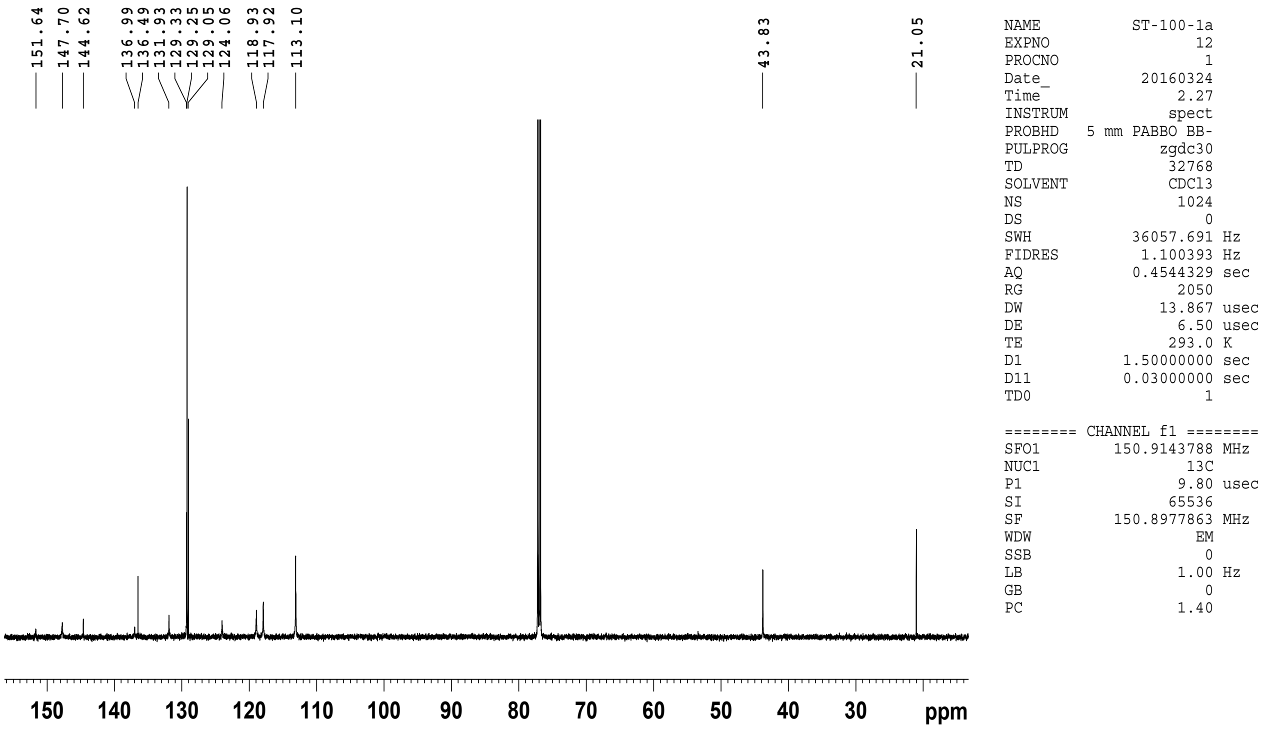

Figure S107. ^{13}C NMR spectrum of compound **4**.

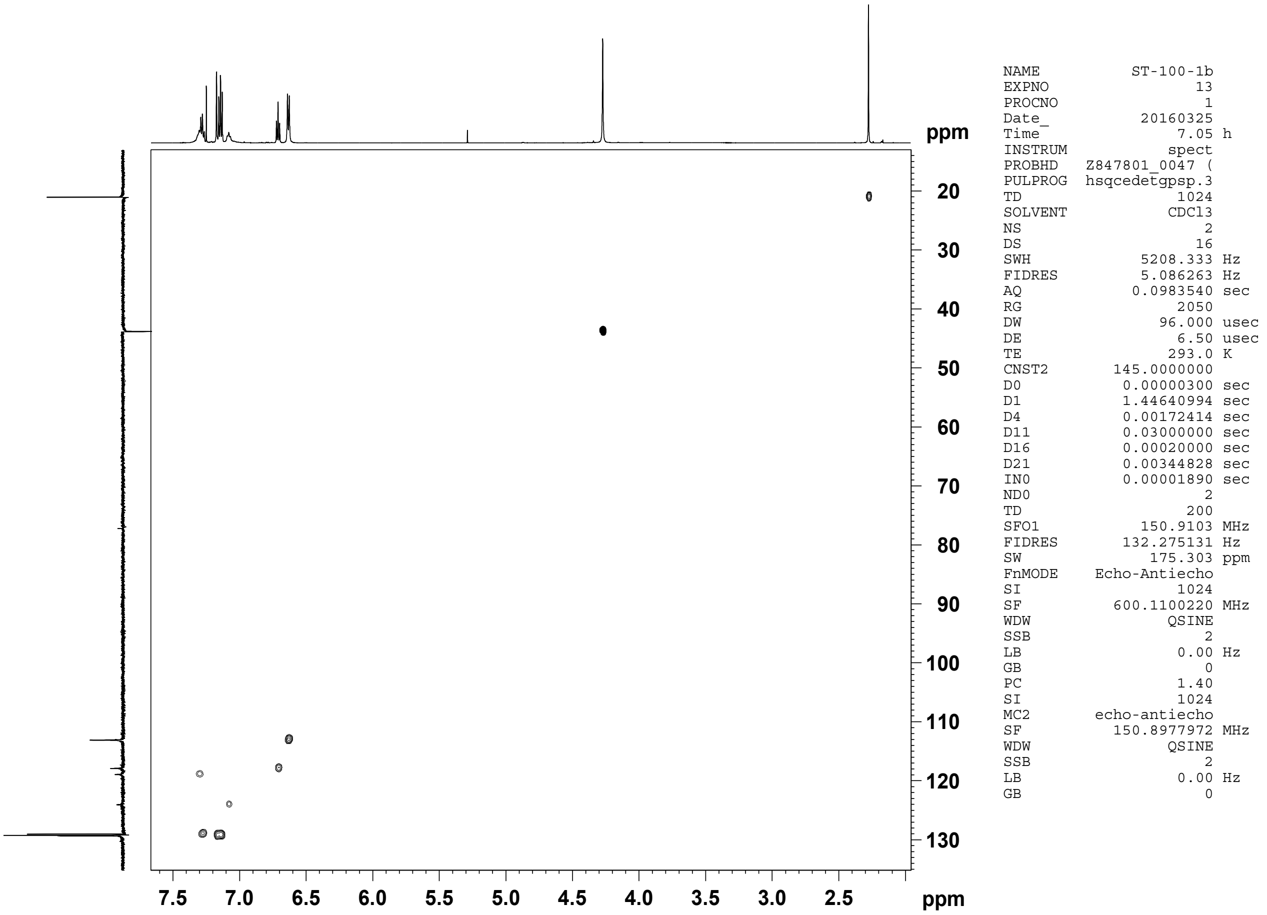

Figure S108. ^{1}H-^{13}C HSQC spectrum of compound **4**.

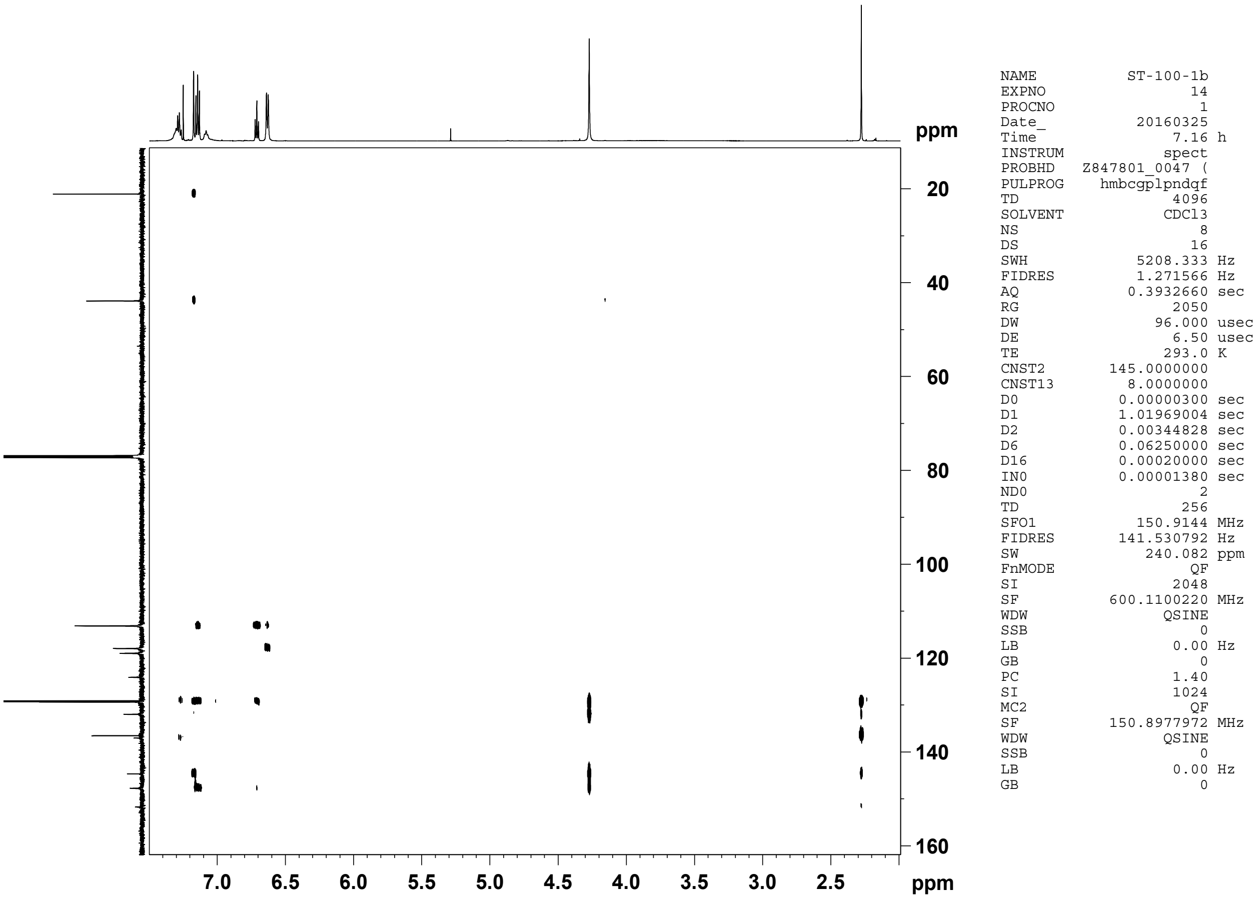

Figure S109. ^{1}H-^{13}C HMBC spectrum of compound **4**.

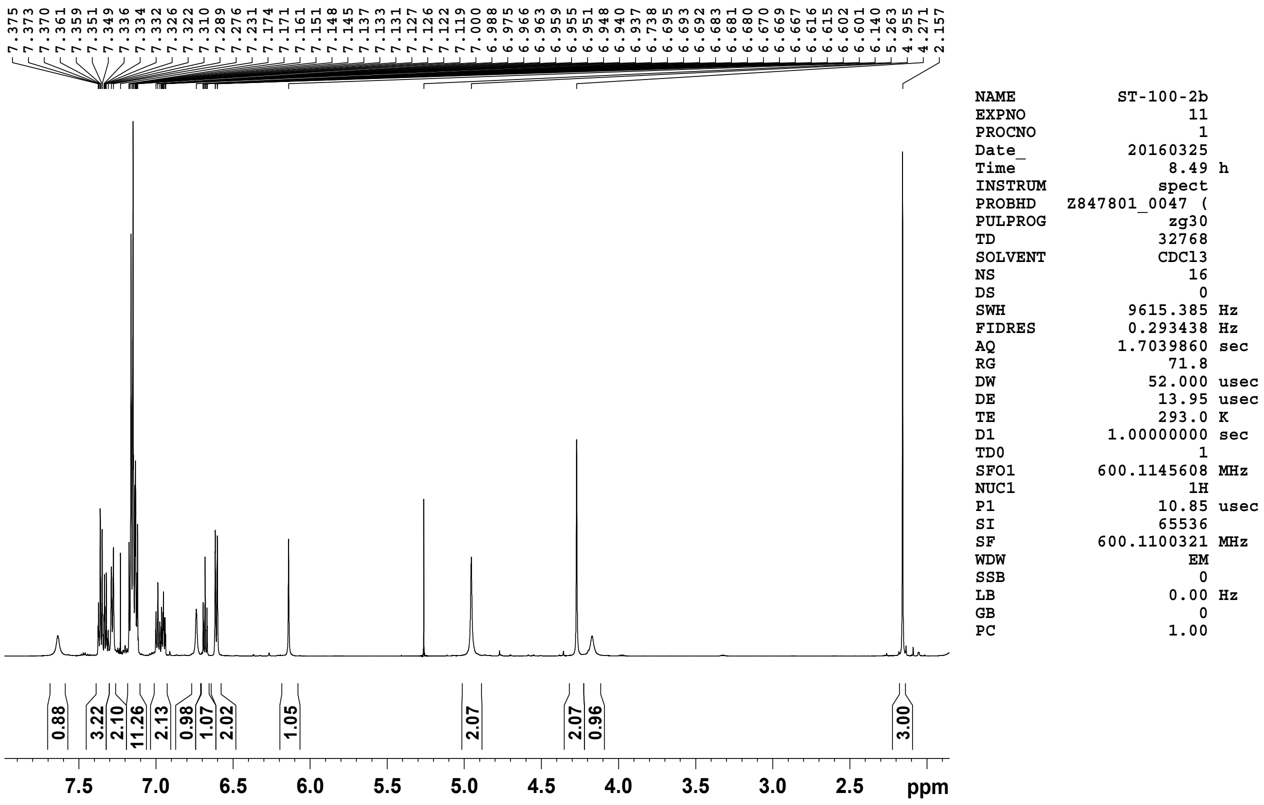

Figure S110. ^{1}H NMR spectrum of compound **5**.

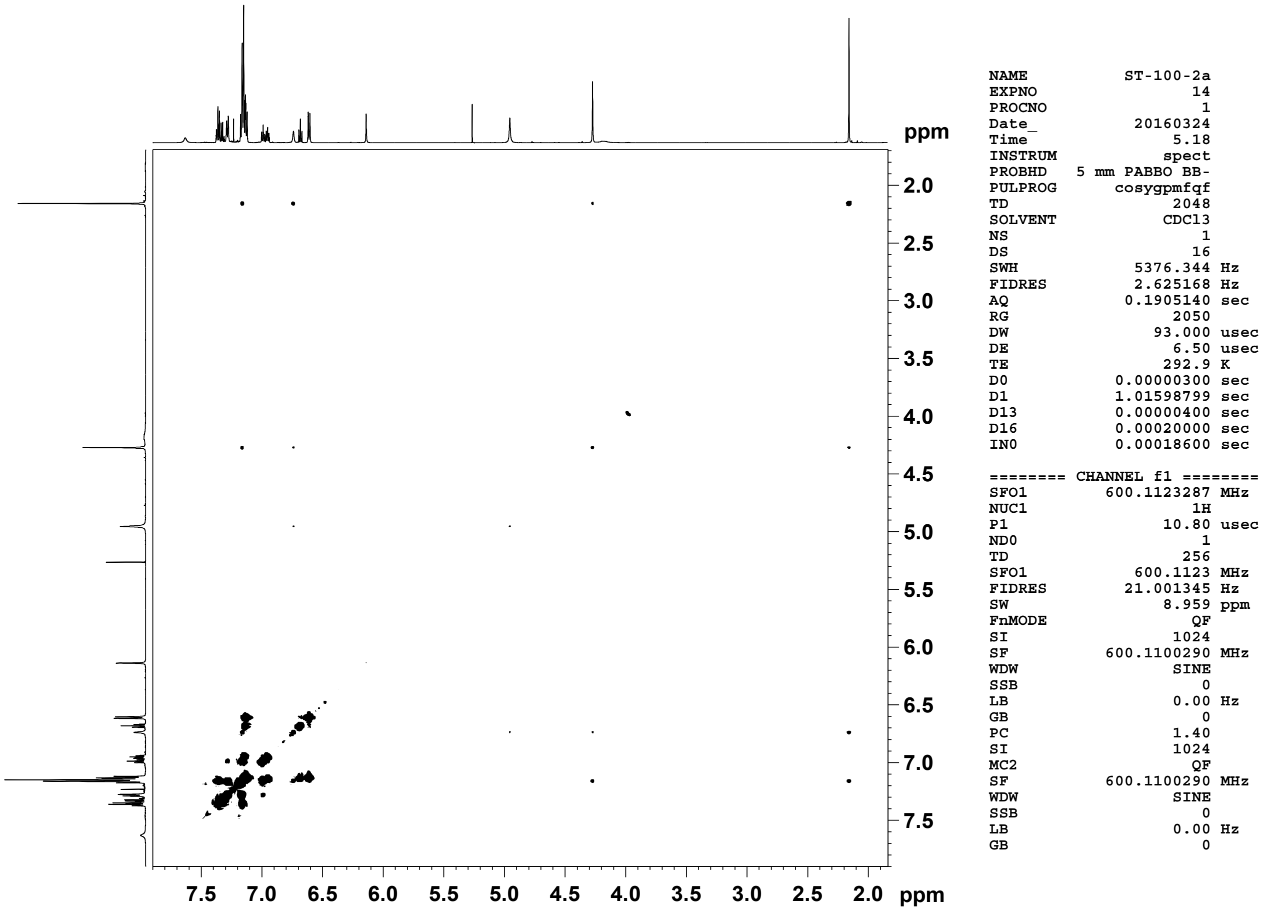

Figure S111. ^{1}H-^{1}H COSY spectrum of compound **5**.

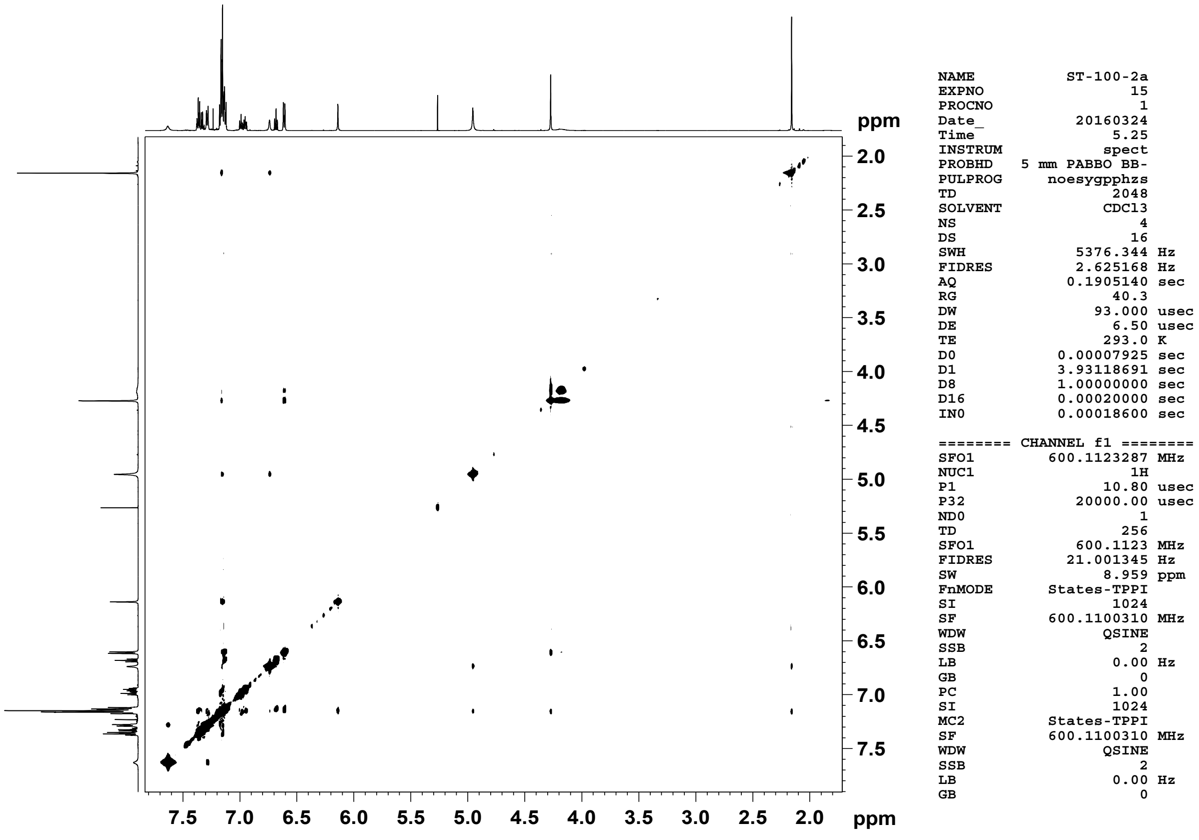

Figure S112. ^{1}H-^{1}H NOESY spectrum of compound **5**.

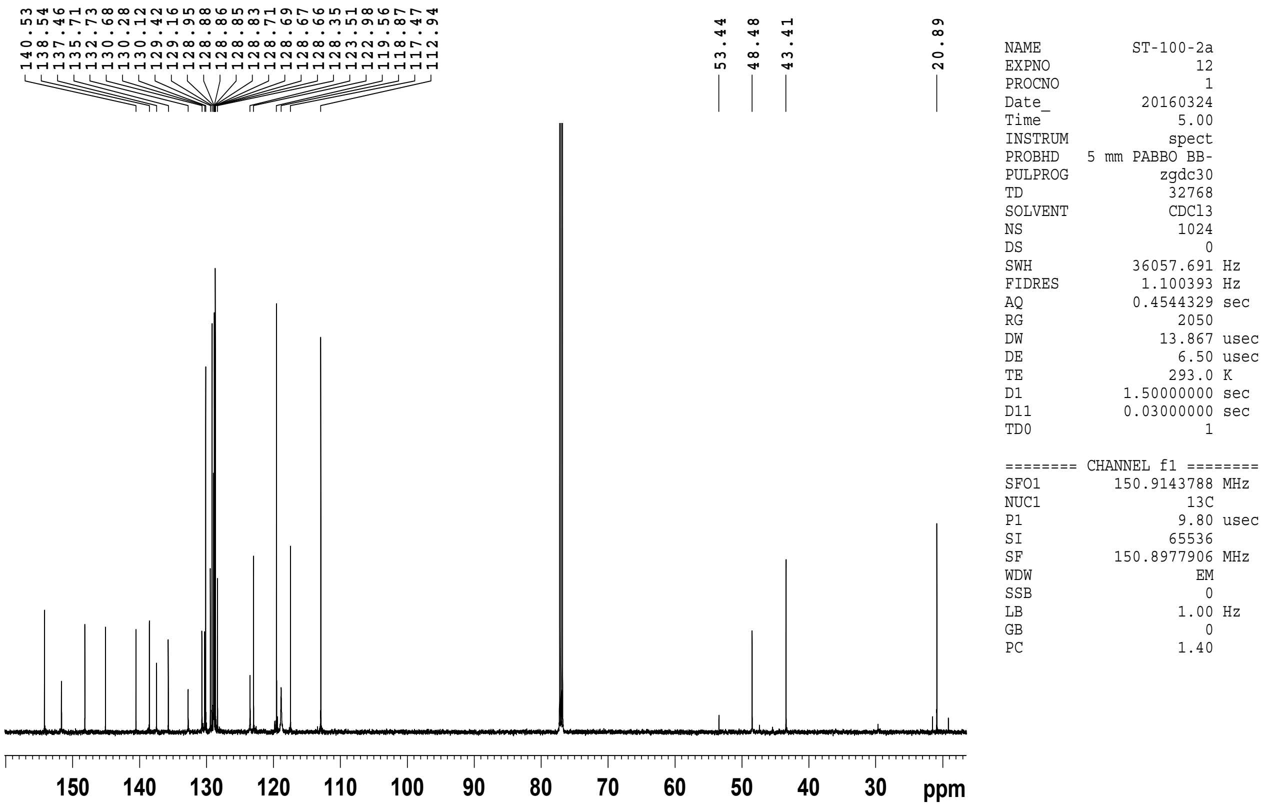

Figure S113. ^{13}C NMR spectrum of compound **5**.

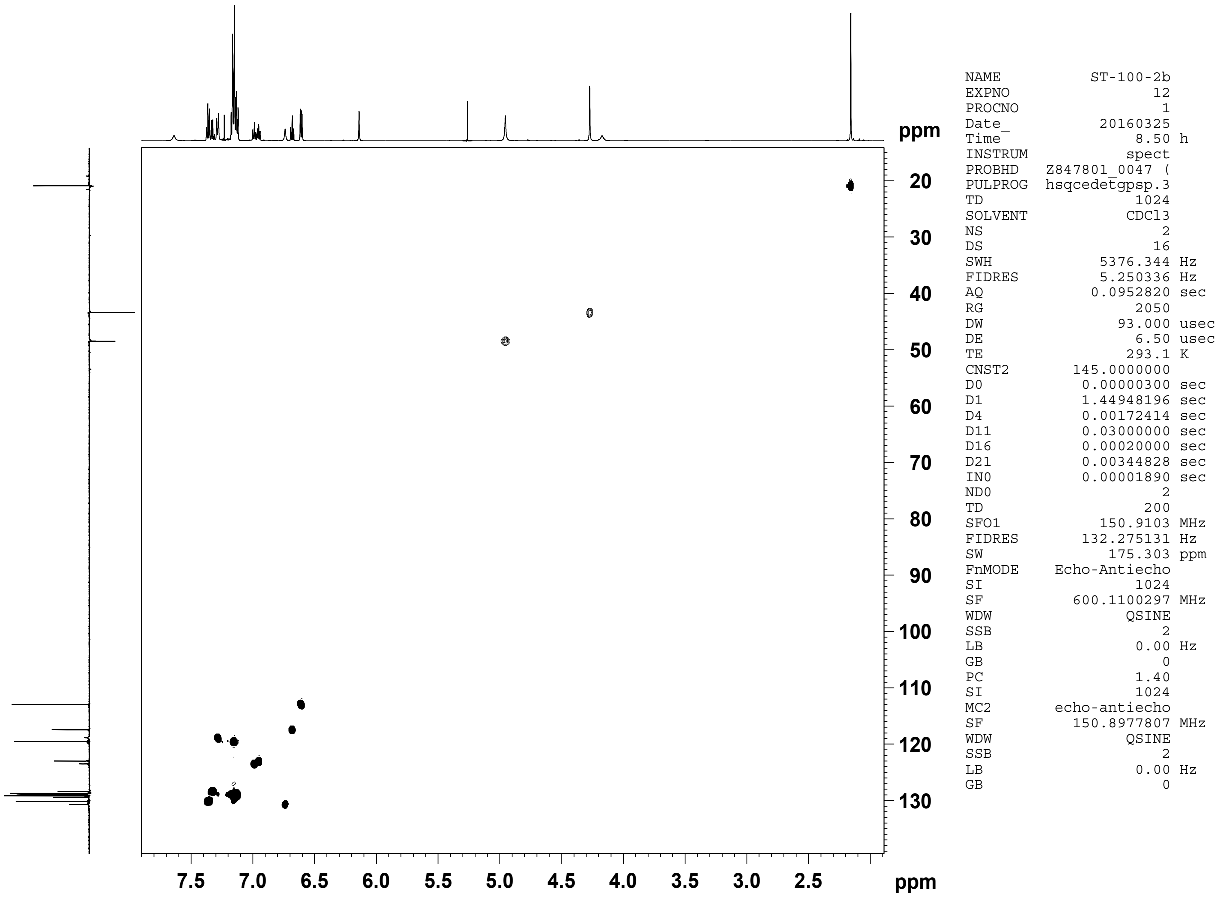

Figure S114. ^{1}H-^{13}C HSQC spectrum of compound **5**.

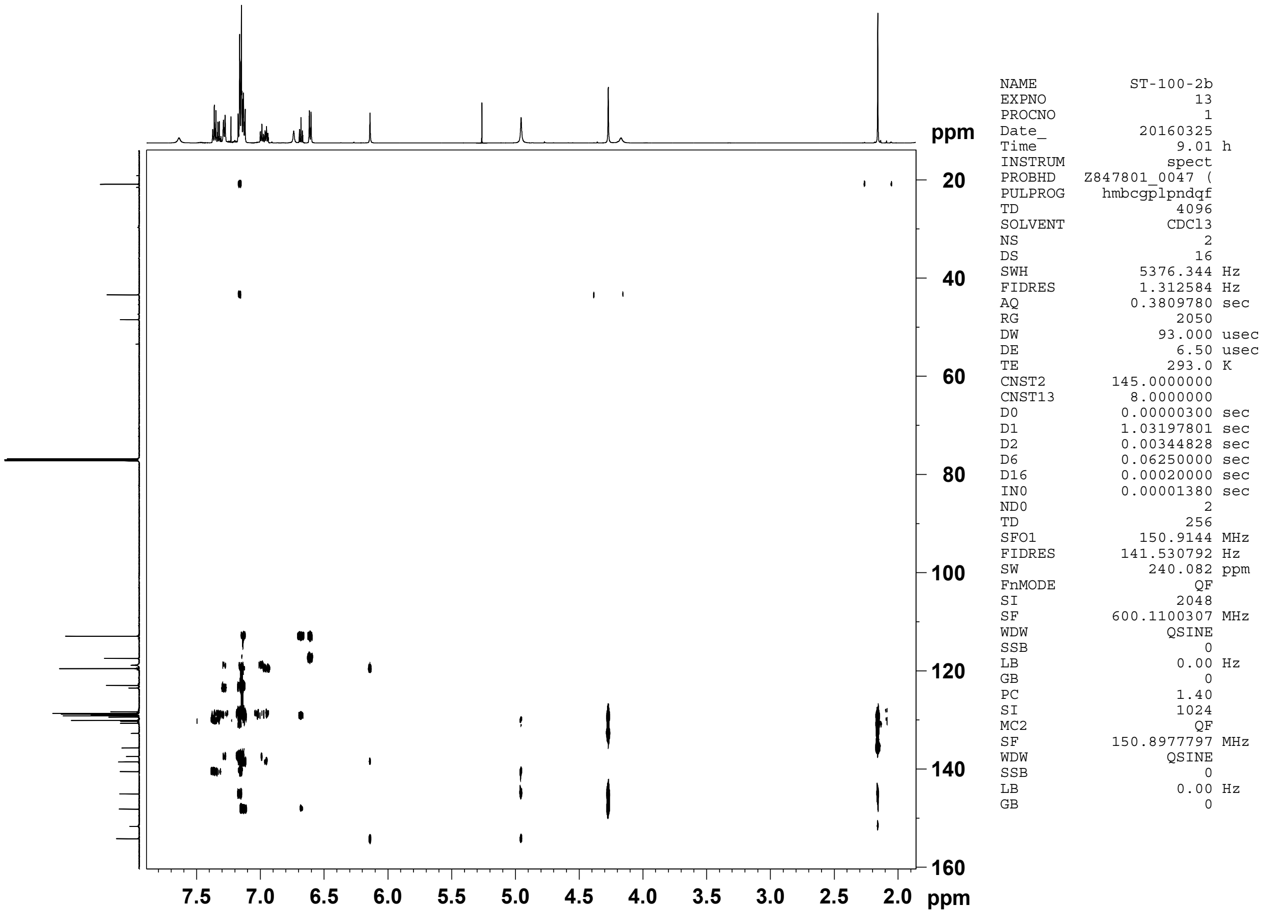

Figure S115. ^{1}H-^{13}C HMBC spectrum of compound **5**.

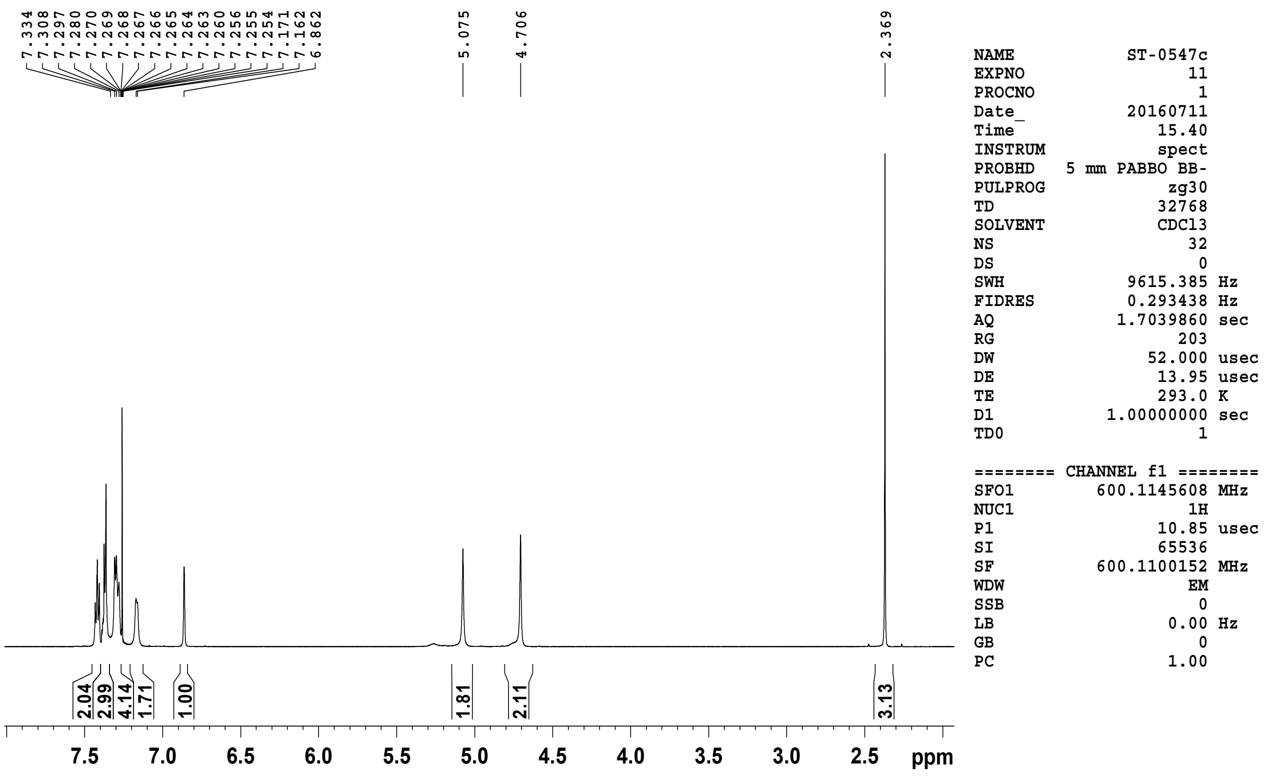

Figure S116. ^{1}H NMR spectrum of compound **6a**.

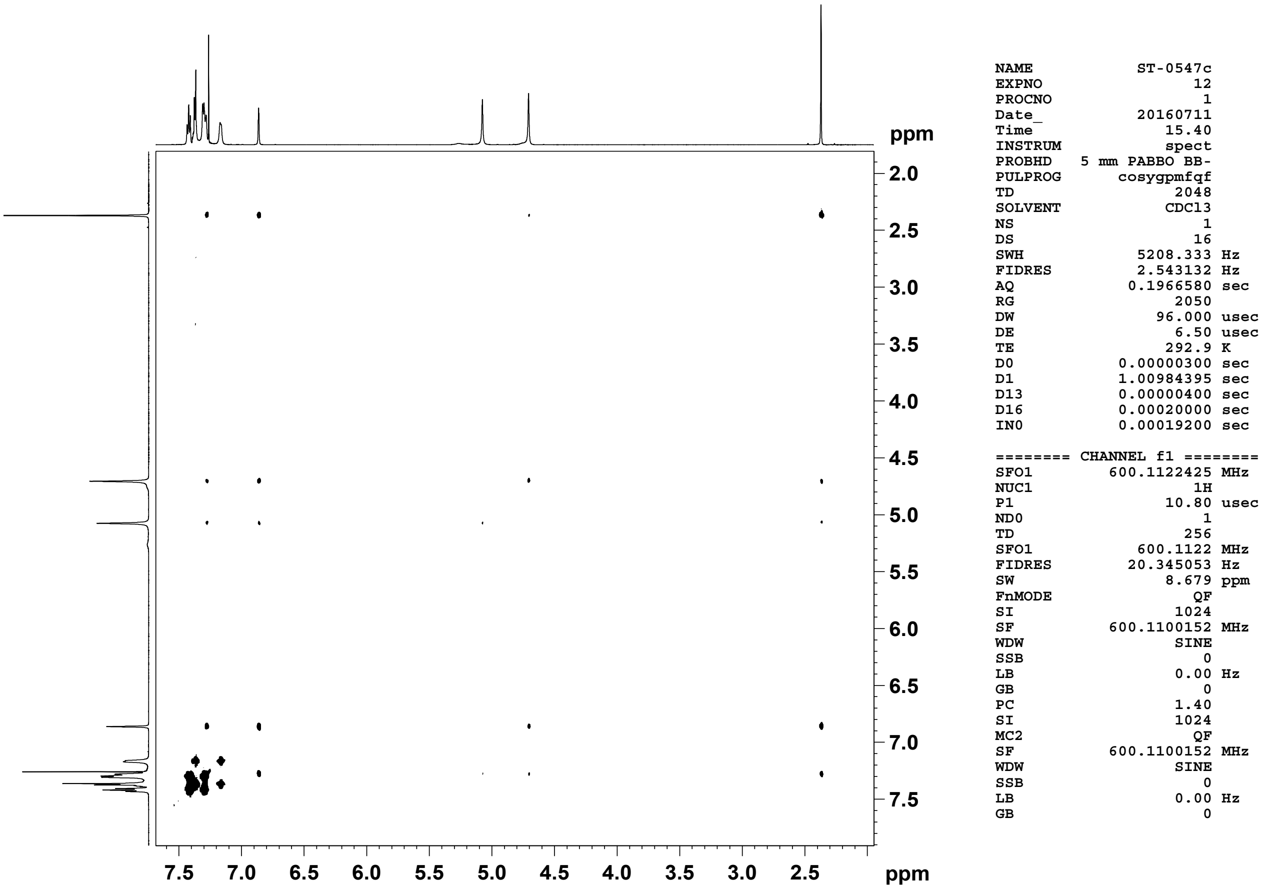

Figure S117. ^{1}H-^{1}H COSY spectrum of compound **6a**.

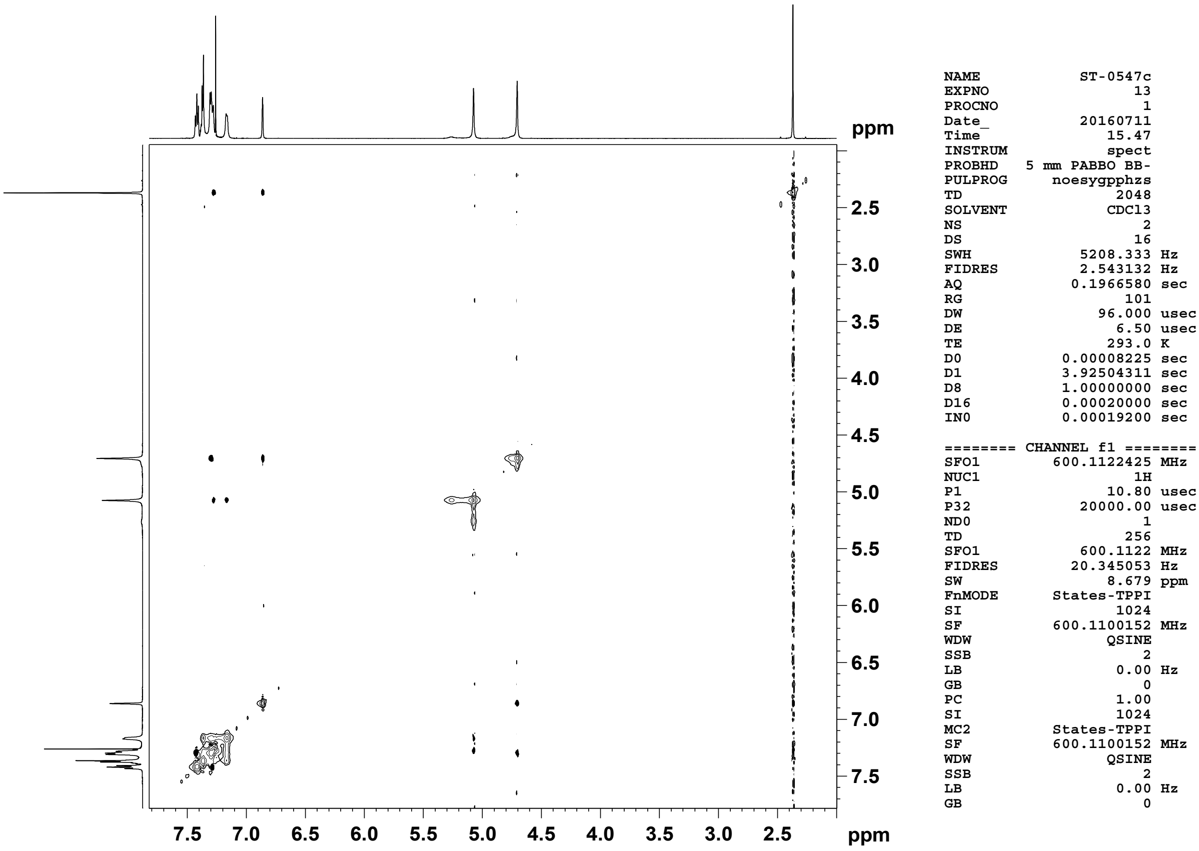

Figure S118. ^{1}H-^{1}H NOESY spectrum of compound **6a**.

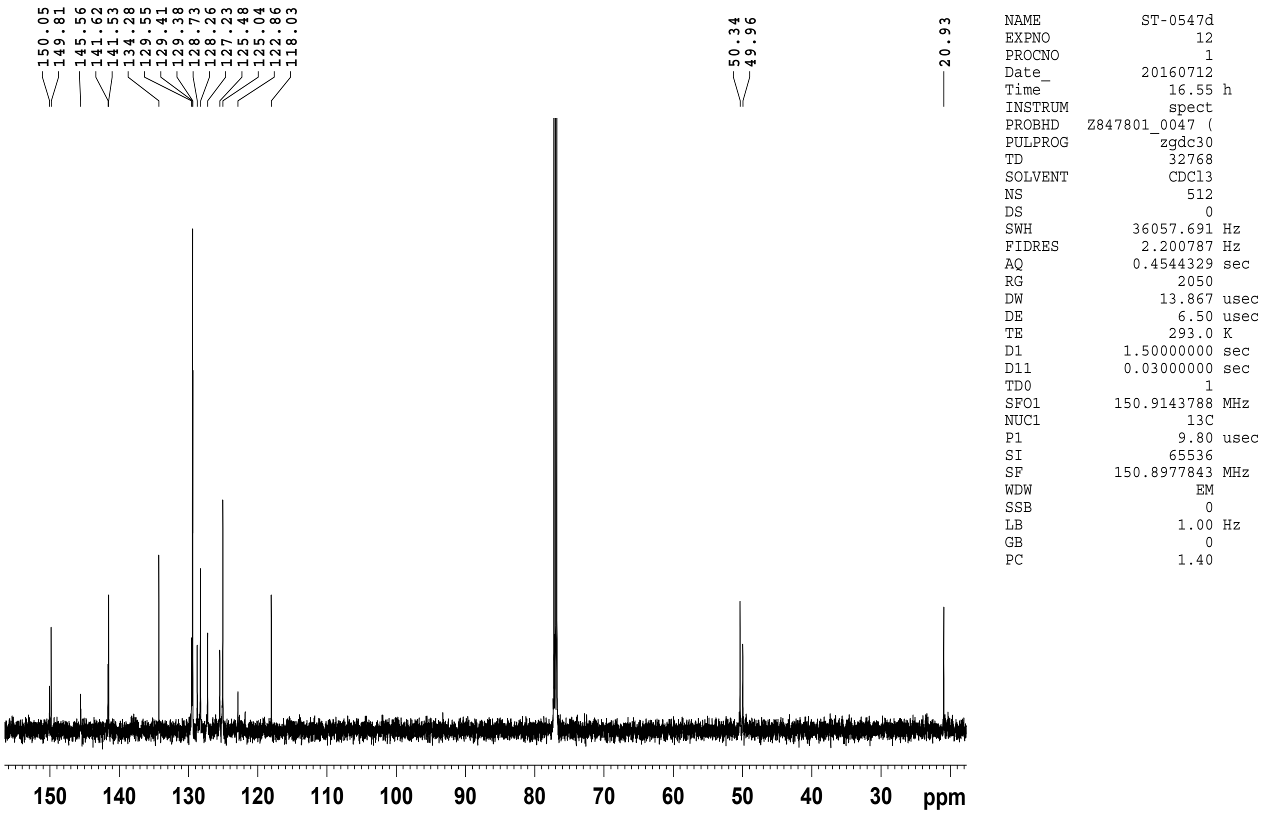

Figure S119. ^{13}C NMR spectrum of compound **6a**.

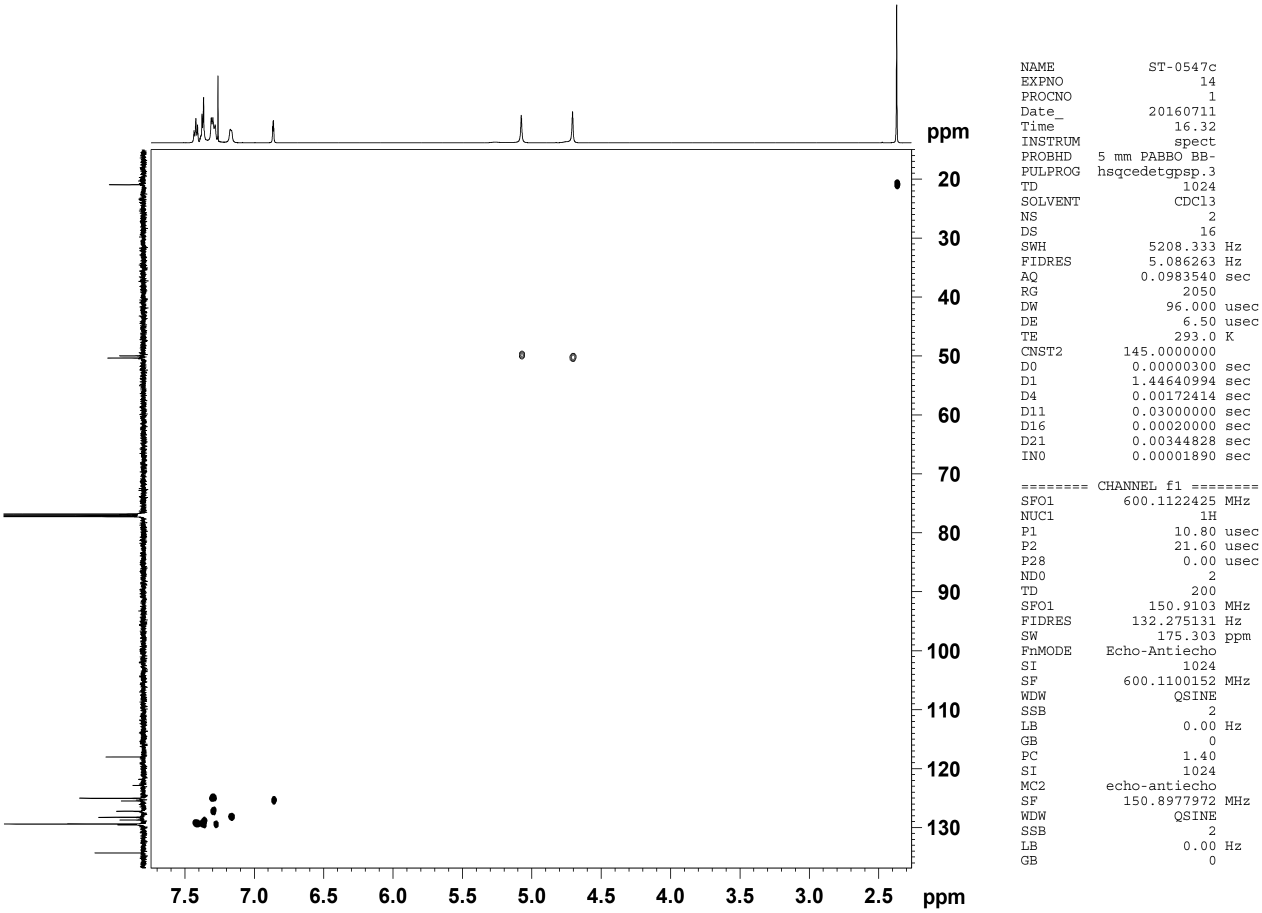

Figure S120. ^{1}H-^{13}C HSQC spectrum of compound **6a**.

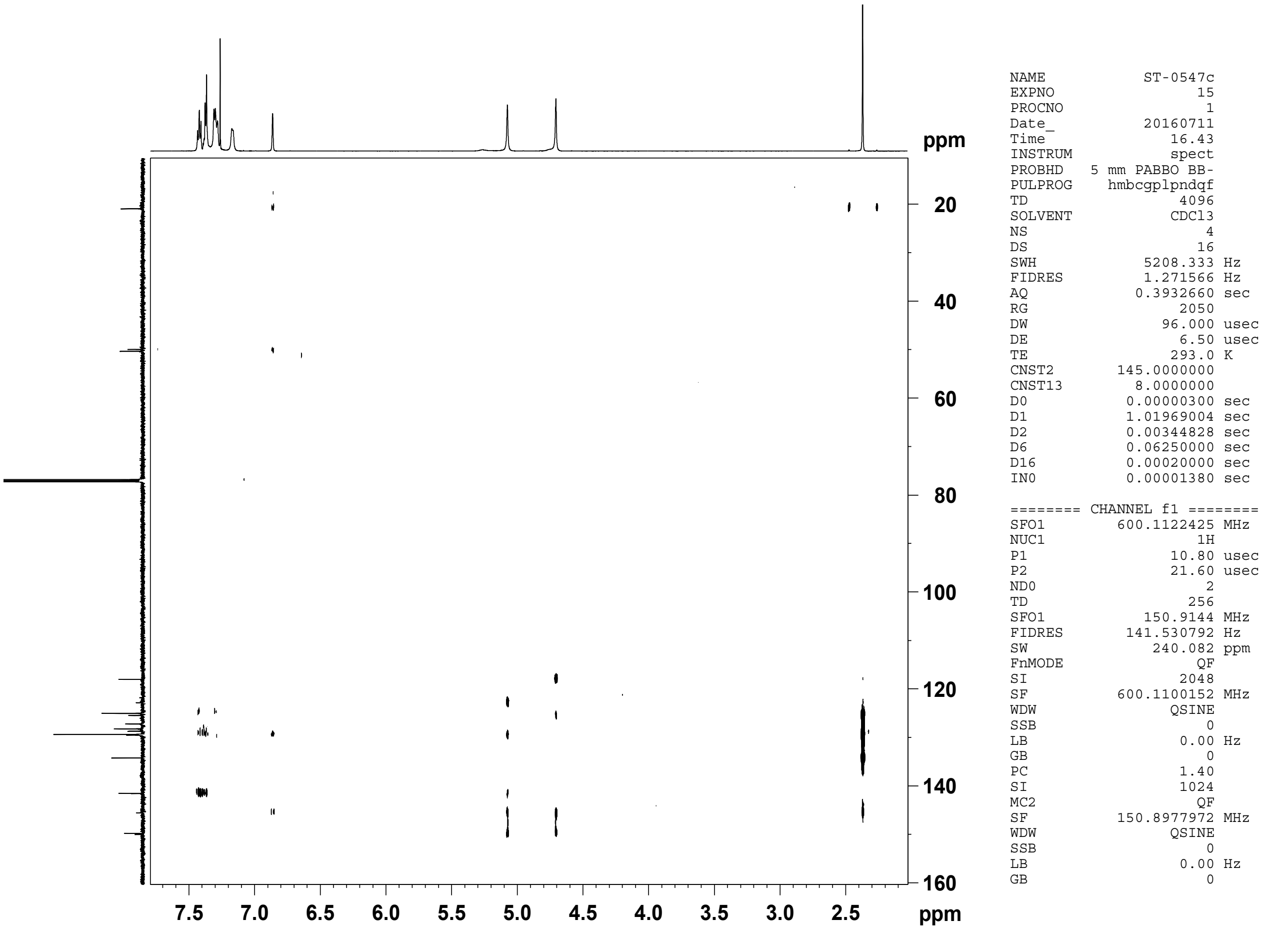

Figure S121. ^{1}H-^{13}C HMBC spectrum of compound **6a**.

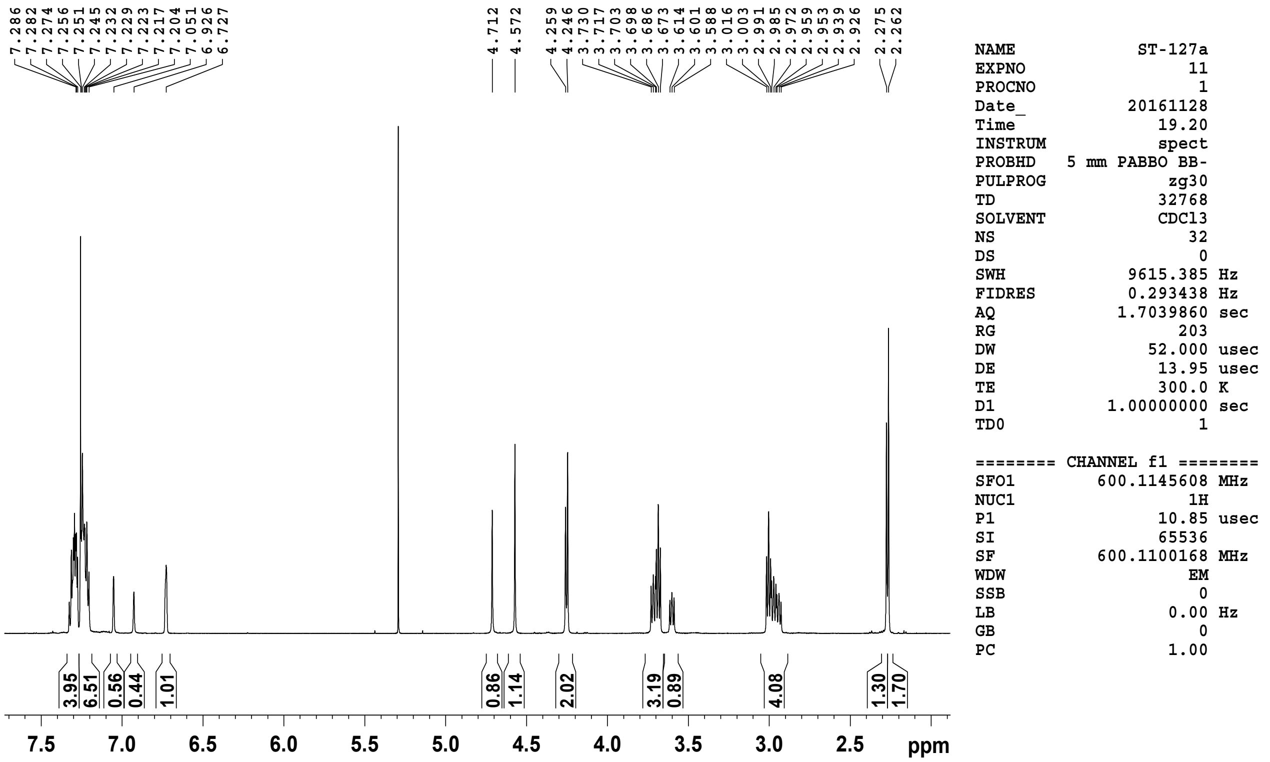

Figure S122. ^{1}H NMR spectrum of compound **6c**.

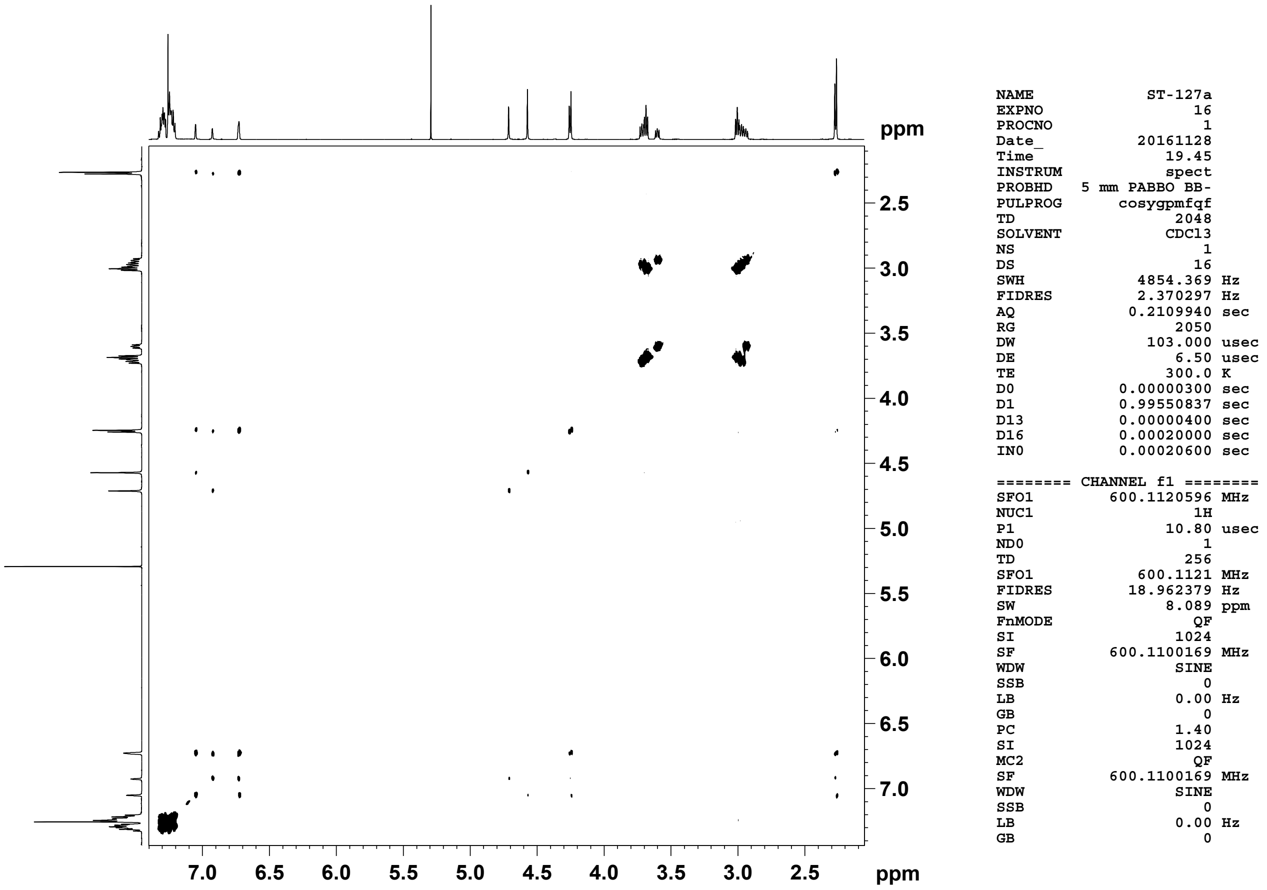

Figure S123. ^{1}H-^{1}H COSY spectrum of compound **6c**.

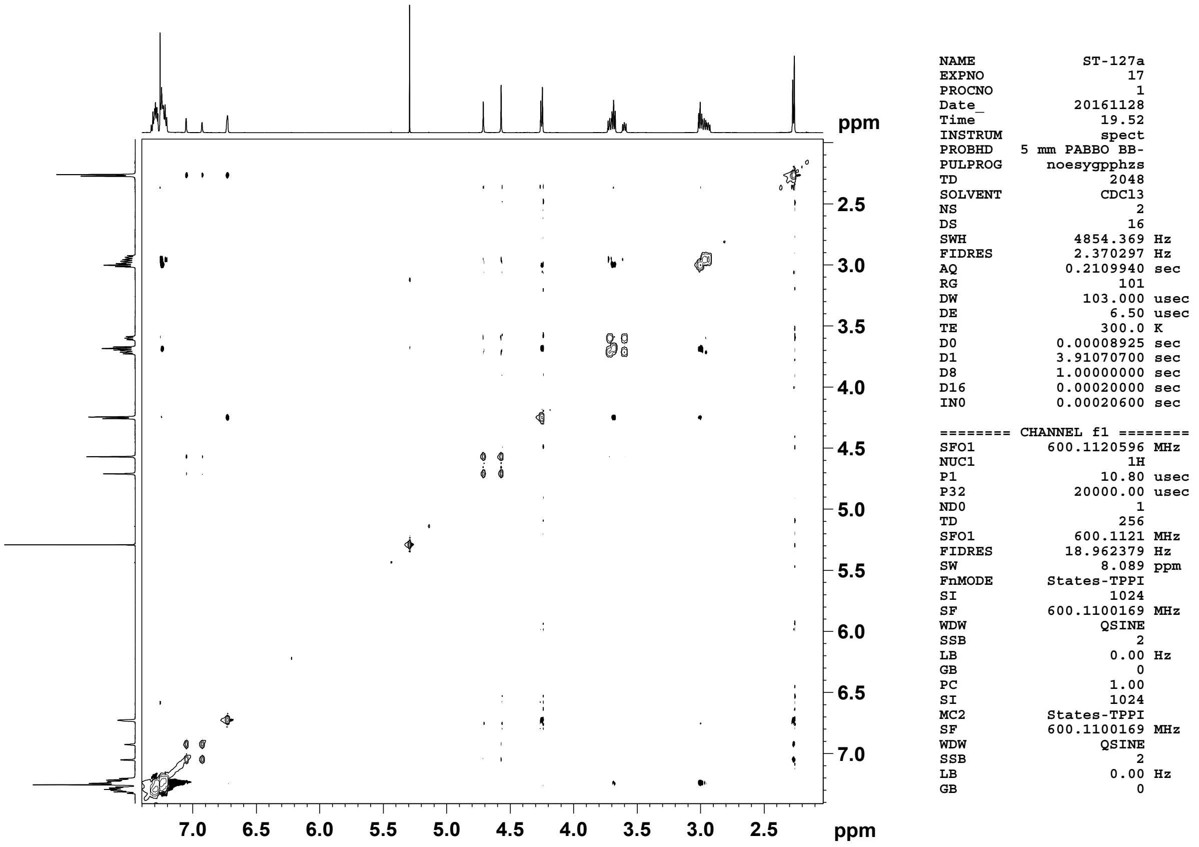

Figure S124. ^{1}H-^{1}H NOESY spectrum of compound **6c**.

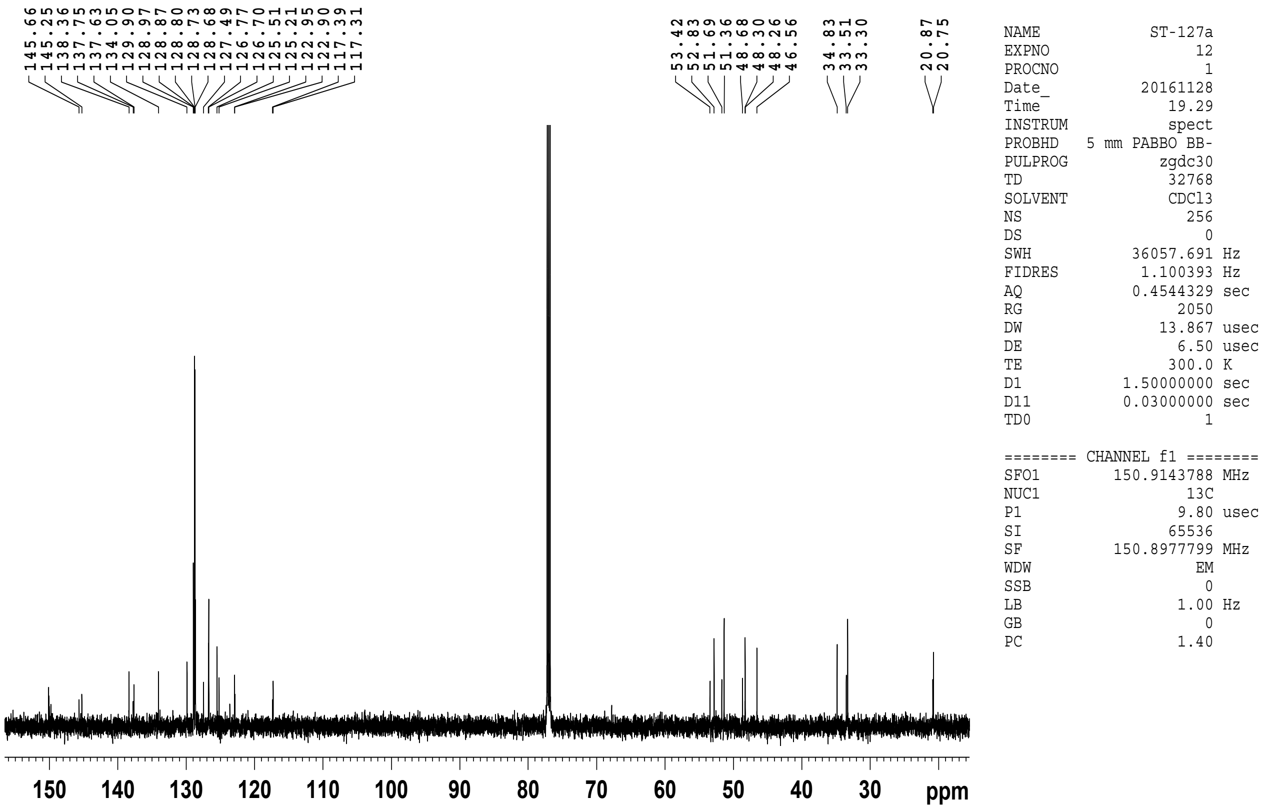

Figure S125. ^{13}C NMR spectrum of compound **6c**.

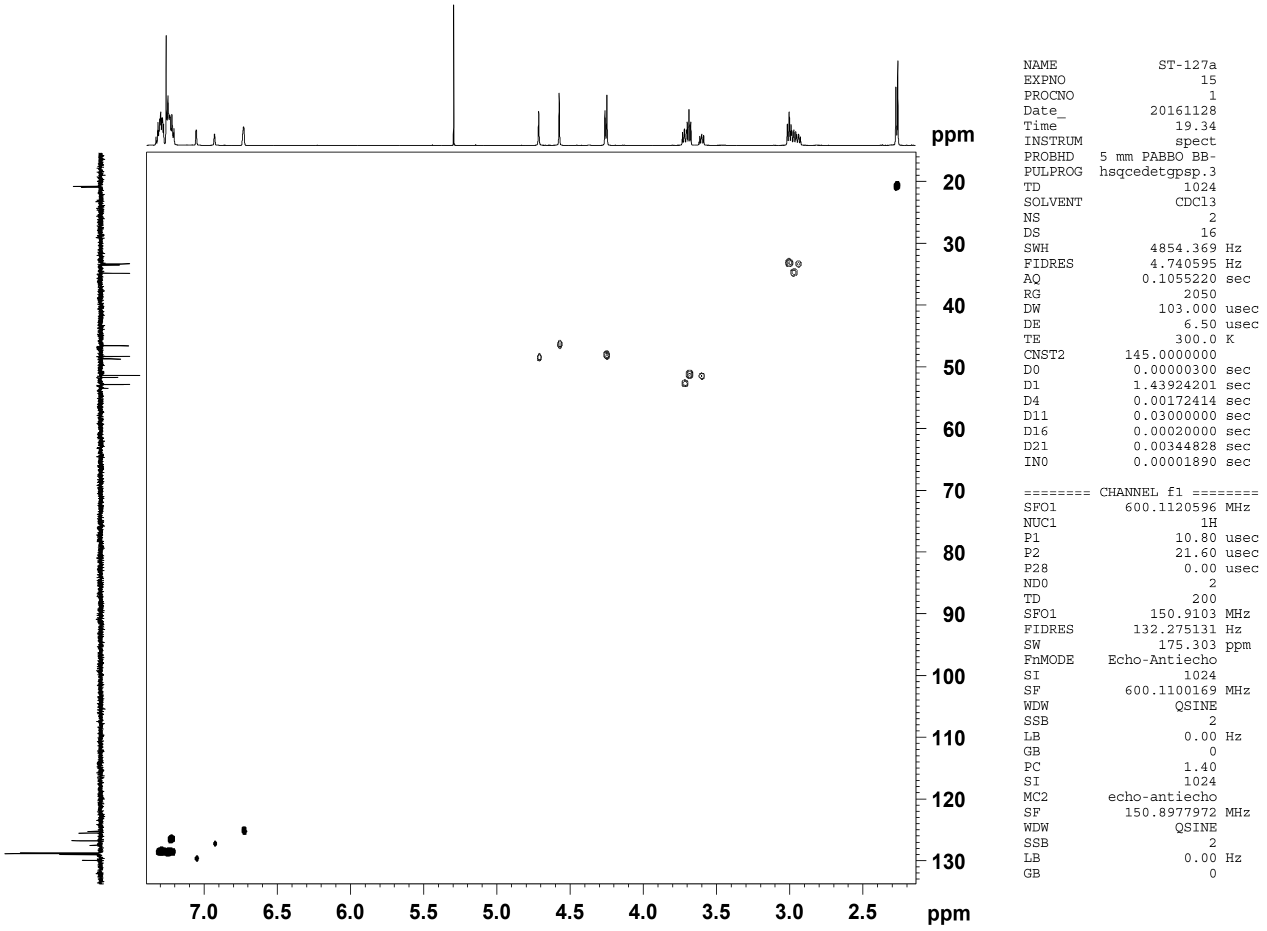

Figure S126. ^{1}H-^{13}C HSQC spectrum of compound **6c**.

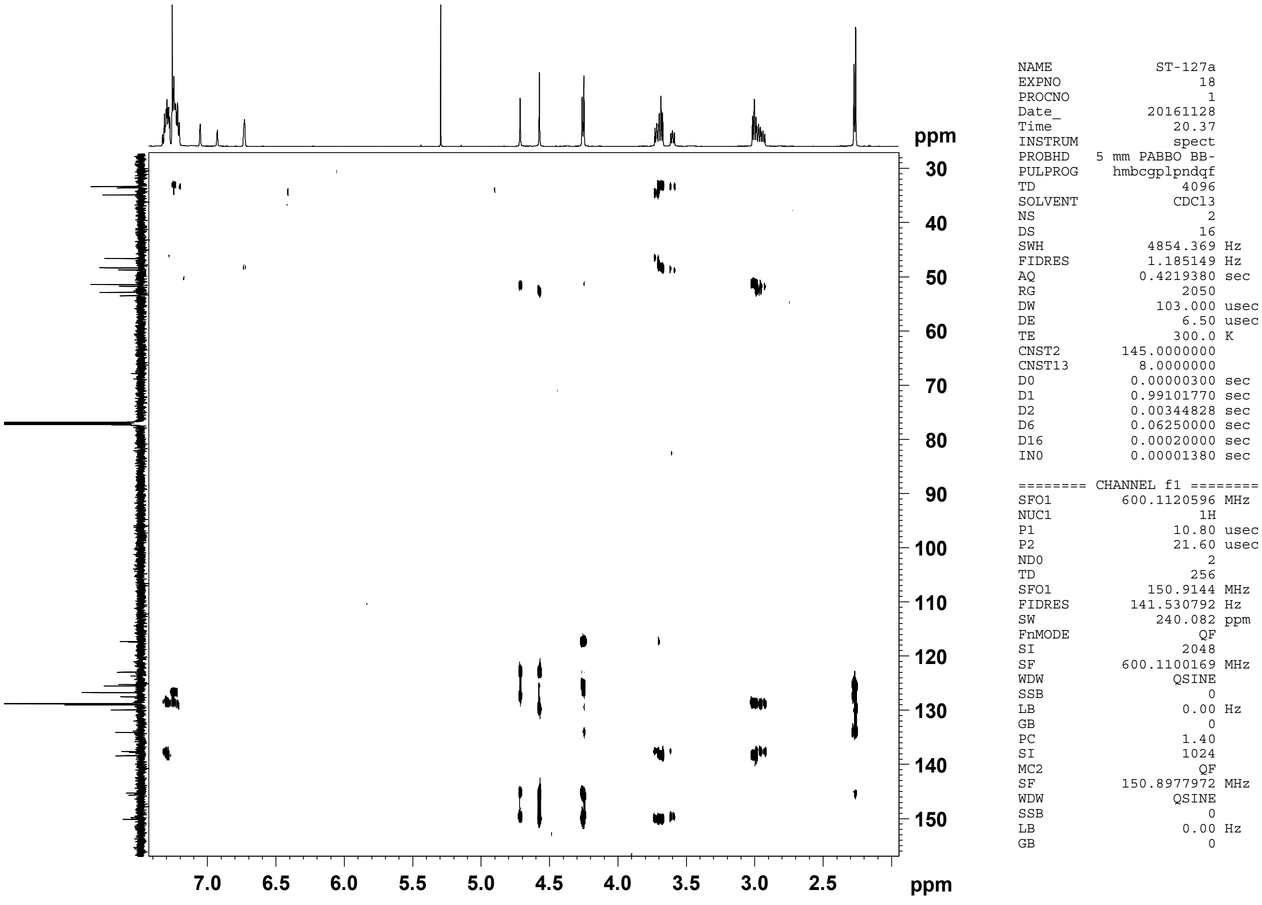

Figure S127. ^{1}H-^{13}C HMBC spectrum of compound **6c**.

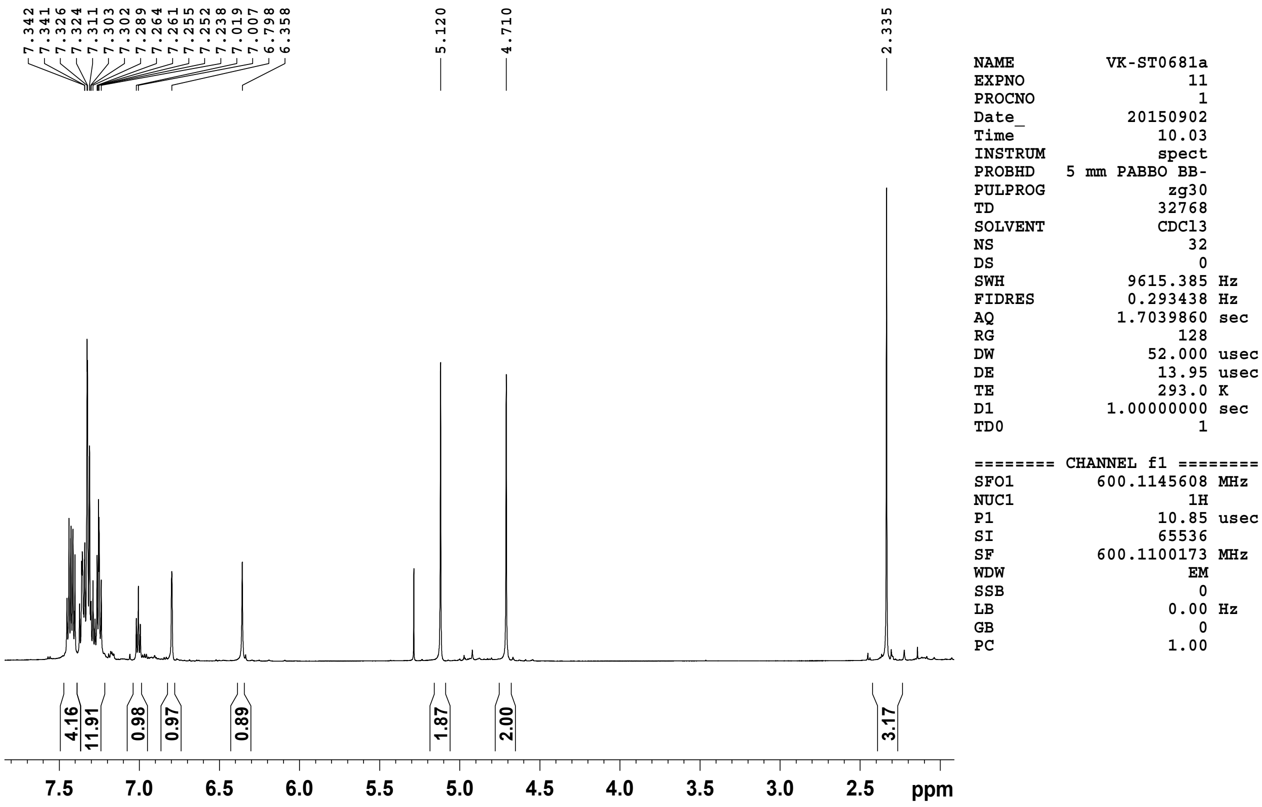

Figure S128. ^{1}H NMR spectrum of compound **7aa**.

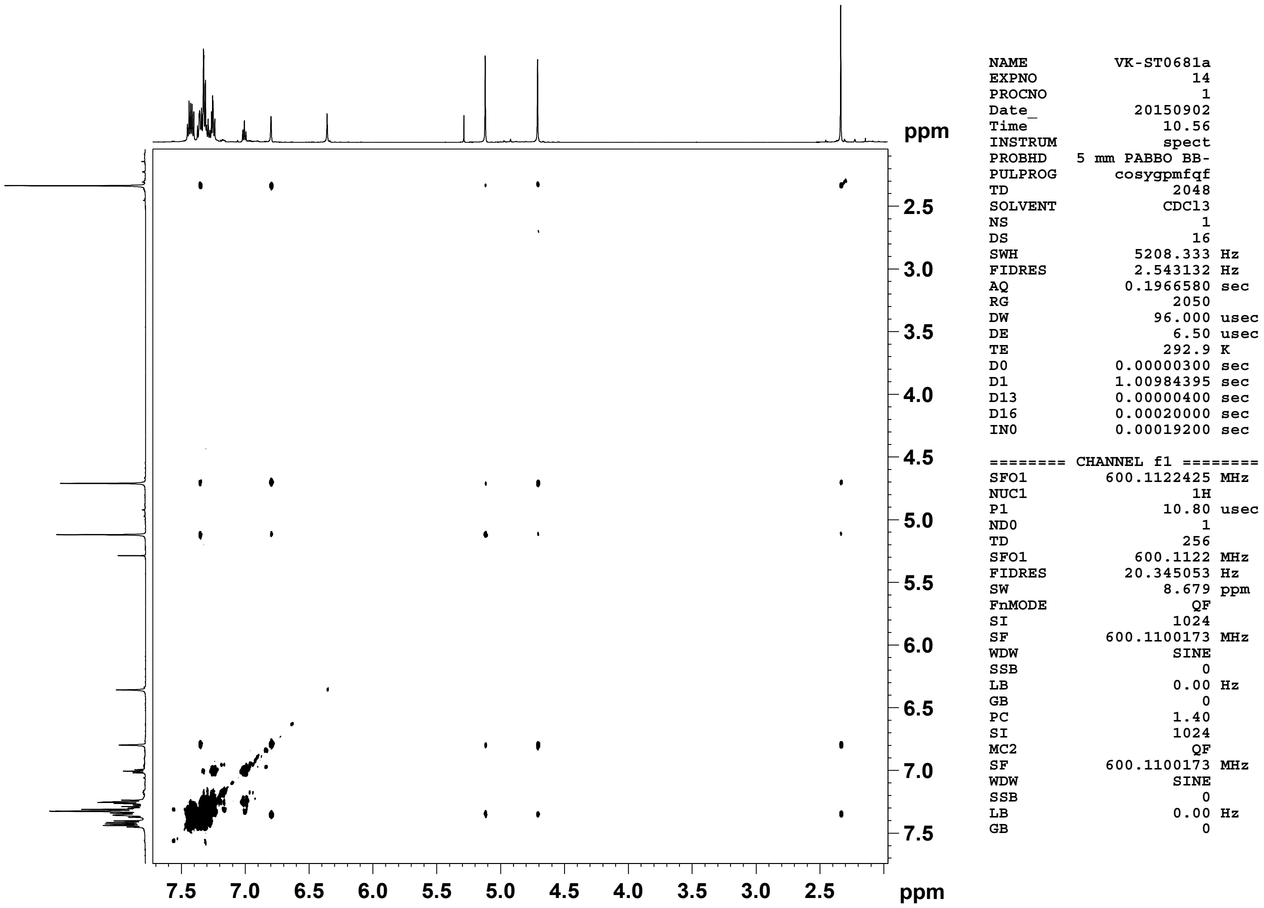

Figure S129. ^{1}H-^{1}H COSY spectrum of compound **7aa**.

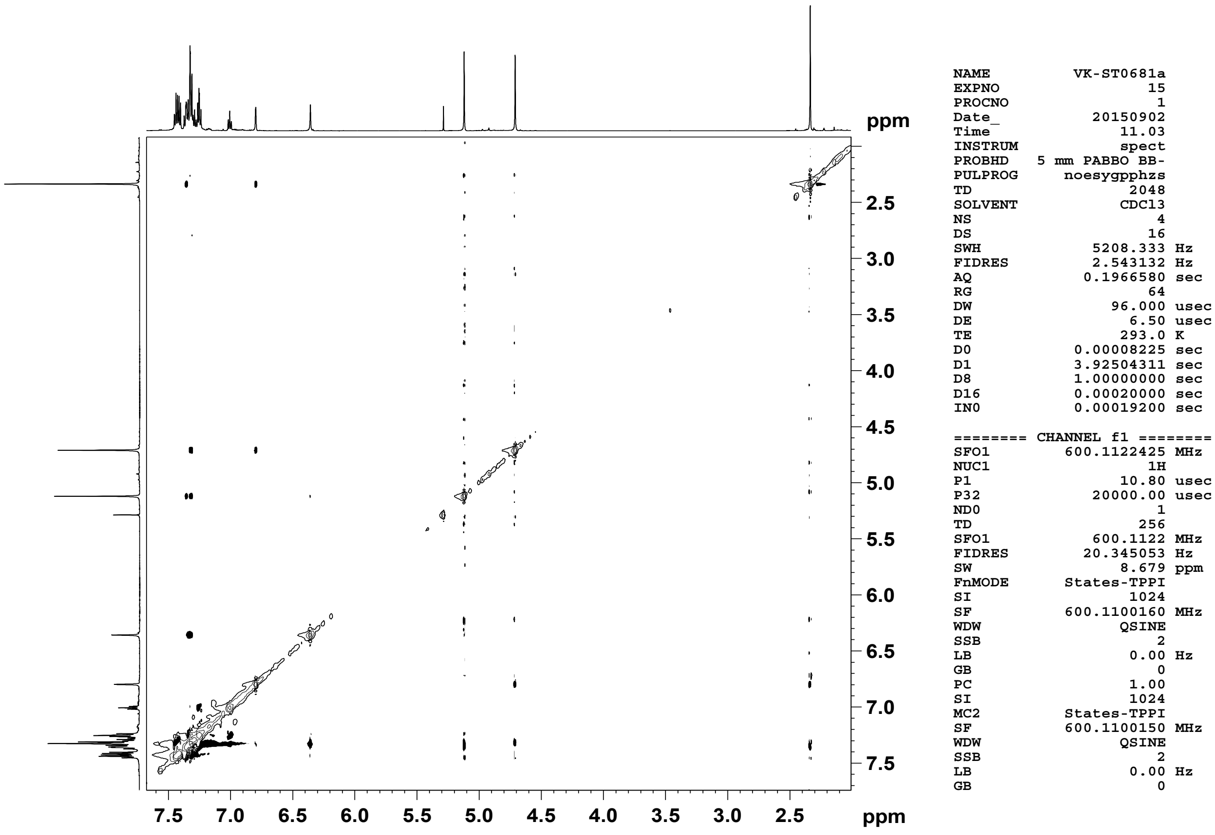

Figure S130. ^{1}H-^{1}H NOESY spectrum of compound **7aa**.

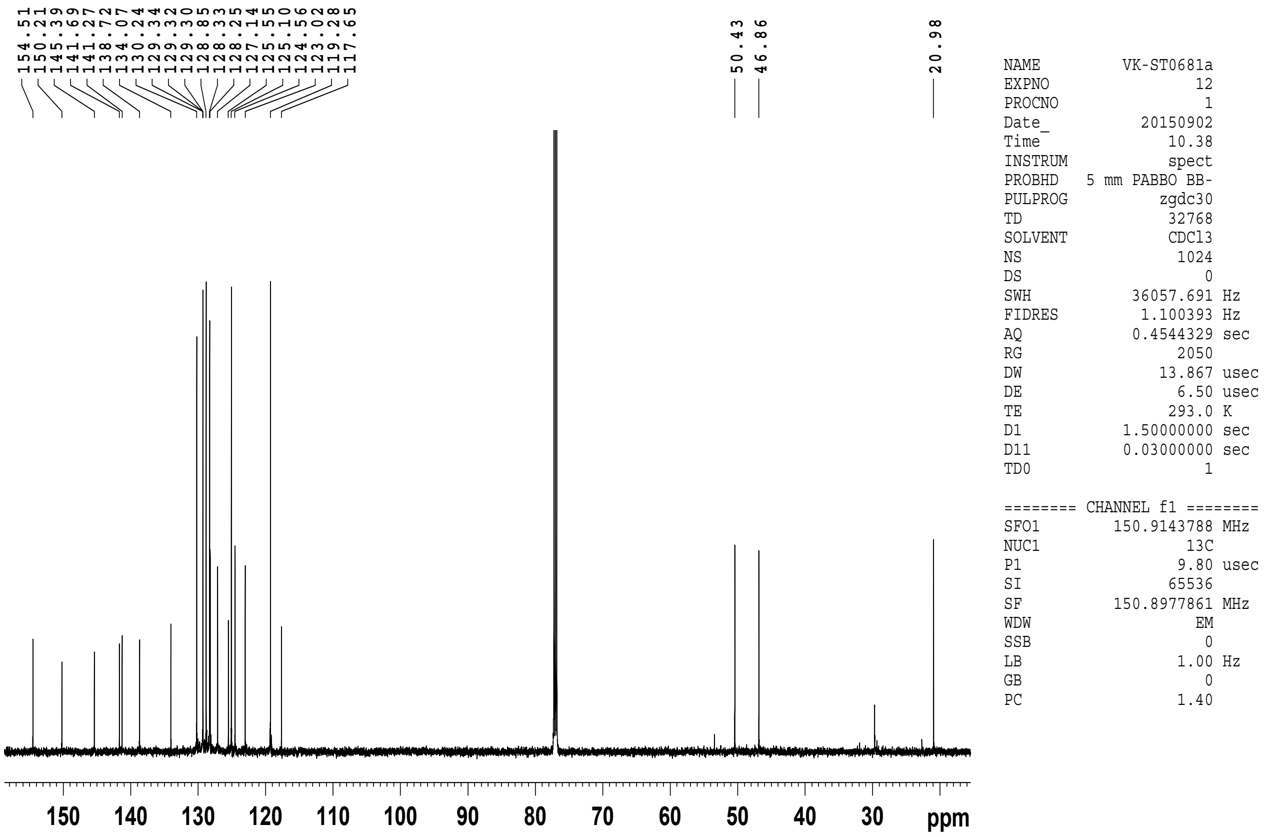

Figure S131. ^{13}C NMR spectrum of compound **7aa**.

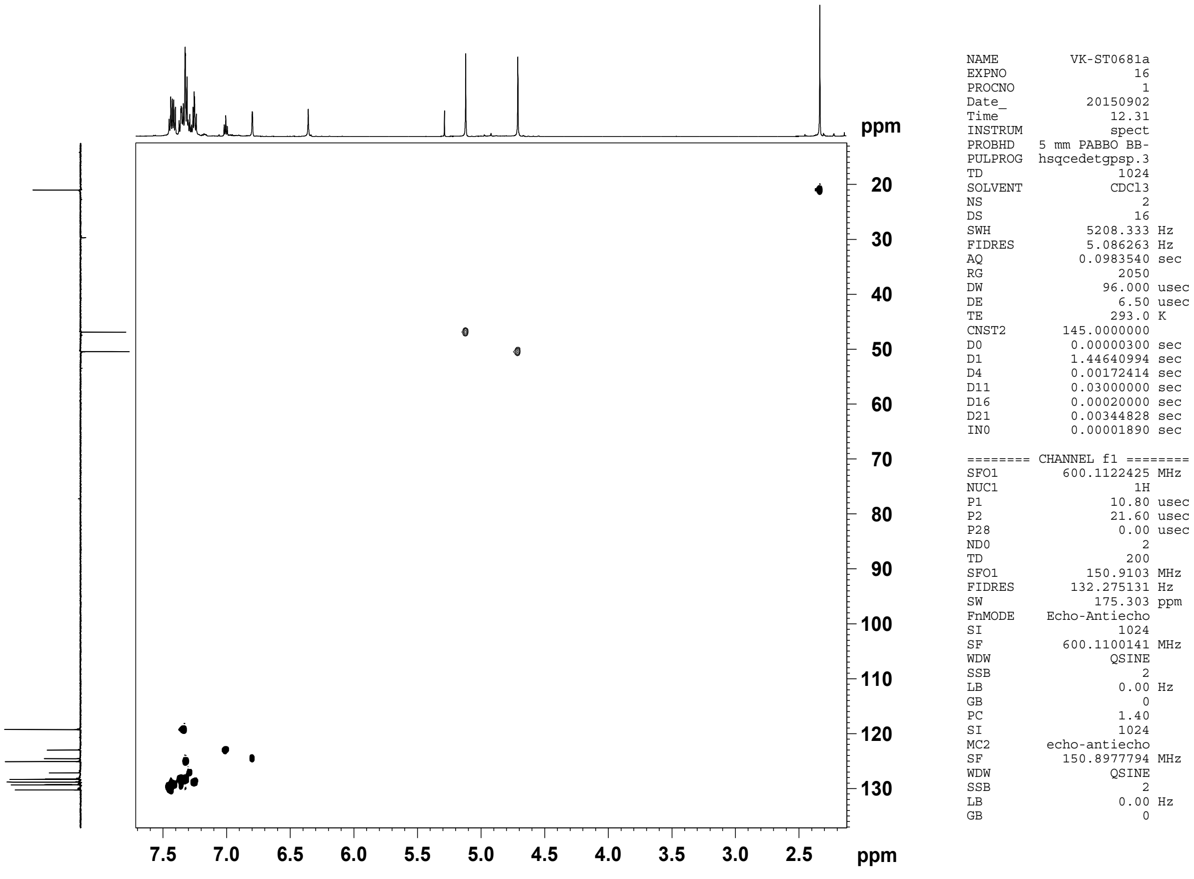

Figure S132. ^{1}H-^{13}C HSQC spectrum of compound **7aa**.

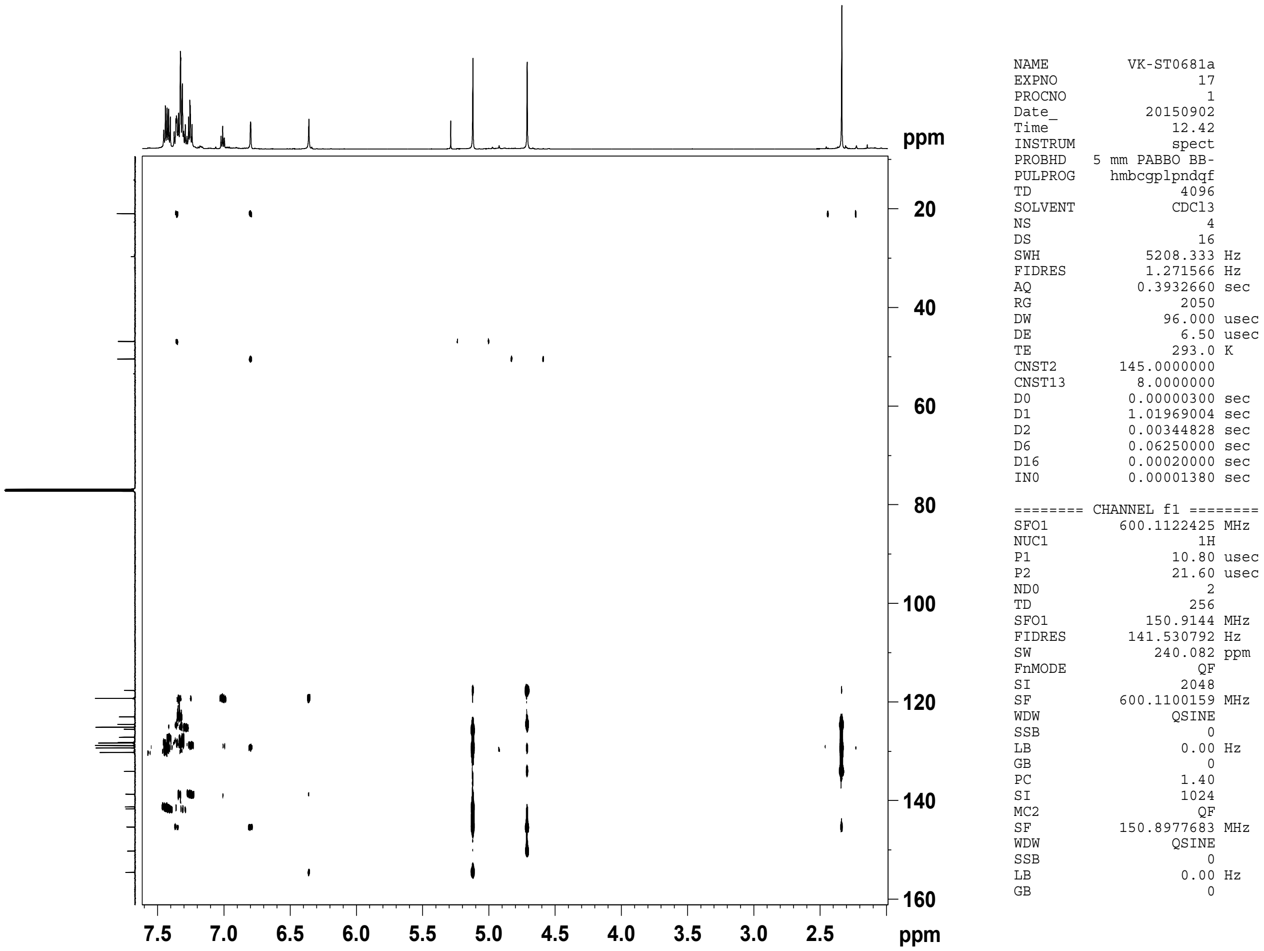

Figure S133. ^{1}H-^{13}C HMBC spectrum of compound **7aa**.

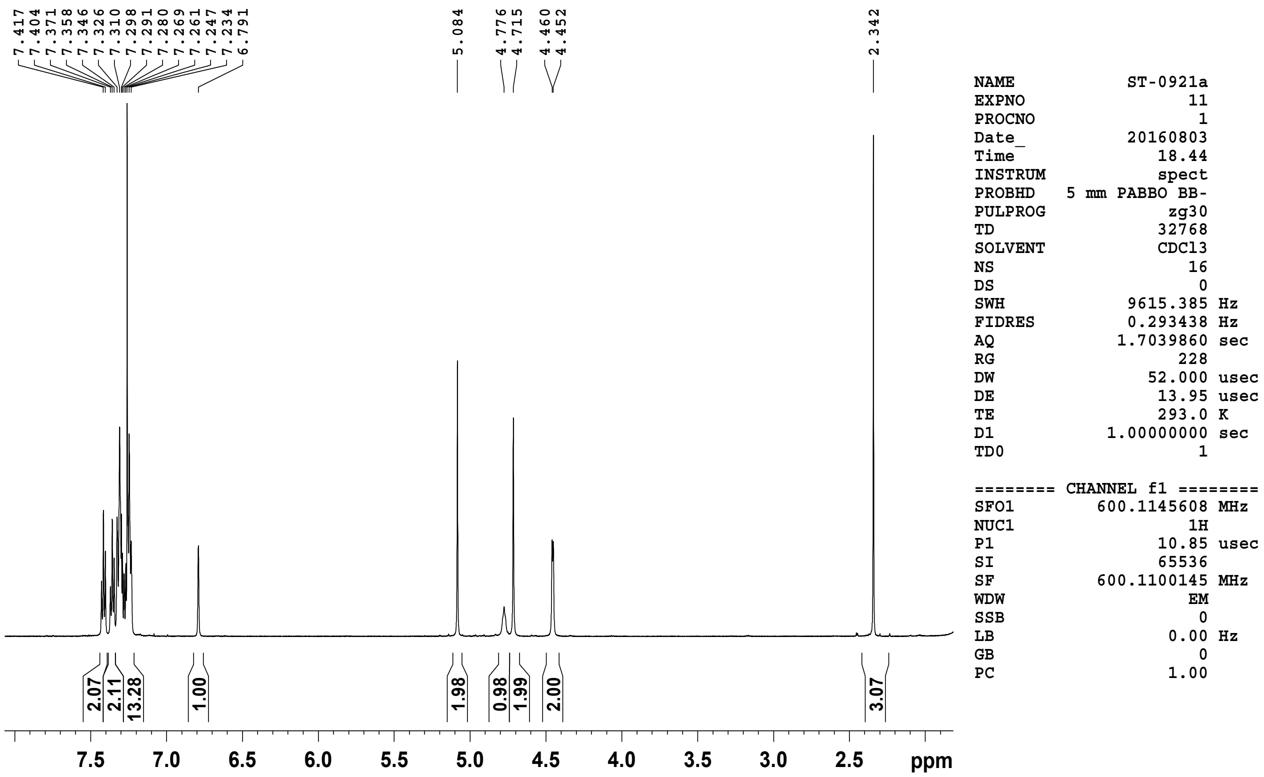

Figure S134. ^{1}H NMR spectrum of compound **7ab**.

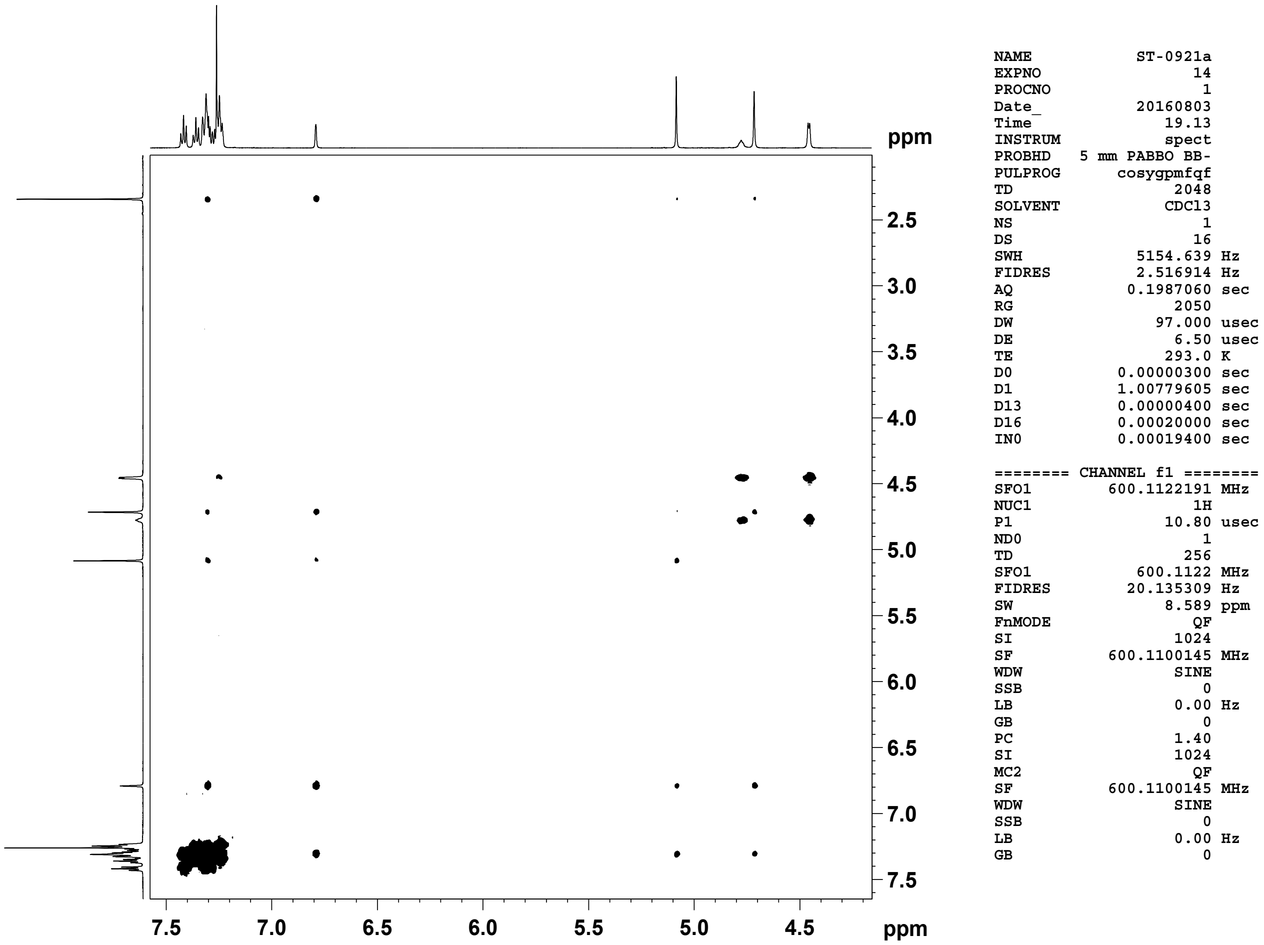

Figure S135. ^{1}H-^{1}H COSY spectrum of compound **7ab**.

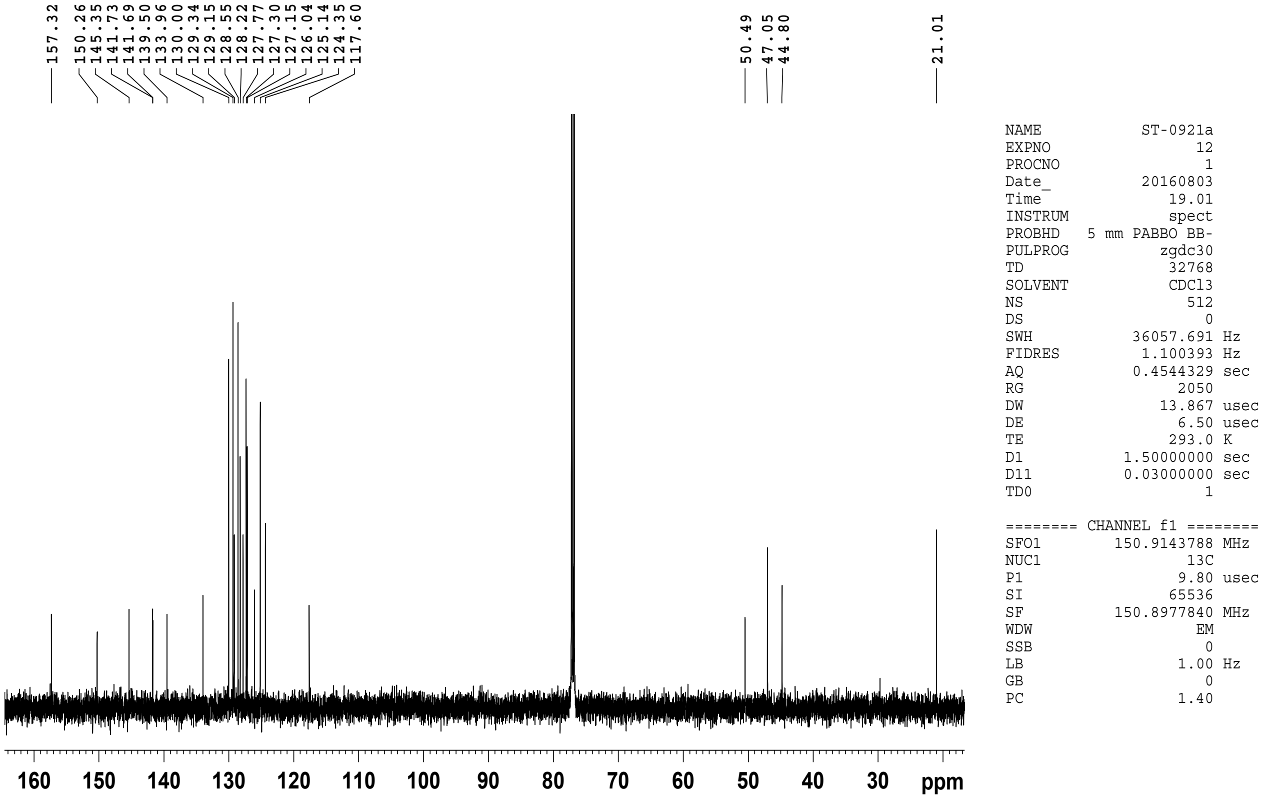

Figure S136. ^{13}C NMR spectrum of compound **7ab**.

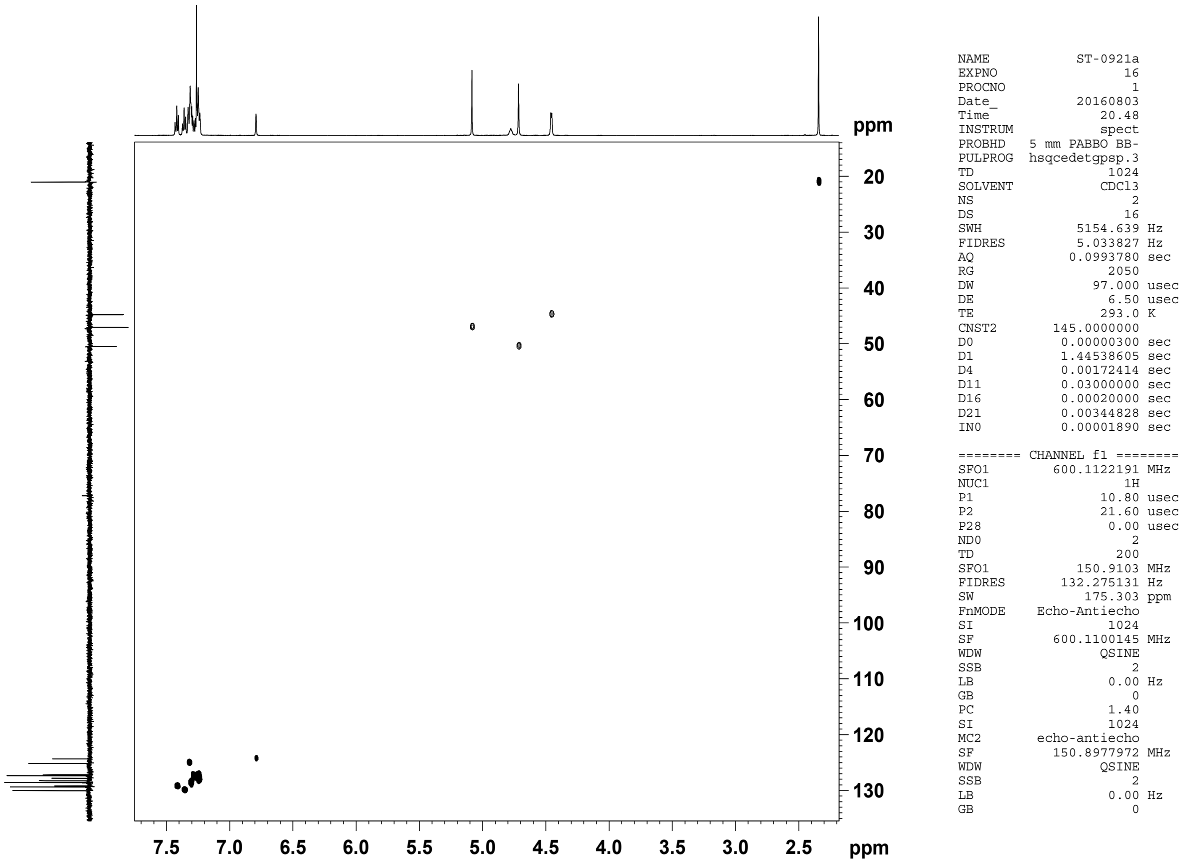

Figure S137. ^{1}H-^{13}C HSQC spectrum of compound **7ab**.

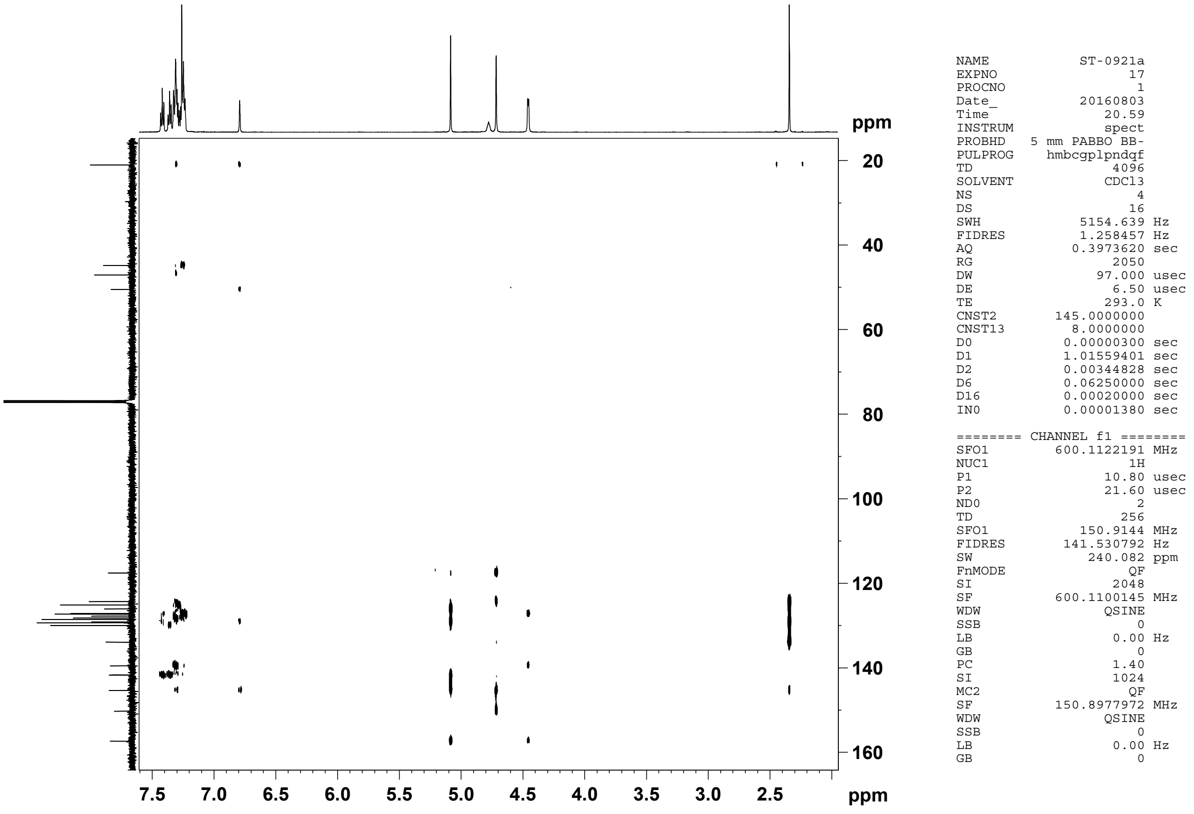

Figure S138. ^{1}H-^{13}C HMBC spectrum of compound **7ab**.

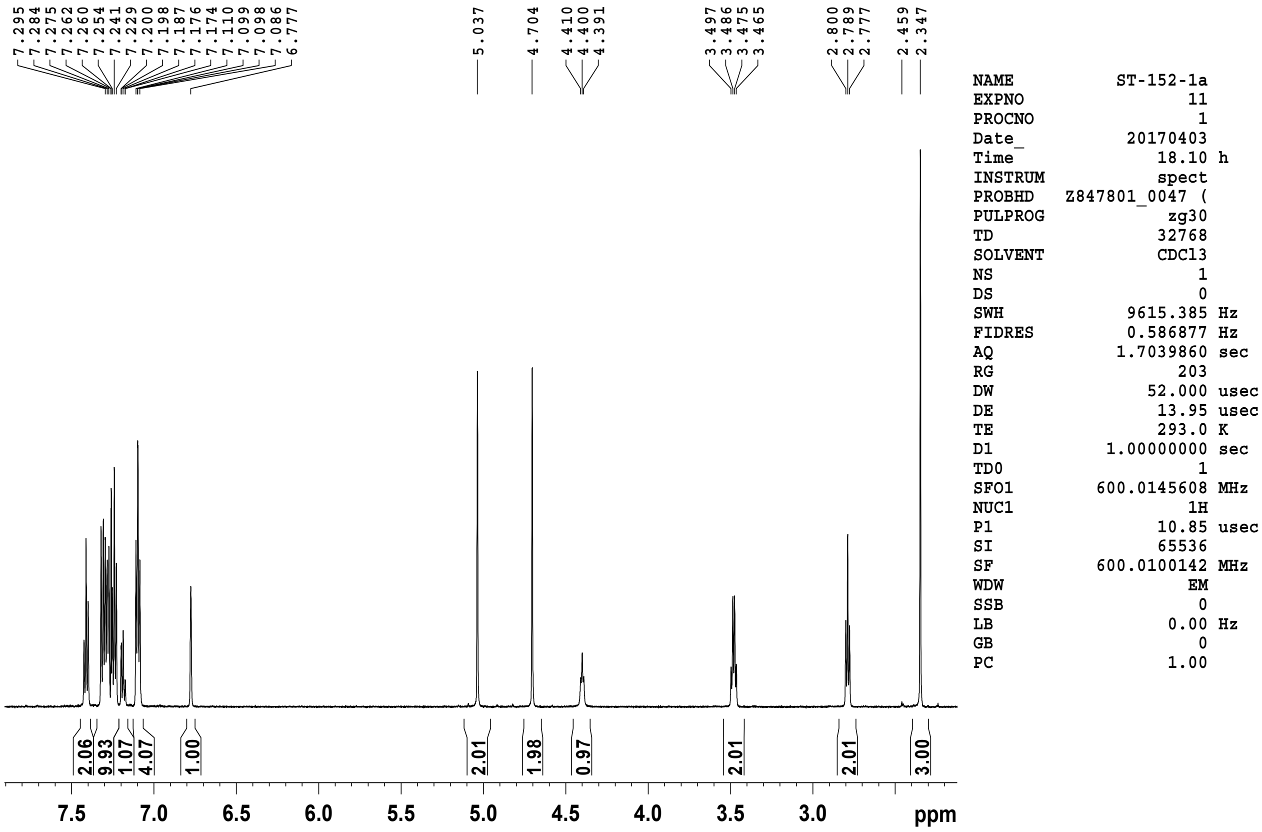

Figure S139. ^{1}H NMR spectrum of compound **7ac**.

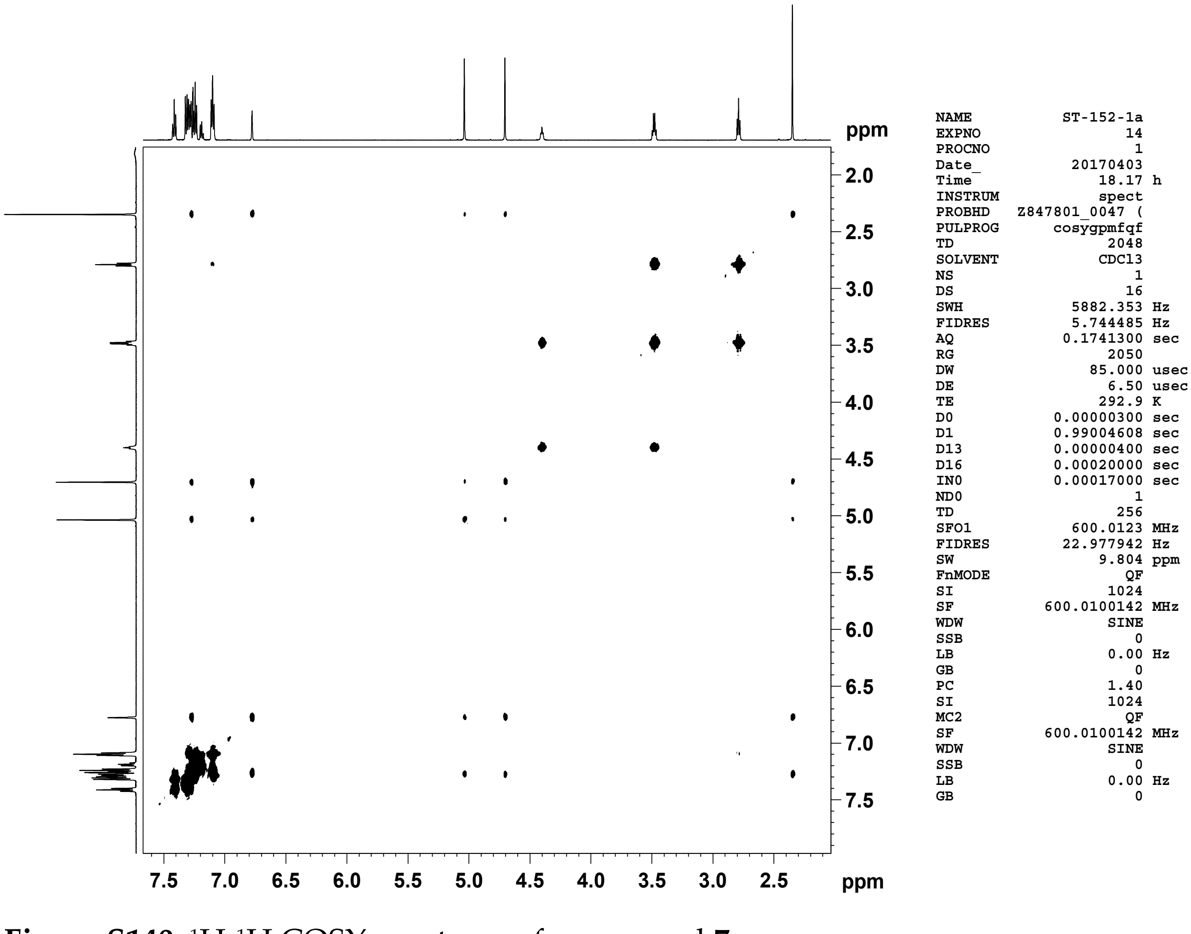

Figure S140. ^{1}H-^{1}H COSY spectrum of compound **7ac**.

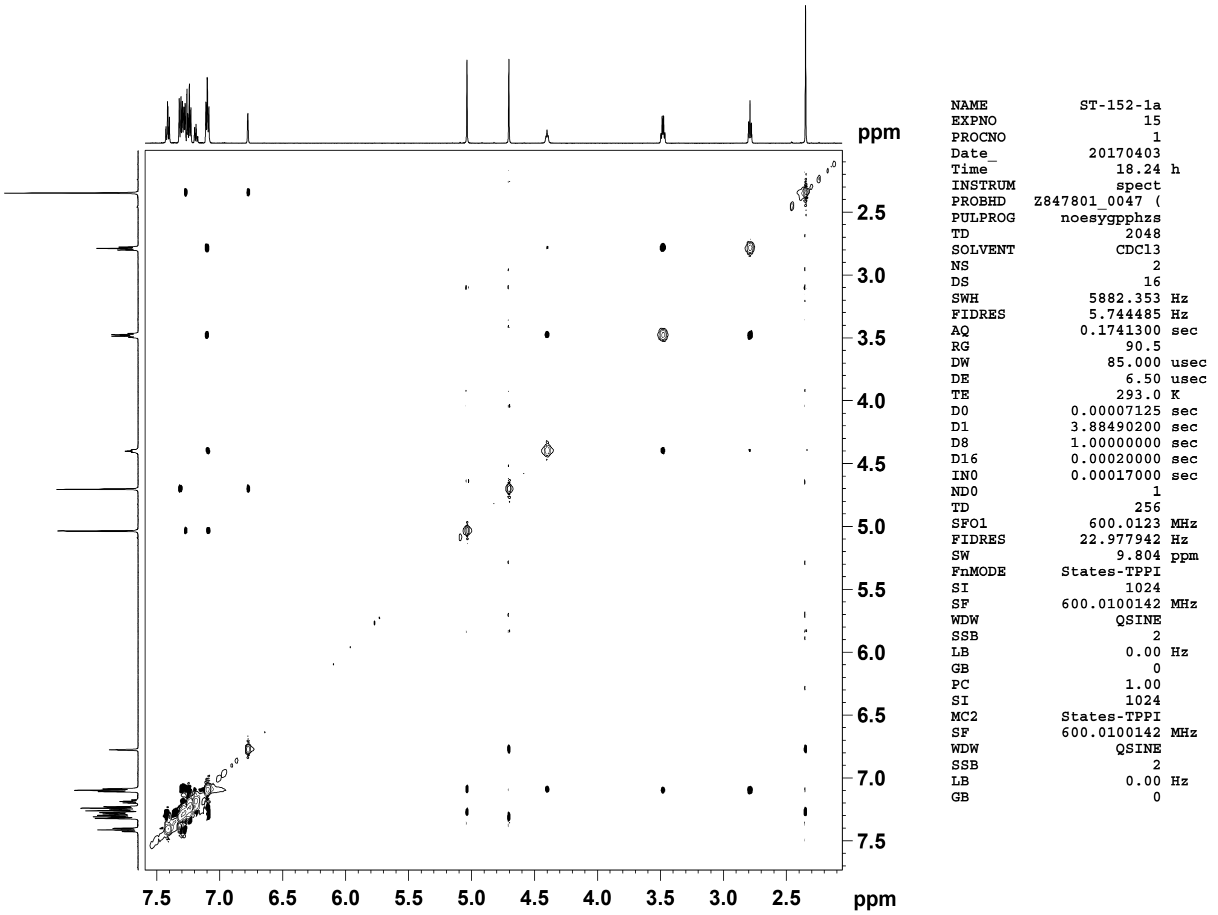

Figure S141. ^{1}H-^{1}H NOESY spectrum of compound **7ac**.

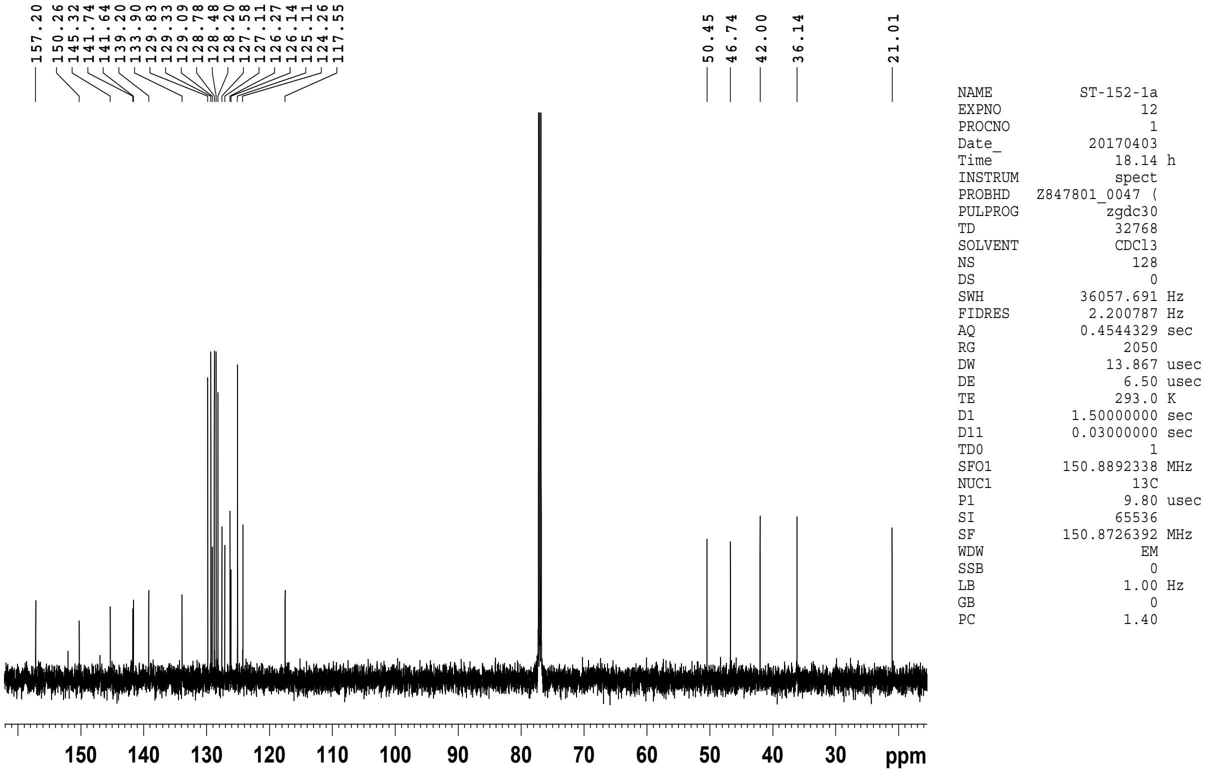

Figure S142. ^{13}C NMR spectrum of compound **7ac**.

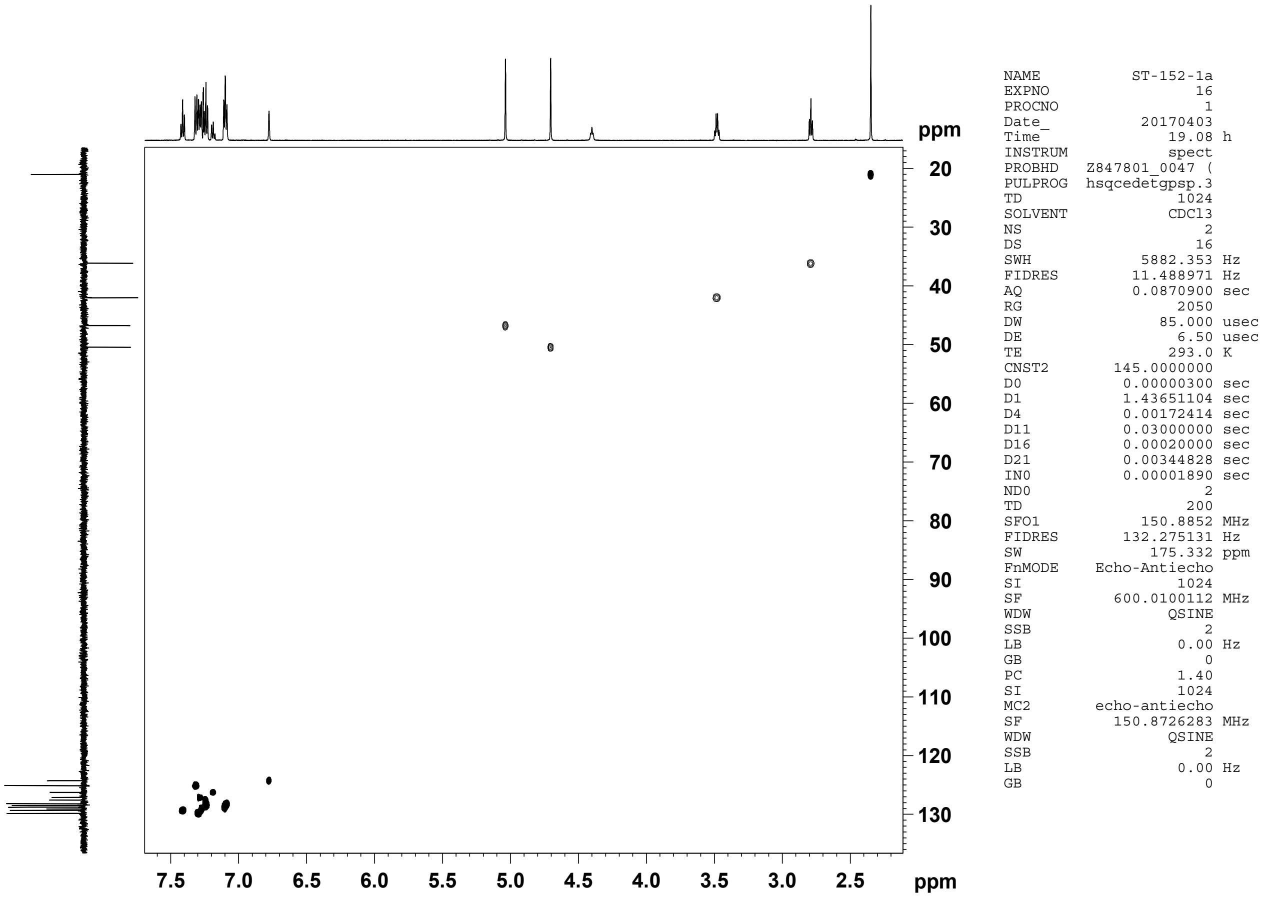

Figure S143. ^{1}H-^{13}C HSQC spectrum of compound **7ac**.

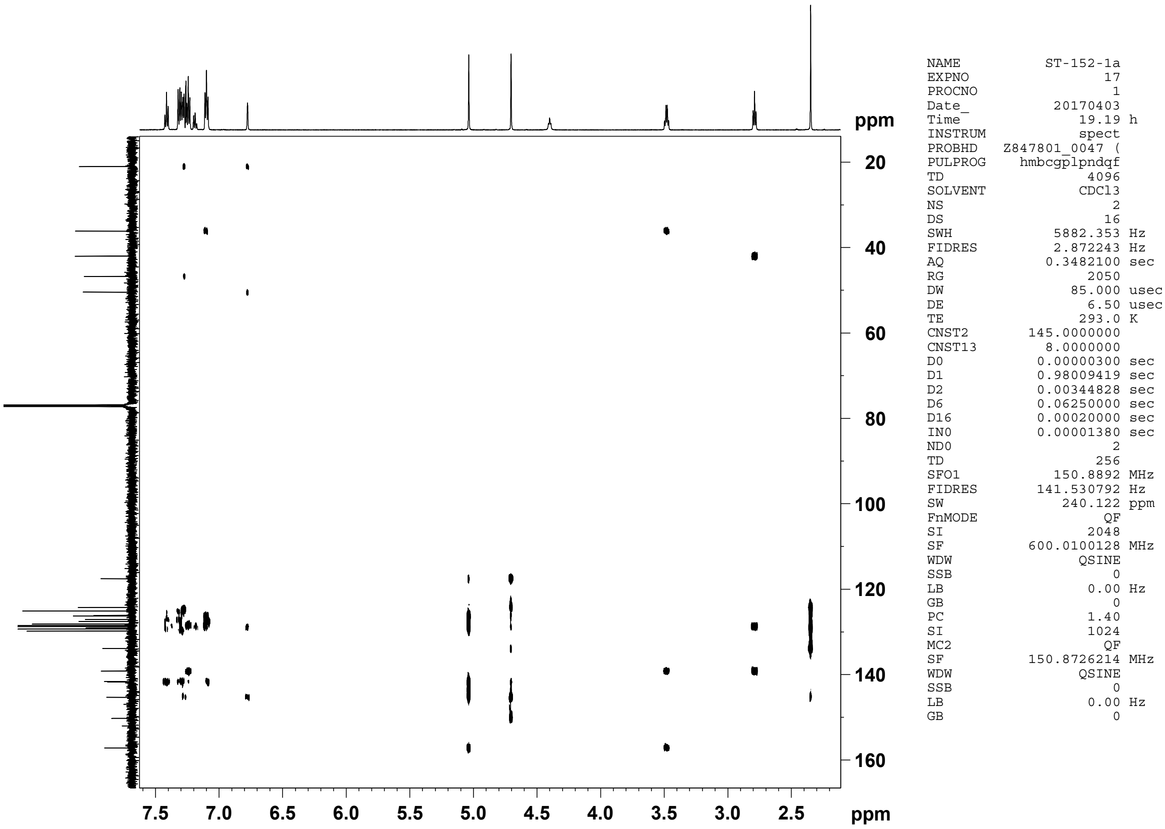

Figure S144. ^{1}H-^{13}C HMBC spectrum of compound **7ac**.

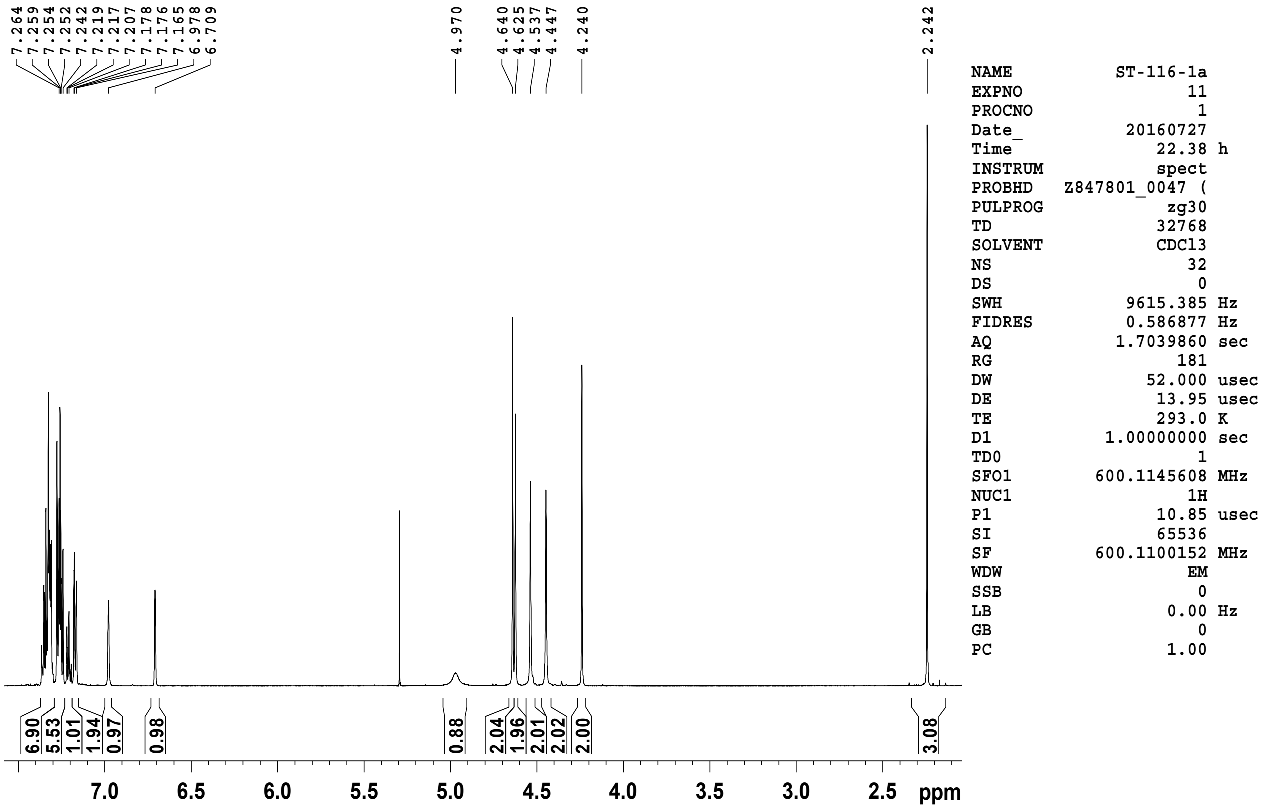

Figure S145. ^{1}H NMR spectrum of compound **7bb**.

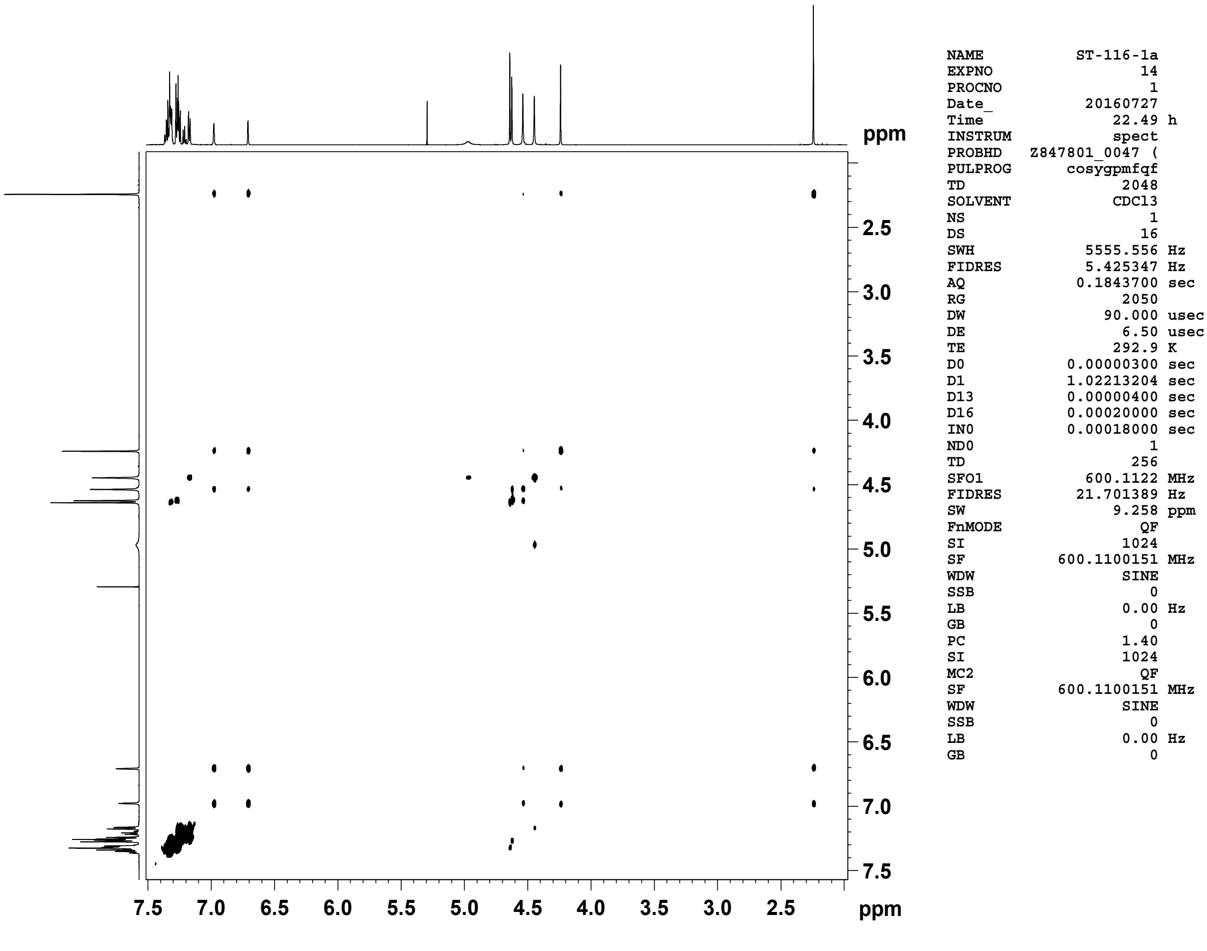

Figure S146. ^{1}H-^{1}H COSY spectrum of compound **7bb**.

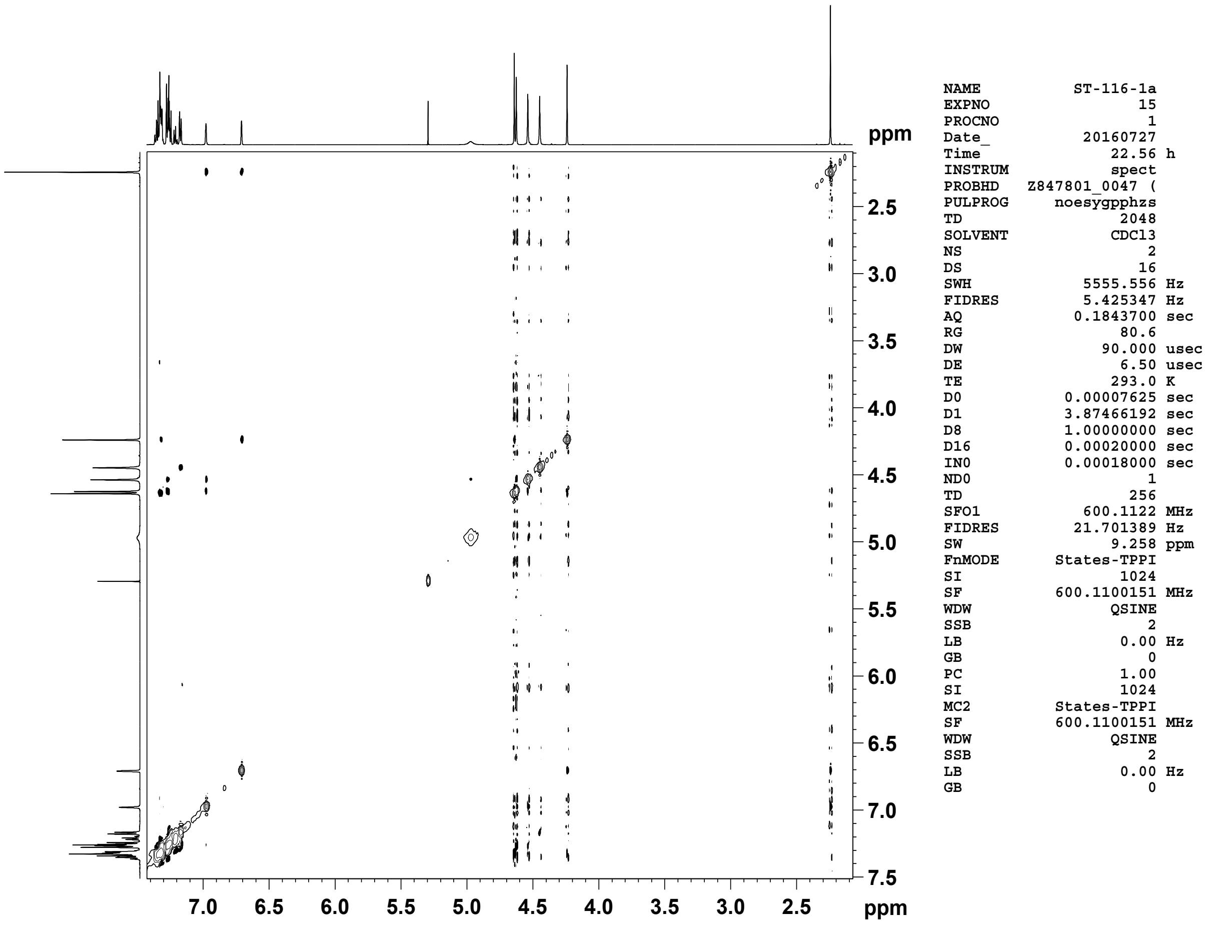

Figure S147. ^1H-^1H NOESY spectrum of compound **7bb**.

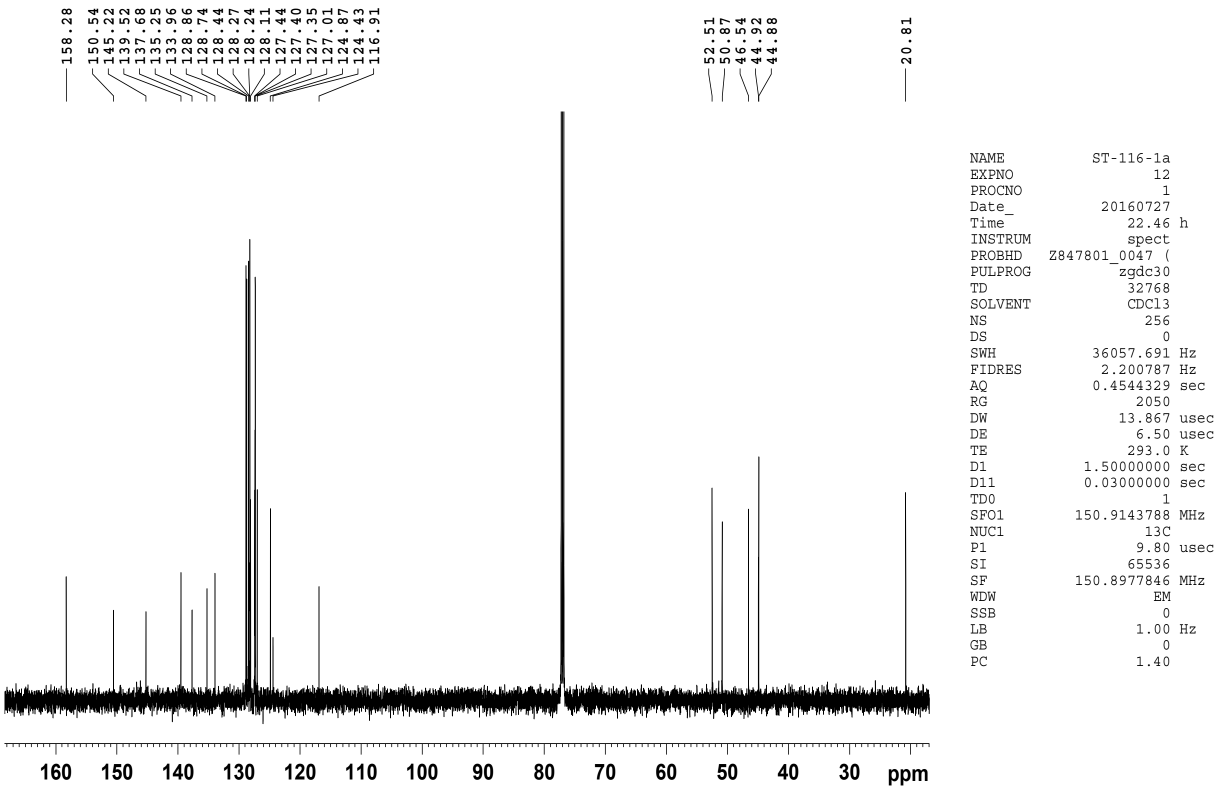

Figure S148. ^{13}C NMR spectrum of compound **7bb**.

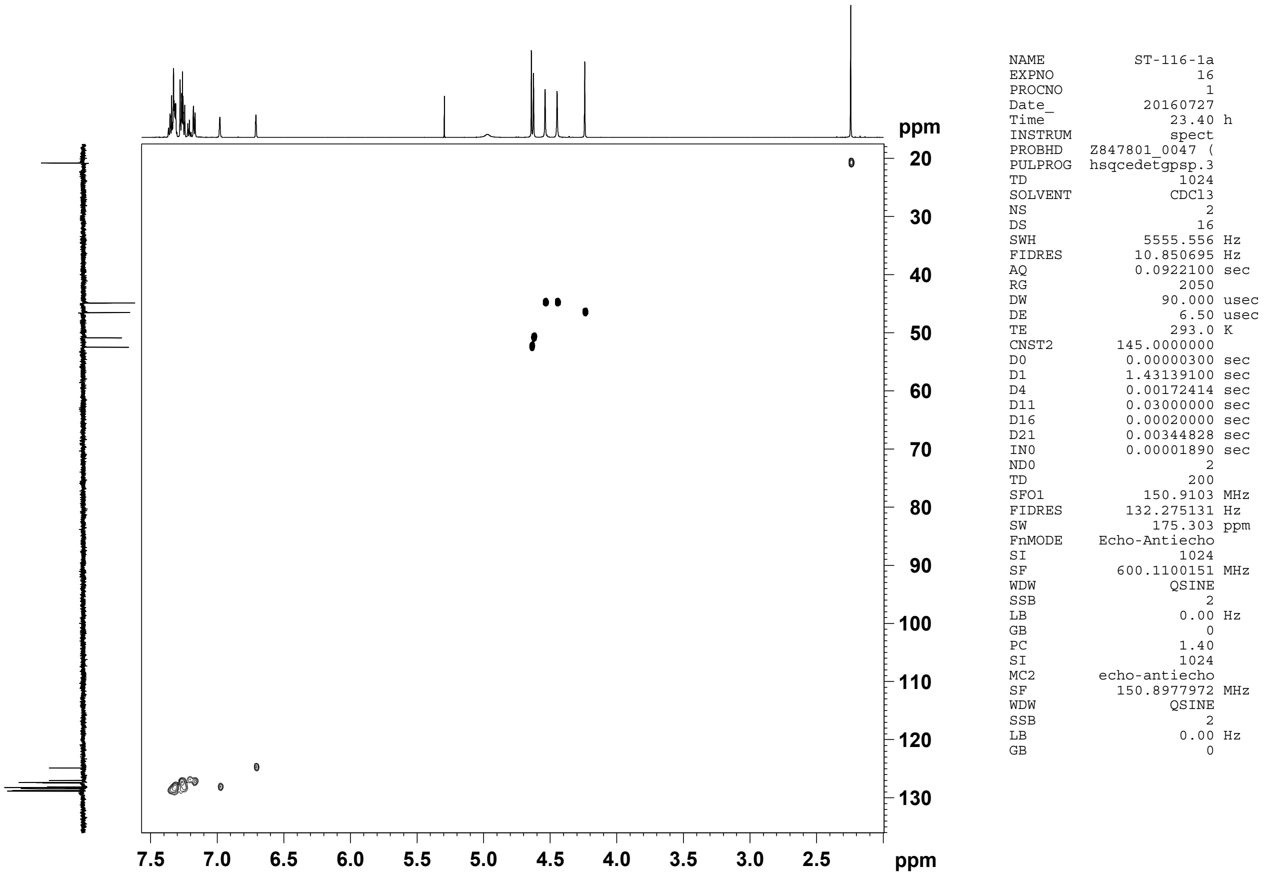

Figure S149. ^{1}H-^{13}C HSQC spectrum of compound **7bb**.

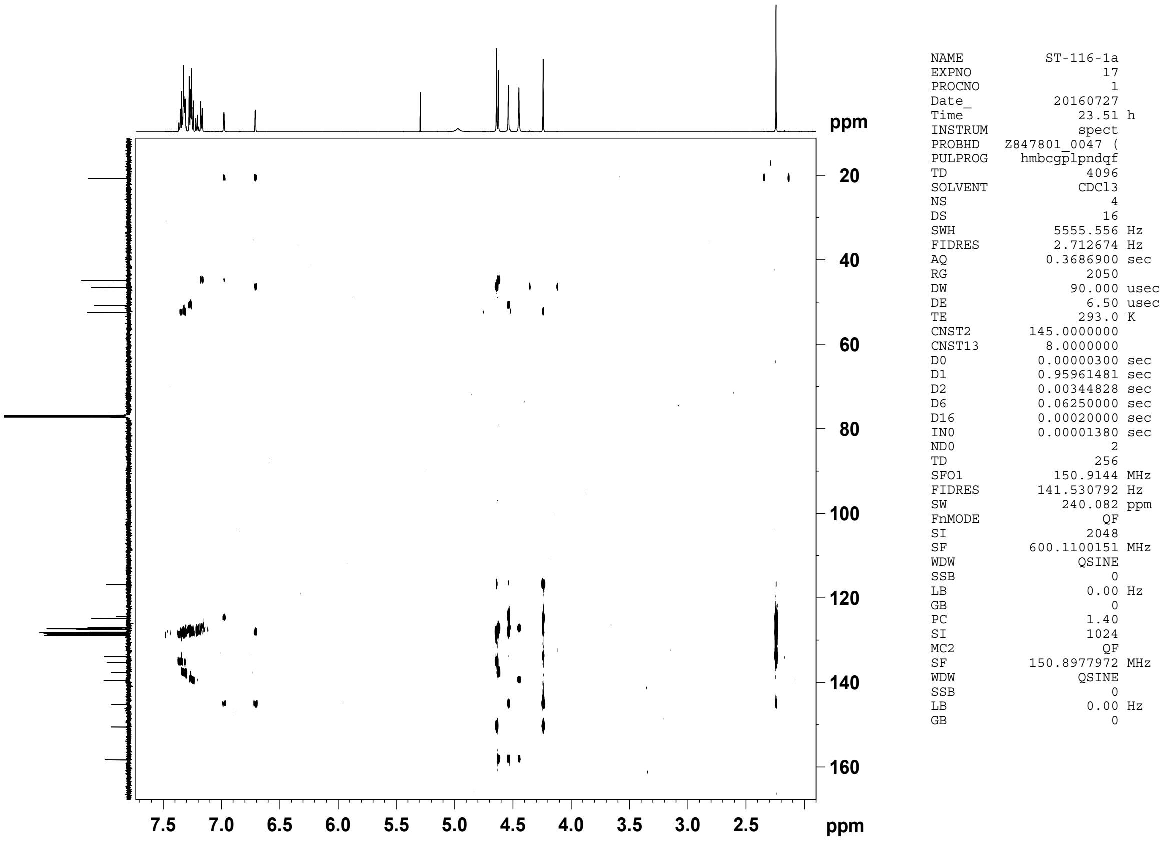

Figure S150. ^{1}H-^{13}C HMBC spectrum of compound **7bb**.

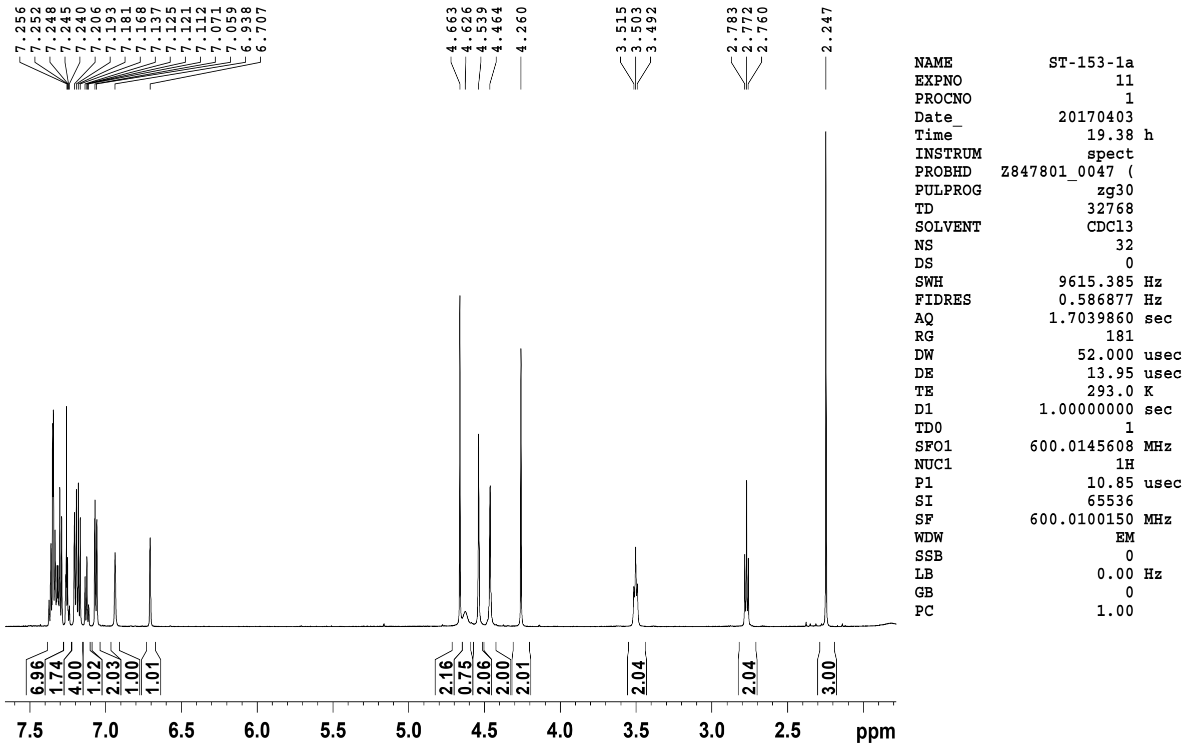

Figure S151. ^{1}H NMR spectrum of compound **7bc**.

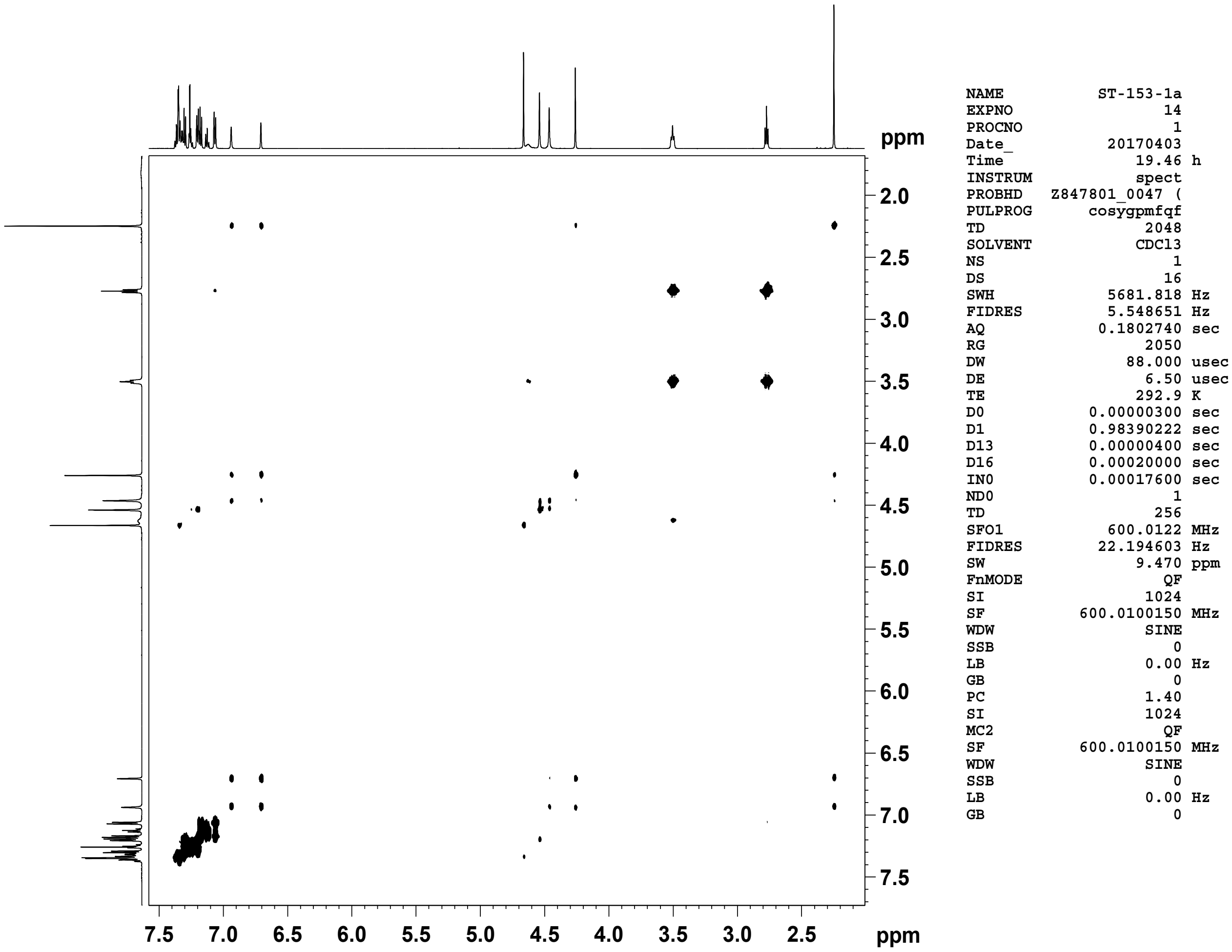

Figure S152. ^{1}H-^{1}H COSY spectrum of compound **7bc**.

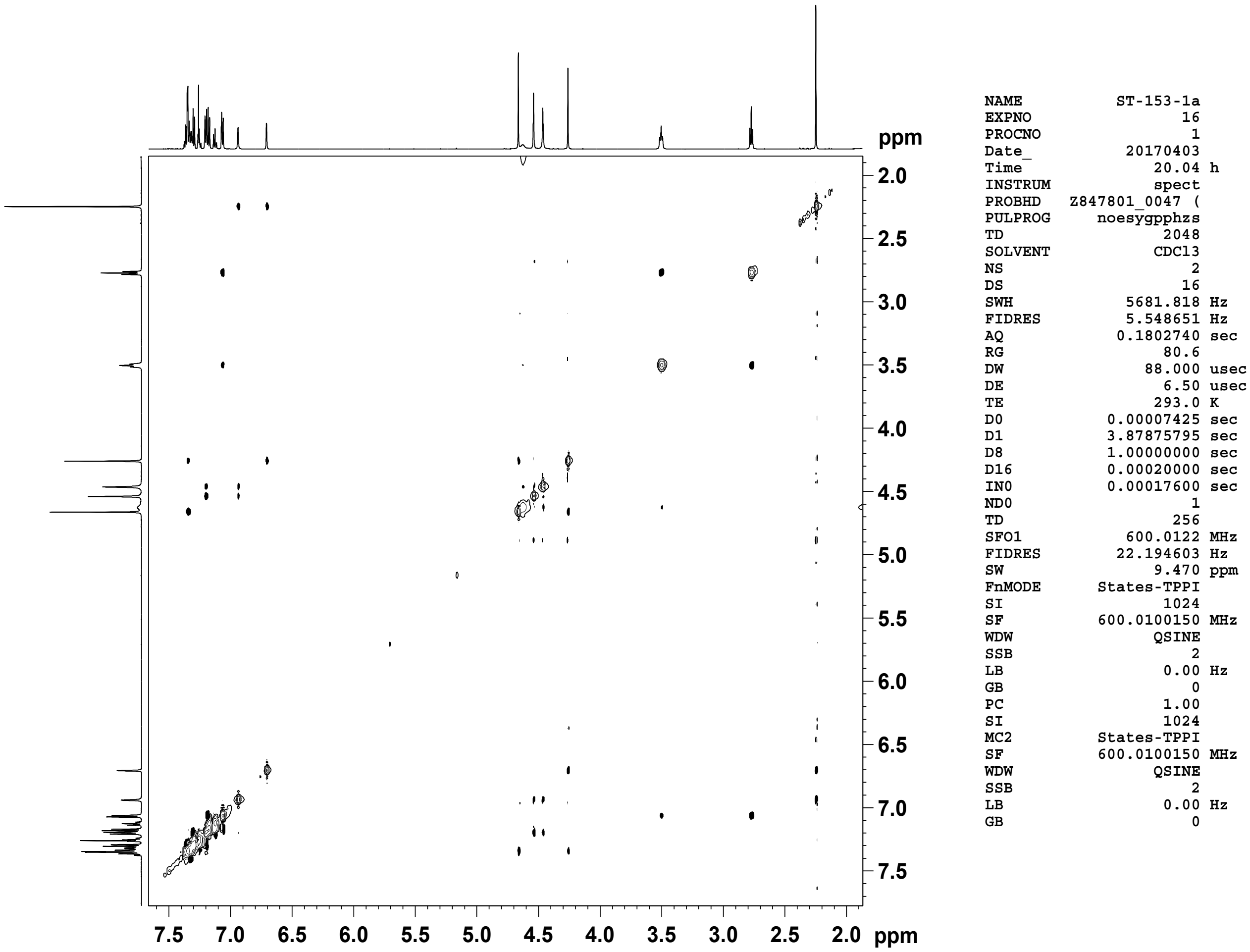

Figure S153. ^1H-^1H NOESY spectrum of compound **7bc**.

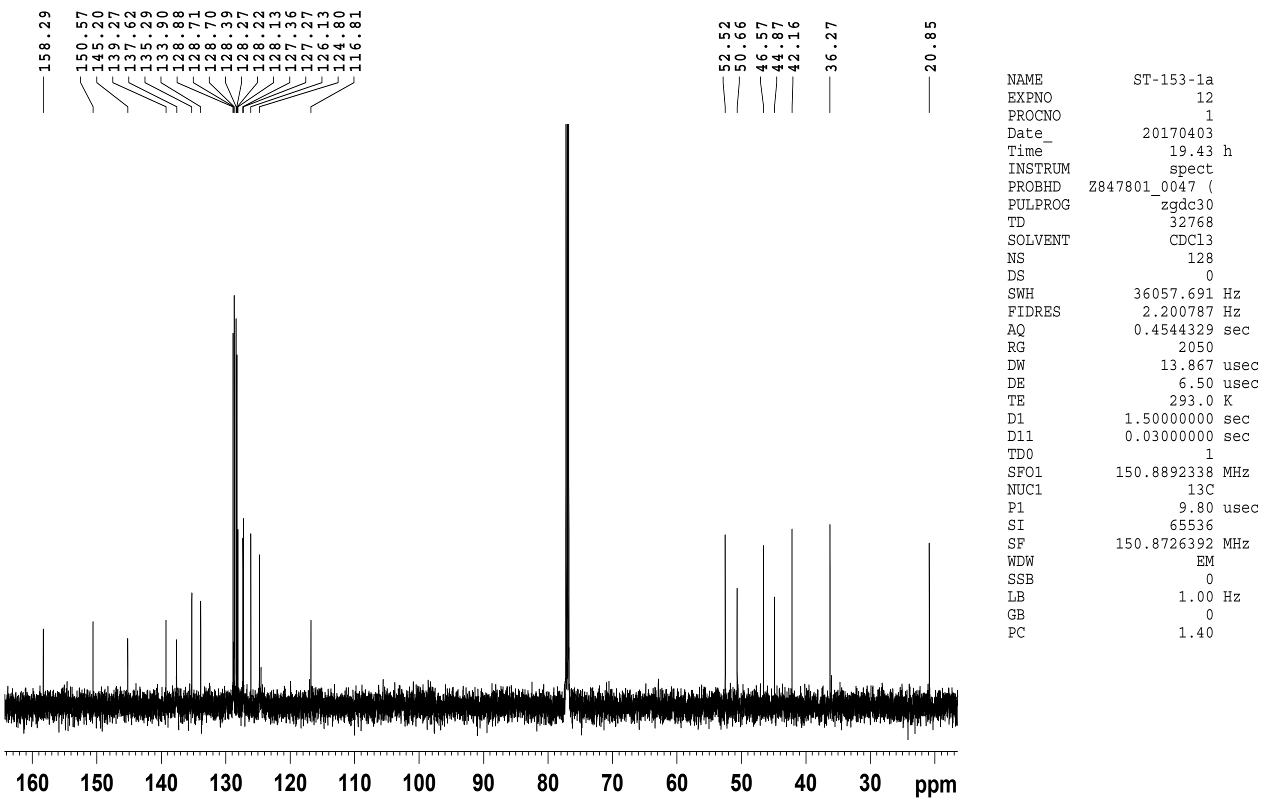

Figure S154. ^{13}C NMR spectrum of compound **7bc**.

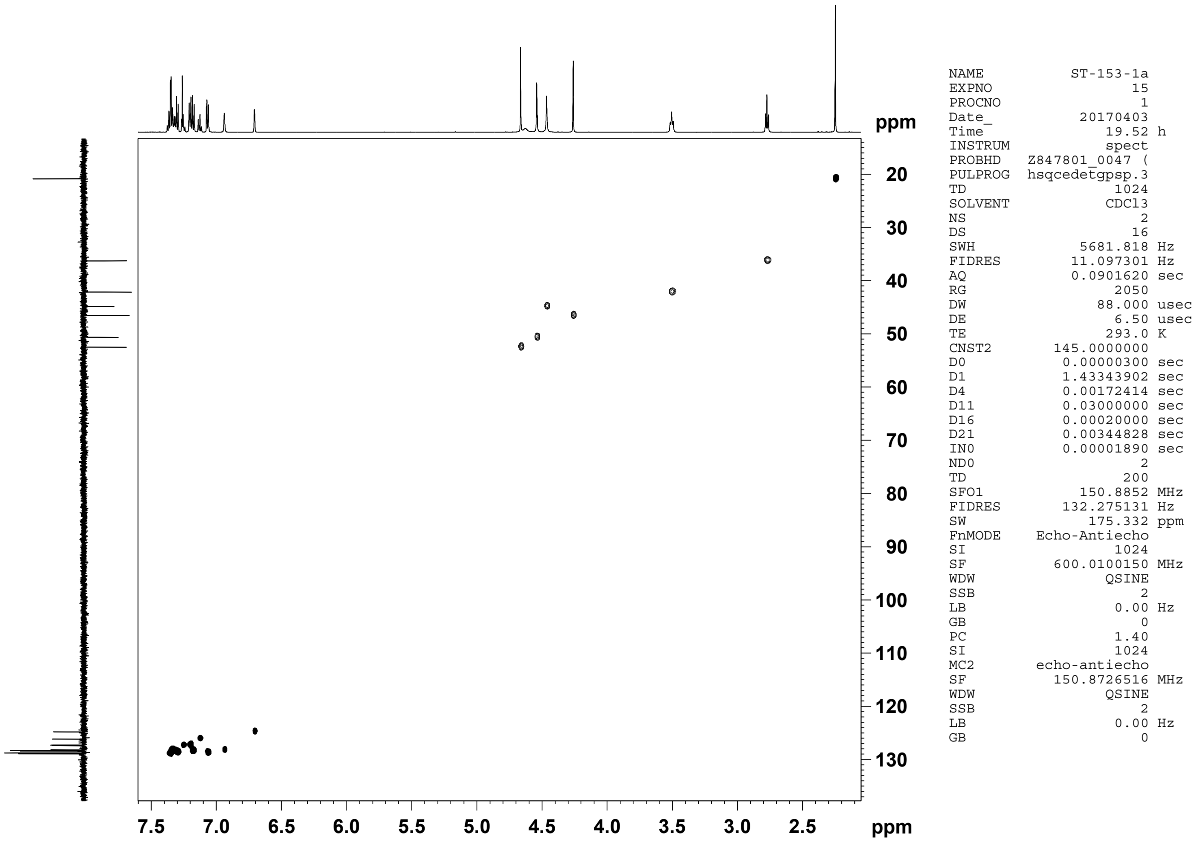

Figure S155. ^{1}H-^{13}C HSQC spectrum of compound **7bc**.

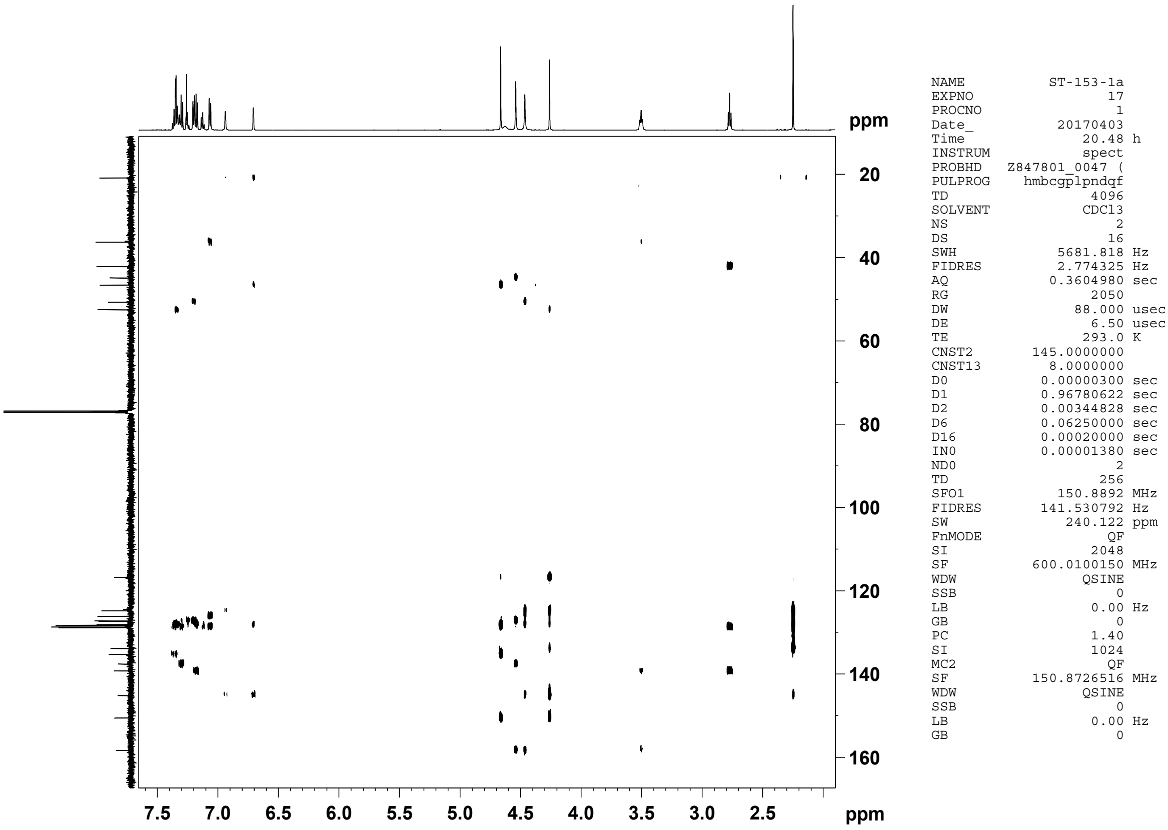

Figure S156. ^{1}H-^{13}C HMBC spectrum of compound **7bc**.

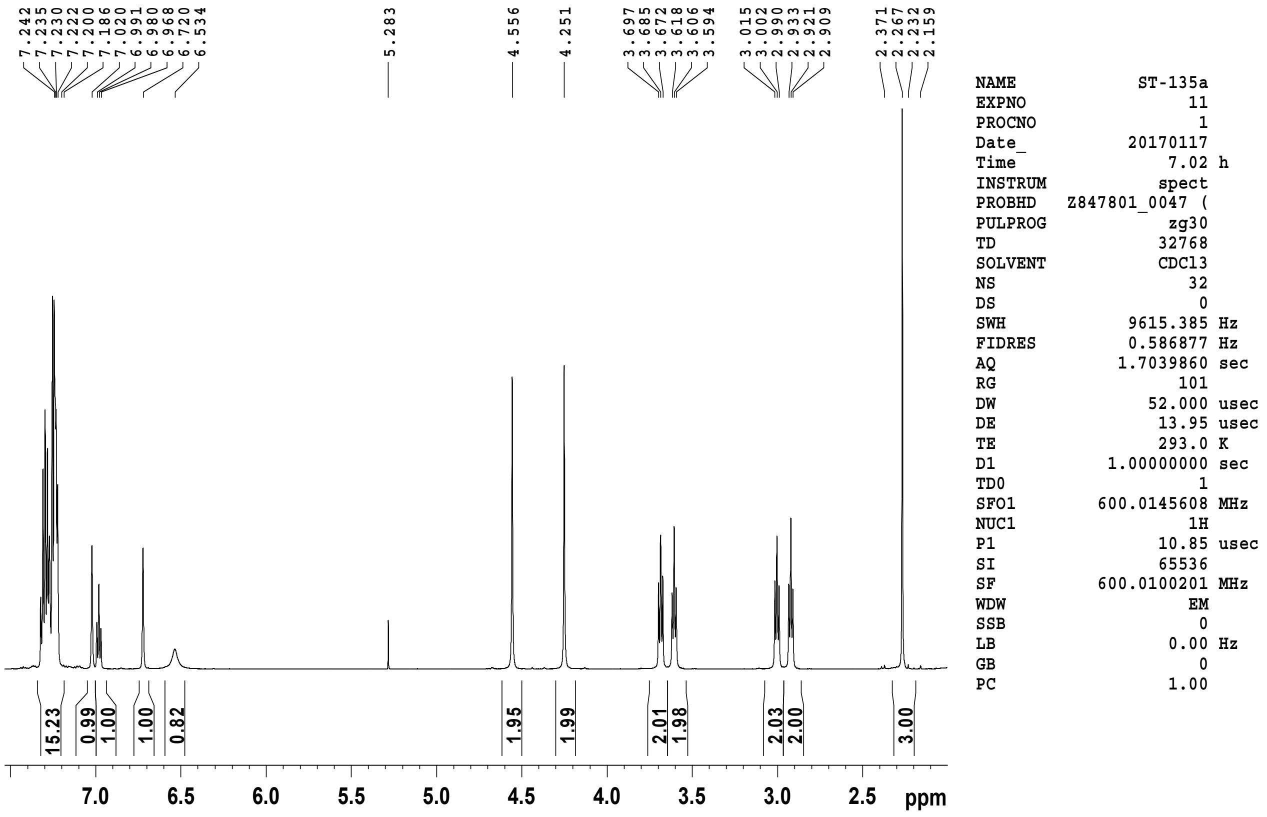

Figure S157. ^{1}H NMR spectrum of compound **7ca**.

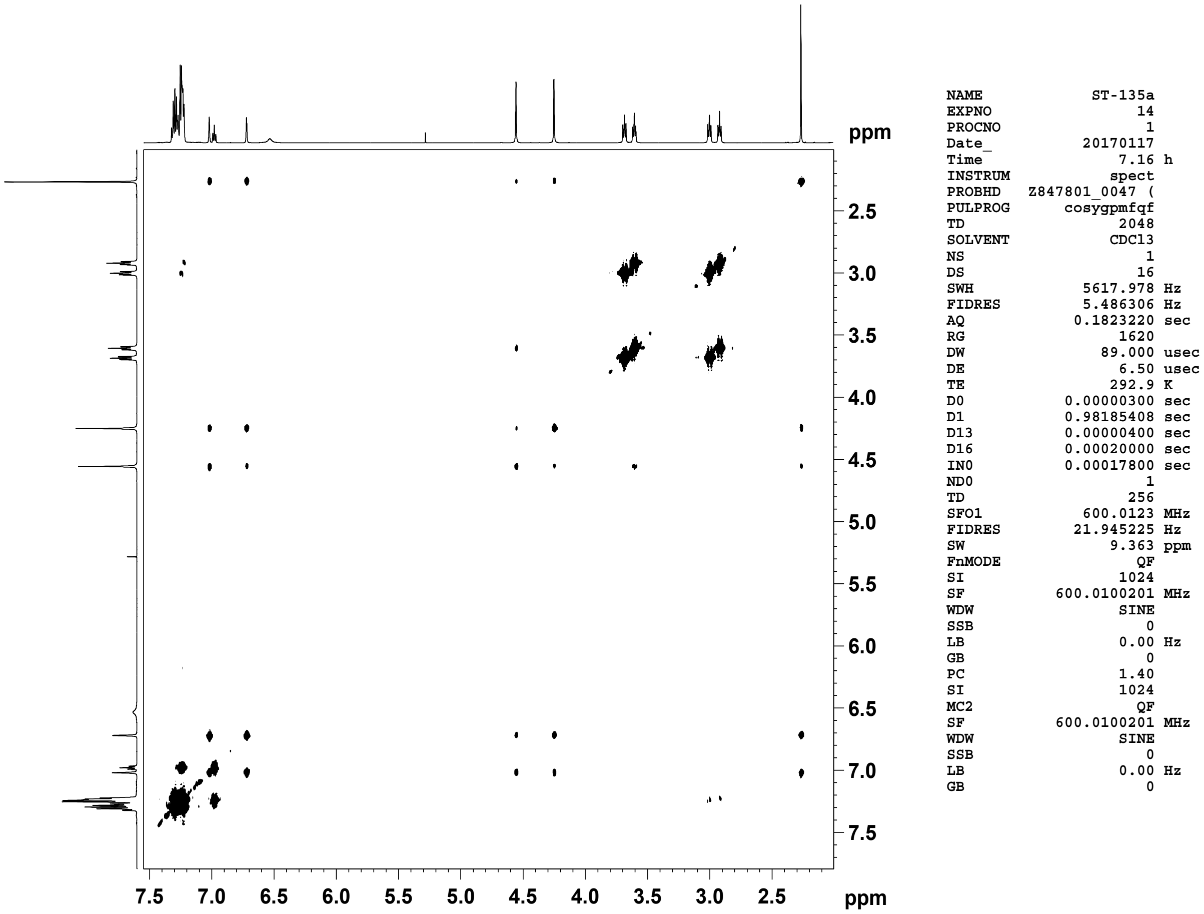

Figure S158. ^{1}H-^{1}H COSY spectrum of compound **7ca**.

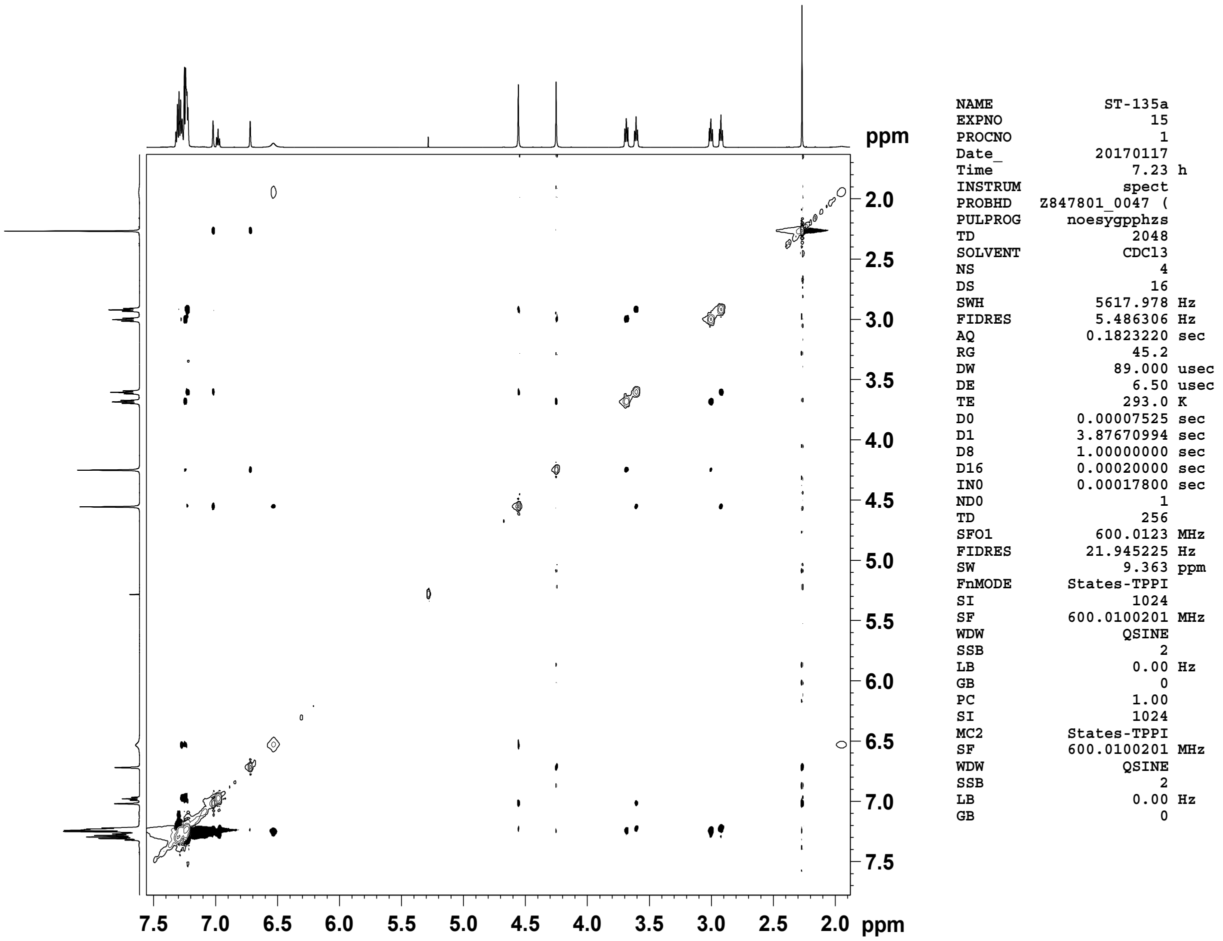

Figure S159. ^1H-^1H NOESY spectrum of compound **7ca**.

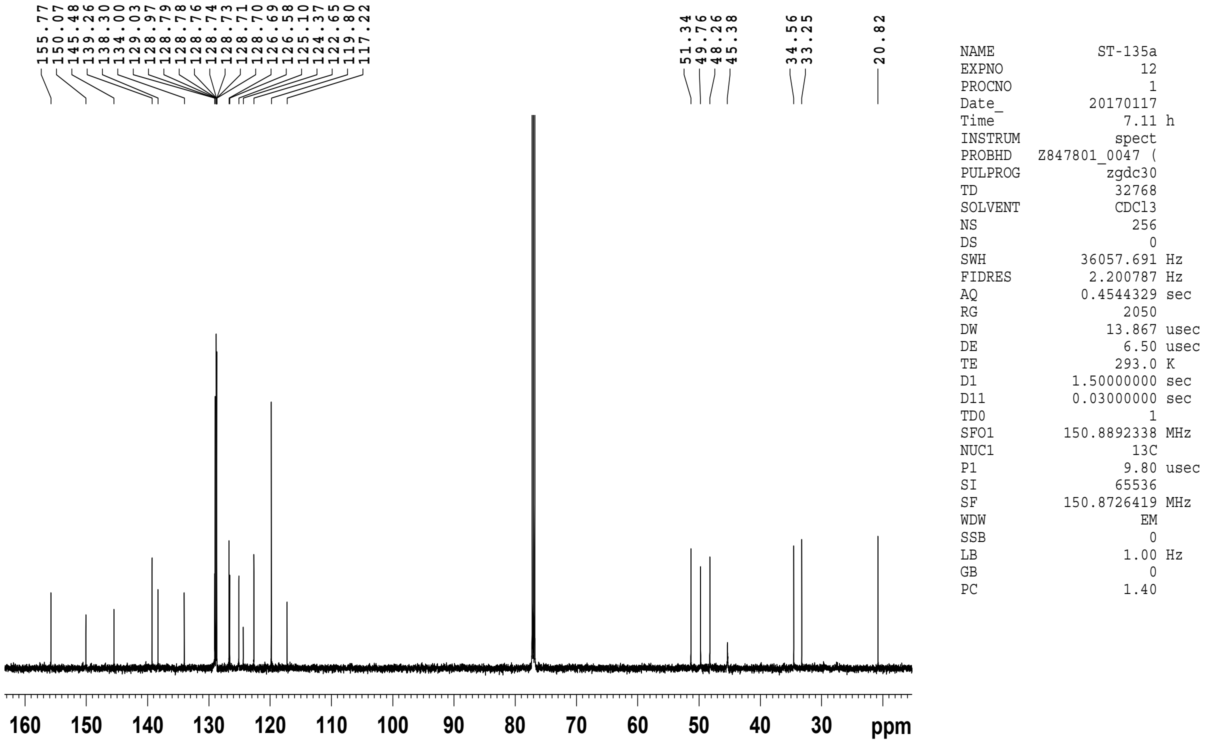

Figure S160. ^{13}C NMR spectrum of compound **7ca**.

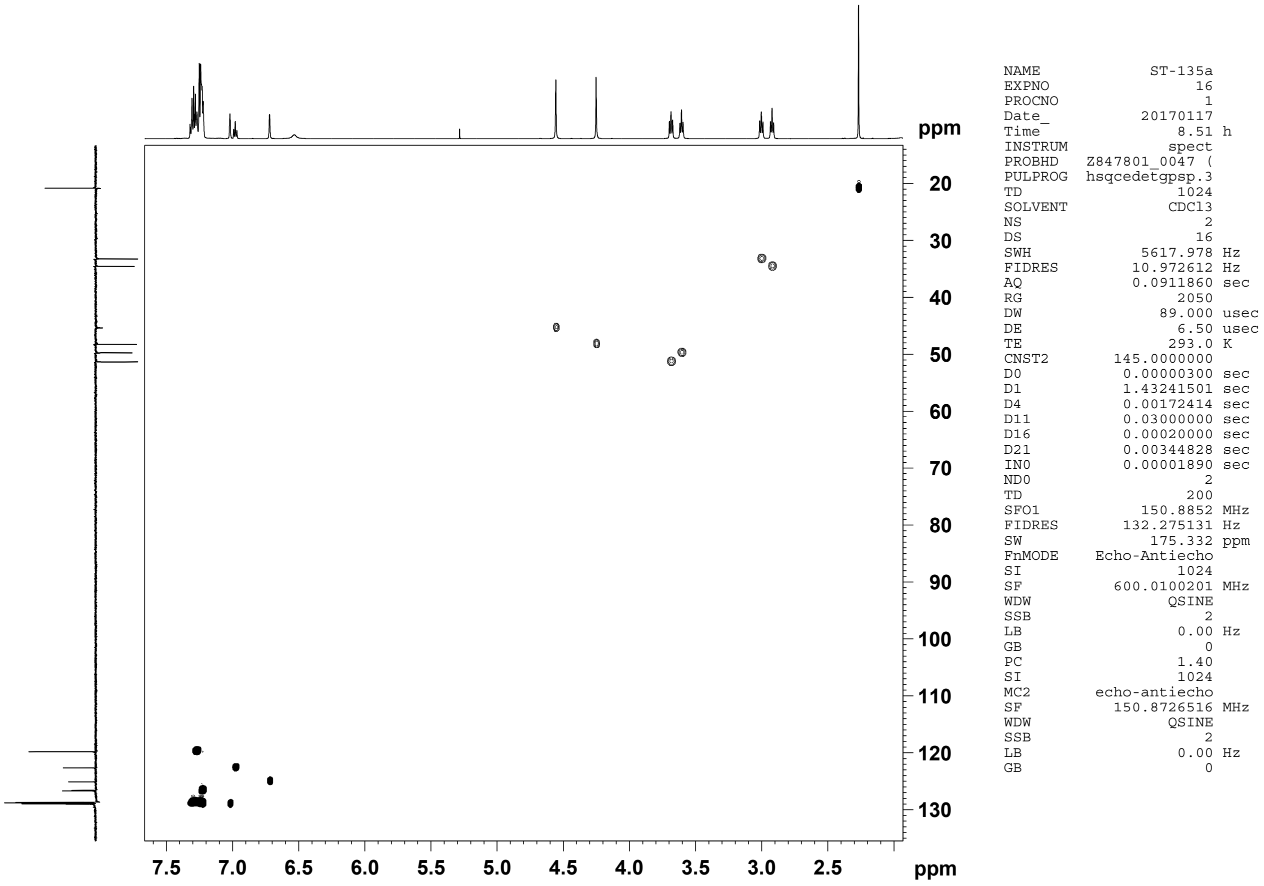

Figure S161. ^{1}H-^{13}C HSQC spectrum of compound **7ca**.

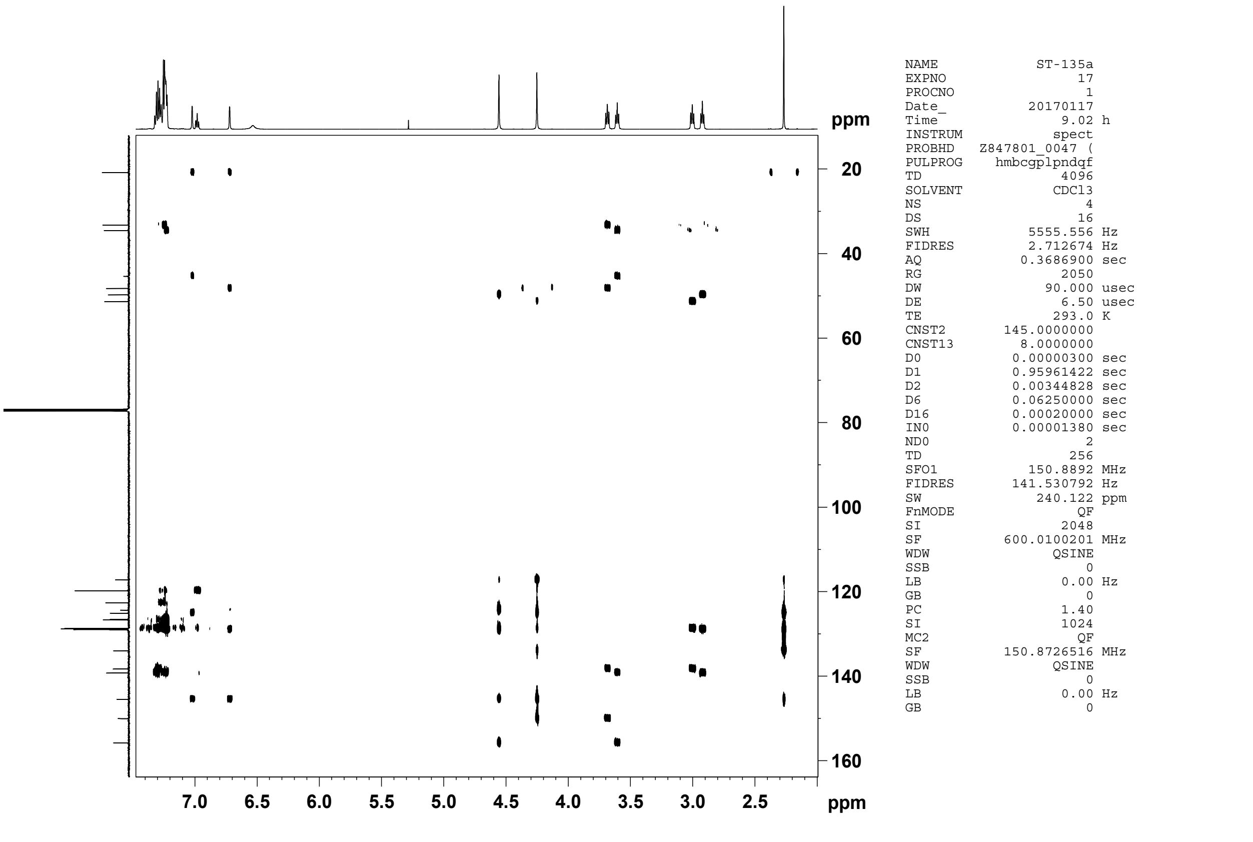

Figure S162. ^{1}H-^{13}C HMBC spectrum of compound **7ca**.

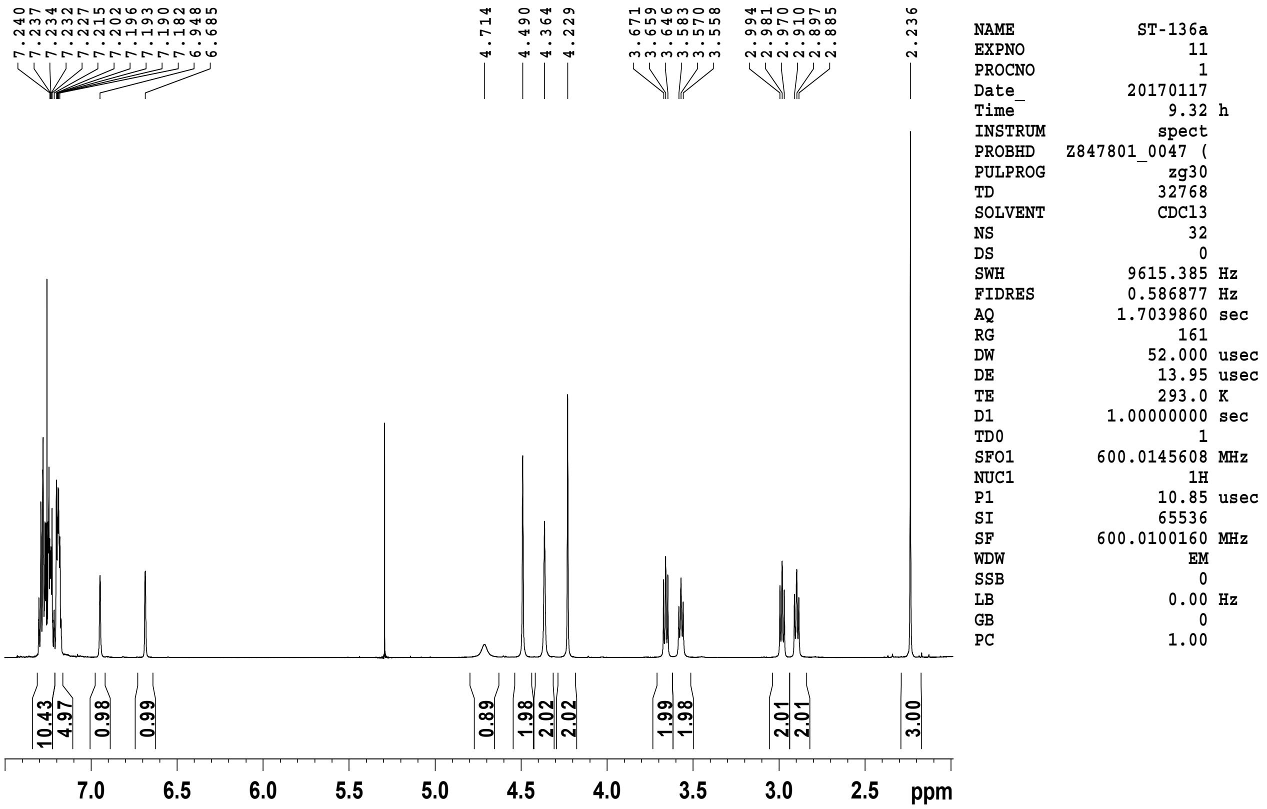

Figure S163. ^{1}H NMR spectrum of compound **7cb**.

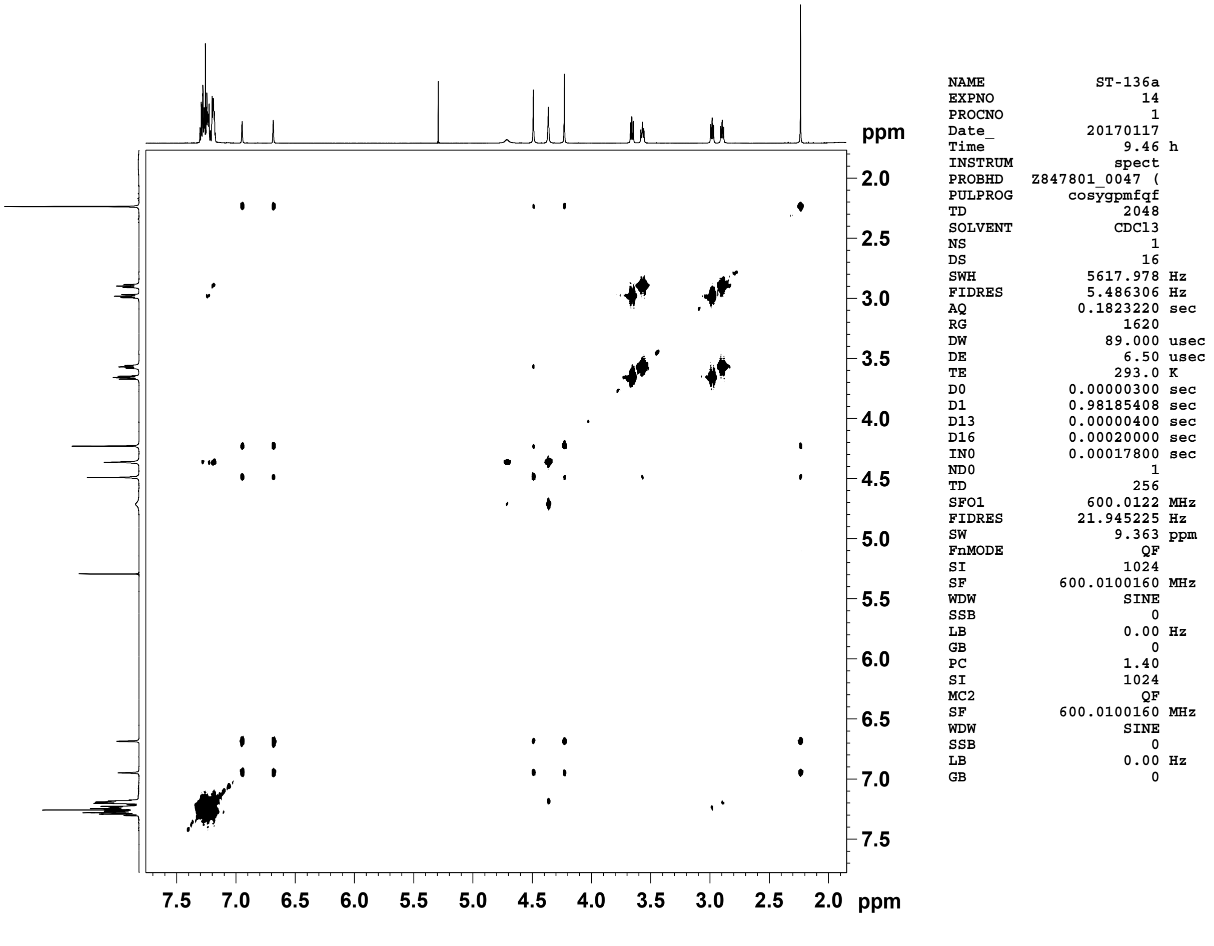

Figure S164. ^{1}H-^{1}H COSY spectrum of compound **7cb**.

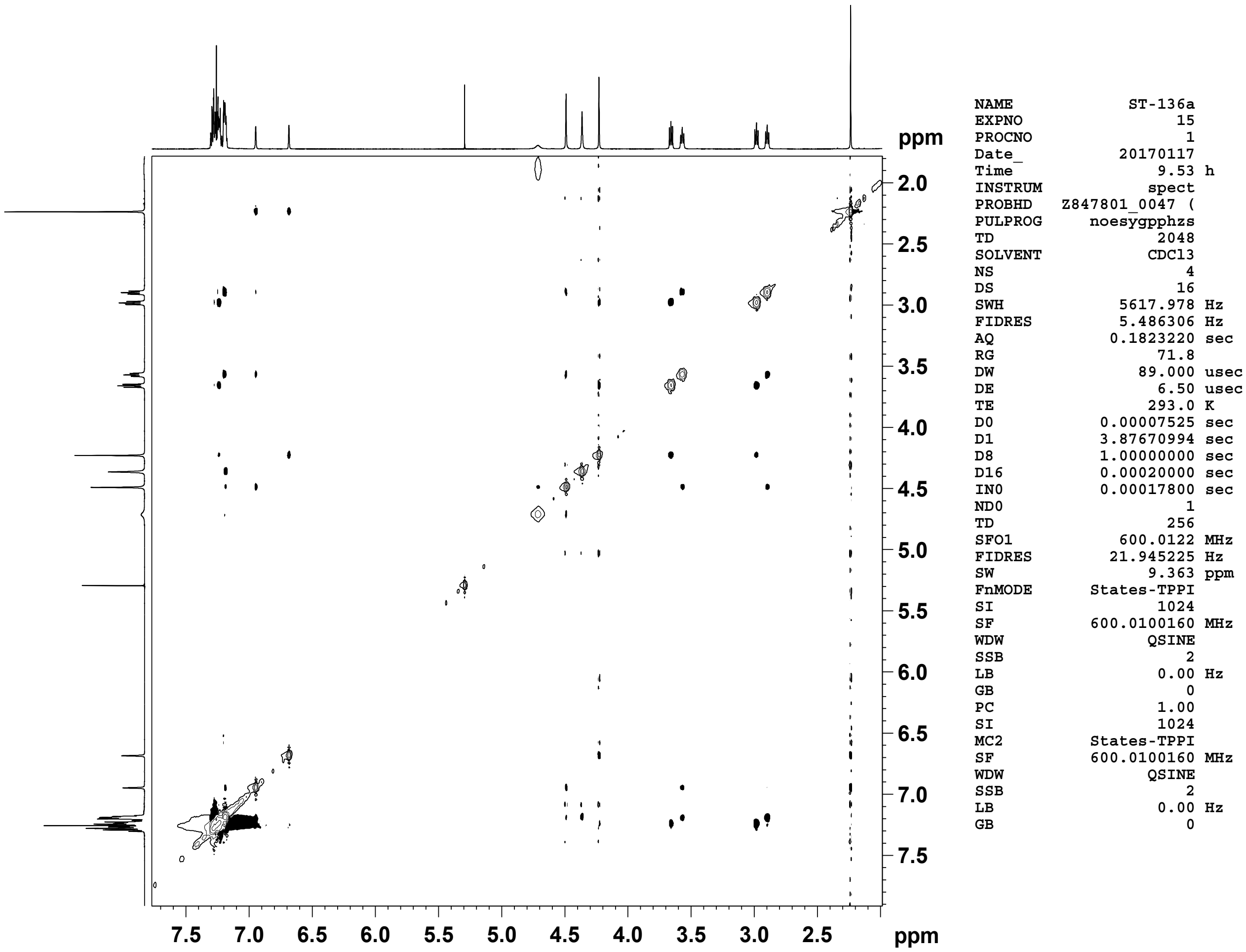

Figure S165. ^{1}H-^{1}H NOESY spectrum of compound **7cb**.

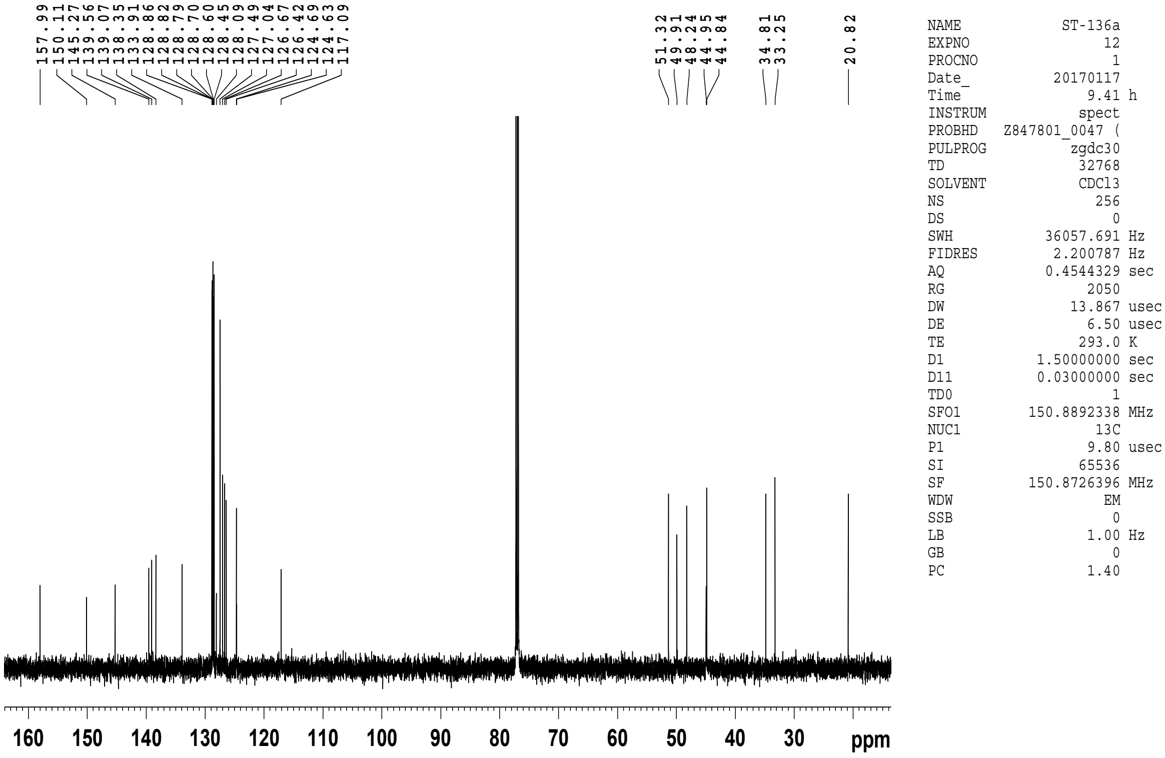

Figure S166. ^{13}C NMR spectrum of compound **7cb**.

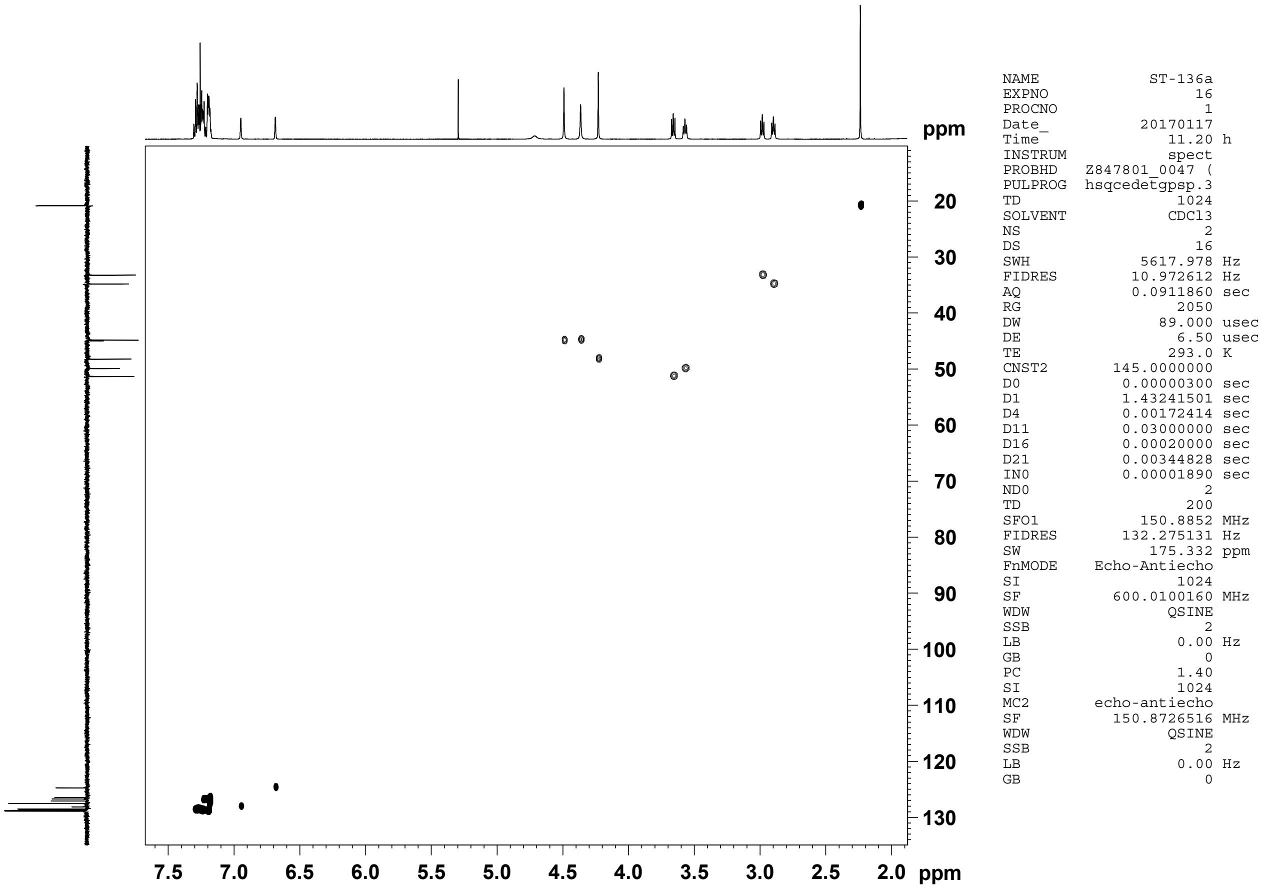

Figure S167. ^{1}H-^{13}C HSQC spectrum of compound **7cb**.

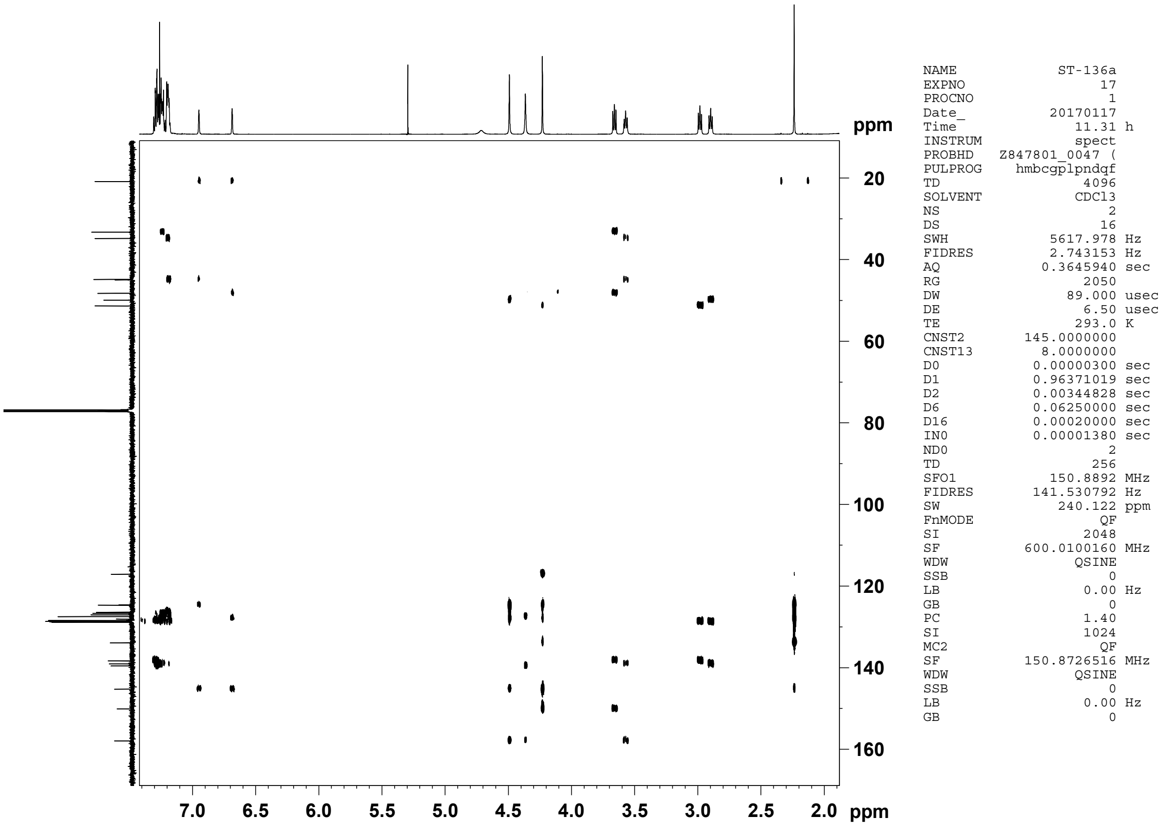

Figure S168. ^{1}H-^{13}C HMBC spectrum of compound **7cb**.

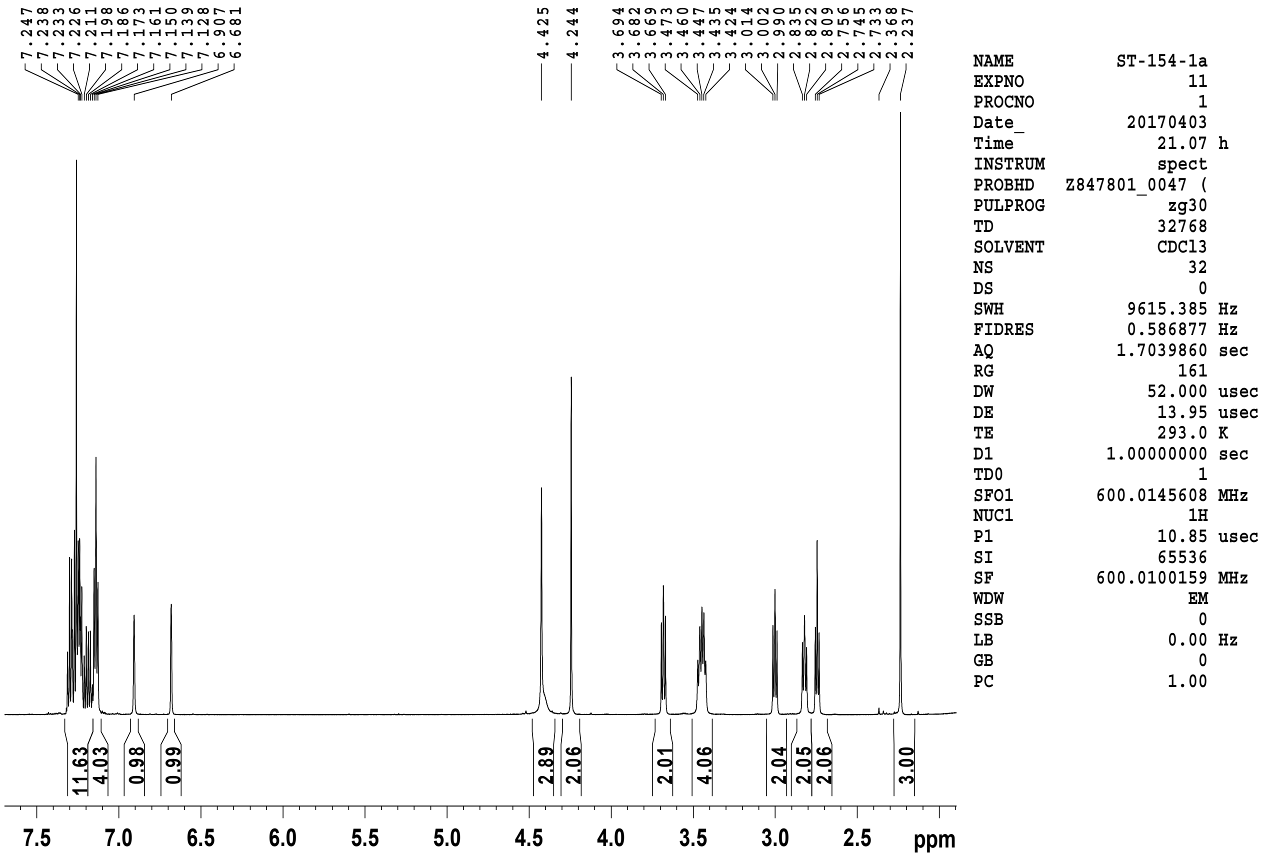

Figure S169. ^{1}H NMR spectrum of compound **7cc**.

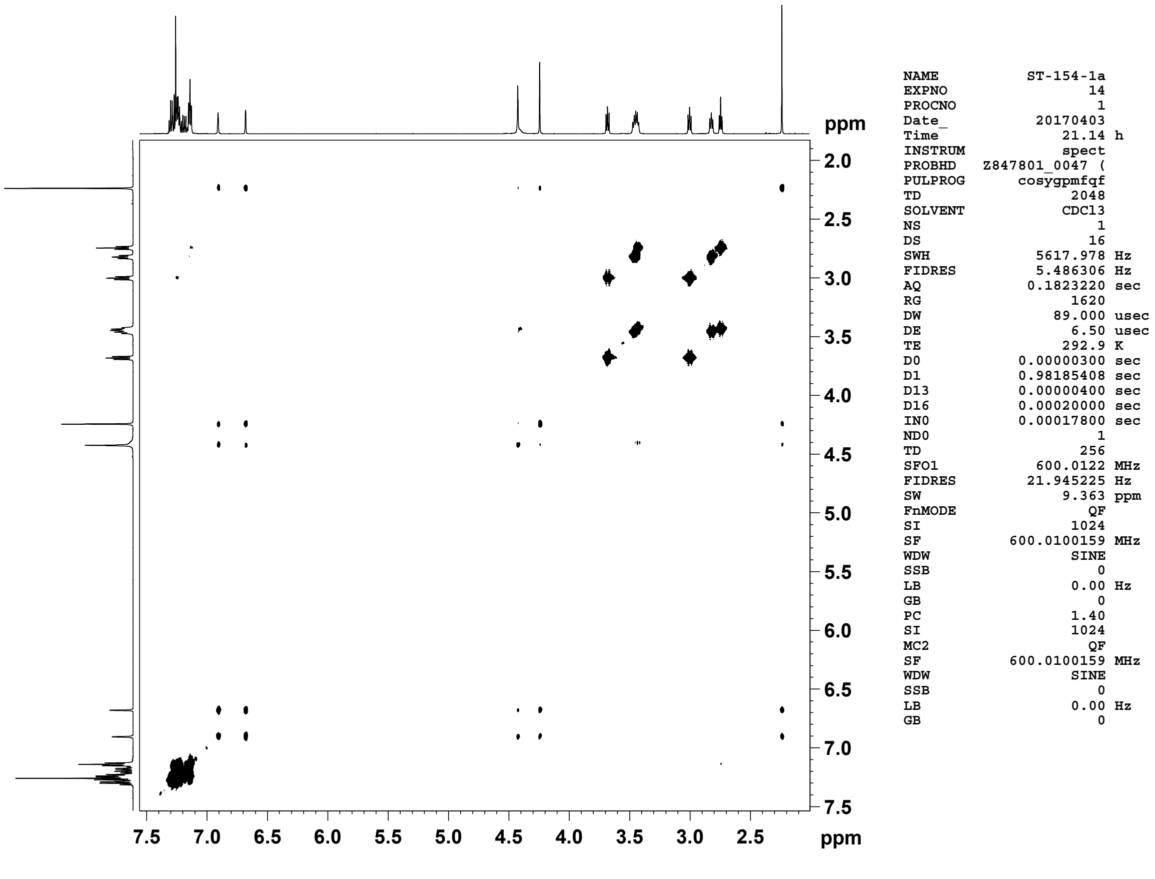

Figure S170. ^{1}H-^{1}H COSY spectrum of compound **7cc**.

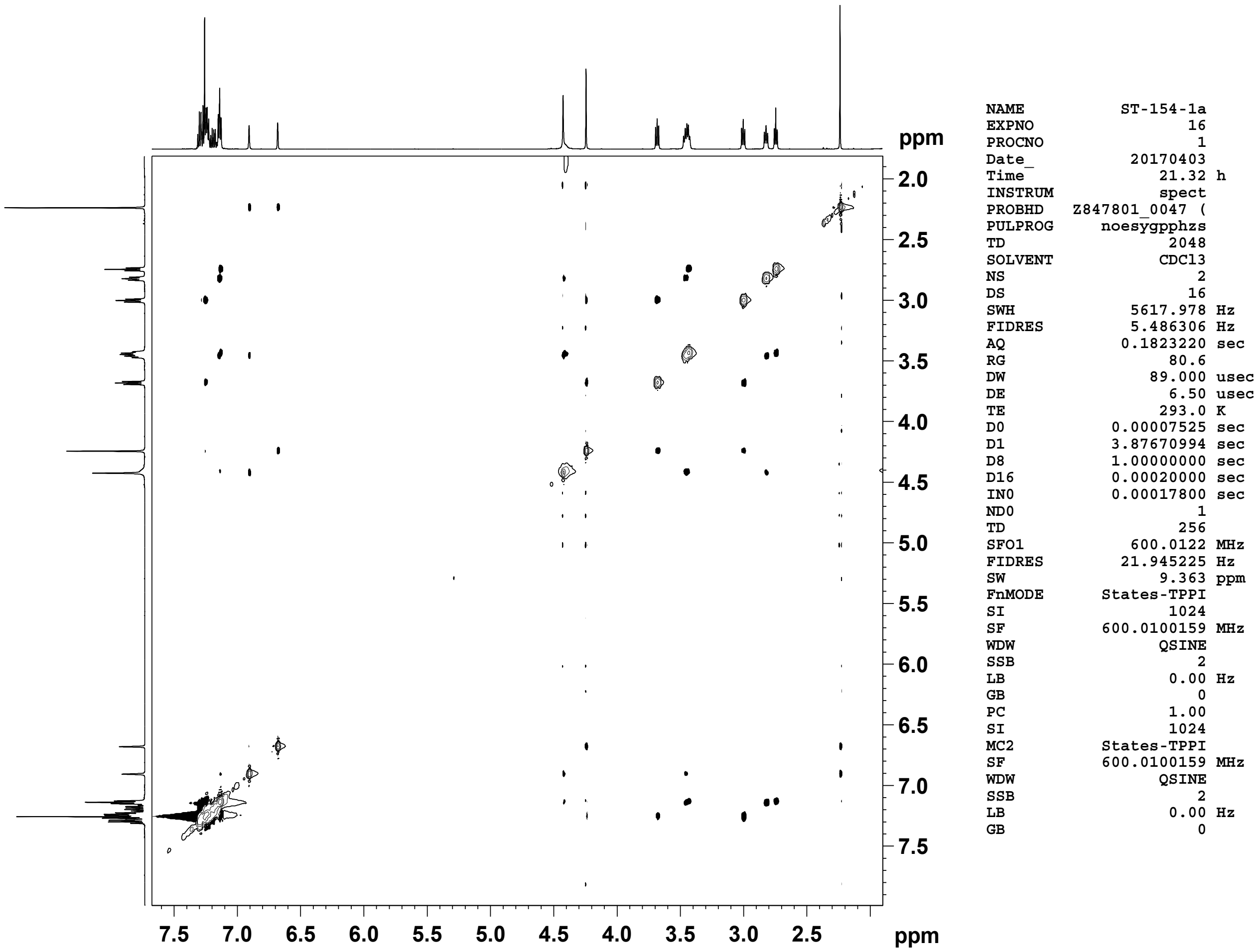

Figure S171. ^{1}H-^{1}H NOESY spectrum of compound **7cc**.

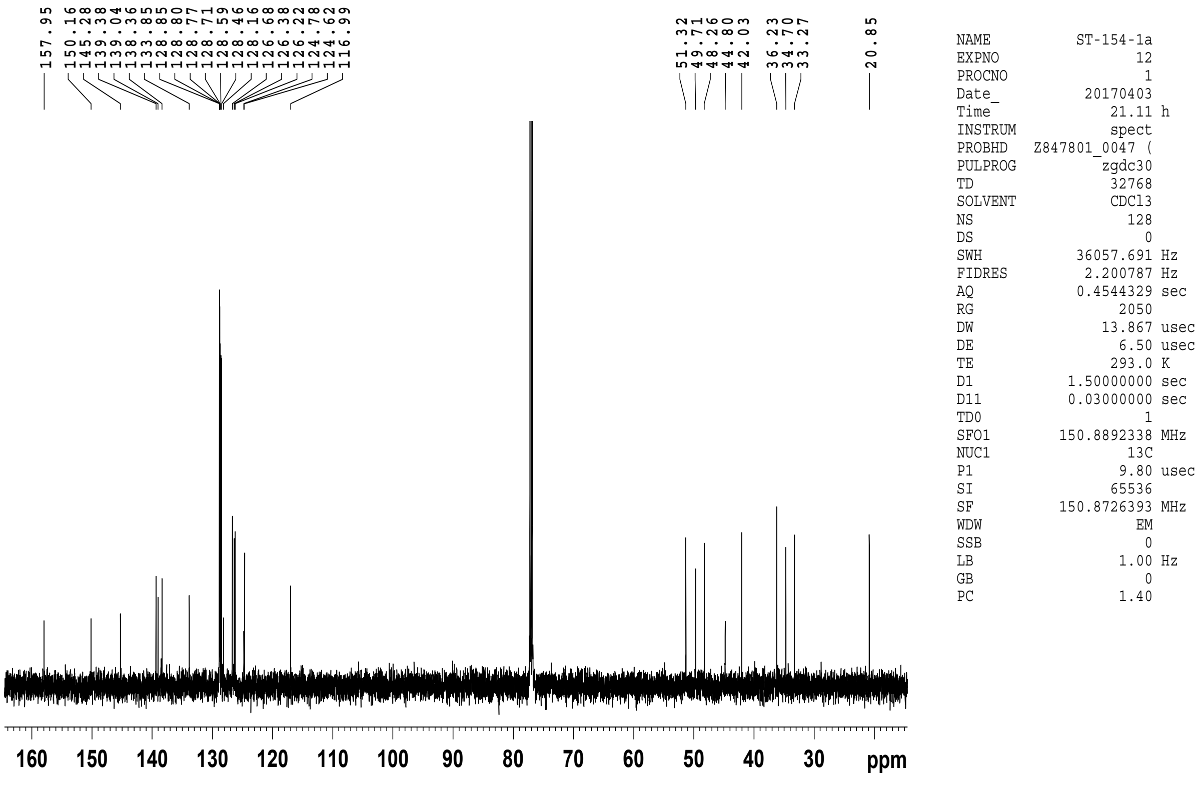

Figure S172. ^{13}C NMR spectrum of compound **7cc**.

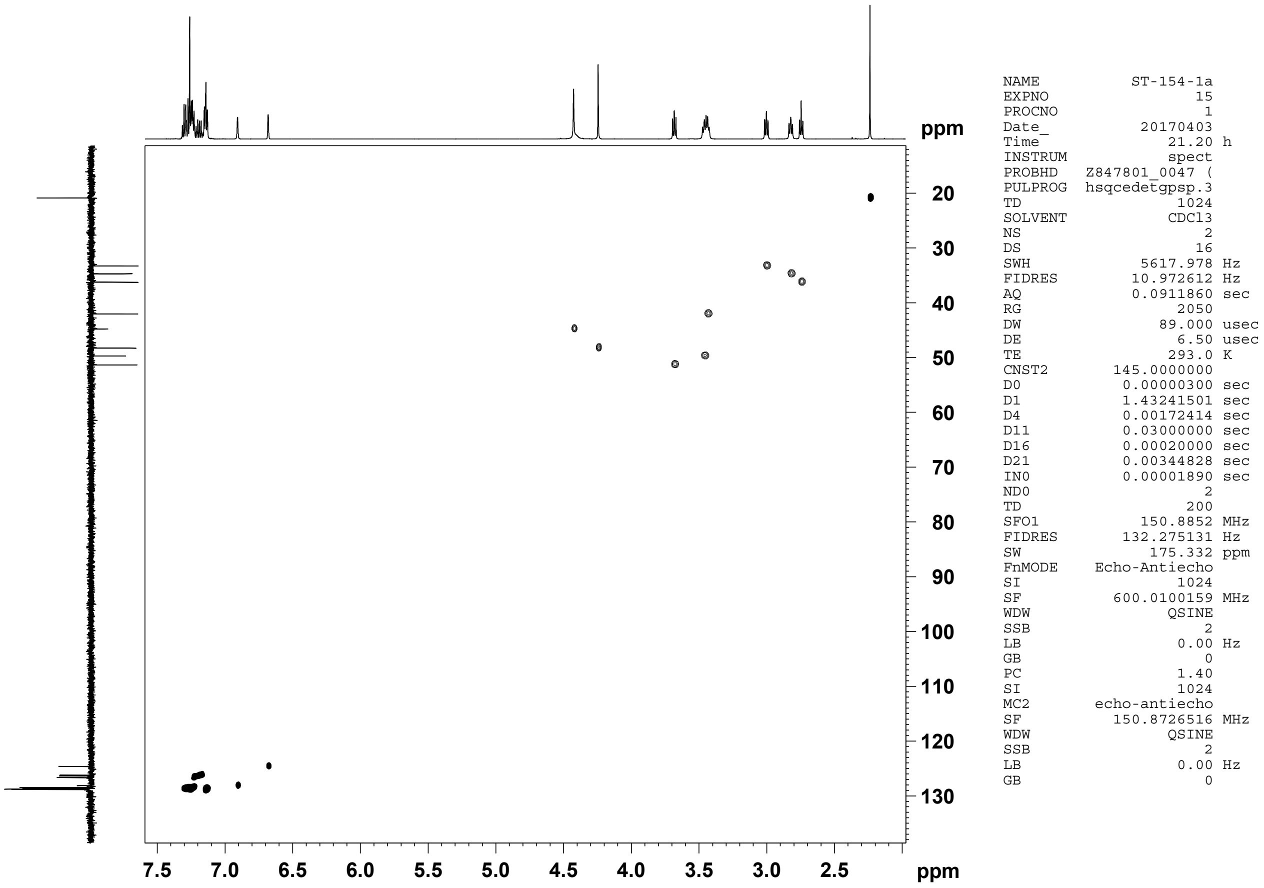

Figure S173. ^{1}H-^{13}C HSQC spectrum of compound **7cc**.

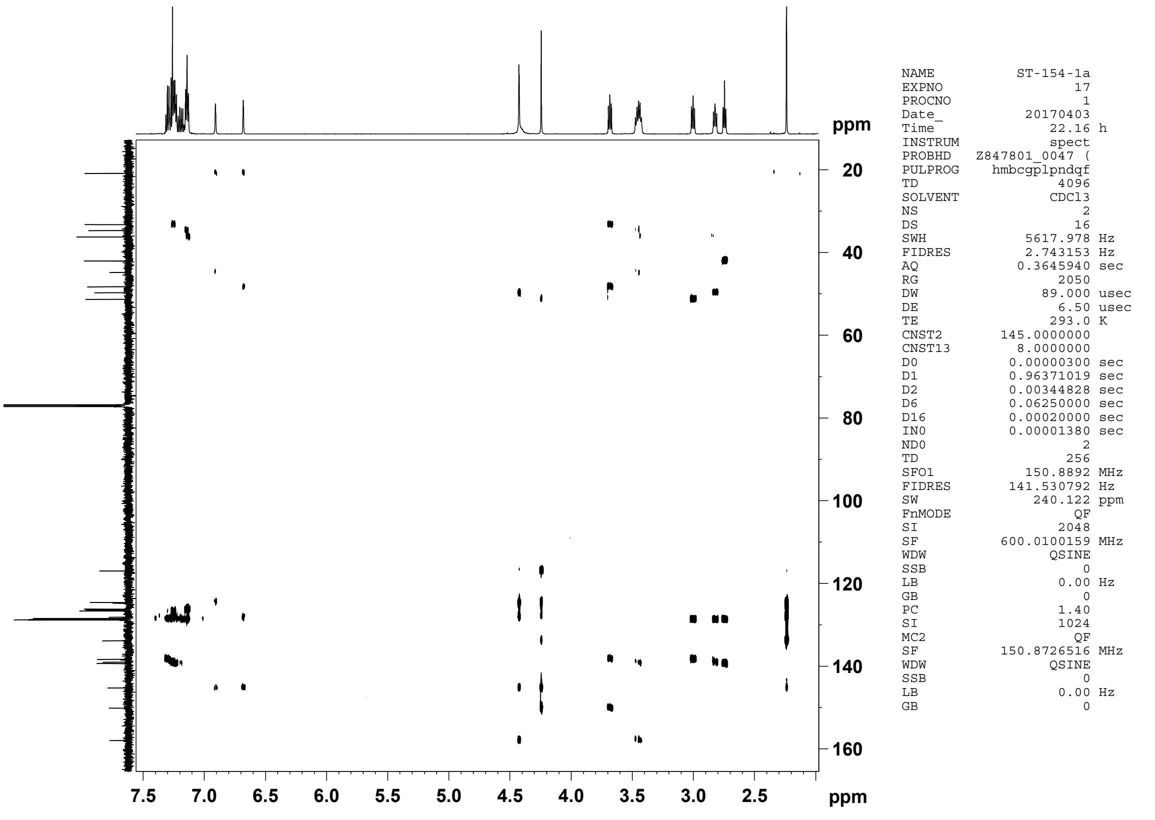

Figure S174. ^{1}H-^{13}C HMBC spectrum of compound **7cc**.

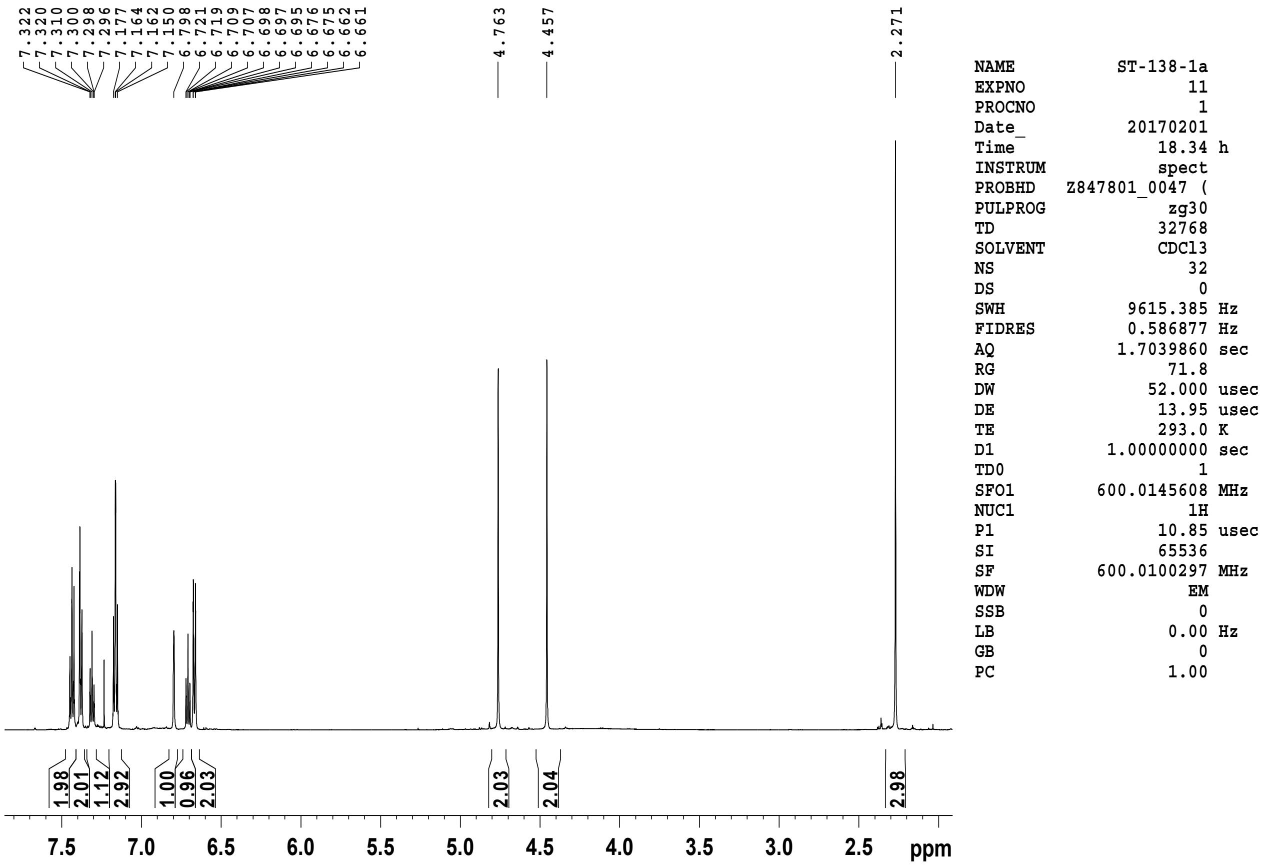

Figure S175. ^{1}H NMR spectrum of compound **8a**.

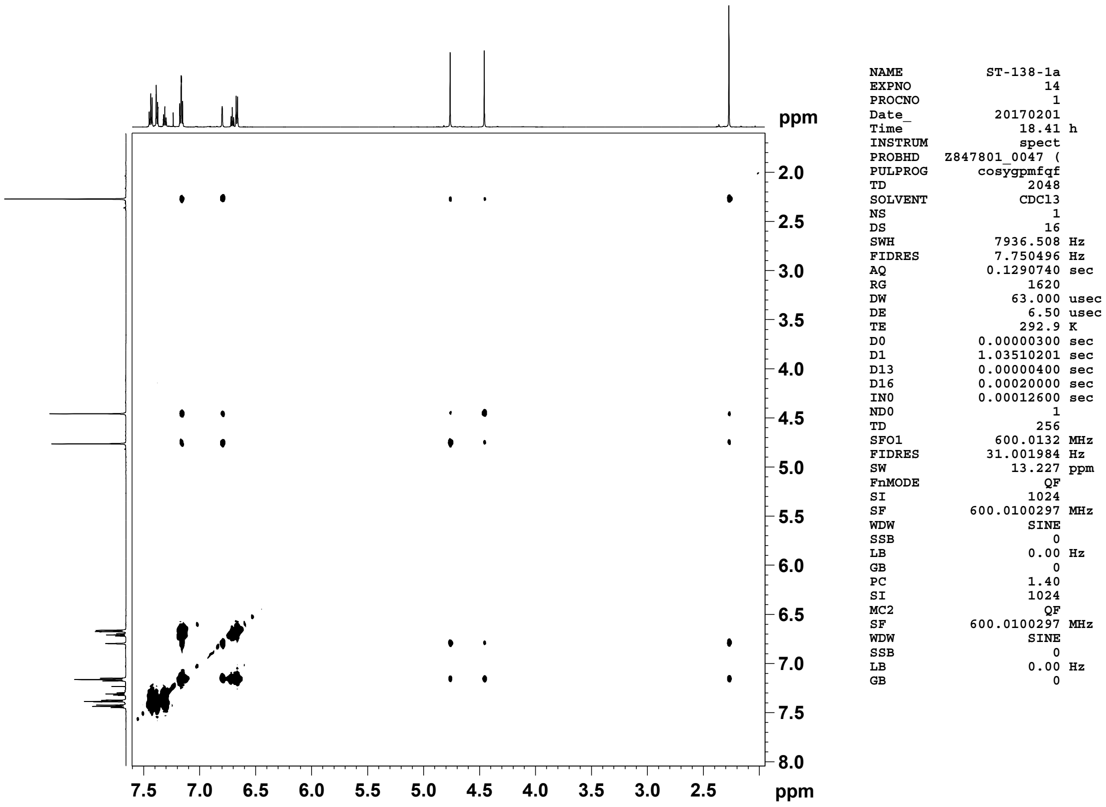

Figure S176. ^{1}H-^{1}H COSY spectrum of compound **8a**.

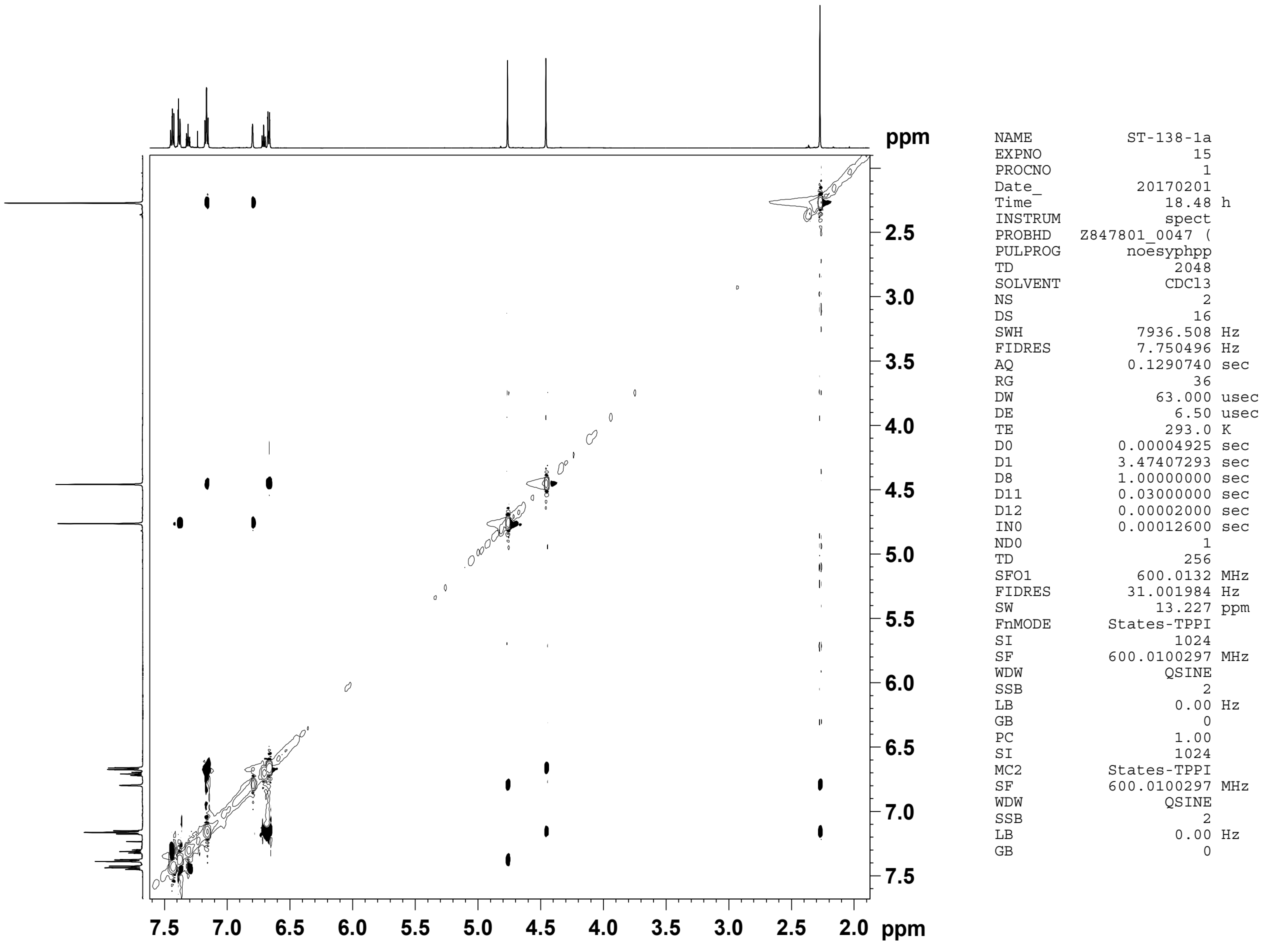

Figure S177. ^1H-^1H NOESY spectrum of compound **8a**.

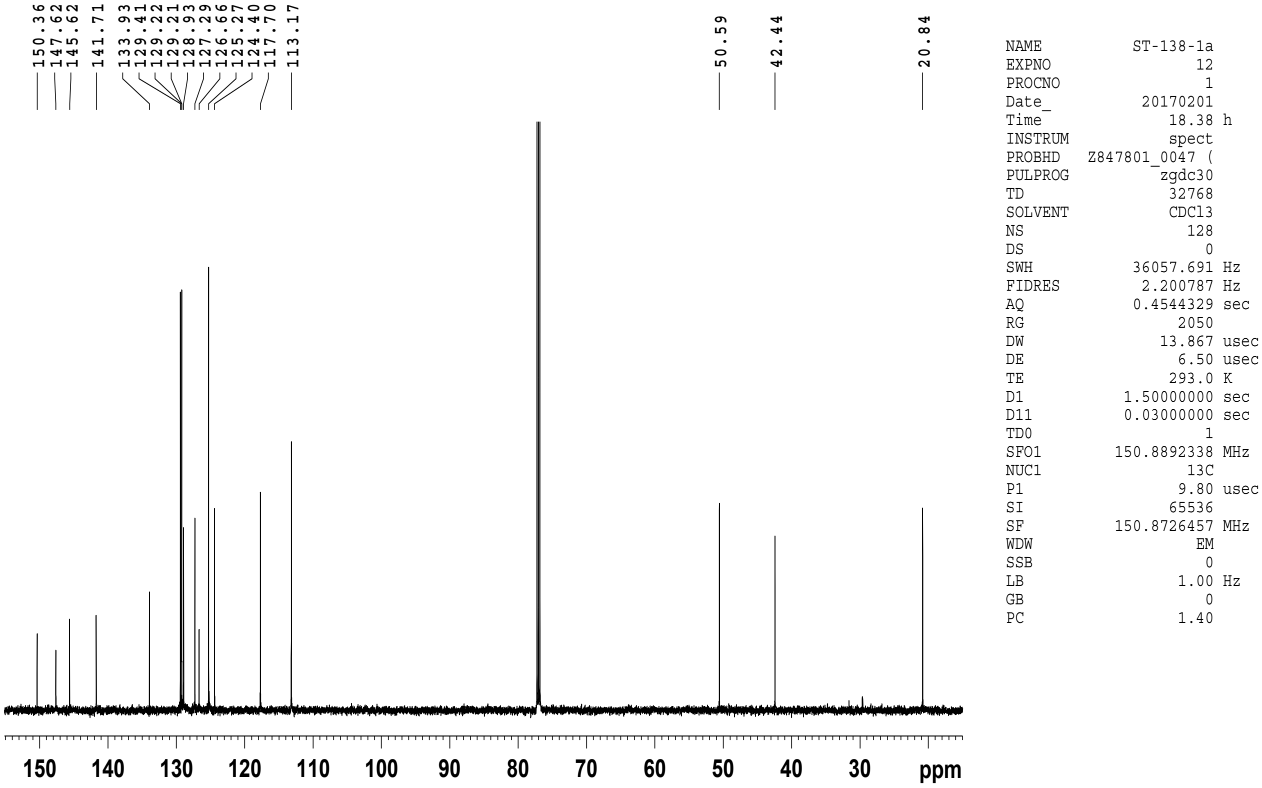

Figure S178. ^{13}C NMR spectrum of compound **8a**.

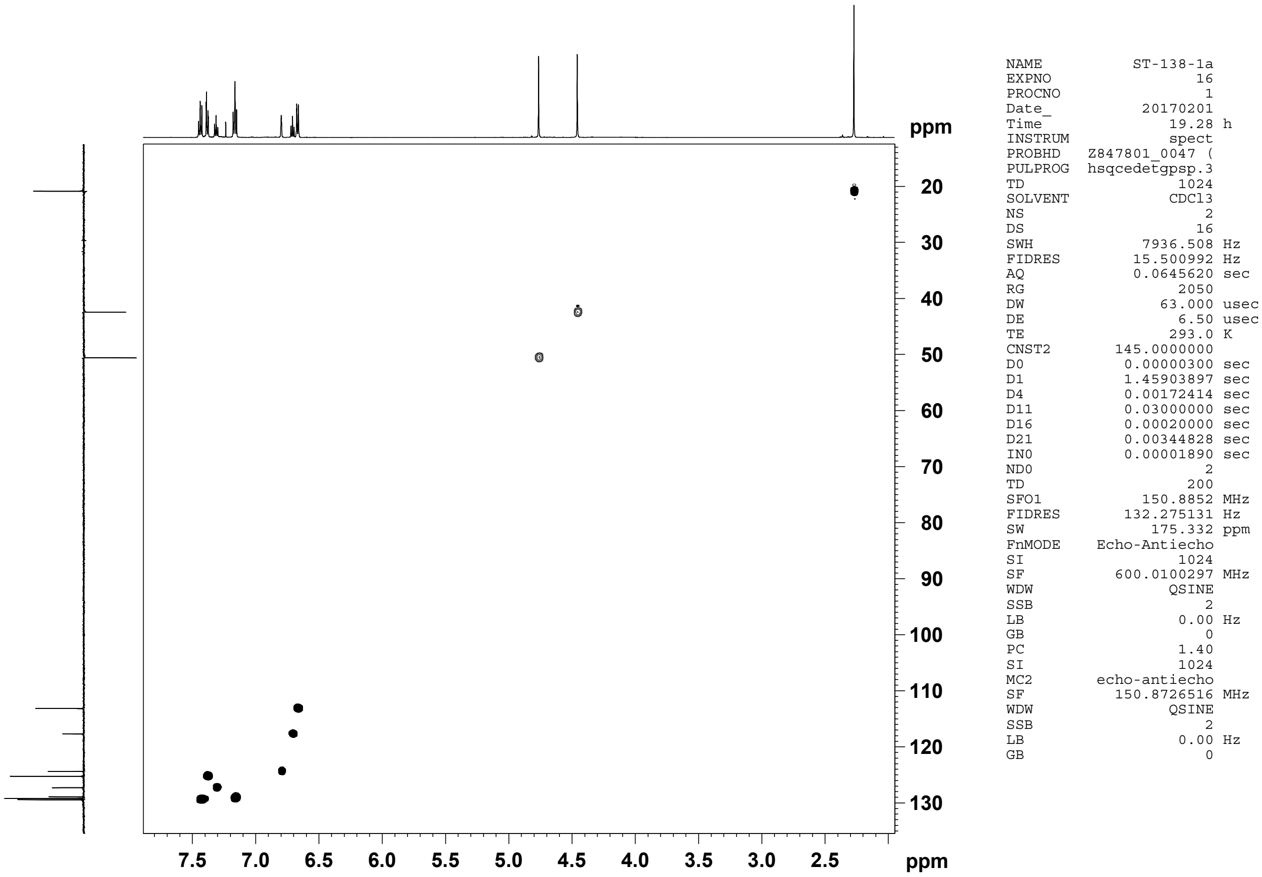

Figure S179. ^{1}H-^{13}C HSQC spectrum of compound **8a**.

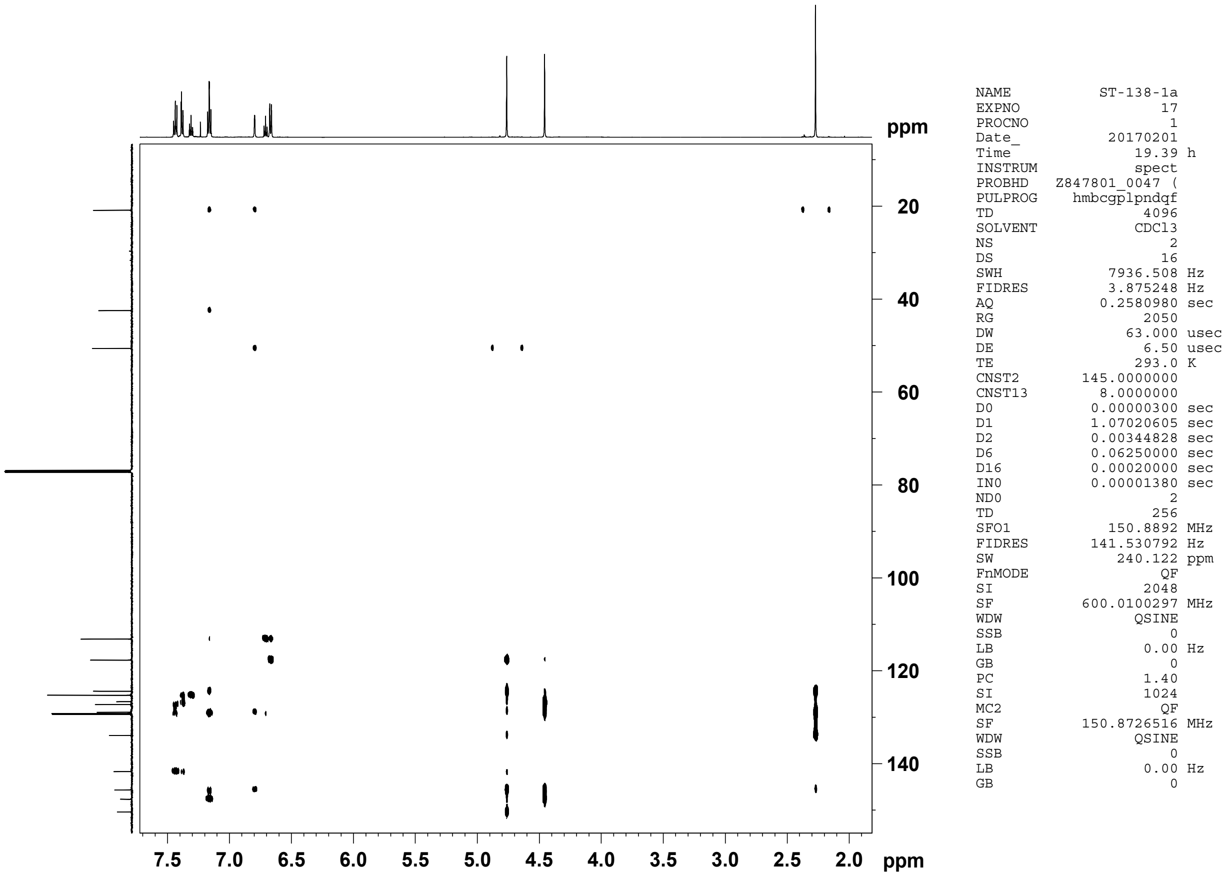

Figure S180. ^{1}H-^{13}C HMBC spectrum of compound **8a**.
